# Super Users’ Reported Best Practices for Coordinating Proactive Integrated Use of Virtual Health Care Resources: Prospective Concurrent Mixed Methods Human-Centered Design Study

**DOI:** 10.2196/81414

**Published:** 2025-11-14

**Authors:** Jolie N Haun, Rachel C Benzinger, Julie McMahon-Grenz, Lisa M Ballistrea, S Angel Klanchar, Rebecca S Bolinski, Justin T McDaniel

**Affiliations:** 1 Research and Development Service James A Haley Veterans' Hospital Tampa, FL United States; 2 Division of Epidemiology Department of Internal Medicine University of Utah Salt Lake City, UT United States; 3 Department of Population Science and Policy School of Medicine Southern Illinois University Springfield, IL United States; 4 School of Human Sciences Southern Illinois University Carbondale, IL United States

**Keywords:** eHealth, medical care teams, quality improvement, remote care, remote delivery, telehealth, veteran, virtual care, virtual healthcare resources, virtual medical modality

## Abstract

**Background:**

Proactive integrated virtual health care resource (VHR) use is the self-initiated, coordinated use of applicable virtual systems as a team or an individual for coordinating timely delivery of high-quality health care. Based on literature and the purpose of this project, super users are defined as clinical team members identified by colleagues as proactive users of VHRs (ie, early adopters) who coordinate care delivery and champion resource use. Super users’ proactive integrated VHR use improves workflow and workload efficiency and supports provider uptake and promotion, which increases patient adoption and sustained use to improve care outcomes. Previous studies have not examined super users’ integrated use of available VHRs across the health care continuum or within service-specific clinical workflows.

**Objective:**

The objective of this project was to describe VHR super users’ activities and outcomes to document their practices and inform identification and dissemination of current and best practices.

**Methods:**

A prospective, concurrent, mixed methods design using qualitative interviewing and human-centered design was applied. Purposive sampling was used to recruit and conduct interviews with super users across 5 specialty services (ie, cardiology, whole health, spinal cord injury, education, and rehabilitation) at a large Department of Veterans Affairs hospital in the Southeastern United States. Tailored scripts and surveys were used to conduct data collection. Rapid iterative content analysis and data triangulation were used to inform data distillation.

**Results:**

Super user (N=15) data, from specialty services (n=5) revealed 57 best practices (48 data-derived and 9 established) using 60 VHRs within 11 major categories of tasks (eg, appointment management, patient care delivery, medication management), with 43 subcategories (eg, scheduling, appointment reminder, preappointment huddles). Computerized patient record system and joint legacy viewer, Secure Messaging, telephone, Department of Veterans Affairs Video Connect, and Microsoft Teams were reported as 5 most commonly used VHRs. Across the care continuum, best practices around patient-generated health data (n=15), and appointment management and patient and staff technology resource access (n=13) were most commonly reported.

**Conclusions:**

Results identify super users’ practices through case scenarios that illustrate the integration of VHRs within and across service-specific clinical workflows. This project informs “best practices” through integrated use of VHRs across the care continuum. While the findings were specific to 5 specialty services, practices can be applied across services throughout workflows. Recommendations were also made for consideration for deimplementation of select practices. Findings can be used for training, education, and establishing best practices and deimplementation of practices. Future research should aim to evaluate outcomes associated with the use of best practices. These data-driven products lay foundational work in identifying and disseminating best practices in VHR use to inform the transformation of the Veterans Health Administration’s learning health care system into a culture of VHR super users.

## Introduction

As the largest integrated health care system in the United States, serving a population of 9 million veterans across more than 1300 facilities nationwide [1], the Veterans Health Administration (VHA) has been committed to providing high-quality, innovative care throughout its 20-year history of telehealth initiatives [2-4]. Pursuant to the 2017 VHA telehealth services “Anywhere to Anywhere” initiative [5] and Section 151 of the 2018 MISSION Act [6], synchronous, asynchronous, and remote patient monitoring telehealth modalities have become critical mechanisms to deliver practical and efficient health care services across Department of Veterans Affairs’ (VA) urban and rural patient populations [7]. While the COVID-19 pandemic shifted the world into a digital-first culture, VA telehealth has been steadily gaining momentum for 2 decades. Data reported in the VHA 2023 annual report showed that telehealth service usage hit an all-time record high, with more than 2.4 million veterans reporting access to these services, resulting in 11.6 million telehealth encounters [8].

To expand access to care and meet the demand for increased telehealth services, the VA, operating as a learning health system, is supporting quality improvement efforts to identify, implement, and disseminate evidence-based best practices to improve system-wide operations [9]. To build upon this momentum, VA is rapidly expanding efforts to prioritize virtual care, and in May 2022, the VA Health Services Research and Development service and the Office of Connected Care (OCC) held a virtual care state-of-the-art conference to create a research agenda to address access, engagement, and outcomes related to veterans’ use of virtual care technologies. While support for virtual health care resources (VHRs) among providers and patients, both pre- and post–COVID-19, has been established [10-14], further exploration into the determinants for using these tools may provide additional insight into the influential factors for sustained use [15].

Research indicates that health care providers drive patients’ engaged use of VHRs [16-18] and proactive use of VHRs can optimize patients’ access to, and perceived experience with, care [19-22]. Though the positive impact of VHR use is indicated in the literature, providers often use VHRs reactively as singular systems, lacking established protocols and processes for proactive integrated use [23]. Proactive integrated VHR use is described as the self-initiated coordinated use of applicable virtual systems as a team or as an individual for coordinating and delivering timely, high-quality patient-centered care [10]. In our previous work to identify proactive integrated VHR use, we found limited examples of these phenomena beyond the team-based integrated use of singular tools, such as secure email messaging between clinical care teams and their patients. However, in this previous work, we identified individuals who were self-identified, or identified by others, as early adopters and innovators that used multiple VHR systems to coordinate and deliver care. These individuals were coined super users.

Evaluating the use of VHRs through the lens of complex clinical workflows requires interdisciplinary and widespread knowledge of technologies, tools, and resources available to clinical care teams to strengthen workflow processes and care delivery outcomes [10,13,24]. Based on previous work, best practices have not been established to guide the use of VHRs in clinical practice, and effective use of integrative VHR use can support the efficiency of VHR implementation by adhering to recommended best practices [10]. The objective of this project is to describe super users’ VHR activities and outcomes to document their practices and inform identification and dissemination of current and best practices to support broader proactive use of multiple VHRs to coordinate care.

For the purposes of this project, super users are defined as clinical team members who believe that a clinical information system is beneficial to delivering care and important in performing work tasks and are willing to share the functionality that they have learned with others, often resulting in a training role. As such, this definition includes those clinical team members who are well-known users of VHRs (ie, early adopters) to coordinate specialty care delivery and promote adoption with their colleagues (ie, champions). For the purposes of this project, a “best practice” is defined as an appropriate and efficient use of one or more VHRs to complete a clinical task. These practices may not be commonly known or practiced but are more common among super users. A “current practice” is an established, appropriate, and efficient use of one or more VHRs to complete a clinical task, which would be commonly known and practiced in the field.

The aims for this project were to (1) describe inputs, activities, and outcomes of identified VHR super users across 5 specialty services, including cardiology, whole health, spinal cord injury, rehabilitation, and education; (2) collaborate with operational partners (eg, OCC) to develop a VHR best practices navigation guide blueprint to inform the development of navigation support for VHR education and information resources across systems (eg, Talented Management System); and (3) develop data visualization assets to organize and illustrate super users’ best practices across the continuum of care in 5 specialty services. Super user–informed products will provide foundational work in identifying and disseminating best practices in proactive integrated VHR use, with the goal of transforming the veterans’ health care system through a culture of VHR super users.

## Methods

### Study Design and Approach

**Figure 1:**
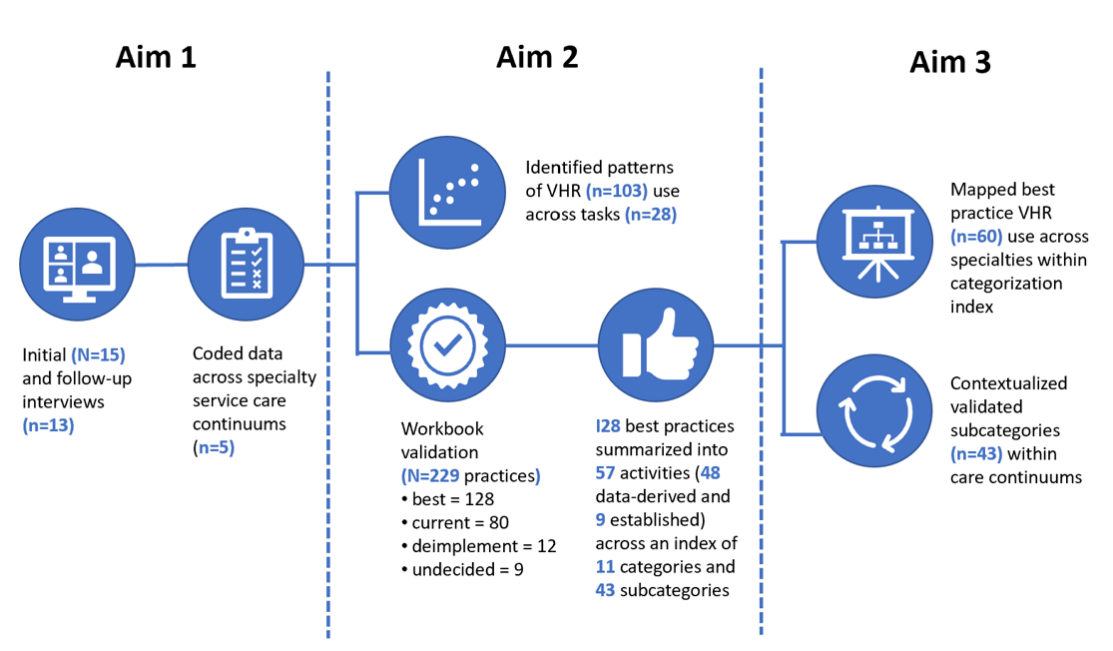
Study flowchart. VHR: virtual health care resource.

This observational quality improvement project used a prospective, concurrent, mixed methods design leveraging qualitative interviewing and a human-centered approach. The primary activities of the 3 aims are illustrated in the study flowchart (Figure 1).

Data collection was contextualized within a super user logic model, using a health care continuum from a previous project [10]. Content analysis was guided by relevant constructs to identify unique and shared best practices in the integrated use of VHRs through inputs, activities, and outcomes.

An adapted purposive (ie, criterion-i, extreme) snowball sampling was used to recruit and conduct interviews with super users across 5 specialty services (ie, cardiology, whole health, spinal cord injury, education, and rehabilitation) [25]. Tailored scripts and surveys were used to conduct data collection. Human-centered design participatory methods, product development protocols, and rapid iterative content analyses were used, in collaboration with OCC partners and clinical specialty care super users. Think-aloud strategies [26] were used in combination with interviewing techniques. Rapid iterative content analysis [27] and data triangulation were used to inform data distillation and visualization products. Validation strategies were used with OCC partners and subject matter experts to confirm use cases, illustrate patterns of behaviors, and document educational resources for the proactive integrated use of VHRs across common tasks within a clinical workflow.

### Setting

This project was conducted across 5 specialty services (ie, cardiology, whole health, spinal cord injury, rehabilitation, and education) at a large VA hospital in the Southeastern United States. This VA hospital is one of the OCC’s Virtual Health Resource Centers, a health organization committed to fostering proactive integrated use of VHRs [28].

### Participants and Sample

Initial interviews were held using Microsoft Teams with VHR super users (N=15) across 5 specialty services. Representative snowball sampling was used, through the on-site virtual health resource center, to recruit clinical champion participants based on their knowledge and proactive integrated use of VHRs, as identified by their clinical team members within each specialty care service. Follow-up interviews (n=13) were conducted using Microsoft Teams for validation and logic model (eg, care continuum) demonstrations.

### Positionality Statement

The quality improvement project team brought together a diverse range of expertise. JNH holds a Doctor of Philosophy degree and has 2 decades of experience in implementation and evaluation across a range of health science topics, including virtual health. RB possesses a Bachelor of Arts and specializes in writing and data visualization. RB managed the data throughout this quality improvement project. JMG is a licensed occupational therapist with clinical proficiency, while LMB is a doctor of physical therapy with clinical expertise. SAK is a registered nurse who served in the military and has experience as a veteran navigating the VA system. Both RSB and JTM hold Doctor of Philosophy degrees with backgrounds in health science. Collectively, the team offers substantial experience and knowledge in clinical workflows, virtual health, and VHA policies, processes, and tools.

### Data Collection

Initial and follow-up interviews were held virtually, which were recorded and transcribed. The initial interview guide (Multimedia Appendix 1) was piloted with team members experienced in VHRs to provide feedback and refinement. Each session included a facilitator and a notetaker (JMG, LMB, and SAK). During each 60-minute initial interview (May-July 2021), super user participants (N=15) were guided through an activity using the care continuum model (ie, pre-encounter, postencounter, and ongoing health management) from a previous project [10]. Throughout this activity, interviewees defined proactive integrated use of VHRs across a clinical workflow and described activities or tasks they complete during their workflow and the VHRs used to complete each task. Interview questions further identified facilitators and barriers of VHR use, examples of patient-generated health data (PGHD) within workflows, suggestions for sharing best practices and expanding VHR uptake across their service, and mechanisms for tracking performance measures.

Prior to follow-up interviews, participants (n=13) were tasked with reviewing precompleted, service-specific care continuums from the initial interviews using the robust list of VHRs identified by all services. The 60-minute follow-up interviews (October-December 2021) prompted responses to the usefulness of VHRs, ease of workflow, facilitators for performance measures, workflow barriers, tools for inpatient settings, tools and practices unique to the service, facilitators for promoting uptake and adoption, and the establishment of best practices.

### Data Analysis

Using human-centered design, rapid, iterative content analysis was used to analyze data. Content analysis was used to topographically categorize VHR use, barriers, facilitators, and recommendations. Aims 1-3 used a multistepped process to collect data, code data, and identify patterns of use and best practices. Within this multistepped process, validation phases were conducted by the team with operational partners and subject matter experts to validate findings. Figure 1 depicts the data analysis workflow for this project.

### Health Care Continuum

**Figure 2:**
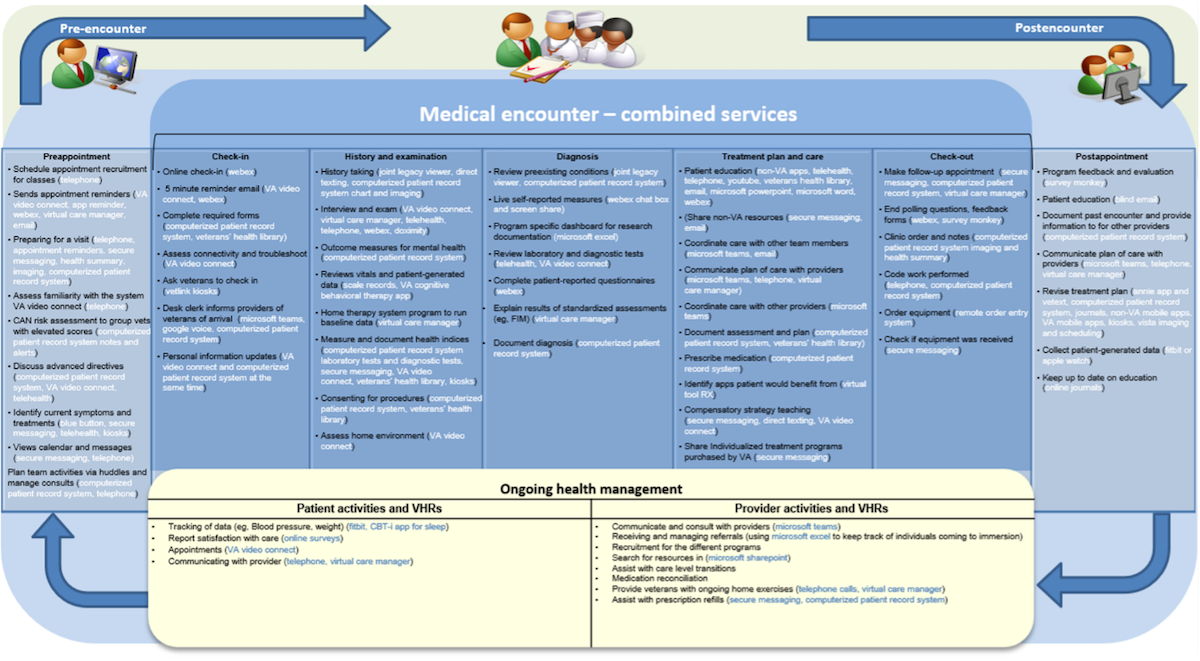
Preliminary super user data collected into a combined services care continuum. CAN: Care Assessment Need; FIM: Functional Independence Measure; RX: prescription; VA: Department of Veteran Affairs; VHR: virtual health care resource. A higher-resolution version of this image is available as Multimedia Appendix 2.

Using a preliminary health care continuum draft from a previous study [10], Aim 1 initial and follow-up interview data were collated by qualitative researchers into unique service care continuums with the inclusion of tasks and VHRs. Using an iterative process, tasks and platforms were documented along the health care continuum. Drafts of the continuum with collated information were provided to participants iteratively to validate and finalize (Figure 2). Ultimately, all the continuums were combined because it was determined that, although there were service-specific uses of VHRs reported, all uses were applicable across all services.

### Specialty Services Best Practices Identification Workbook

To establish current and best practices, as well as practices for deimplementation, the team developed an interactive specialty services best practices identification workbook (Multimedia Appendix 3). The workbook included use cases across each phase of the care continuum to assess and categorize reported practices and establish consensus among tasks and VHRs between the research team and operational partners. VHR interview data were thematically organized and categorized by purpose (eg, patient-generated tools, VA apps, provider vs patient-initiated communication).

The workbook of current reported practices (eg, task) was organized alphabetically by service line. Clinical tasks with documented VHRs were listed underneath each activity category across sequential care continuum phases. The interactive workbook provided detailed instructions for use: (1) read each task, (2) choose the appropriate practice type from a drop-down menu (ie, current, best, deimplement, undecided, or no response), and (3) enter any relevant notes in the designed field, as appropriate.

Operational partners (CA and TH) completed the first round of the workbook after a live demonstration with the team and sent the completed workbook via email upon completion. Team members were available during this process to answer any questions or troubleshoot. For any practices that were out of scope, undecided, or required additional subject matter expertise, the team sent the identified pages of the workbook to clinical experts for further consideration. Each task and VHR were mapped to clinical workflow care continuum phases with clinical and operational input. Operational partners analyzed the data within the specialty services workbook, selecting the most appropriate practice for each activity. After partners completed their review, the team conducted several working meetings with partners to finalize validation of each practice through consensus, established across multiple decision-making rounds.

In tandem, the team used the workbook to evaluate current practices in the field via a crosswalk of instructional materials and resources on the Connected Care Academy website and other VA resources, and documented the location of educational links for each VHR and instructional materials for how to perform each practice in the field.

### Data Trustworthiness and Rigor of Analysis

Several precautions were taken to ensure trustworthiness and rigor of data collection and analysis. The qualitative data team routinely discussed the definitions and relevance of codes, refining data categorizations as needed to guarantee consistency and quality of coding. Personal bias was minimized by using cross-checking practices among multiple analyses. Discrepancies were reconciled during several team discussions.

### Ethical Considerations

This protocol was reviewed by the James A Haley Veterans’ Hospital Research and Development Committee (IRBNet #1605571) and the OCC. Based on documentation, this protocol was deemed nonresearch to support quality improvement efforts. No informed consent was required. All methods were carried out in accordance with relevant guidelines and regulations. No compensation was provided to the participants in this quality improvement project. Participants’ identities were confidential and deidentified. Data were stored behind VA firewalls and presented anonymously in an aggregate form in accordance with the VHA policies and regulations.

## Results

Interview participants (N=15) were predominantly White (9/15, 60%) and female (10/15, 66.7%), who spent 4-7 hours per day using VHR (6/15, 40%). Respondents held various clinical roles, including nurse (4/15, 33.3%), medical providers (3/15, 20%; medical doctor, doctor of osteopathy, and advanced registered nurse practitioner), therapist (2/15, 13.3%; occupational, physical, and speech), support staff (2/15, 13.3%), and a psychologist (1/15, 6.7%). Among participants, the average number of years in practice was 18.4, and the average tenure at VA was 16.7 years (Table 1).

Participants reported a total of 103 VHRs used in day-to-day clinic operations (Textbox 1). Multimedia Appendix 4 further illustrates the reported use of these VHRs, as listed on the x-axis, to conduct 28 tasks across the y-axis. Of note, super users reported the use of up to 28 unique VHRs to complete a single task.

**Figure 3:**
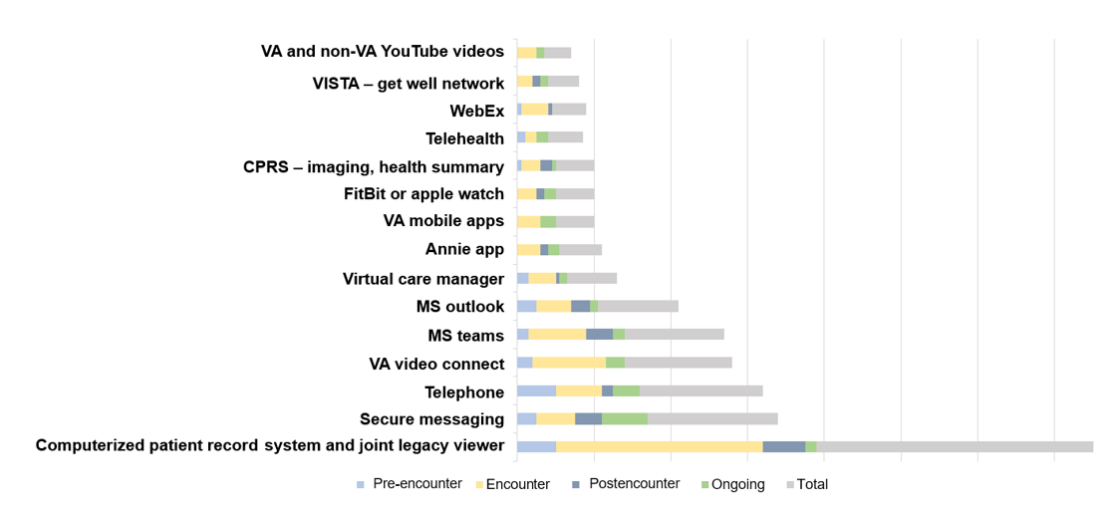
Top used virtual health care resources reported across each phase of the care continuum. CPRS: computerized patient record system; MS: Microsoft; VA: Department of Veterans Affairs; VISTA: Veterans Health Information System Technology Architecture.

Across the health care continuum, the most widely used VHR was the dual computerized patient record systems (CPRS) and joint legacy viewer (JLV) electronic health record system. The dual CPRS and JLV system was most frequently used during patient encounters and was used equally as much as telephones during the pre-encounter phase. Secure Messaging proved to be the most frequented on an ongoing basis (Figure 3).

**Table 1:** Demographics of super user participants (N=15).

Characteristics		Value	
Age (years), mean (SD)		47.5 (10.14)	
Years in practice, mean (SD)		18.4 (9.09)	
Years in VA^a^, mean (SD)		16.7 (10.55)	
**Role, n (%)**			
	Provider (MD^b^ or DO^c^)	3 (20)	
	Nurse (ARPN^d^, RN^e^, or LPN^f^)	5 (33.3)	
	Psychologist	1 (6.7)	
	Therapist (occupational, physical, or speech)	2 (13.3)	
	Other (telehealth coordinator or health education coordinator)	2 (13.3)	
	Decline to respond	2 (13.3)	
**Race, n (%)**			
	Black or African American	1 (6.7)	
	Asian (Chinese, Filipino, Japanese, Korean, etc)	1 (6.7)	
	White or Caucasian	9 (60)	
	Other (Indian)	1 (6.7)	
	Decline to respond	3 (20)	
**Ethnicity, n (%)**			
	Hispanic	1 (6.7)	
	Not Hispanic	11 (73.3)	
	Decline to respond	3 (20)	
**Sex, n (%)**			
	Female	10 (66.7)	
	Male	3 (20)	
	Decline to respond	2 (13.3)	
**Highest professional degree, n (%)**			
	Graduate degree	4 (26.7)	
	Master’s degree	7 (46.7)	
	Bachelor’s degree	1 (6.7)	
	Some college	1 (6.7)	
	Decline to respond	2 (13.3)	
**Frequency of VHR^g^ used in a workday, n (%)**			
	1-3 hours a day	3 (20)	
	4-7 hours a day	6 (40)	
	≥8 hours a day	4 (26.7)	
	Decline to respond	2 (13.3)	

^a^VA: Department of Veterans Affairs.

^b^MD: medical doctor.

^c^DO: doctor of osteopathy.

^d^ARPN: advanced registered nurse practitioner.

^e^RN: registered nurse.

^f^LPN: licensed practical nurse.

^g^VHR: virtual health care resource.

**Virtual health care resources (VHRs; n=103) identified by super users.**

**Single VHRs**

- 3D Camera
- Accessibility Management Platform (not included in final analysis due to lack of saturation for the threshold of targeted use; hereafter referred to as “not included”)
- Blood pressure monitor (peripheral device)
- Commission on Accreditation of Rehabilitation Facilities Survey
- Cisco Jabber
- Clinical video telehealth (not included)
- Computerized patient record system (CPRS)
- Digital scale (peripheral device)
- Direct texting
- Document Storage Systems DocManager (not included)
- Doximity
- EarlySense Monitoring System (not included)
- Echo Device (peripheral device; not included)
- Electronic Health Record Modernization Cerner Millennium (not included)
- Engineered Care (not included)
- Fitbit or Apple Watch
- Get Well Network (Veterans Health Information System Technology Architecture [VistA])
- Glucometer
- Google Voice (not included)
- iPad
- Light Electronic Action Framework (not included)
- Medline Plus (not included)
- Mobile devices (not included)
- My HealtheVet (MHV)
- Online Journal Websites (not included)
- PDF (not included)
- Personal Health Inventory Questionnaire
- Pulse oximeter (peripheral device)
- Qualtrics
- RedCap
- Stethoscope (peripheral device)
- Survey Monkey
- Telehealth (not included)
- Telephone
- Talent Management System (not included)
- URL generator
- USA Mobility (not included)
- Department of Veteran Affairs (VA) and non-VA apps
- VA and non-VA online resources (not included)
- VA and non-VA YouTube videos
- VA-approved acute enterprise standard (VistA; not included)
- VA intranet (not included)
- VA National Surveillance Tool (NST; not included)
- VA Video Connect (VVC) or VVC Now
- VA Virtual Toolkit Prescription Pad
- VEText (not included)
- VetLink Kiosk
- Virtual Care Manager
- Vocera (not included)
- Web VistA Remote Access Management (not included)
- WebEx
- Weight Management Scale (peripheral device)
- Your IT (not included)
- Zio Patch
- Zoom

**Apps**

- Alivecor
- Annie app for clinicians
- Annie app for veterans
- CBT-i Coach
- CPT Coach
- Image viewing solution (VistA app)
- Insomnia Coach
- Live Whole Health
- My VA Images (not included)
- PTSD Coach
- Share My Health Data
- VA Health Chat (not included)
- Virtual Tool RX

**CPRS**

- Barcode medication administration (not included)
- Care Assessment Need Risk Assessment
- Consults
- Flags
- Health Summary (not included)
- iMedConsent (not included)
- Inpatient blood pressure machine
- Inpatient vital machine
- Joint Longitudinal Viewer (not included)
- KRAMES or KRAMES On-Demand
- Laboratory tests
- Mental health assistant (not included)
- Notes and alerts
- Patient Care Assessment System (not included)
- Remote Order Entry System
- Return to Clinic Order (not included)
- Veterans’ Health Library (not included)
- VistA Imaging (not included)
- Veterans Integrated Service Network 8 Nucleus

**MHV**

- Blue Button (not included)
- Healtheliving Assessment (not included)
- RX Refill app
- Secure Messaging
- Track Health-Journals (not included)
- Track Health-Vitals (not included)
- VA Appointments Tool (not included)
- VA NST (not included)
- VA RX Refill
- Wellness Reminders (not included)

**Microsoft Corporation**

- Excel
- Outlook
- PowerPoint
- SharePoint
- Teams
- Word

From these novel VHR integrations, we identified 128 best practices across 5 VA specialty care services. To reduce redundancy, we grouped similar practices into summaries (n=57; 48 data-derived and 9 established) that incorporated all VHRs and tasks (Table 2).

**Table 2:** Virtual health care resource (VHR) best practices categorization index table. Summaries corresponding to each summary number are provided in Multimedia Appendix 5.

Index category and Index subcategory task		Relevant summary number
**1. Appointment management**		
	1.1 Scheduling medical appointment	12
	1.2 Sending appointment reminder to veteran	20, 23, 24, 25
	1.3 Sharing virtual appointment access	5^a^
	1.4 Preparing for virtual appointment	22, 26, 28
	1.5 Preappointment huddles	22, 13^a^
	1.6 Follow-up appointment	2, 26
**2. Patient and staff technology and resource access**		
	2.1 Dedicated resources for device consult, or troubleshooting, or responding to alert	4, 26
	2.2 Prepare for visit and assess technology	16, 26, 27
	2.3 Provide troubleshooting	1, 26, 29
	2.4 Provide training and support	16, 28
	2.5 Obtaining digital equipment	21, 26, 36
**3. Communication and referrals**		
	3.1 Provider, veteran, and nonveteran communication	2, 14, 15, 18, 30, 34
	3.2 Internal and external interdisciplinary coordination and communication	3, 4, 10, 22, 25, 29, 33
	3.3 Veteran-initiated communication	18
	3.4 Provider-initiated communication	14
	3.5 Adaptive communication with veteran	1, 6, 8, 15
	3.6 Preparation for communication with veterans and caregivers	17
	3.7 Facilitating virtual communication and access for inpatients	31, 48
**4. Patient care delivery**		
	4.1 Check-in and triage	32, 33
	4.2 Take history	7, 12
	4.3 Conducting virtual assessment	5, 6, 7, 12, 34, 36, 38, 39, 42
	4.4 Providing treatment and recommendations	7, 19, 34
	4.5 Conducting group appointment	35^a^
	4.6 Follow-up	14
**5. Laboratory and diagnostic tests management**		
	5.1 Request laboratory tests or imaging	56^a^
	5.2 Deliver laboratory tests or imaging results	57^a^
**6. Referral management**		
	6.1 Consult for device issuance	36
	6.2 Provider referrals for care	53^a^
	6.3 Referrals for technology	54^a^
**7. Data collection management**		
	7.1 Program feedback and evaluation	37, 55^a^
	7.2 Documenting data summaries	37
	7.3 Outcome measures	8, 18, 40, 41, 42, 50, 51
**8. PGHD management**		
	8.1 Consult for PGHD^b^ device issuance	36, 52^a^
	8.2 Track PGHD, record vitals, and monitor health indices	9, 11, 19, 30, 37, 39, 42, 49, 50, 51
	8.3 Measure PGHD, vitals, and health indices	11, 42, 49, 50
	8.4 Veteran-based VHR	11, 19, 30, 38, 42, 43, 49
**9. Medication management**		
	9.1 Requesting prescription or treatment by veteran	43
	9.2 Refill and track medication	43
**10. Resources and education**		
	10.1 Obtaining and organizing educational material	44, 45
	10.2 Delivering educational material to patients	7, 8, 14, 20, 28, 46, 48
	10.3 Continuing education and access to resources for providers	47
**11. Documentation**		
	11.1 Chart review and check records, or laboratory tests or imaging	10
	11.2 Provider notes	10

^a^Indicates a summary that is an established best practice.

^b^PGHD: patient-generated health data.

**Figure 4:**
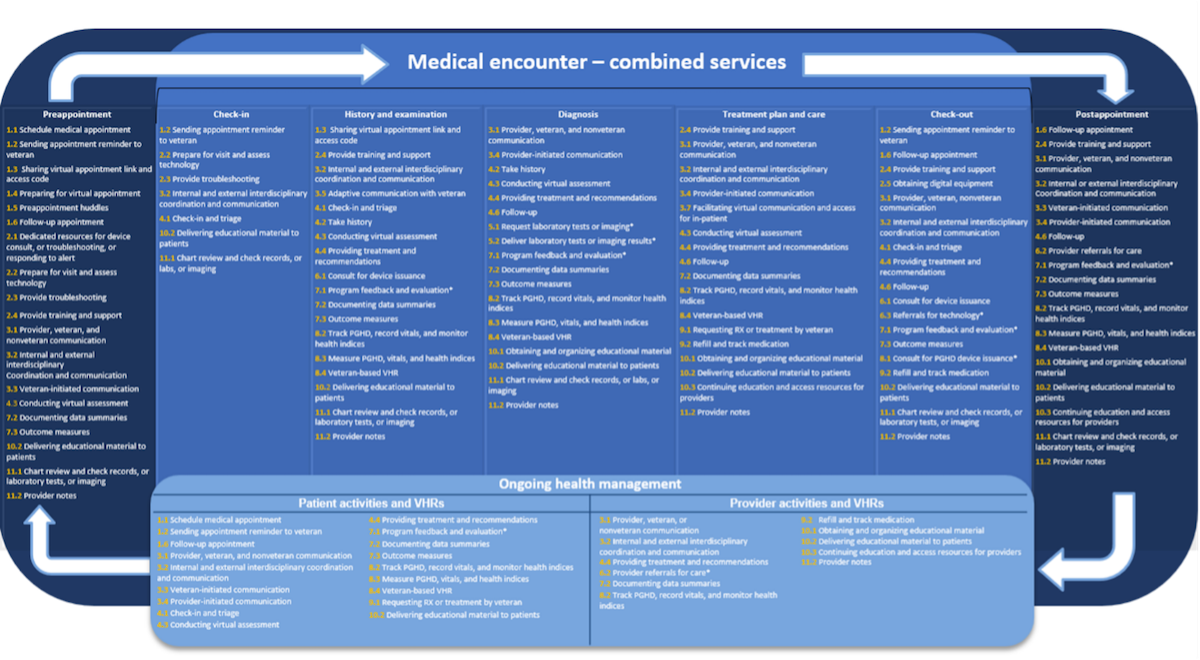
Taxonomic integration of categories and subcategory tasks into the combined services care continuum. Asterisk indicates established best practice. PGHD: patient-generated health data; RX: prescription; VHR: virtual health care resource. A higher-resolution version of this image is available as Multimedia Appendix 6.

Finally, we created taxonomy categories (n=11) and subcategory tasks (n=43) to which we mapped the best practice summaries, thereby producing a practical, scalable guide to effective VHR integration across the care continuum (Table 2 and Figure 4).

These data visualization assets demonstrate how multiple VHRs can be successfully integrated within a specific clinical task. For example, as illustrated in Textbox 2, depicting application in a single category across subcategory tasks, summary 50 describes how a provider can review tracked data from a VA-specific mobile app (eg, CBT-i Coach) synced to a veteran wearable device (eg, Fitbit or Apple Watch) and recommend adjustments.

**Example summary of virtual health care resource use across several subcategory tasks to depict patient-generated health data (PGHD) management.**

**Example summary used across 3 PGHD management subcategory tasks (8.2 Track PGHD, record vitals, and monitor health indices; 8.3 Measure PGHD, vitals, and health indices; and 8.4 Veteran-based virtual health care resource).**

- Summary 50: You can use the CBT-i Coach app and the data from Apple Watch or Fitbit to review and track weekly sleep cycles and can adjust the schedule when needed. You can use information from the service connection disability rating and vitals generated in both computerized patient record system and CBT-i Coach app to ensure veterans are improving over the course of time together with the provider.

Additionally, a cross-examination of these data identified summaries 11 and 26 as the most widely used practices across the care continuum. For example, summary 11 was prevalently used in the index category 8 (PGHD management), which included 15 total instances of use across 8.2, 8.3, and 8.4 subcategory tasks. Similarly, summary 26 was primarily used across 2 index categories: appointment management (category 1) and patient and staff technology and resource access (category 2). This included 13 total instances across 1.4, 1.6, 2.1, 2.2, 2.3, and 2.5 subcategory tasks (Table 3).

**Table 3:** The use of summaries 11 and 26 across index categories, care continuum phases, and relevant subcategory tasks.

Summary number, index category, and care continuum phase			Index subcategory task
**11**			
	**8. PGHD^a^ Management**		
		History and examination	8.2 Track PGHD, record vitals, and monitor health indices 8.3 Measure PGHD, vitals, and health indices 8.4 Veteran-based VHR
		Diagnosis	8.2 Track PGHD, record vitals, and monitor health indices 8.3 Measure PGHD, vitals, and health indices 8.4 Veteran-based VHR
		Treatment plan and care	8.2 Track PGHD, record vitals, and monitor health indices 8.4 Veteran-based VHR
		Postappointment	8.2 Track PGHD, record vitals, and monitor health indices 8.3 Measure PGHD, vitals, and health indices 8.4 Veteran-based VHR
		Ongoing health management patient activities	8.2 Track PGHD, record vitals, and monitor health indices 8.3 Measure PGHD, vitals, and health indices 8.4 Veteran-based VHR
		Ongoing health management provider activities	8.2 Track PGHD, record vitals, and monitor health indices
**26**			
	**1. Appointment management**		
		Appointment management	1.4 Preparing for virtual appointment 1.6 Follow-up appointment
		Preappointment	1.4 Preparing for virtual appointment 1.6 Follow-up appointment
		Check-out	1.6 Follow-up appointment
		Postappointment	1.6 Follow-up appointment
		Ongoing health management patient activities	1.6 Follow-up appointment
	**2. Patient and staff technology and resource access**		
		Preappointment	2.1 Dedicated resources for device consult or troubleshooting or responding to alert 2.2 Prepare for visit and assess technology 2.3 Provide troubleshooting
		Check-in	2.2 Prepare for visit and assess technology 2.3 Provide troubleshooting
		Check-out	2.5 Obtaining digital equipment

To further understand the integration of best practice summaries within the care continuum model, Multimedia Appendix 7 provides an example for the PGHD management category and subsequent listed subcategory tasks: (1) consult for PGHD device issuance; (2) tracking PGHD, recording vitals, and monitoring health indices; (3) measuring PGHD, vitals, and health indices; and (4) veteran-based VHR. Across these subcategory tasks, 15 unique best practices can be applied to improve clinical workflows and patient-centered outcomes.

Furthermore, in the first subcategory task, consult for PGHD device issuance, clinicians may use CPRS and the remote order entry system during the history and examination phase of treatment. Further, a significant amount of PGHD can be collected using VA apps, which can be prescribed using the VA Virtual Toolkit Prescription Pad. Consults for device issuance of items addressing the digital divide, such as iPads, are entered through the consults tool (CPRS). Additional PGHD equipment, such as a wearable tracking device (ie, Fitbit or Apple Watch), can be ordered through the VA Light Electronic Action Framework system. Additional examples across other phases of the care continuum are available in Multimedia Appendix 8. The full categorization index that corresponds to the combined care continuum is available in Multimedia Appendix 9.

Beyond providing important data on VHR integration, clinical partners validated findings and further identified practices that lacked efficiency and created additional burdens on clinicians, staff, and patients. These included sending surveys to veterans using Survey Monkey via Microsoft Outlook, using WebEx and Survey Monkey for feedback collection, using Microsoft Outlook and WebEx chat box to send presentations, using WebEx and Survey Monkey for polling, sending survey links via Microsoft Outlook, and using Zoom for provider-veteran communication. As such, these practices and VHRs should be considered for deimplementation. The VHR use best practice matrix illustrates the journey of each service line’s use of VHRs across the clinical workflow categorization index (Multimedia Appendix 10).

## Discussion

### Principal Findings

Super user data gleaned 57 best practices (48 data-derived and 9 established) using 60 VHRs within 11 major categories of 43 subcategory tasks. These data represent the first in-depth review of super user behaviors to proactively coordinate specialty care using multiple VHRs within a large, complex health care system. Although the findings were specific to 5 specialty services, practices can be applied across services throughout the care continuum workflow. Importantly, findings illustrate how super users integrate multiple VHRs to improve efficiency, optimize workflow, and advance patient-centered outcomes.

VHR super users engage in innovative practices from which other health care providers in the broader specialty care clinician population can learn. Though the pace of innovation adoption is speeding up, research indicates it takes several years to fully realize the normalization of technology use [29], which is applicable to proactive integrated use of VHRs in health care settings [30]. By defining and disseminating best practices that can be replicated to increase spread and uptake over time [31], the field can increase the rate at which proactive integrated VHR use is adopted and spread.

These data can inform “train-the-trainer” program content to normalize the use of VHR within and beyond specialty care. With ongoing efforts to increase awareness and access to VHR, these products also provide a practical wireframe for searching and accessing VHR educational database resources. These data and products provide a blueprint for search term algorithms to inform the development of VA educational platforms. Given the unique depth and breadth of this work, these data serve as a baseline measure of enterprise-level super user–level use of VHRs.

For the past 8 years, a collaboration between veterans, the Consumers Health Informatics Office, and OCC resulted in the evaluation, implementation, and identification and dissemination of best practices [17,31-34]. The identification of 57 best practices in proactive integrated VHR use across 5 VA specialty services is a key strength of this paper and provides an important addition to the literature on the systematic application of digital tools within clinical workflows. The findings are consistent with past research, suggesting that engagement among clinicians in the early adoption and integration of VHRs contributes to improved care efficiency, workflow optimization, and patient-centered outcomes [35]. Diverging from previous reports, which have mainly emphasized the barriers to the adoption of VHRs [36], this study provides innovative examples of how super users actively facilitate integration by aligning VHR use along various phases of the care continuum. The documentation of tools and their applications across clinical tasks fills an important gap in the literature via the offering of replicable models for broader implementation, deimplementation, and dissemination.

### Comparison to Prior Work

We show that proactive VHR integration is most effective when undertaken via interdisciplinary collaboration. Findings indicate that super users make frequent use of communication and coordination platforms, including Microsoft Teams and Virtual Care Manager, to facilitate efficient communication across care teams and between services. These findings support work discussed in previous studies on the benefits of interdisciplinary collaboration to enhance care coordination and promote successful telehealth integration [37]. However, a key distinction of this study from past work pertains to the identification of specific mechanisms and digital tools used to support team-based care planning, including preappointment huddles and shared access to patient clinical data. As such, we share new information about the operationalization of integrated virtual care workflows, which is a domain that remains underdeveloped in current implementation science literature [38].

Furthermore, we demonstrate that tools such as the Annie app for veterans, CBT-i Coach, and wearable devices are used not only to collect and monitor PGHD but also to synchronize data within existing clinical systems, like CPRS. The finding regarding PGHD is consistent with past research, which has examined the potential of PGHD use for chronic disease management and health monitoring [39]. However, we advance the literature in this area by demonstrating how PGHD can be operationalized within clinical workflows through provider-initiated strategies and supportive digital infrastructure. These outcomes are responsive to prior calls for system-level protocols that ensure meaningful and sustained use of PGHD in routine care [40].

### Future Directions

This study addresses long-standing concerns regarding the digital divide by illustrating practical and scalable strategies used to support equitable access to virtual care. For example, findings suggest using consults to address the digital divide and the issuance of devices via remote order entry system are implemented in VA to mitigate historically identified barriers to use. Activities such as these align with recent national efforts to expand use of digital health resources among military veterans [41]. Unlike past studies that focus on implications exclusively for health care policy, this study offers granular, practice-based evidence to inform training interventions, workflow redesign, and national standards for VHR use among veterans. Thus, this work demands a broader transformation of the VA health care system toward a culture that normalizes and sustains integrated virtual care practices.

### Strengths and Limitations

These data represent some of the first in-depth, actionable practices used by identified super users to coordinate care using multiple VHRs. The power of these results is in their innovative use of multiple VHRs to coordinate care across the care continuum in a way that can be objectively operationalized for broad dissemination and adoption. These data not only provide insights into the potential of super use by individuals but can inform the integrated adoption by teams to support cultural transformation within specialty clinical care environments to optimize the delivery of virtual care. The implications of this transformation cannot be overstated, given the realization of the need for remote access to care for diverse audiences (eg, rurally located patients), as well as during global pandemics (eg, COVID-19).

Interpretation of findings should be considered within the context of the following limitations. First, this project provides a scan of perceived “best practices” self-reported by super users. Content may not be exhaustive due to respondents’ knowledge base. Practices may not have been reported, or respondents may be unaware that a practice exists, or their “workaround” may not be an ideal practice (eg, consideration for deimplementation). With the constant change in the VHR climate, especially post pandemic and within a large government health care system, policies and practices change regularly, which may impact the accuracy and currency of these data as virtual systems continue to evolve. Second, the study team acknowledges the limited scope of these findings, only representing 5 specialty services at 1 VA site. However, it is notable that this site was ideally selected for its status as a virtual health resource center. As such, these data represent an in-depth case review of individual practices within a clinical resource hub. We assert that best practices are applicable and transferable to other service lines across the VA enterprise. Third, during analysis, we had to balance brevity and precision with a comprehensive illustration of proactive integrated use of VHRs. In general, only practices that emerged from the data are illustrated; however, knowledge gleaned from the field is denoted throughout. Fourth, the subjective nature of methods and analysis, including the determination of a “best practice,” and the rapid change in VHR policies and practices must be contextualized within the team’s definitions, operationalization strategies, and perceptions when interpreting results over time. To address this limitation, the study team worked with multiple super users, subject matter experts, and operational partners to ensure diverse representation in the sample to elicit valid data on proactive integrated use of VHRs. Further, multiple analysts reviewed data to determine “best practices.” Identified practices were further validated by operational partners. Finally, the observational nature of this foundational work is descriptive in nature and limited in its capacity to inform the identification and measurement of outcomes and impact of super user practices. However, with this groundwork laid, future work can focus on measures of adoption and use of integrated VHR systems by individuals and clinical teams and operationalize outcomes at the individual, team, patient, and system levels over time.

### Conclusions

As individuals and clinical care teams embrace the use of VHRs to coordinate remote care in a postpandemic climate, data from this study identify practices through case scenarios that illustrate the integration of VHRs within and across service-specific clinical workflows. This project informs “best practices” in the proactive integrated use of VHRs across the continuum of care. Super users’ best practices using VHRs can inform training, skill development, and revised workflows to maximize clinical efficiency and patient benefit while operationalizing VHR practices to test implementation and deimplementation strategies to promote uptake and spread. Future work should seek to build knowledge, skills, and capacity for the proactive integrative use of VHRs across the continuum of care and adapt and enhance workflows to promote the cultural transformation of VHR super users across the VA system of care and beyond.

## Appendix

Multimedia Appendix 1 Interview guide and care continuum activity.

Multimedia Appendix 2 High resolution Figure 2.

Multimedia Appendix 3 Specialty services best practices identification workbook.

Multimedia Appendix 4 Super users’ reported use of 103 virtual health care resources across 28 tasks.

Multimedia Appendix 5 Best practice summaries listed in consecutive order.

Multimedia Appendix 6 High resolution Figure 4.

Multimedia Appendix 7 Example: Patient-Generated Health Data (PGHD) management summaries on use of virtual health care resources (VHRs) to complete 4 identified common tasks.

Multimedia Appendix 8 Additional examples of the taxonomic structure of best practices across other phases of the care continuum.

Multimedia Appendix 9 Categorization index of best practice virtual healthcare resources (VHRs) use.

Multimedia Appendix 10 Super user journey and virtual health care resources (VHRs) use.

## Abbreviations

CPRS: computerized patient record system
JLV: joint legacy viewer
OCC: Office of Connected Care
PGHD: patient-generated health data
VA: Department of Veterans Affairs
VHA: Veterans Health Administration
VHR: virtual health care resource

## Data Availability

Data supporting this manuscript are available from the corresponding author upon reasonable request.
