# Supplementary material for: Super Users’ Reported Best Practices for Coordinating Proactive Integrated Use of Virtual Health Care Resources: Prospective Concurrent Mixed Methods Human-Centered Design Study
Source: J Med Internet Res. 2025 Nov 14;27:e81414. doi: 10.2196/81414 (PMC12663705; doi:10.2196/81414)
Supplement: Multimedia Appendix 1 [file jmir_v27i1e81414_app1.pdf]

Super Users Interview Guide

“We would like to record this discussion so we can capture everything that is shared. It will include video and audio. If you prefer not to be on video, you can turn off your camera. Do I have your consent to record our discussion?”

“Thank you for agreeing to participate in this interview. My name is [project staff name]. You have been identified as a “Super user”, that is a clinical team member who believes that VA virtual resources are beneficial to delivering care and important in improving workflow. Examples of virtual resources are listed to the right side of the System Use Across the Health Care Continuum figure we shared with you in our earlier email. Virtual resources include things like CPRS, VISTA, Teams, mobile apps…..really any technology you can think of that are available to use as tools to improve patient care, communication and workflow. Today, I will be talking with you about your best practices in using VA virtual resources for delivering care and improving your workflow. This interview is unique in that you will drive the discussion in how you use virtual resources to deliver care. This interview has two parts, the first part is an activity on how you use virtual resources for care delivery across the health care continuum using a model called “System Use Across the Health Care Continuum”. The second part is a discussion on how you use virtual resources to deliver care. We will use this information to spread virtual resource best practices across the VA to improve workflow and Veteran care.

“I would like you to answer openly and to the best of your ability. There is no right or wrong answer. We just want to know about your experiences and what you think about the topic. You can choose not to answer questions or stop participating at any time.”

“The information you share will be confidential and will not be shared beyond the scope of this project. Remember that any reports from this project that include your responses will not be linked with your name.”

“Do you have any questions before we begin?”

First, I would like to get to know more about you:

1) Please describe your current position.

Now, we will share a slide with you for the activity portion of the interview.

<u>Activity:</u>

On the “System Use Across the Health Care Continuum” slide, we have a model of patient health care from pre-encounter to ongoing health management. Under each phase of health care, we have listed some tasks and processes providers have reported using. To the right of this model is a list of some of available virtual resources. Feel free to look to this as a reference during this activity and add any resources that are not currently listed. What we really want to know with this activity is how you use virtual resources in a proactive integrated way. We define proactive integrated use as a self-initiated

approach to coordinated use of applicable virtual resources systems for the purposes of coordinating and delivering timely high-quality patient-centered care. Proactive integrative use really defines your best practices, the things you find improve patient care and workflow, for each phase of the patient health care continuum. Your best practices may not be limited to one phase but instead may cover multiple phases and that is ok.

2) Please explain how you use virtual resources to deliver care and describe tasks you perform with virtual resources during the pre-encounter.
   a) Please give me examples of the VR you use and how you use it in a proactive integrative way during the pre-encounter.

3) Please explain how you use virtual resources to deliver care and describe tasks you perform with virtual resources during the medical encounter.
   a) Please give me examples of the VR you use and how you use it in a proactive integrative way during the medical encounter.

4) Please explain how you use virtual resources to deliver care and describe tasks you perform with virtual resources during the post-encounter.
   a) Please give me examples of the VR you use and how you use it in a proactive integrative way during the post-encounter.
5) In the last section, which tasks do you perform using virtual resources for ongoing health management?
   a) Which virtual resources do you use in a proactive integrative way for these tasks?

Now, we will move to the second part of the interview which is the discussion. We will keep up the slide for you to use as a reference.

<u>Questions:</u>

6) Think about the tasks you mentioned in the activity using VR and tell us how the resources have changed your workflow.

Probes:

   a) How has the integration of VR affected your healthcare delivery?
   b) Tell us when these VR are ideal for delivering care. Provide an example.
   c) In your opinion, when are these VR inappropriate? Provide an example.
   d) What VR are you not currently using? Why?

7) What kind of barriers have you faced in the past using VR?

Probes:

   a) Who do you go to if you experience a barrier using virtual resources?

b) How did you overcome the barriers?

8) Who typically comes to you for assistance with using virtual resources?
Probes:
a) What types of problems do you help resolve? How often?

9) As virtual resources and technology advances, we have more opportunity to receive information and data from our patients throughout the health care continuum. What type of patient generated health data do you currently integrate into your practice? (*i.e. data that are captured by patients, family or caregivers, such as health history, screening questionnaires, symptoms, blood glucose, BP, diet tracking, medication adherence, etc.*).
a) How are you currently using patient generated data in your practice?
b) What types of virtual resources do you and your patients use to share this data?
c) How do you enter this data into the patient health care record?
d) What type of patient generated health data do you wish you could integrate into your practice?
e) How do you envision patient generated data being used to enhance your practice and workflow?

10) Moving forward, what other virtual resources do you envision integrating in your practice?

11) We are planning on packaging all these recommendations to share your best practices with other specialty team members. How would you suggest we share your best-practices?
a) What suggestions do you have for other super users to expand the use of virtual resources within their specialty service?

12) What are a few of your performance measures? How are you using VR to meet your performance measures/annual review?

Extra question to nurse and patient education: We are doing this interview with specialists from different services (cardiology, dermatology, WH, PMR, SCI), what is your experience with these services? How do you facilitate integrating VR into their best practices?

Closure:

13) Based on your experiences as a super user, do you have anything you'd like to add about best practices that we didn't discuss?

Thank you for your time and important input!

(***ASK FOR REFERRALS**: Need to be a Super User)

Date:
PID:

VIRTUAL RESOURCE USE ACROSS THE CARE CONTINUUM

Pre-encounter → 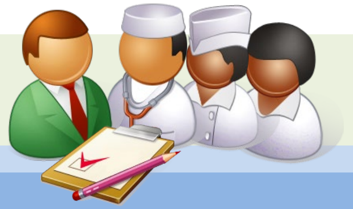 → **Post-encounter**

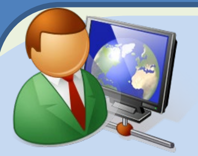

Medical Encounter

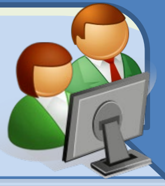

Pre-Appointment	Check-in	History/ Examination	Diagnosis	Treatment Plan & Care	Check-out	Post-Appointment
•Scheduling Medical Visit<br>•Preparing for a Medical Visit<br>•YOUR SUGGESTIONS	•Personal information updates<br>•Insurance information updates<br>•YOUR SUGGESTIONS	•Measurement of Health Indices/vitals<br>•Presenting Complaint<br>•History Taking<br>•Physical Examination<br>•YOUR SUGGESTIONS	• Labs/tests<br>• YOUR SUGGESTIONS	•Patient Education<br>•Counseling<br>•Prescriptions<br>•YOUR SUGGESTIONS	•Make follow up appointment<br>•YOUR SUGGESTIONS	•Treatment Plan Activities<br>•Seeking Information About Condition<br>•Follow Up with Health Care Provider<br>•YOUR SUGGESTIONS

Ongoing Health Management

Patient Activities		Provider Activities	
• Self Management Activities<br>• Monitoring Health Indices/vitals<br>• Managing Personal Health Information<br>• Managing Medications<br>• Seeking Information About Condition<br>• Identifying Need for a Medical Visit<br>• Communicating with Health Care Provider<br>• Labs/tests<br>• YOUR SUGGESTIONS		• Medication Reconciliation<br>• Patient Data Reporting<br>• Health Education<br>• YOUR SUGGESTIONS	

Virtual Resource List

1.ANNIE
2.Appointment Reminders
3.Appointment Reminders App
4.Blue Button
5.Blue Button App
6.CAN Risk Assessment
7.CPRS Flags
8.Healthy Living Assessments
9.Joint Legacy viewer
10.Journals
11.Labs and Tests
12.Non-VA Mobile Apps
13.Rx Refill
14.Secure Messaging
15.Telehealth
16.Telephone
17.VA and Non-VA YouTube videos for education
18.VA Mobile Apps
19.Veterans Health Library
20.VetLink Kiosks
21.VISTA - Imaging
22.Vitals Tracker
23.Wellness Reminders

RED = Removed
GREEN = Added

PID: _________________ Date: ___________ Observers: _____________ Service:_________

Observation of a Super User using Virtual Healthcare Resources

Good morning/afternoon, thank you for meeting with us today to demonstrate your proactive integrated use of VHR in your workflow.

We would like your permission to video record this session to fully document your workflow. We will later use these videos to share your best practices. Do we have your agreement?

As a reminder, today we are collecting data from you as a super user to understand your practices in proactive integrated use of virtual health resources. In this project, we define *proactive integrated use of virtual health resources* as a self-initiated approach to coordinated use of applicable virtual health resource systems for the purposes of coordinating and delivering timely high-quality patient-centered care. In today's meeting, we will have you use a scenarios with a mock patient to give you a context for demonstrating your approach to using virtual health resources.

Activity – Health care continuum revisited (10 min.)

We are going to start with a brief refresher of the VHRs use across the health care continuum. In the slides we emailed you previously, we present a consolidated rather comprehensive list of VHRs that we have collected during the first phase of the project from our participants, [**open slide and share on screen**]. The VHRs you and others in your service area already mentioned using, we formatted in light gray. Other VHRs that you haven't mentioned we kept in black. Please take a couple of minutes to review if there are additional resources on the list that you typically use but weren't listed in the first session.

[**Present slide #2**] In this slide, we also gathered the tasks and VHRs you and your service colleagues suggested you use as part of your proactive integrated approach across the continuum of care. In the top section of each phase. Below the black line are additional tasks other service providers reported using. We would like to review this consolidated information to assess whether we need to make adjustments to your input. are there any additional tasks you would like to add?

Demonstration think-a-loud (30-40 min.)

Next,

Based on our first session and previous conversations the scenario selected for today's think-aloud mock appointment is

[Enter selected scenario] ____

Please walk us through the process from pre encounter to encounter and post encounter indicating the tasks you complete, the VHR you use for completing these tasks, and ways these resources interconnect.

If you run into technical difficulties, we can proceed with a verbal description of the process and continue moving forward.

So, let's start with the pre-encounter phase…

Optional… Now moving to the encounter phase (check in, Hx, examination, diagnosis, treatment plan and care, check out),

Next is post encounter

What do you complete as an ongoing process?

Optional Directing questions during think aloud:

- Can you highlight the important features of this VHR?
- How do you navigate between these (VHR X and VHR Y) two systems? Why? Do you share this (screen/information) with the patient? Team members? Other providers? How?
- Why would this be done during in-person appointment? How?
- Are there scenarios in which you would select a different VHR to accomplish that task?
- What made you choose this particular VHR for the task? [for tasks that can be completed using several VHRs]
- How did you learn about the VHRs you are using?

Follow up questions (10 min.):

Based on the workflow you demonstrated today….

PID: _________________ Date: ___________ Observers: _____________ Service:_________

1. What features of proactive integrated VHR use are most useful to you?

2. How does a proactive integrated use of VHR make your work easier?
   a. Probe: How does it facilitate workflow?

3. How does the use proactive integrated use of VHR help you and your team to meet performance measures?

4. What would you change in VHRs to better fit your needs? Your service needs?

   a. If need to further probe: what would make the VHRs easier to use?

   b. Which other VHR might you use for inpatient setting? For what tasks?

5. How does proactive integrated use of VHR hinder your workflow?

6. How is this workflow using proactively VHRs unique to your service?

   a. What are some tasks/best practices unique to your that have benefited from your proactive use of virtual health tools?
   b. How is it applicable across services?

7. How do you establish this workflow as a best practice in your service?

We truly appreciate your time and effort in showing us how you use VHRs in a proactive integrated way. Is there anything you would like to share with us that wasn't covered earlier?

PID: ________________ Date: ___________ Observers: _____________ Service:_________

Demonstration/Observation checklist [To be completed after the session]:

1. Platform used: -
   - ☐ WebEx
   - ☐ VVC
   - ☐ Teams
   - ☐ Other: ________________________

2. Hardware that was used (check all that apply):

☐ Tablet ☐ Laptop ☐ Cell phone
☐ Desk computer ☐ Hard line phone ☐ Other: ______________________

2. Virtual Resources used (check all that apply and indicate how each were used).

	Virtual Resources	Description of How Used	Phase of the continuum
1			
2			
3			

3. How long was the scenario demonstration?

☐ 0-10 min ☐ 11-20 min ☐ 21-30 min ☐ 31-40 min
☐ 41-50 min ☐ 51-60 min ☐ Over 60 min

4. What is the intent or purpose of the scenario?

5. What were the challenges of using virtual resources?

☐ Connection was slow ☐ Image was unclear ☐ Sound was unclear
☐ Other:

6. What were the strengths of using virtual resources?

☐ Connection was fast ☐ Image was clear ☐ Sound was clear
☐ Other:

Any additional observations, thoughts: ______________________________________
