# Supplementary figures and images for: Super Users’ Reported Best Practices for Coordinating Proactive Integrated Use of Virtual Health Care Resources: Prospective Concurrent Mixed Methods Human-Centered Design Study
Source: J Med Internet Res. 2025 Nov 14;27:e81414. doi: 10.2196/81414 (PMC12663705; doi:10.2196/81414)

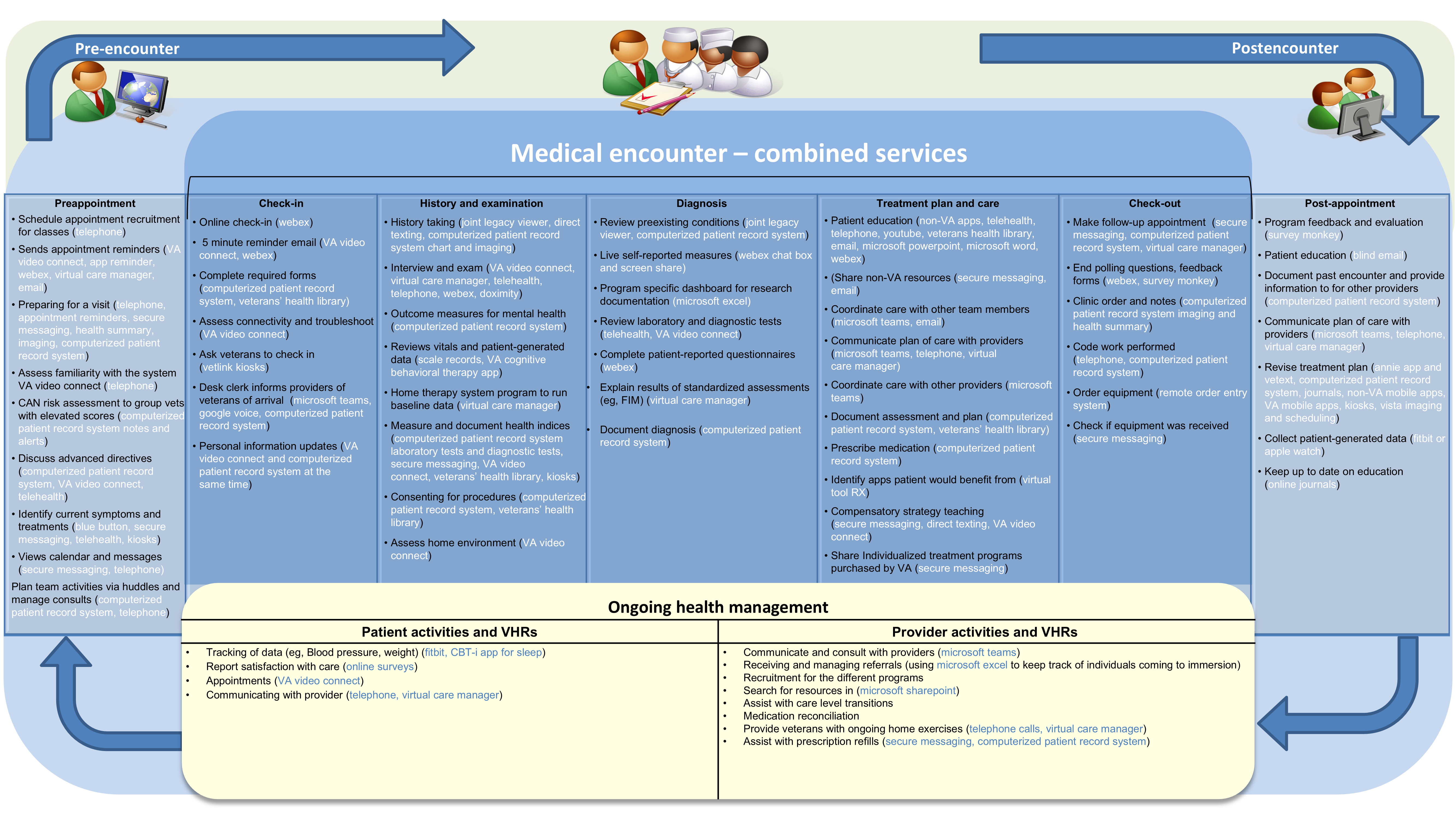

Supplement: Multimedia Appendix 2 [file jmir_v27i1e81414_app2.png]

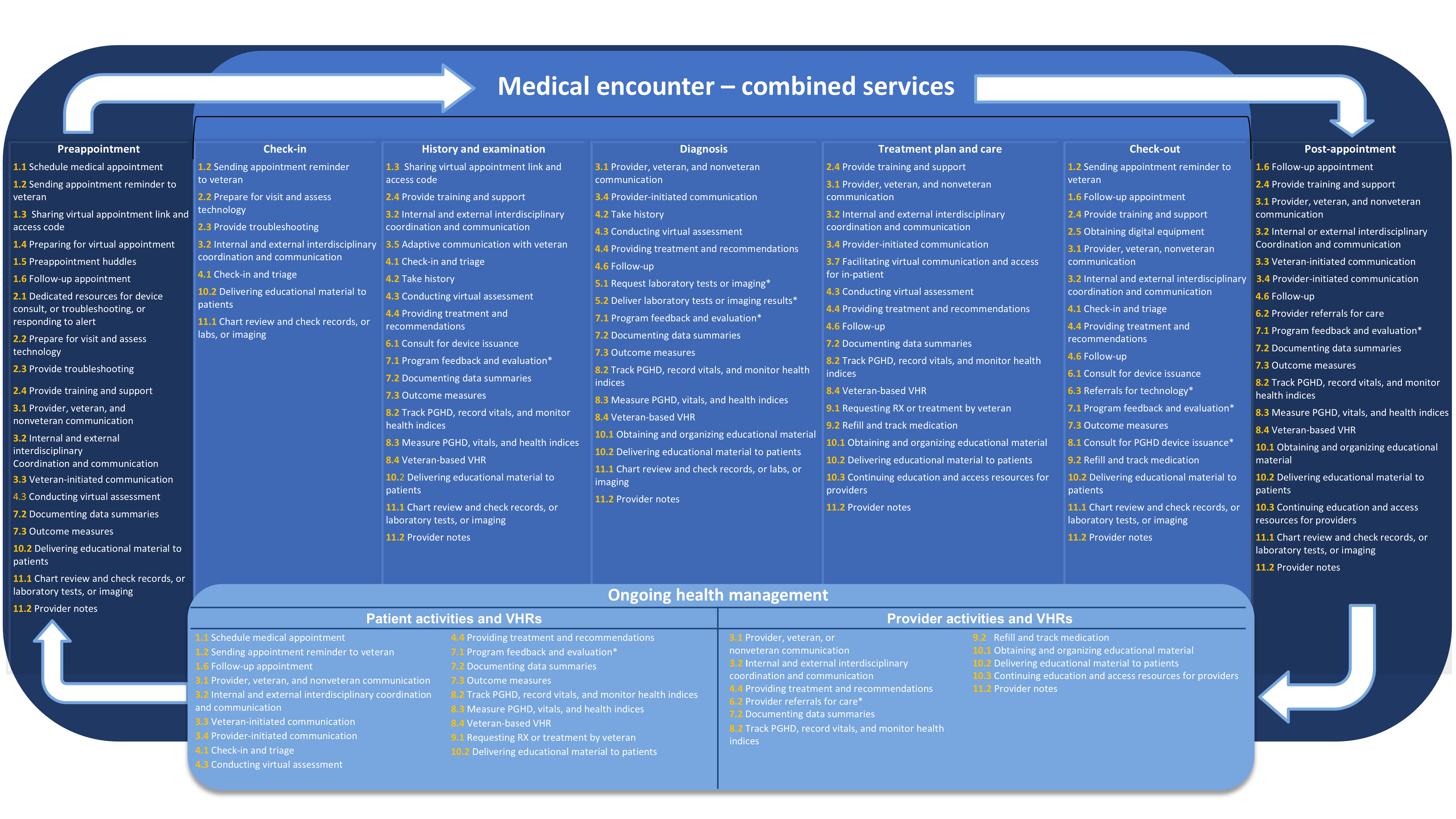

Supplement: Multimedia Appendix 6 [file jmir_v27i1e81414_app6.png]
