# Supplementary material for: Super Users’ Reported Best Practices for Coordinating Proactive Integrated Use of Virtual Health Care Resources: Prospective Concurrent Mixed Methods Human-Centered Design Study
Source: J Med Internet Res. 2025 Nov 14;27:e81414. doi: 10.2196/81414 (PMC12663705; doi:10.2196/81414)
Supplement: Multimedia Appendix 3 [file jmir_v27i1e81414_app3.pdf]

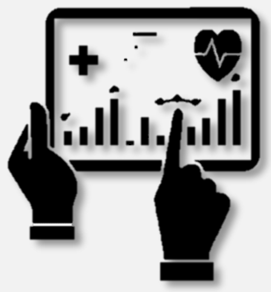

Specialty Services Best Practices Identification Workbook

Use the following pages in this workbook to help our team identify the best practices stemming from the current practices of Super User Specialty Services

How to use this best practices identification workbook

How do I use this workbook and what actions do I need to complete?

You will see the following layout on the consecutive pages of this workbook for each Super User service. Underneath the color-coded care continuum phases are activities and tasks that correspond. Please read the current tasks that correlate and choose the most appropriate selection from the drop-down: **1) Current Practice 2) Best Practice or 3) De-Implement Practice 4) Undecided 5) No Response.** Undecided can be chosen if further collaboration and/or information is needed before an appropriate decision can be made, while No Response indicates that the task as a current or best practice is unknown at this time. Utilize the text box underneath each activity category to add any notes that would be beneficial for our team to know.

CARDIOLOGY

Current Practices Identified

Pre-Encounter Activities

Color-Coded Care Continuum Phase

1 Appointment Reminders

Activity Category

- Provider sends Veteran appointment reminders via **Outlook *(Microsoft Suite)*** in addition to a courtesy reminder **telephone** call by assigned volunteer team member (who also handles the logistics of the virtual appointment).

Current Practice

Choose the most appropriate practice for each task within the activity category.

Description of task with Bolded VHR.

Directly type any notes that are appropriate for the team to know when developing the list of best practices for the tasks and activities within the care continuum phases.

Cardiology: Current Practices

For each task listed underneath the activity, choose the most appropriate practice from the drop-down: Current Practice, Best Practice, De-Implement, Undecided or No Response. Use the comment box for any notes.

CARDIOLOGY

Current Practices Identified

Pre-Encounter Activities

1 Appointment Reminders

- Provider sends Veteran appointment reminders via **Outlook *(Microsoft Suite)*** in addition to a courtesy reminder **telephone** call by assigned volunteer team member (who also handles the logistics of the virtual appointment).

No Response

2 Preparing for a visit

- Assigned team member provides Veteran with training on how to do virtual visits using **VA & Non-VA YouTube Videos, VA & Non-VA apps**, and provides a demonstration.

No Response

Medical Encounter Activities

1 Medical Encounter Check-In

- Provider conducts virtual visits using **VA Video Connect *(VVC)*** and **Virtual Care Manager *(VCM)***, with Veterans at home or through Clinical Video Telehealth (CVT).

No Response

- Provider uses links provided on **Virtual Care Manager *(VCM)*** (when connecting) as a virtual check- in to log on.

No Response

- Provider communicates via **Teams *(Microsoft Suite)*** if **Vetlink Kiosk** goes down, so clerks can send messages.

No Response

Current Practices Identified

Medical Encounter Activities *(Continued)*

2 History intake, Review Diagnostic Data, Perform Interview and Exam

- Provider established virtual group (shared appointment) health lifestyle and promotion using **VA Video Connect *(VVC)***; this was a transition from in-person since the onset of COVID-19. — No Response
- Provider and Veteran uses **Virtual Tool Rx *(app)*** specific to the Veteran's needs and goals. — No Response
- Provider completes prescription refills or prescriptions through **CPRS**. — No Response
- Provider reviews Veterans health history on **CPRS/JLV** when cardiology procedures were done elsewhere in the VA system and **VISTA Imaging *(CPRS)*** for those Veterans with records from private practice. — No Response
- Provider uses **CPRS** to put in orders such as labs, consults and procedures. — No Response
- Provider uses **VA Video Connect *(VVC)*** for video and **Doximity** with a **telephone** for a phone call visit with Veterans. — No Response

3 Review Veteran Generated Health Data (PGHD), Implement Protocols, Measure & Document Health Indices

- Veteran generated data collected through **Apple watch/Fitbit, Alivecor *(app)***, and/or **Pulse Oximeter** machine to collect vitals and cardiac information. All can be and some are currently being integrated to automatically upload that information to a VA portal. — No Response
- Provider reviews **VA National Surveillance *(Website)*** for health information gathered from Veterans devices (pacemakers, defibrillators). — No Response
- Provider reviews **Annie app for Clinicians *(app)*** to see Veteran generated health data. — No Response
- Provider uses **Sync My Health Data *(app)*** to gather information entered from the Veteran and his or her medical devices . — No Response

Current Practices Identified

Medical Encounter Activities *(Continued)*

4 Follow-up Appointment, Referrals, D/C Summaries

- Provider uses **Return to Clinic *(CPRS)*** to create a return to clinic for veterans. — No Response
- Provider enters summaries and documentation of Veterans appointment to **CPRS**. — No Response

5 Consult for Device Issuance

- Provider uses the **Consults Tool (*CPRS*)** to generate a digital divide consult to offer technology devices to the Veteran. — No Response

Post-Encounter Activities

1 Provider = Veteran/Non-Veteran Communication

- Veteran sends updates to provider post operation via **Secure Messaging *(MHV)*** in ***My HealtheVet*** or **telephone**. — No Response
- Provider reviews Veterans BP uploads on **My HealtheVet *(MHV)***. — No Response

2 Provider – Provider Communication (Sharing Veteran information and info with other providers)

- Team members use **Teams *(Microsoft Suite)*** to communicate with other team members. — No Response

CARDIOLOGY

Current Practices Identified

Ongoing Veteran Activities

1 Tracking Veteran Generated Health Data

- Vitals taken by **Apple Watch/Fitbit** and/or **Pulse Oximeter** machine. All can be and some are currently being integrated to automatically upload that information to a VA portal. — No Response
- Veteran enters vitals (BP, HR) to **Annie app for Veterans *(app)*** and any activities are generated as data in a dashboard for the providers to view in the **Annie app for Clinicians *(app)***. — No Response
- Veteran uploads health data into **Sync My Health Data *(app)*** from medical devices. — No Response
- Provider reviews Veterans' data from the **Sync My Health Data *(app)***. — No Response

2 Request Medication Refill

- Veteran uses **Secure Messaging *(MHV)*** in ***My HealtheVet*** to request a medication refill. — No Response
- Veteran uses **Rx Refill *(MHV)*** to request an existing medication refill. — No Response
- Provider prescribes medication refill and sets follow-up appointments via **CPRS**. — No Response

Current Practices Identified

Ongoing Veteran Activities *(Continued)*

3

Record Vitals, BP, Glucose & Monitor Health Indices

- Provider gathers data (vitals, blood glucose, scale) from **Telehealth** and enters a note to allow tracking Veterans' progress over time.

No Response

4

Veteran – Provider Communication (Seek Information about condition; Assess Need for Visit)

- Provider uses **Secure Messaging *(MHV)*** in ***My HealtheVet*** and **telephone** to relay information from Veterans on their progress after the treatment plan.

No Response

Ongoing Provider Activities

1

Provider Notes (Vital Signs from Telehealth are Compiled)

- **Telehealth** (vitals, blood glucose, scale) gets compiled into a note that tracks progress over time and is saved on **CPRS**.

No Response

2

Communicate and Consult with Healthcare Providers

- Provider developed a resource list for colleagues requesting solutions to technology issues that facilitate VHR use.

No Response

CARDIOLOGY

Current Practices Identified

Activities Across All Timeframes

1 Schedule Medical Appointments

- Provider answers questions and schedules medical appointments using **Secure Messaging *(MHV)*** in ***My HealtheVet***. — No Response
- HAS schedules the appointment and uses **Outlook *(Microsoft Suite)*** to send the Veteran an email with an attached PDF file of educational handouts. — No Response

2 Provider Accesses, Identifies, Packages & Sends Veteran Education Materials (i.e.: general, diagnosis, prognosis & Rx)

- Provider locates Veteran education and resources via **VA YouTube videos** and **MyHealtheVet**. — No Response
- Provider uses **Nursing Link** to search articles and personalized educational materials for the Veteran in both English and Spanish. — No Response
- Provider uses **Outlook *(Microsoft Suite)*** to email educational materials to the Veteran. — No Response
- Provider shares **VA YouTube videos** with Veterans. — No Response

3 Interdisciplinary Coordination & Communication (Provider to Provider)

- Provider uses **Teams *(Microsoft Suite)*** to communicate with other team members throughout the VA system. — No Response

SCI:
Current Practices

For each task listed underneath the activity, choose the most appropriate practice from the drop-down: Current Practice, Best Practice, De-Implement, Undecided or No Response. Use the comment box for any notes.

SCI

Current Practices Identified

Pre-Encounter Activities

1 Appointment Reminders

- Telehealth Coordinator (RN) calls Veteran on the **telephone** to assess if they are ready to perform a video appointment and assess their familiarity of connecting to a video conference.

No Response

- Providers (i.e., Clinical care coordinator, Social worker, Physician) uses **Teams *(Microsoft Suite)*** to discuss with Veterans about what the Veteran program has to offer and what they can expect from the program to make a decision if they want to enter the program via **VA Video Connect *(VVC)***.

No Response

2 Chart Review, Update Personal Information, Perform Assessment

- Providers receives and reviews a hard copy of the Veteran's file packet shared by the doctor. **CPRS** is also reviewed.

No Response

3 Preparing for a Visit

- Telehealth Coordinator (RN) sets up a test call where the Veteran is assisted to **VA Video Connect**.***(VVC)*** Documents the test call action in **CPRS** and then, once identifies the Veteran as video capable, sets up a link in **Virtual Care Manager *(VCM)*** for Veteran's future use.

No Response

- Provider uses **telephone** during the exam.

No Response

- Telehealth Coordinator (RN) and HAS contacts Veteran via **telephone** to make sure they have equipment and performs a test run to make sure they are ready for the appointment.

No Response

- Nurses work with Veterans to coordinate travel to the facility.

No Response

Current Practices Identified

Pre-Encounter Activities *(Continued)*

4 Team Conference & Huddles

- Provider uses **Teams *(Microsoft Suite)*** to coordinate annual visit. — No Response
- Provider uses **Teams *(Microsoft Suite)*** or **telephone** to communicate. — No Response
- Provider uses **Teams *(Microsoft Suite)*** to conduct a huddle, if Veteran needs a follow-up. — No Response

Medical Encounter Activities

1 Medical Encounter Check-In

- Telehealth Coordinator (RN) uses **VA Video Connect *(VVC)*** with the Veteran for any troubleshooting assistance and make sure they are appropriately connected and details the process for how the annual evaluation will be conducted virtually. — No Response
- Telehealth Coordinator (RN) uses **CPRS** to check-in Veteran. — No Response
- Providers use **Care Assessment Needs (CAN) Risk Assessment *(CPRS)*** to help with deciding who needs a more urgent appointment. — No Response

2 History intake, Review Diagnostic Data, Perform Interview and Exam

- Provider uses **Echo Device *(VVC)*** from a live link sent by the Veteran through **VA Video Connect *(VVC)*** and uses a headset to listen to the Veteran's heart and lungs. — No Response
- Provider trialing out using **Stethoscope *(VVC)*** that interacts with Veteran to listen to their lungs and heart virtually. — No Response
- Telehealth Coordinator (RN) have **Teams *(Microsoft Suite)*** set up with a list of links for all providers associated with the appointment to access and coordinate who is next in the queue to connect via **VA Video Connect *(VVC)***. — No Response
- Provider uses **3D camera** to assist with tracking wound care. — No Response

Current Practices Identified

Medical Encounter Activities *(Continued)*

3

Review Veteran Generated Health Data (PGHD), Implement Protocols, Measure & Document Health Indices

- Provider uses a **Blood Pressure Machine** which populates the vital signs directly into **CPRS** to avoid errors. No Response
- Provider uses **Apple Watch/FitBit** to look at sleep patterns to monitor sleep cycle and provide feedback. No Response
- Provider tracks bladder and bowel care through **Annie app for Clinicians *(app)***. No Response

4

Assess Home Environment

- Provider uses **VA Video Connect *(VVC)*** to incorporate Whole Health (i.e., nutrition) as part of the annual evaluations, specifically to view the Veteran's refrigerator. No Response

5

Provider-Veteran Care Coordination & Communication

- Telehealth Coordinator (RN) uses **telephone** to conduct follow-up appointments. No Response
- Telehealth Coordinator (RN) uses **Return to Clinic *(CPRS)*** for any return to clinic orders. No Response

6

Consult for Device Issuance

- Telehealth Coordinator (RN) uses the **Consults Tool *(CPRS)*** to generate a digital divide consult and collaborates with a Social Worker to issue the Veteran an **iPad**. No Response

SCI

Current Practices Identified

Post-Encounter Activities

1 Provider = Veteran/Non-Veteran Communication

- Veterans use **Image Viewing Solution *(app; VistA)*** to send still images of a wound to their providers. Provider uses **VistA Imaging (*CPRS*)** to view and comment on the wound, which can automatically update to the Veteran's chart in **CPRS**. — No Response
- Providers follow up on medication management every 3 months using **telephone** for renewals and updates. — No Response

2 Provider – Provider Care Coordination & Communication

- Provider uses **Teams *(Microsoft Suite)*** or **telephone** to communicate with the pharmacy or other staff for medications and supplies for the Veteran prior to D/C. — No Response
- Provider uses **CPRS** for D/C summaries. — No Response

3 Check Records, Labs, Imaging & Procedures

- Provider uses **Flags *(CPRS)*** for reminders to order labs, which includes a function for an automatic follow-up appointment. — No Response

Current Practices Identified

Ongoing Veteran Activities

1

Veteran Generated Health Data

- Veteran enters bladder and bowel data to the **Annie app for Veterans *(app)***.

No Response

2

Request Medication Refill

- Veteran uses **Secure Messaging *(MHV)*** in **My HealtheVet** to request prescription refills

No Response

3

Record Vitals, BP, Glucose & Monitor Health Indices

- Provider uses **Telehealth Program** to monitor health indices and if BP or sugars are elevated, the levels are automatically uploaded to **CPRS**.

No Response

SCI

Current Practices Identified

Ongoing Veteran Activities *(Continued)*

4 Veteran – Provider Communication (Seek Information about condition; Assess Need for Visit)

- Veteran use **Secure Messaging *(MHV)*** *in* ***My HealtheVet*** to contact staff about anything related to their medical care.

Undecided

Activities Across All Timeframes

1 Provider Accesses, Identifies, Packages & Sends Veteran Education Materials (i.e.: general, diagnosis, prognosis & Rx)

- Provider uses **VISTA-Get Well Network, CPRS/JLV, MyHealtheVet *(MHV)*** & **Secure Messaging *(MHV)*** to identify resources. All these resources can be used for Veteran education. If Veteran doesn't have a Premium Account, provider can begin the process of upgrading.

No Response

2 Interdisciplinary Coordination & Communication (Provider to Provider)

- On-floor staff can be reach via **telephone** or **Vocera**.

No Response

- Provider uses **Teams *(Microsoft Suite)*** when communicating with multiple disciplines at once.

No Response

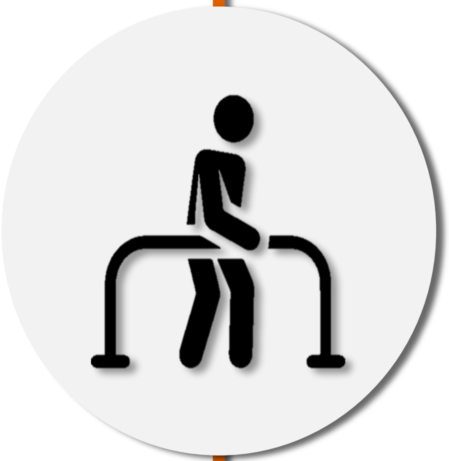

PM&R:
Current Practices

For each task listed underneath the activity, choose the most appropriate practice from the drop-down: Current Practice, Best Practice, De-Implement, Undecided or No Response. Use the comment box for any notes.

PM&R

Current Practices Identified

Pre-Encounter Activities

1 Appointment Reminders

- Provider uses **Secure Messaging *(MHV)*** in **My HealtheVet, appointment reminders *(MHV)*, telephone,** or **Doximity** with **telephone** to remind Veterans of upcoming appointments. No Response
- **Outlook** is used by providers, per Veteran choice, for appointment reminders because it is easy for Veterans with TBI to transpose appointments to the Outlook calendar. No Response

2 Chart Review, Update Personal Information & Perform Assessment

- **CPRS** is accessed by providers to review Veteran history and is kept open during the encounter with the Veteran while using the **VA Video Connect *(VVC)***. No Response

3 Preparing for a Visit

- Provider uses **telephone** or **Doximity** with **telephone,** and **VA Video Connect *(VVC)*** to make sure equipment is working prior to the appointment. No Response

4 Team Conference/Huddles

- Provider uses **Teams *(Microsoft Suite)*** to conduct team meetings and huddles with other providers. No Response

PM&R

Current Practices Identified

Medical Encounter Activities

1 Medical Encounter Check-In

- Provider uses **telephone** with **Doxmity** to remind Veterans to complete the pre-check screener during check-in for the appointment. — No Response
- When Veterans do not show up to an appointment, the provider uses **CPRS** to find Veterans phone number and then **Google Voice** to call the Veteran. — No Response
- Provider uses **Teams *(Microsoft Suite)*** to know that the Veterans are there for the appointment and to instant message the clerk to update Veteran records if needed. — No Response
- Provider uses **Consults Management *(CPRS)*** to check-in Veterans. — No Response

2 History intake, Review Diagnostic Data, Perform Interview and Exam

- Provider uses **CPRS** to review chart and document encounter with Veterans. — No Response
- Provider sends materials to Veterans via **direct texting**, **Outlook**, or **Secure Messaging *(MHV)***. — No Response
- Provider uses **VA Video Connect *(VVC)*** to perform testing and see Veterans in their home environment. — No Response
- Provider uses **Home Therapy System** to perform baseline visual tests with Veterans. — No Response
- Provider uses **VA Video Connect *(VVC)*** to lock therapy session with Veterans so there are no unnecessary intrusions from others and to perform 3-way calling. — No Response
- Provider uses **VA Video Connect *(VVC)*** to perform 3-way calling with Veterans and other providers or family members. — No Response
- Providers uses **VA and Non-VA YouTube videos** for training and education with Veterans. — No Response
- Provider uses **direct texting** to communicate with Veterans who are non-verbal. — No Response
- Provider uses **VA Video Connect *(VVC)*** to invite family members into sessions with Veterans. — No Response
- Provider uses **Teams *(Microsoft Suite)*** to pull in providers during the session with Veterans. — No Response
- Provider uses **Home Therapy System** to run baseline data on Veterans to design a treatment program. — No Response

PM&R

Current Practices Identified

Medical Encounter Activities *(Continued)*

3

Provider – Veteran Care Coordination & Communication

- During the medical encounter, provider uses **Secure Messaging *(MHV)*** in ***My HealtheVet*** to send education handouts to Veterans.

No Response

4

Follow-up Appointment, Referrals, D/C Summaries

- Provider uses **Return to Clinic *(CPRS)*** to enter orders in Veteran charts.

No Response

- Provider uses **Secure Messaging *(MHV)*** in ***My HealtheVet*** to make follow-up appointments with Veterans.

No Response

5

Consult for Device Issuance

- Provider uses **Secure Messaging *(MHV)*** in ***My HealtheVet*** to confirm that the Veterans received equipment ordered.

Undecided

PM&R

Current Practices Identified

Post-Encounter Activities

1 Provider = Veteran/Non-Veteran Communication

- Veterans send updates related to care to provider via **Secure Messaging *(MHV)*** in ***My HealtheVet*** or **telephone** post-appointment. — No Response
- Provider sends Veterans **non-VA online resources** related to care. — No Response
- Provider uses **URL generator** to send a static link via **VA Video Connect *(VVC)*** to Veterans who are unable to navigate Outlook. — No Response
- Provider sends Veterans education information via **Secure Messaging *(MHV)*** in ***My HealtheVet*** or **Outlook**. — No Response
- Provider encourages Veterans to use **Secure Messaging *(MHV)*** in ***My HealtheVet*** to communication with providers because it is tracked. — No Response

2 Provider – Provider Communication (Sharing Veteran information and info with other providers)

- Provider uses **Teams *(Microsoft Suite)*** to reach out to other providers to discuss Veteran care. — No Response
- Provider uses **CPRS, telephone, Teams *(Microsoft Suite)* or VA Video Connect *(VVC)*** to give information related to Veteran care to providers. — No Response
- Provider uses **online journals *(websites)*** for up-to-date education. — No Response

3 Provide Program Feedback & Evaluation

- Provider sends satisfaction surveys to Veterans using **Survey Monkey** via **Outlook**. — No Response

PM&R

Current Practices Identified

Ongoing Veteran Activities

1

Veteran – Provider Communication (Seek Information about condition; Assess Need for Visit)

- Veterans use **Secure Messaging *(MHV)*** in ***My HealtheVet*** to contact team and express concerns and needs to providers.

No Response

Ongoing Provider Activities

1

Communicate & Consult with Healthcare Providers

- Provider uses **Teams *(Microsoft Suite)*** to communicate with other disciplines regarding Veteran care.

No Response

- Provider uses **Teams *(Microsoft Suite)*** to support other providers with troubleshooting technology issues.

No Response

PM&R

Current Practices Identified

Activities Across All Timeframes

1 Schedule Medical Appointment

- Provider uses **Secure Messaging *(MHV)*** in ***My HealtheVet*, telephone,** and **VA Video Connect *(VVC)* Now** to make follow-up appointments with Veterans.

No Response

2 Provider Accesses, Identifies, Packages & Sends Veteran Education Materials (i.e.: general, diagnosis, prognosis & Rx)

- Provider sends Veterans at-home exercises using **Outlook** or **Virtual Care Manager (*VCM*)**.

No Response

- Provider sends educational materials to Veterans using **VA Video Connect *(VVC)*, Secure Messaging *(MHV)*** in ***My HealtheVet*** or **Outlook *(Microsoft Suite)*** (encrypted).

No Response

3 Interdisciplinary Coordination & Communication (Provider to Provider)

- Provider uses **Teams (*Microsoft Suite*)**, **Virtual Care Manager (*VCM*)**, or **telephone** to communicate with other providers.

No Response

Whole Health: Current Practices

For each task listed underneath the activity, choose the most appropriate practice from the drop-down: Current Practice, Best Practice, De-Implement, Undecided or No Response. Use the comment box for any notes.

Whole Health

Current Practices Identified

Pre-Encounter Activities

1 Appointment Reminders

- Provider uses **VA Video Connect *(VVC)*** for appointment reminder if the Veteran was absent for the last visit. No Response
- MSA uses **Outlook** to block off time and **Virtual Care Manager (*VCM)*** for calendar invite reminders and to make sure provider isn't double booked with another appointment. MSA also uses **Virtual Care Manager (*VCM)*** for providers only to place a reminder note to the provider about the Veteran's appointment. No Response
- MSA or Provider uses **telephone** to remind the Veteran if he/she forgets an appointment. No Response
- Provider opens message in **Secure Messaging *(MHV)*** in ***My HealtheVet***, reads the exchange and adds findings and goes to **Outlook *(Microsoft Suite)*** to pull out the invitation that was created by another provider. The provider can then join the meetings. No Response
- Provider uses **WebEx** and clicks the invite and remind button so the Veteran can receive an email reminder 5 minutes prior to the appointment. To enter the visit, they are being reminded on multiple fronts. No Response

2 Chart Review, Update Personal Information, Perform Assessment

- Provider receives an **alert *(CPRS/JLV)*** or addendum in **CPRS** then provider will give a call and go through the process. No Response
- Provider uses the **Care Assessment Need (CAN) Risk Assessment *(CPRS)*** to assess who has an elevated CAN score to regroup Veteran who fulfill that criteria. No Response
- Provider opens **Secure Messaging *(MHV)*** in ***My HealtheVet*** to read exchanges with the Veteran. No Response
- Provider reviews **Flags; Labs & Tests; Consults Management (*CPRS*)**, as well as **Telehealth** and **CPRS** for Veteran chart review. No Response

Current Practices Identified

Pre-Encounter Activities *(Continued)*

3

Preparing for a Visit

- Dedicated IT team member uses **CPRS** to look at a Veteran's scheduled appointment to figure out when he/she needs to contact before their next appointment. Dedicated IT team member then goes to **notes & alerts *(CPRS)*** to scan any notes if they had any issues previously and the outcomes. Dedicated IT Team member then checks if the Veteran has a VA-issued iPad. When looking at the notes, gives dedicated IT team member an idea of the platform the Veteran is comfortable using and finds out what equipment Veteran already has issued. — Undecided
- Dedicated IT Team member opens **CPRS** & **ROES** to prepare for the call while simultaneously looking at face sheet for Veteran telephone number and email address. — No Response
- Dedicated IT Team member uses **Cisco Jabber** or **Doximity** with a **telephone** to call the Veteran, which include caller ID from a VA number. During this call, dedicated IT team member troubleshoots with the Veteran while using **VA Video Connect *(VVC)***. — No Response
- Provider uses **PowerPoint *(Microsoft Suite)*** and **Word *(Microsoft Suite)*** to prepare for the group session. — No Response
- While on the **telephone**, dedicated IT team member informs the Veteran to complete a test call. — No Response
- Dedicated IT team member may receive a viewer alert from **notes & alerts *(CPRS)*, Secure Messaging *(MHV)*** in ***My HealtheVet***, **Teams *(Microsoft Suite)*** or **Outlook *(Microsoft Suite)*** about a Veteran having difficulty connecting or needs set up. — No Response
- Dedicated IT team member uses the serial number from **Remote Entry Order System *(CPRS)*** and copies and place in **URL generator** to create a static link to directly connect to the Veteran. — Undecided

Medical Encounter Activities

1

Medical Encounter Check-In

- Provider uses **WebEx** and clicks the invite and remind button so the Veteran can receive an email reminder 5 minutes prior to the appointment. To enter the visit, they are being reminded on multiple fronts. This includes a dedicated IT team member who comes into each virtual group to help with any technological issues. — No Response
- Provider or dedicated IT team member sends the appointment link from **Virtual Care Manager (*VCM)*** while on the **telephone** with the Veteran and then sends a second email reminder on the day of the appointment and may send a third **Outlook** email reminder a few minutes before the appointment in case the Veteran still can't locate the email link. The purpose of this day-of email is to have the appointment link at the top of their inbox. — No Response
- Provider uses **Outlook** calendar to view **WebEx** to start the virtual meeting. Before the session begins, the provider gives a review of **WebEx** functionalities and features with the Veteran. The Provider then uses the **telephone** to show how and instruct the Veteran to download the **CBT-i-Coach** and how to enter data to the app. — No Response
- If in-person appointment, Provider asks the Veteran to check in via the **Vet Link kiosk.** — No Response

Current Practices Identified

Medical Encounter Activities *(Continued)*

2

History Intake, Review Diagnostic Data, Perform Interview & Exam

- Provider reviews **Flags; Labs & Tests; Consults Management *(CPRS)*** as well as **Telehealth** and **CPRS**. — No Response
- Provider uses **WebEx** to conduct the appointment and asks for current weight and gathers mental health, psychosocial and medical history as well as specific presenting complaints. — No Response
- Provider runs videos on **PowerPoint *(Microsoft Suite)* or WebEx**; VA Video Connect (VVC) lacks capability for video demonstrations. — No Response
- While using **WebEx**, provider shares screen with a self-report measure to answer questions. This is only done in a 1:1 visit, not a group format. — No Response
- While on **VA Video Connect *(VVC)*** or **WebEx**, provider asks for Veteran to give informed consent at the beginning of the appointment and talks about confidentiality. The plan is then documented in **Notes** & Alerts ***(CPRS)***. — No Response

3

Review Veteran Generated Health Data (PGHD), Implement Protocols, Measure & Document Health Indices

- Provider utilizes information on their service connection disability rating and vitals that are generated in **CPRS** and **CBT-i-Coach** to ensure they are improving over the course of their time together. — No Response
- Provider uses **Apple Watch/FitBit** data for Veteran heart rate monitoring when they are having anxiety. — No Response

Current Practices Identified

Medical Encounter Activities *(Continued)*

4

Provider – Veteran Care Coordination & Communication

- Provider or dedicated IT team member uses **Doximity** with **telephone** when **Virtual Care Manager (*VCM)*** or **VA Video Connect *(VVC)*** is down.

No Response

- Provider or dedicated IT team member Uses **WebEx** for Android users when they can't connect with **Virtual Care Manager (*VCM)***.

No Response

5

Document Outcome Measures

- Provide uses **Survey Monkey** if a Veteran says they feel uncomfortable talking in the group.

No Response

- Provider receives a generated email from **Qualtrics** if someone endorses suicidal ideation.

No Response

- Provider utilizes **Mental Health Assistance *(CPRS)*** to gather data under self-report measures for a CBTI group, evaluation or individual assistance. Mental health assistance has the tests ready and can receive the inputted answers to create the results. The survey answers are automatically populated in **Notes** & Alerts ***(CPRS)***.

No Response

- Provider sends a personal health inventory questionnaire via **Secure Messaging *(MHV)*** in ***My HealtheVet***. If the Veteran does not have SM, the provider uses **Outlook *(Microsoft Suite)*** (do not reply) to send.

No Response

- Provider uses **WebEx** and **Survey Monkey** for polling. Once the survey is completed online, the provider accesses the results. The link to the survey is sent via **Outlook *(Microsoft Suite)*** (blind copy). When survey results are ready, the Veteran receives an email notification.

No Response

- Provider uses **Virtual Care Manager (*VCM)*** to find educational materials.

No Response

Whole Health

Current Practices Identified

Medical Encounter Activities *(Continued)*

6

Follow-up Appointment, Referrals, D/C Summaries

- Provider uses **Return to Clinic *(CPRS)*** to set another appointment. No Response
- Provider uses **Virtual Care Manager (*VCM)*** and **Outlook *(Microsoft Suite)*** to schedule a follow-up appointment. No Response

7

Consult for Device Issuance

- Provider submits a request in the **LEAF** system to order a **FitBit**. No Response
- Dedicated IT team member uses **Remote Entry Order System *(CPRS)*** for tablet or **iPad** orders and uses this to track the order when delivered to set up a follow-up appointment to instruct Veteran on how to set up and use the device. No Response
- Provider uses **Remote Entry Order System *(CPRS)*** to submit orders and requests. This can be used to prescribe certain equipment like a cervical pillow for sleep. No Response

Post-Encounter Activities

1

Provider – Provider Communication (Sharing Veteran information and info with other providers)

- Provider uses **Teams *(Microsoft Suite)*** to receive chats about a specific Veteran. Provider perceives this is not secure, so provider sends encrypted information via **Outlook *(Microsoft Suite)*** and then can chat on **Teams *(Microsoft Suite)*.** No Response

2

Provide Program Feedback & Evaluation

- Provider uses **WebEx** and **Survey Monkey** for feedback forms. **Outlook *(Microsoft Suite)*** (blind copy) and the **WebEx** chat box are used to send digital **PowerPoint *(Microsoft Suite)*** presentations. No Response

Whole Health

Current Practices Identified

Ongoing Veteran Activities

1 Tracking Veteran Generated Health Data

- Provider uses **CBT-i-Coach *(app)*** to ensure Veterans are improving over the course of time together with Provider. — No Response
- Provider uses **CBT-i-Coach *(app)*** to track sleep and/or FitBit to track food intake or steps. — No Response
- Provider uses **FitBit** to track health, sleep, steps and pulse. — No Response

2 Record Vitals, BP, Glucose & Monitor Health Indices

- Provider uses **CBT-i-Coach *(app)*** for Veteran to download to follow along with during the session. This app mimics what they do on a regular basis but allows Veterans to add information and goals from their phone. This app sends an alert/reminder that can be set daily or hourly to remind to drink water or walk. — No Response
- Provider uses **CBT-i-Coach *(app)*** and **PTSD Coach *(app)*** for self-management of activities like meditation and mindfulness. — No Response
- Provider uses **CBT-i-Coach *(app)*** to track sleep on a weekly basis, which provides data regarding their weekly sleep schedule so the provider can adjust the schedule in the group format. — No Response

3 Veteran – Provider Communication (Seek Information about condition; Assess Need for Visit)

- Veteran might take a photograph of exam results and share with the provider using **Secure Messaging *(MHV)*** in ***My HealtheVet*** — No Response

Whole Health

Current Practices Identified

Ongoing Provider Activities

1

Provider Notes (Compiling Vital Signs from Telehealth)

- Provider or dedicated IT team member uses **Group Notes *(CPRS)*** to make multiple notes at once or individualized notes within the program, which can automatically link with **CPRS**. No Response
- Provider completes encounter form on **CPRS** to document workload. This is beneficial for document effort performance. No Response

2

Communicate & Consult with Healthcare Providers; Receive & Manage Referrals

- Provider or dedicated IT team member uses **Teams *(Microsoft Suite)*** or **Outlook *(Microsoft Suite)*** to consult with other providers on the team. No Response
- Provider uses **Virtual Care Manager (*VCM)*** because it saves time, easy to use and loads quickly. The provider can see all video appointments in one spot and doesn't have to open CPRS which may take longer to load. No Response
- Provider uses **Teams *(Microsoft Suite)*** to reach out to another colleague to consult about specific questions or matters. If more background information is needed, provider will visit **JLV *(CPRS/JLV)***. No Response
- Provider or dedicated IT team member uses **Virtual Care Manager (*VCM)*** or **telephone** to communicate with other providers. No Response
- Provider uses **Excel *(Microsoft Suite)*** to manage referrals for Veterans from THRIVE who are interested in THRIVE Immersion or Cognitive Behavioral Therapy for Insomnia. No Response
- Provider gathers the list of referrals on a **Word *(Microsoft Suite)*** document saved in **Teams *(Microsoft Suite)*** under a secure/locked Teams files folder that is password protected and calls the Veteran via the **telephone** to explain the program and schedules them. No Response
- Referrals from CBTM don't go through consult, and the provider receives an alert in a **note** or addendum to the note ***(CPRS)*** and then will give a **telephone** call to the Veteran to go through the process. No Response

Current Practices Identified

Ongoing Provider Activities *(Continued)*

3

Identification & Use of Professional Resources

- Provider views the Weekly Telehealth VHA service updates **SharePoint *(Microsoft Suite)***. If there are any new VCM updates, the provider would inform the champions of his or her service.

No Response

- Provider uses **online journals *(websites)*** to stay abreast on education.

No Response

Activities Across All Timeframes

1

Schedule Medical Appointment

- Provider uses **telephone** to schedule a medical visit for recruitment.

No Response

- Provider or MSA schedules a visit through **CPRS, VA Video Connect *(VVC)*** or **WebEx.**

No Response

- Provider gathers the list of referrals on a **Word *(Microsoft Suite)*** document saved in **Teams *(Microsoft Suite)*** under a secure/locked **Teams *(Microsoft Suite)*** files folder that is password protected and calls the Veteran on the **telephone** to explain the program and schedule them.

No Response

- Dedicated IT team member uses **telephone** to call the Veteran and sends the appointment link via **Outlook *(Microsoft Suite)*** simultaneously to ensure the Veteran receive it to prevent the Veteran from saying they never received the appointment. Dedicated IT team member also instructs the Veteran on how to save the link in their calendar and how to add reminder notes.

No Response

- Provider uses **Teams *(Microsoft Suite)*** to instant message the MSA know to schedule the appointment if same day appointment.

No Response

Current Practices Identified

Activities Across All Timeframes *(Continued)*

2

Provider Accesses, Identifies, Packages & Sends Veteran Education Materials (i.e.: general, diagnosis, prognosis & Rx)

- Provider uses Whole Health **SharePoint *(Microsoft Suite)*** for any Veteran resources, materials or documents that the Veteran expressed interest in or would benefit from.

No Response

- Provider uses **Outlook** (blind email) or **WebEx** chat box to send **journals**, **VA & Non-VA YouTube videos**, and **PowerPoint *(Microsoft Suite)*** presentations. Educational videos are embedded in **PowerPoint *(Microsoft Suite)*** slides that are shared on **WebEx**, which include **VA & Non-VA YouTube videos**.

No Response

3

Interdisciplinary Coordination & Communication (Provider to Provider)

- Provider uses **Teams *(Microsoft Suite)*** or **Outlook *(Microsoft Suite)*** for consultation with other providers of the team.

No Response

- Provider uses **Teams *(Microsoft Suite)*** because it's functional for communication in-service, interdisciplinary and external to VA. Communication using this platform with other facilities for Interfacility Council is very functional.

No Response

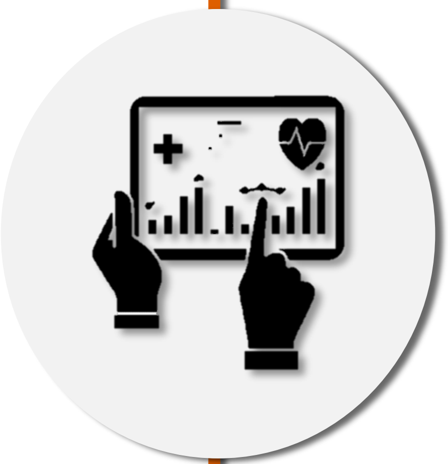

Education: Current Practices

For each task listed underneath the activity, choose the most appropriate practice from the drop-down: Current Practice, Best Practice, De-Implement, Undecided or No Response. Use the comment box for any notes.

Education

Current Practices Identified

Pre-Encounter Activities

1 Appointment Reminders

- Provider shares automated **appointment reminders *(MHV)*** with staff and Veterans. — No Response

2 Chart Review, Update Personal Information, Perform Assessment

- Provider uses **CPRS** to review the Veteran's records to find out if a wrist band is needed. — No Response
- Provider uses **CPRS** to check for the release of the order. — No Response
- Provider checks **Notes & Alerts; Labs & Tests; Imaging *(CPRS)*** as well as immunizations, and previously-documented Veteran education in **CPRS**. — No Response
- Provider uses **BCMA** or **VistA Imaging *(VistA)*** to look up any medications that are due. — No Response

3 Preparing for a Visit

- Provider informs Veterans on educational resources via **virtual Veteran orientation**. — No Response
- Provider uses **BCMA** for charting. — No Response
- Provider simultaneously opens **CPRS** and **I-Shaped** tools. When all of the Veteran information is entered in **I-Shaped**, it is transmitted to each nurse on the unit when it is printed out in the report. **I-Shaped** can also be used for the charge nurse as it has information about DNR's/fall risk/alcohol risk/seizure withdrawals. — No Response

Education

Current Practices Identified

Medical Encounter Activities

1

Medical Encounter Check-In

- Provider uses **BCMA** to administer medications, look up reports such as medication times and types of medications whether taken by mouth, IV infusions or continuous infusions. During check-in, Provider assess the Veteran's needs and what orders need to be addressed and how to go about the orders. No Response
- Provider uses **Get Well Network *(VistA)*** for Veteran check-in and to send mandatory videos (ie: how to order meals in-Veteran). No Response
- Staff ask Veterans to check-in using the **VetLink Kiosks**. No Response
- Staff reviews the **Care Assessment Need (CAN) Risk Assessment *(CPRS*)**. No Response

2

History Intake, Review Diagnostic Data, Perform Interview & Exam

- Nurses complete administrative duties via **CPRS**. Nurses can place necessary consults that haven't been ordered yet by either placing orders themselves or finding an appropriate physician to enter a consult. No Response

3

Review Veteran Generated Health Data (PGHD), Implement Protocols, Measure & Document Health Indices

- Provider uses **AMP** for the generation of reports for Veteran chart auditing. No Response

Current Practices Identified

Medical Encounter Activities *(Continued)*

4

Provider – Veteran Care Coordination & Communication

- Provider uses **Get Well Network *(VistA)*** to provide in-patient/Veteran education via in-room television.

No Response

5

Follow-up Appointment, Referrals, D/C Summaries

- Provider uses **Imed** consent as a witness if the doctor's do this.

No Response

Post-Encounter Activities

1

Check Records, Labs, Imaging & Procedures

- Provider checks **CPRS** to make sure a consult has been placed prior to the Veteran's discharge.

No Response

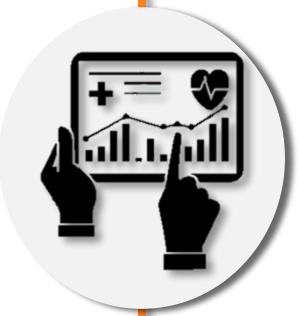

Education

Current Practices Identified

Ongoing Veteran Activities

1 Tracking Veteran Generated Health Data

- Veteran uses **Annie app for Veterans *(app)*** for reminders.

No Response

2 Veteran – Provider Communication (Seek Information about condition; Assess Need for Visit)

- Veteran uses **Secure Messaging *(MHV)*** in ***My HealtheVet*** to contact team and express concerns and/or needs.

No Response

Ongoing Provider Activities

1 Communicate & Consult with Healthcare Providers; Receive & Manage Referrals

- Provider uses **Teams *(Microsoft Suite)*** to contact staff and begin D/C planning such as ordering a BP cuff to send home with the Veteran.

No Response

- If on a weekend or a holiday, provider uses **SharePoint *(Microsoft Suite)*** to look for the doctor on call.

No Response

2 Identification & Use of Professional Resources

- Provider uses **DMC** to look up policies and procedures.

No Response

- Staff promotes the use of **VA mobile apps** and **FitBit/Apple Watch**.

No Response

Current Practices Identified

Activities Across All Timeframes

1 Schedule Medical Appointment

- Provider shares **MHV** with staff and Veterans for a list of appointments and preparing Veterans for prescreening. — No Response

2 Provider Accesses, Identifies, Packages & Sends Veteran Education Materials (i.e.: general, diagnosis, prognosis & Rx)

- Provider shares at-home exercises with the Veteran. — No Response
- Provider uses the **CARF Survey** to explore what's available for Veteran education. — No Response
- Provider creates topic-specific folders on **KRAMES**. — No Response
- Provider opens **CPRS** and searches the **VISN 8 Nucleus *(CPRS)*** database to find a medical animation of the Veteran's conduction and sends to the Veteran via **Secure Messaging *(MHV)*** in ***My HealtheVet.*** — No Response
- Provider searches for education or Rx resources using **KRAMES On Demand, Micromedex and/or Medline Plus.** — No Response
- Provider searches for medication administration times in **BCMA** to compare with how the Veteran take their medications at home and will make any changes as needed. — No Response

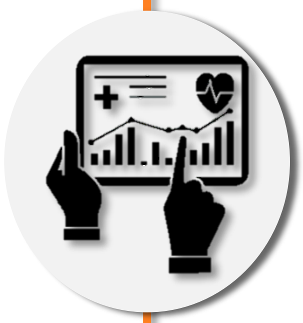

Education

Current Practices Identified

Activities Across All Timeframes *(Continued)*

3 Veteran Receives & Accesses Educational Resources

- Veteran accesses **VA-YouTube Videos** for education. — No Response
- Veteran accesses and uses **VA mobile apps**. — No Response
- Veteran accesses the medical animation of the condition that was delivered via **Secure Messaging *(MHV)*** in ***My HealtheVet.*** — No Response

4 Interdisciplinary Coordination & Communication (Provider to Provider)

- Provider uses **USA Mobility** to communicate with other providers. — No Response
- Provider uses **Teams *(Microsoft Suite)*** to contact other disciplines. — No Response
- Provider uses **Teams *(Microsoft Suite)*** and **Outlook *(Microsoft Suite)*** to share staff education. — No Response

5 Provider – Veteran Care Coordination & Communication

- Provider uses **telephone** for communication with the Veteran and caregiver or family and to follow up to make sure screening tests have been completed. . — No Response
- For in-Veteran, provider gives Veteran an **iPad** to virtually connect with the caregiver or family. — No Response
