# Supplementary material for: Super Users’ Reported Best Practices for Coordinating Proactive Integrated Use of Virtual Health Care Resources: Prospective Concurrent Mixed Methods Human-Centered Design Study
Source: J Med Internet Res. 2025 Nov 14;27:e81414. doi: 10.2196/81414 (PMC12663705; doi:10.2196/81414)
Supplement: Multimedia Appendix 5 [file jmir_v27i1e81414_app5.docx]

**Table S1.** Best practice summaries listed in consecutive order.

| **Summary 1** | When experiencing connectivity issues or other technical problems during **VVC^b^** appointment or session, provider or dedicated IT^c^ team member supplement their communication with the Veteran with a phone call **using Doximity** or **Cisco Jabber** to continue the session or troubleshoot the problem. |
| --- | --- |
| **Summary 2** | Healthcare staff can schedule follow up appointments by using **SM^d^ (MHV^e^), VVC Now, WebEx, VCM^f^ + MS^g^ Outlook, CPRS^h^** and **Telephone.** Healthcare staff can use **SM (MHV)** to answer any questions and schedule medical appointments. |
| **Summary 3** | Healthcare staff can use **MS Teams, MS Outlook, Telephone, VCM** for interdisciplinary communication and coordination regarding healthcare consultation, data collection planning, ordering equipment or to conduct team meetings and huddles. Specifically, IT members can use **VCM** to communicate or consult on IT issues with other providers. |
| **Summary 4** | Dedicated IT team member or qualified team member may receive a viewer alert from another team member from **CPRS + Notes and Alerts, SM, MHV, MS Teams** or **MS Outlook** about a Veteran having difficulty connecting or needs set up for a virtual appointment. |
| **Established Best Practice Summary 5^a^** | You can use a **Virtual Remote Stethoscope** with **VVC** and a headset to listen to a veterans heart and lungs. The veteran will send a link from **VVC** to allow the provider access and connect the device remotely to perform the activity. |
| **Summary 6** | While in a remote environment, you can use **VVC** to see veterans in their home environment and view the veteran’s refrigerator to incorporate Whole Health components (ie, nutrition) during annual evaluations. With veteran consent, non-veterans such as caregivers can participate in the appointment*.* |
| **Summary 7** | Providers can use **VVC, VCM, MS Outlook**, and **Virtual Tool RX^i^ app** to conduct virtual assessment, provide treatment, and make recommendations when taking history, performing an exam, or across all timeframes on the healthcare continuum. For example, a provider can send a veteran exercise to perform at home using **MS Outlook** or **VCM** at any time across the healthcare continuum. |
| **Summary 8** | You can use the **Live Whole Health app** or **SM (MHV)** to send a **Personal Health Inventory Questionnaire** to a veteran. **MS Outlook** (do not reply) can be used in lieu of **SM (MHV)** if veteran doesn’t have an account. |
| **Summary 9** | You can promote the use of **VA mobile apps** and **Fitbit** or **Apple watch. Fitbit** can be used to track health, sleep, steps, and pulse and to track veteran heart rate monitoring in situations like when they are experiencing anxiety. |
| **Summary 10** | You can use **CPRS, Telephone, MS Teams, VVC** among clinical team members, including other disciplines, to share information and discuss veteran care during the session, to prepare for follow-ups and data collection, or to alert providers to join a session. Healthcare staff can use these VHRs^j^ to manage communication and notes about medications, appointments, or supplies. **CPRS** can be used to track notes throughout the care continuum phases. |
| **Summary 11** | When a veteran enters vitals (eg, blood pressure, heart rate) to **Annie App for Veterans,** these data and other activities are generated into a dashboard in which you can view in the **Annie app for Clinicians.** You can also use the **Annie app for Clinicians** to track veteran-specific health data like bladder and bowel care. |
| **Summary 12** | **VCM** can be used to schedule a medical appointment with any veteran, even if at a different clinic through the anywhere-to-anywhere directive. You can use **WebEx** or **VVC** to conduct a remote appointment with a veteran to address weight, medical history, and presenting complaints. While using **VVC**, you can also access **CPRS** to review veteran history, which can be kept open during the encounter with the veteran. |
| **Established Best Practice Summary 13^a^** | Staff can use **MS Teams** to conduct a huddle if a veteran needs a follow-up. **MS Teams** can also be used to coordinate an annual visit. |
| **Summary 14** | Provider follows up with veteran via **SM (MHV), MS Outlook**, or **Telephone** to get updates from the veteran, assess progress after treatment plan, confirm device was received, and share education material. Communication with the Veteran can be done via **SM (MHV)** or **MS Outlook.** |
| **Summary 15** | **Direct texting** via an appropriate texting platform, or **SM (MHV)** provides asynchronous communication for non-verbal patients. These are appropriate forms of communication as an alternative to verbal communication. |
| **Summary 16** | You can use **VA and Non-VA YouTube Videos, VA and Non-VA apps**, and other applicable educational resources to provide training to prepare for a virtual visit and use the technology required. You can even provide a live demonstration with a veteran. |
| **Summary 17** | Dedicated IT member prepares contact information through **CPRS** (from face sheet) and other forms or features needed for the virtual communication with the veteran (such as **ROES^k^**) before communicating with the veteran. |
| **Summary 18** | Veteran shares treatment and post operation updates, or express concerns or any matter related to care via **SM (MHV)** or **Telephone** to obtain provider’s feedback. Test results can be accessed with **MHV**; and images can be shared in **My VA Images** to facilitate these communications. |
| **Summary 19** | Provider can use apps such as **CBT-i^l^-Coach app, PTSD^m^ Coach app, CPT^n^ Coach app** and **Insomnia Coach app** to use during post-appointment for providing treatment and to manage and track PGHD^o^. |
| **Summary 20** | Veteran-based VHR such as **Annie app for Veterans**, can also be used for the Veteran to receive reminders or educational material. |
| **Summary 21** | Dedicated IT team member or qualified staff member can use **CPRS + ROES** for **iPad** orders for Veterans. The order can be tracked and when delivered, a follow-up appoint can be set up to instruct Veteran on how to set up and use the device. |
| **Summary 22** | There are several ways healthcare staff can prepare for an upcoming virtual appointment. 1) Healthcare staff can use **MS Outlook** to block off time to ensure the healthcare staff is not double booked and save time by using **VCM** to see all virtual appointments in one location to provide reminder notes to the healthcare staff about an upcoming veteran appointment. 2) Healthcare staff can use **MS Teams** to coordinate an [interdisciplinary] annual visit during a team huddle. |
| **Summary 23** | You can use **SM (MHV)**, **MHV**, **VVC**, **VCM** to manage appointment reminders between staff and veterans. |
| **Summary 24** | You can use **WebEx** to remind veterans 5 minutes before the appointment is scheduled to occur. |
| **Summary 25** | Staff use several ways to send appointment links to the veteran and healthcare staff at different time periods. Staff can use **VCM** to send an appointment link while on the telephone with the veteran to have a link sent directly to their email. Alternatively, staff can use a **URL^p^ generator** to send a link via **VVC** for veterans who are unable to navigate their email or locate the serial number [of VA issued device] in **CPRS + ROES** to create a static link in the **URL generator** to directly connect to the veteran’s VA issued device **[iPad**]. Healthcare staff can also use **MS Teams** to set up links for interdisciplinary appointments to coordinate the next provider in the queue to connect to the **VVC** link for the veteran appointment. |
| **Summary 26** | Dedicated healthcare staff can prepare for an upcoming virtual appointment utilizing dedicated team member support with the use of digital VA issued equipment by using **CPRS** to look up veteran’s next appointment, then goes to **CPRS + Notes and Alerts** to scan previous notes related to issues with technology, find out what platform the veteran used in the past and what type of equipment the veteran was previously issued. A dedicated healthcare staff member can use **CPRS + ROES** to track ordered technology to prepare for follow up appointment to assess and set up of the device by contacting the veteran via telephone to ensure they have proper equipment and performs a test run to prepare for their upcoming appointment. |
| **Summary 27** | You can open **CPRS + ROES** to prepare for a call while simultaneously looking at a face sheet for the veteran’s contact information such as telephone number and email address. |
| **Summary 28** | Healthcare staff can prepare for an upcoming virtual appointment with the delivery of educational materials using a dedicated healthcare staff to provide training materials to the veterans on how to use the virtual platform via **VA and Non-VA YouTube Videos, VA and Non-VA apps.** Healthcare staff can use the **Telephone** to instruct the veteran on how to download and enter data in the **CBTi-Coach app.** Healthcare staff can prepare for an upcoming virtual appointment utilizing dedicated team member support by using **Telephone, Telephone + Doximity,** or **VVC** to contact a veteran to ensure the technology is working properly and provide troubleshooting for issues. Healthcare staff can use **WebEx** for Android users when they can’t connect with **VCM.** Before the start of the virtual session, healthcare staff can use **WebEx** or **VVC** to review an informed consent, explain how the visit will be conducted virtually and review features and functionalities of the platform. |
| **Summary 29** | **MS Teams** can be used to assist with troubleshooting technology issues. |
| **Summary 30** | Staff and providers encourage veterans to use **SM (MHV)** to communicate and to use **VA Mobile apps** and tracking devices such as **Fitbit** or **Apple Watch** for tracking communication and health data. |
| **Summary 31** | Provider in the inpatient setting gives the veteran the necessary device such as **iPad** or **tablet** to communicate with family. |
| **Summary 32** | Healthcare staff can monitor veteran check-in when in person or virtually for their scheduled appointment. Veterans can check-in using stand-alone self-service kiosks when in person. Healthcare staff can use **VCM** to locate the links for a virtual check-in of a veteran. |
| **Summary 33** | Healthcare staff can use **MS Teams** to alert clerk that veteran has arrived for his or her appointment and can use the instant message feature to ask a clerk to update the veteran’s records if necessary. |
| **Summary 34** | You can use **VVC** to lock a therapy session with veterans so there are no unnecessary intrusions from others. Healthcare staff can also use **VVC** to perform 3-way calling and invite family members into sessions with veterans. |
| **Established Best Practice Summary 35^a^** | **VVC** is the preferred platform to conduct group appointments, where the provider can promote healthy lifestyle and encourage the use of **VVC.** |
| **Summary 36** | During the history and examination phase of treatment, staff can use **CPRS + ROES** to consult for device issuance. |
| **Summary 37** | Providers can monitor health indices and identify abnormalities by reviewing the **Care Assessment Need (CAN^q^) Risk Assessment (CPRS)** and other health data uploaded into the patients’ charts through the telehealth program. Additional health indices can be monitored when the patient shares PGHD during a healthcare visit that they collected or documented on various apps or devices, but it is important to promote the use of select VA mobile apps or preferred devices with the veteran. |
| **Summary 38** | When assessing the health of a veteran, a significant amount of information needed is obtained by the veteran. When conducting the appointment virtually, the veteran can share their PGHD using a variety of VA mobile apps, such as **Annie app for Veterans.** |
| **Summary 39** | You can use the **3D camera** to assist with tracking wound care. |
| **Summary 40** | **Qualtrics** will send an autogenerated email to a healthcare team member if a veteran endorses suicidal ideation. Collaborations with Central Office indicate that **Qualtrics** and **REDCap** can be used for surveys between staff and veterans externally outside VA firewall at home, as it is approved for storage of PHI^r^ and PII^s^ and has public-facing survey capability. |
| **Summary 41** | You can use the **CAN Risk Assessment (CPRS)** to assess who has an elevated CAN score to group Veterans who fulfill those criteria. |
| **Summary 42** | Monitored health information can be gathered and acquired of variety of ways. PGHD can be obtained asynchronously through equipment such as a wearable health tracking device **(Fitbit, Apple Watch), Pulse Oximeter, Glucometer, Digital Scale, or Blood Pressure Monitor.** Veterans can independently input monitored health indices using apps such as **CBT-i Coach app, Annie app for Veterans, and Cardiac Monitoring Devices (ie, Alivecor app, Zio patch).** For synchronous collection of monitored health data during an appointment, additional VHR, such as the **3D Camera** used for monitoring wounds, can be invaluable. |
| **Summary 43** | The veteran can request refill of medications 3 different ways: Requests for more refills can be sent to the provider using **SM (MHV)** or – if there are already refills available, they can order them through **VA Prescriptions Refill (MHV)** or use the **RX Refill app.** |
| **Summary 44** | Provider can obtain educational materials appropriate for the patient veteran through: CPRS linked resources such as **Krames, VISN^t^ 8 Nucleus** and other systems such as **SM​ (MHV), MHV, VA YouTube channels,** and **VISTA^u^-Get Well Network for (inpatients).** Material might include written material, videos, animation, and images. |
| **Summary 45** | Provider organizes selected educational material by topics or relevance in folders using **Krames.** |
| **Summary 46** | Provider uses **SM (MHV), Krames, Veterans Health Library** to refer or provide education materials to veteran. For example, provider can deliver a medical animation regarding a veteran’s medical condition for educational purposes. Providers can also use **Annie App** to deliver educational material. |
| **Summary 47** | Staff can use **MS SharePoint, MS Teams,** and **MS Outlook** to deliver education materials to internal team members. For example, **MS Teams** and **MS Outlook** are used to share educational information with staff, while **MS SharePoint** can be used to share service updates. |
| **Summary 48** | Provider uses **Annie app, SM (MHV), VVC, WebEx, MS Outlook** (blind email or encrypted), and **Direct Texting** to send education materials to Veterans. Provider uses **Get Well Network (VISTA)** to provide inpatient veteran education via in-room television. |
| **Summary 49** | You can use the **CBT-i Coach app** and the data from **Apple Watch** or **Fitbit** to review and track weekly sleep cycles and can adjust the schedule when needed. You can utilize information from the service connection disability rating and vitals generated in both **CPRS** and **CBT-i Coach** to ensure veterans are improving over the course of time together with the provider. |
| **Summary 50** | Through the telehealth program, you can monitor health indices such as sugar levels and can use a **Blood Pressure Monitor** to measure and automatically populate vital signs directly into **CPRS** to avoid errors. Telehealth vitals can be compiled into a note that tracks progress over time, which is saved on **CPRS.** You can also review the **Care Assessment Need (CAN) Risk Assessment** in **CPRS.** |
| **Summary 51** | You can collect veteran-generated health data through **Apple watch** or **Fitbit, Alivecor app,** or **Pulse Oximeter machine** for vitals and cardiac information. All can be and some are currently integrated to automatically upload data to **SMHD^v^ app** for providers to view. |
| **Established Best Practice Summary 52^a^** | Consults for PGHD device issuance are entered through **CPRS + ROES.** A significant amount of PGHD can be collected using **VA apps,** which can be prescribed using the **VA Virtual Toolkit Prescription Pad.** Consults for device issuance of items addressing the digital divide, such as **iPads,** are entered through the **Consults Tool (CPRS).** Additional PGHD equipment, such as a wearable tracking device **(ie, Fitbit)** can be ordered through **LEAF^w^.** |
| **Established Best Practice Summary 53^a^** | Healthcare staff can use **MS Excel** to receive and manage referrals, for example, with veterans who are interested in coming to WH Immersion. |
| **Established Best Practice Summary 54^a^** | A digital divide consult can be placed to order and issue VA-loaned devices. Any VA staff member who intends to manage the veteran’s care via **VVC** (or their designee) can place the digital divide consult. The consult should be placed for veterans who would benefit from **VVC** into their home but lack an affordable or quality internet connection or a video-capable device. The veteran must also meet one or more of the clinical criteria listed on the consult. If the provider determines the veteran’s care will be enhanced by using peripheral devices, these are ordered or identified via the consult. Peripherals include blood pressure monitors, weight scales, stethoscopes, pulse oximeters, and thermometers. The social worker places the video device order consult after determining the veteran has qualified for a VA loaned device. The consult alerts the local ROES coordinator of the devices (eg, tablet, peripherals) that the veteran requires to place the order in **ROES.** |
| **Established Best Practice Summary 55^a^** | Collaborations with Central Office indicate that **Qualtrics** and **REDCap** can be used for surveys between staff and veterans externally outside VA firewall at home, as it is approved for storage of PHI and PII and has public-facing survey capability. |
| **Established Best Practice Summary 56^a^** | Provider uses **CPRS** to put in orders such as labs, consults, and procedures. |
| **Established Best Practice Summary 57^a^** | Literature by Haun et al, published best practices in **SM (MHV)** for delivering labs, tests, and imaging **(My VA Images)** results. |

^a^Not included in final analysis due to lack of saturation for the threshold of targeted use. ^b^VVC: VA Video Connect, ^c^IT: Information Technology, ^d^SM: secure messaging, ^e^MHV: My HealtheVet, ^f^VCM: Virtual Care Manager, ^g^MS: Microsoft, ^h^CPRS: Computerized Patient Record System, ^i^RX: Prescription, ^j^VHRs: Virtual Health care Resources, ^k^ROES: Remote Order Entry System, ^l^CBT-i: Cognitive Behavioral Therapy-Insomnia, ^m^PTSD: Post-Traumatic Stress Disorder, ^n^CPT: Cognitive Processing Therapy, ^o^PGHD^:^Patient-Generated Health Data, ^p^URL: Uniform Resource Locator, ^q^CAN: Care Assessment Need, ^r^PHI: Protected Health Information, ^s^PII: Personally Identifiable Information, ^t^VISN: Veterans Integrated Service Network, ^u^VISTA: Veterans Health Information System Technology Architecture, ^v^SMHD: Share My Health Data, ^w^LEAF: Light Electronic Action Framework.
