# Supplementary material for: Super Users’ Reported Best Practices for Coordinating Proactive Integrated Use of Virtual Health Care Resources: Prospective Concurrent Mixed Methods Human-Centered Design Study
Source: J Med Internet Res. 2025 Nov 14;27:e81414. doi: 10.2196/81414 (PMC12663705; doi:10.2196/81414)
Supplement: Multimedia Appendix 7 [file jmir_v27i1e81414_app7.docx]

**Table S1.** Example: Patient-Generated Health Data (PGHD) management summaries on use of virtual health care resources (VHRs) to complete four identified common tasks.

| **8.1 Consult for PGHD^a^ Device Issuance** | **Summary 36:** During the history and examination phase of treatment, staff can use **CPRS^b^ + ROES^c^** to consult for device issuance. |
| --- | --- |
|  | **Established Best Practice Summary 52^d^:** Consults for PGHD device issuance are entered through the **CPRS + ROES.** A significant amount of PGHD can be collected using **VA apps,** which can be prescribed using the **VA Virtual Toolkit Prescription Pad.** Consults for device issuance of items addressing the digital divide, such as **iPads,** are entered through the **Consults Tool (CPRS).** Additional PGHD equipment, such as a wearable tracking device **(ie, Fitbit),** can be ordered through **LEAF^e^.** |
| **8.2 Track PGHD, Record Vitals and Monitor Health Indices** | **Summary 9:** You can promote the use of **VA mobile apps** and **Fitbit** or **Apple Watch. Fitbit** can be used to track health, sleep, steps, and pulse, and to track veteran heart rate monitoring in situations like when they are experiencing anxiety. |
|  | **Summary 11:** When a veteran enters vitals (eg, blood pressure, heart rate) to **Annie App for Veterans,** these data and other activities are generated into a dashboard in which you can view in the **Annie app for Clinicians.** You can also use the **Annie app for Clinicians** to track veteran-specific health data like bladder and bowel care. |
|  | **Summary 19:** Provider can use apps such as **CBT-i^f^ Coach app, PTSD^g^ Coach app, CPT^h^ Coach app,** and **Insomnia Coach app** to use during post-appointment for providing treatment and to manage and track PGHD. |
|  | **Summary 30:** Staff and providers encourage veterans to use **SM^i^ (MHV^j^)** to communicate and to use **VA Mobile apps** and tracking devices such as **Fitbit** or **Apple Watch** for tracking communication and health data. |
|  | **Summary 37:** Providers can monitor health indices and identify abnormalities by reviewing the **CAN^k^ Risk Assessment (CPRS)** and other health data uploaded into the patients’ charts through the telehealth program. Additional health indices can be monitored when the patient shares PGHD during a healthcare visit that they collected or documented on various apps or devices, but it is important to promote the use of select VA mobile apps or preferred devices with the veteran. |
|  | **Summary 39:** You can use the **3D camera** to assist with tracking wound care. |
|  | **Summary** **42:** Monitored health information can be gathered and acquired in a variety of ways. PGHD can be obtained asynchronously through equipment such as a wearable health tracking device **(Fitbit, Apple Watch), Pulse Oximeter, Glucometer, Digital Scale, or Blood Pressure Monitor.** Veterans can independently input monitored health indices using apps such as **the CBT-i Coach app, Annie app for Veterans, and Cardiac Monitoring Devices (ie, Alivecor app, Zio patch).** For synchronous collection of monitored health data during an appointment, additional VHR^l^, such as the **3D Camera** used for monitoring wounds, can be invaluable. |
|  | **Summary 49:** You can use the **CBT-i Coach app** and the data from **Apple Watch** or **Fitbit** to review and track weekly sleep cycles and can adjust the schedule when needed. You can utilize information from the service connection disability rating and vitals generated in both **CPRS** and **CBT-i** Coach to ensure veterans are improving over the course of time together with the provider. |
|  | **Summary 50:** Through the telehealth program, you can monitor health indices such as sugar levels and use a **Blood Pressure Monitor** to measure and automatically populate vital signs directly into **CPRS** to avoid errors. Telehealth vitals can be compiled into a note that tracks progress over time, which is saved on **CPRS.** You can also review the **CAN Risk Assessment** in **CPRS.** |
|  | **Summary 51:** You can collect veteran-generated health data through **Apple Watch** or **Fitbit, Alivecor app,** or **Pulse Oximeter Machine** for vitals and cardiac information. All can be, and some are currently integrated to automatically upload data to **the SMHD^m^ app** for providers to view. |
| **8.3 Measure PGHD, Vitals, and Health Indices** | **Summary 11:** When a Veteran enters vitals (blood pressure, heart rate) to **Annie App for Veterans,** these data and other activities are generated into a dashboard, which you can view in the **Annie app for Clinicians.** You can also use the **Annie app for Clinicians** to track Veteran-specific health data like bladder and bowel care. |
|  | **Summary 42:** Monitored health information can be gathered and acquired in a variety of ways. PGHD can be obtained asynchronously through equipment such as a wearable health tracking device **(Fitbit, Apple Watch), Pulse Oximeter, Glucometer, Digital Scale, or Blood Pressure Monitor.** Veterans can independently input monitored health indices using apps such as **the CBT-i Coach app, Annie app for Veterans, and Cardiac Monitoring Devices (ie, Alivecor app, Zio patch).** For synchronous collection of monitored health data during an appointment, additional VHR, such as the **3D Camera** used for monitoring wounds, can be invaluable. |
|  | **Summary 49:** You can use the **CBT-i Coach app** and the data from **Apple Watch** or **Fitbit** to review and track weekly sleep cycles and can adjust the schedule when needed. You can utilize information from the service connection disability rating and vitals generated in both **CPRS** and **CBT-i** Coach to ensure veterans are improving over the course of time together with the provider. |
|  | **Summary 50:** Through the telehealth program, you can monitor health indices such as sugar levels and use a **Blood Pressure Monitor** to measure and automatically populate vital signs directly into **CPRS** to avoid errors. Telehealth vitals can be compiled into a note that tracks progress over time, which is saved on **CPRS**. You can also review the **CAN Risk Assessment** in **CPRS.** |
| **8.4 Veteran-Based VHR** | **Summary 11:** When a veteran enters vitals (blood pressure, heart rate) to **Annie App for Veterans,** these data and other activities are generated into a dashboard that you can view in the **Annie app for Clinicians.** You can also use the **Annie app for Clinicians** to track veteran-specific health data like bladder and bowel care. |
|  | **Summary 19:** Provider can use apps such as the **CBT-i Coach app, PTSD Coach app, CPT Coach app,** and **Insomnia Coach app** to use during post-appointment for providing treatment and manage and track PGHD. |
|  | **Summary 30:** Staff and providers encourage veterans to use **SM (MHV)** to communicate and to use **VA Mobile apps** and tracking devices such as **Fitbit** or **Apple Watch** for tracking communication and health data. |
|  | **Summary 38:** When assessing the health of a veteran, a significant amount of information needed is obtained from the veteran. When conducting the appointment virtually, the veteran can share their PGHD using a variety of **VA mobile apps**, such as **the Annie app for Veterans.** |
|  | **Summary 42:** Monitored health information can be gathered and acquired in a variety of ways. PGHD can be obtained asynchronously through equipment such as a wearable health tracking device **(Fitbit, Apple Watch), Pulse Oximeter, Glucometer, Digital Scale, or Blood Pressure Monitor.** Veterans can independently input monitored health indices using apps such as **the CBT-i Coach app, Annie app for Veterans, and Cardiac Monitoring Devices (ie, Alivecor app, Zio patch).** For synchronous collection of monitored health data during an appointment, additional VHR, such as the **3D Camera** used for monitoring wounds, can be invaluable*.* |
|  | **Summary 43:** The veteran can request a refill of medications in 3 different ways: Requests for more refills can be sent to the provider using **SM (MHV)**; if there are already refills available, they can order them through **VA Prescriptions Refill (MHV)**; or use the **RX^n^ Refill app.** |
|  | **Summary 49:** You can use the **CBT-i Coach app** and the data from **Apple Watch or Fitbit** to review and track weekly sleep cycles and can adjust the schedule when needed. You can utilize information from the service connection disability rating and vitals generated in both **CPRS** and **CBT-i** Coach to ensure veterans are improving over the course of time together with the provider. |

^a^PGHD: Patient-Generated Health Data, ^b^CPRS: computerized patient reporting system, ^c^ROES: Remote Order Entry System, ^d^Indicates summary is an established best practice and has gone through cultural transformation, ^e^LEAF: Light Electronic Action Framework, ^f^CBT-i: Cognitive Behavioral Therapy-Insomnia, ^g^PTSD: Post Traumatic Stress Disorder, ^h^CPT: Cognitive Processing Therapy, ^i^SM: secure messaging, ^j^MHV: My HealtheVet, ^k^CAN: Care Assessment Need, ^l^VHR: Virtual Health care Resource, ^m^SMHD: Share My Health Data, ^n^RX: Perscription.
