# Supplementary material for: Super Users’ Reported Best Practices for Coordinating Proactive Integrated Use of Virtual Health Care Resources: Prospective Concurrent Mixed Methods Human-Centered Design Study
Source: J Med Internet Res. 2025 Nov 14;27:e81414. doi: 10.2196/81414 (PMC12663705; doi:10.2196/81414)
Supplement: Multimedia Appendix 9 [file jmir_v27i1e81414_app9.docx]

| **Task** | **Summary and Service Line with Virtual Healthcare Resources Bolded** | **Best Practice Resource Links** | **Educational Resource Links** |
| --- | --- | --- | --- |
| **1.1 Scheduling Medical Appointment** | **Summary #12^1,3,5^: VCM** can be used to schedule a medical appointment with any Veteran, even if at a different clinic through the anywhere-to-anywhere directive. You can use **WebEx** or **VVC** to conduct a remote appointment with a Veteran to address weight, medical history, and presenting complaints. While using **VVC**, you can also access **CPRS** to review Veteran history, which can be kept open during the encounter with the Veteran. | - <https://telehealth.va.gov/> - [Scheduling -Telehealth – Scheduling - Telehealth Community (blackboard.com)](https://vaots.blackboard.com/webapps/blackboard/execute/modulepage/view?course_id=_2466_1&cmp_tab_id=_3176_1&mode=view) | **WebEx:**   - [Approved Video Technologies \| Following the Pandemic \| VHA Telehealth Services Intranet (va.gov)](https://vaww.telehealth.va.gov/technology/alt/post-pandemic.asp)   **VVC:**   - [VA Video Connect (VVC) – VA Video Connect (VVC... (blackboard.com)](https://vaots.blackboard.com/webapps/blackboard/execute/modulepage/view?course_id=_2591_1&cmp_tab_id=_3396_1&mode=view) - [https://vaots.blackboard.com/bbcswebdav/library/LibraryContent/VA Video Connect/VVC Instructional Videos/Provider-to-Scheduler Handoff in VVC video/Provider to Scheduler Handoff Video.html](https://vaots.blackboard.com/bbcswebdav/library/LibraryContent/VA%20Video%20Connect/VVC%20Instructional%20Videos/Provider-to-Scheduler%20Handoff%20in%20VVC%20video/Provider%20to%20Scheduler%20Handoff%20Video.html) - [VA Video Connect \| VA Mobile](https://mobile.va.gov/app/va-video-connect)   **VCM:**   - [Virtual Care Manager – Virtual Care Manager Community (blackboard.com)](https://vaots.blackboard.com/webapps/blackboard/execute/modulepage/view?course_id=_2540_1&cmp_tab_id=_3308_1&mode=view) - [Virtual Care Manager User Guide VCM 2.0 (va.gov)](https://mobile.va.gov/sites/default/files/user-manual-vcm.pdf)   **CPRS:**   - [CPRS Technical Manual (va.gov)](https://www.va.gov/vdl/documents/Clinical/Comp_Patient_Recrd_Sys_(CPRS)/cprslmtm.pdf) - [CPRS Session 1 Getting Started](https://www.youtube.com/watch?v=y52UtvS95F0) |
| **1.2 Sending Appointment Reminder to Veteran** | **Summary #20^2^:** Veteran-based VHR such as **Annie app for Veterans**, can also be used for the Veteran to receive reminders or educational material. | - [Annie App – Annie App Community (blackboard.com)](https://vaots.blackboard.com/webapps/blackboard/execute/modulepage/view?course_id=_2548_1&cmp_tab_id=_3324_1&mode=view) | **Annie App for Veterans:**   - [Annie App for Veterans \| VA Mobile](https://mobile.va.gov/app/annie-app-veterans)   **Annie App for Clinicians**:   - [Annie App for Clinicians \| VA Mobile](https://mobile.va.gov/app/annie-app-clinicians) |
|  | **Summary #23^2,3,5^:** You can use **SM (MHV)**, **MHV**, **VVC**, **VCM** to manage appointment reminders between staff and Veterans. | - [Scheduling -Telehealth – Scheduling - Telehealth Community (blackboard.com)](https://vaots.blackboard.com/webapps/blackboard/execute/modulepage/view?course_id=_2466_1&cmp_tab_id=_3176_1&mode=view) | **VCM:**   - [Virtual Care Manager – Virtual Care Manager Community (blackboard.com)](https://vaots.blackboard.com/webapps/blackboard/execute/modulepage/view?course_id=_2540_1&cmp_tab_id=_3308_1&mode=view) - [Virtual Care Manager User Guide VCM 2.0 (va.gov)](https://mobile.va.gov/sites/default/files/user-manual-vcm.pdf) - <https://vaots.blackboard.com/webapps/blackboard/execute/courseMain?course_id=_2693_1>   **MHV:**   - <https://www.myhealth.va.gov/mhv-portal-web/web/myhealthevet/keeping-up-with-your-va-appointments> - <https://www.myhealth.va.gov/mhv-portal-web/home>   **SM (MHV):**   - [My HealtheVet Resources – My HealtheVet Resources](https://vaots.blackboard.com/webapps/blackboard/execute/modulepage/view?course_id=_3174_1&cmp_tab_id=_4591_1&mode=view) - [Secure Messaging Through My HealtheVet - My HealtheVet Product (va.gov)](https://vaww.va.gov/MYHEALTHEVET/Secure_Messaging.asp)   **VVC:**   - [Comprehensive Scheduling Guide](https://learn-us-gov-west-1-prod-fleet01-xythos.s3.us-gov-west-1.amazonaws.com/612eabb1bae6a/926548?response-cache-control=private%2C%20max-age%3D10800&response-content-disposition=inline%3B%20filename%2A%3DUTF-8%27%27Telehealth%2520Comprehensive%2520Scheduling%2520Guide.pdf&response-content-type=application%2Fpdf&X-Amz-Security-Token=FwoDYXdzEFwaDFdWBwu8Qnljj33raSLlBEG79G6uSgqS1gV7XumFLL29mVzfZR2ItKpdNNIpazlzKSA7vQ8KQ1cKwss0xhZ695qcPNvlnF5nnE7uf2LJrvuU1bEy%2FhBw1gDgKC1M3H2cKleQDi8qdOuWHuuT%2Bz%2B1vBciloAvE%2B9njPATZmayJYXPPUOsUBuW%2FyBHutOojxVAqQfHQvr5Glk5E4YLC44TC5M72YUiHQVMQNGszErhiq67CXl4R3%2BCkXKwxw1jGAB9xRen3ciPWVeGqlJafjnlztzO2kHUeJwIXcAyUEOWsdnEMqHnGww9Y%2FXG38FU6Lm51Sobk7dK%2BC84HY6NtFOdaZdBO5ybDv4pIAFIO6C1h%2F3gXLEbvwDlDc3GfAyaenOsXPTyWqnLEjqNL4FG7MVEOqRLpLCjjI2kzl7T4qRa704S0SvxfiwB2ipFj53eO7YpYPNIf%2FDPujx%2B3GAbv8eyP9QILqsuuMfBMBsA7On0fXXLIeVnSNKQOci447WoTR6sJ1%2BL6oqlMFN5GFiyaWBtxlVW5Ua0ZqleRApO6Yyy6QToLJe%2FT4O%2FxXzjiewo2bSxR8GVjlXFzj9gR8NL50z6D4Qv3bjbwiHpA%2Feit%2B%2FSOZmx%2BD7ibJg50bfu2Crn5861OzgWX1ZOnMwGsrJPxcAm%2BQQjx4DcvdWAZgFf%2Fd8mcf6zVAj2Htdqgajw5q2ID5INY6y7nBFZ1ZMgE0mgCLwIjgAbyfzy7iAC13MFYnO5gQPVGU63XBm8C6W2XDe7%2BcxXk8PjfRzNQTomG64178nUOF73OmyHIw2ylJADjhuFXgLLJxLnH0%2FZAxG2qlCjzgi7n4jr1k8oifGSvQYyQYSvtarPuTGyldoMxXoqU9pFz8jOeDmGGSItueJhk3bh752TCo1NRA8NtJKtzMop%2FtL1U7GwxcvdPvgd5baWPzP%2F&X-Amz-Algorithm=AWS4-HMAC-SHA256&X-Amz-Date=20250206T133000Z&X-Amz-SignedHeaders=host&X-Amz-Expires=10800&X-Amz-Credential=ASIAUMDY6U44YUBHHZ4X%2F20250206%2Fus-gov-west-1%2Fs3%2Faws4_request&X-Amz-Signature=61adc7da9732853a6f4cd38437847c17e2758de282d83589c0d0f86f9e74ad0c) |
|  | **Summary #24^5^**: You can use **WebEx** to remind Veterans 5 minutes before the appointment is scheduled to occur. | - [Scheduling -Telehealth – Scheduling - Telehealth Community (blackboard.com)](https://vaots.blackboard.com/webapps/blackboard/execute/modulepage/view?course_id=_2466_1&cmp_tab_id=_3176_1&mode=view) | **WebEx:**   - [Approved Video Technologies \| Following the Pandemic \| VHA Telehealth Services Intranet (va.gov)](https://vaww.telehealth.va.gov/technology/alt/post-pandemic.asp) - <https://help.webex.com/en-us/article/ruxycv/Sending-Invitations,-Reminders,-and-Registration-Notifications-in-Cisco-Webex-Training> |
|  | **Summary #25^3,4,5^:** Staff use several ways to send appointment links to the Veteran and healthcare staff at different time periods. Staff can use **VCM** to send an appointment link while on the telephone with the Veteran to have a link sent directly to their email. Alternatively, staff can use a **URL generator** to send a link via **VVC** for Veterans who are unable to navigate their email or locate the serial number [of VA issued device] in **CPRS + ROES** to create a static link in the **URL generator** to directly connect to the Veteran's VA issued device **[iPad**]. Healthcare staff can also use **MS Teams** to set up links for interdisciplinary appointments to coordinate the next provider in the queue to connect to the **VVC** link for the Veteran appointment. | - [Scheduling -Telehealth – Scheduling - Telehealth Community (blackboard.com)](https://vaots.blackboard.com/webapps/blackboard/execute/modulepage/view?course_id=_2466_1&cmp_tab_id=_3176_1&mode=view) - [VVC Flip Book (blackboard.com)](https://vaots.blackboard.com/bbcswebdav/orgs/vaots_th_sched/thquickflipresource/index.html) - [Microsoft Office Fact Sheet_8-2020 Final__Correction_508_9-2021_corrected_links11172021.pdf (sharepoint.com)](https://dvagov.sharepoint.com/sites/vacovetsprivacy/vhapo/Documents/Guidebooks,%20Fact%20Sheets%20and%20Practice%20Briefs/Microsoft%20Office%20Fact%20Sheet_8-2020%20Final__Correction_508_9-2021_corrected_links11172021.pdf) - [Remote Patient Monitoring-Home Telehealth – Remote ... (blackboard.com)](https://vaots.blackboard.com/webapps/blackboard/execute/modulepage/view?course_id=_2686_1&cmp_tab_id=_3628_1&mode=view) - [Remote Patient Monitoring - Home Telehealth Support Staff Community (blackboard.com)](https://vaots.blackboard.com/webapps/blackboard/execute/modulepage/view?course_id=_2501_1&cmp_tab_id=_3225_1&mode=view) | **VCM:**   - <https://mobile.va.gov/sites/default/files/user-manual-vcm.pdf> - <https://vaots.blackboard.com/bbcswebdav/xid-512168_1>  **VVC:** - [VA Video Connect (VVC) – VA Video Connect (VVC... (blackboard.com)](https://vaots.blackboard.com/webapps/blackboard/execute/modulepage/view?course_id=_2591_1&cmp_tab_id=_3396_1&mode=view) - <https://vaots.blackboard.com/bbcswebdav/library/LibraryContent/VA%20Video%20Connect/VA%20Video%20Connect%20Web%202.X%20Provider%20Guide.pdf> - [VA Video Connect \| VA Mobile](https://mobile.va.gov/app/va-video-connect)   **CPRS + ROES:**   - [Remote Order Entry System (ROES) - VA Authentication Federation Infrastructure (VAAFI)](https://www.va.gov/EAUTH/ROES/index.asp) - <https://www.va.gov/vdl/documents/Clinical/Remote_Order_Entry_Sys_(ROES)/rmpf3_0um.pdf?msclkid=2eb7cc0bd15211ec98d209e073e362de>   **URL Generator:**   - [URLs - VA.gov Design System](https://design.va.gov/content-style-guide/url-standards) - [vvc-url.xlsm (sharepoint.com)](https://dvagov.sharepoint.com/:x:/r/sites/VHA-Telehealth/_layouts/15/Doc.aspx?sourcedoc=%7BB74F5C10-C485-4FE2-A0FF-CEF98721F9A9%7D&file=vvc-url.xlsm&action=default&mobileredirect=true) - [Links - VA.gov Design System](https://design.va.gov/content-style-guide/links)   **iPad**   - [Veteran Device Profiles and Setup \| Digital Divide Consult \| VHA Telehealth Services Intranet (va.gov)](https://vaww.telehealth.va.gov/technology/devices/veteran-devices.asp) - [VA Loaned Device App \| VHA Telehealth Services Intranet](https://vaww.telehealth.va.gov/technology/devices/vald.asp)   **MS Teams**   - [Veterans Affairs Teams Site - Home (sharepoint.com)](https://dvagov.sharepoint.com/sites/MSTeams) - [Microsoft Teams (va.gov)](https://www.oit.va.gov/Services/TRM/ToolPage.aspx?tid=14196) - [Teams Community of Practice (sharepoint.com)](https://dvagov.sharepoint.com/sites/MSTeams/LYNC/SitePages/Teams-Community-of-Practice(1).aspx) - [Bulletins - All Documents (sharepoint.com)](https://dvagov.sharepoint.com/sites/OITEPMOEPMDES/Projects/MSTeams/Bulletins/Forms/AllItems.aspx) |
| **1.3 Sharing Virtual Appointment Access** | **Summary #5^4^:** You can use a **virtual remote stethoscope**with **VVC** and a headset to listen to a Veterans heart and lungs. The Veteran will send a link from **VVC** to allow the provider access and connect the device remotely to perform the activity. | - [Asynchronous Store and Forward Telehealth – Asynchronous... (blackboard.com)](https://vaots.blackboard.com/webapps/blackboard/execute/modulepage/view?course_id=_2439_1&cmp_tab_id=_3136_1) - <https://connectedcare.va.gov/about/outreach-toolkit> - [VA Video Connect (VVC) – VA Video Connect (VVC... (blackboard.com)](https://vaots.blackboard.com/webapps/blackboard/execute/modulepage/view?course_id=_2591_1&cmp_tab_id=_3396_1&mode=view) | **VVC:**   - <https://vaots.blackboard.com/bbcswebdav/library/LibraryContent/VA%20Video%20Connect/VA%20Video%20Connect%20Web%202.X%20Provider%20Guide.pdf> - [VA Video Connect \| VA Mobile](https://mobile.va.gov/app/va-video-connect)   **Virtual remote stethoscope:**   - <https://vaww.telehealth.va.gov/pgm/vvc/during/peripherals.asp> - [vaots.blackboard.com/bbcswebdav/library/Recycle Bin/Technology Videos/EKO Stethoscope Instructional Video/VVC and EKP Stethoscope Instructional VIdeo.html](https://vaots.blackboard.com/bbcswebdav/library/Recycle%20Bin/Technology%20Videos/EKO%20Stethoscope%20Instructional%20Video/VVC%20and%20EKP%20Stethoscope%20Instructional%20VIdeo.html) |
| **1.4 Preparing for Virtual Appointment** | **Summary #22^4,5^:** There are several ways healthcare staff can prepare for an upcoming virtual appointment. 1) Healthcare staff (MSA) can use **MS Outlook** to block off time to ensure the healthcare staff is not double booked and save time by using **VCM** to see all virtual appointments in one location to provide reminder notes to the healthcare staff about an upcoming Veteran appointment. 2) Healthcare staff can use **MS Teams** to coordinate an [interdisciplinary] annual visit during a team huddle. | - [Scheduling -Telehealth – Scheduling - Telehealth Community (blackboard.com)](https://vaots.blackboard.com/webapps/blackboard/execute/modulepage/view?course_id=_2466_1&cmp_tab_id=_3176_1&mode=view) - [VVC Flip Book (blackboard.com)](https://vaots.blackboard.com/bbcswebdav/orgs/vaots_th_sched/thquickflipresource/index.html) - [Microsoft Office Fact Sheet_8-2020 Final__Correction_508_9-2021_corrected_links11172021.pdf (sharepoint.com)](https://dvagov.sharepoint.com/sites/vacovetsprivacy/vhapo/Documents/Guidebooks,%20Fact%20Sheets%20and%20Practice%20Briefs/Microsoft%20Office%20Fact%20Sheet_8-2020%20Final__Correction_508_9-2021_corrected_links11172021.pdf) | **MS Outlook:**   - [Introduction to the Outlook Calendar - Microsoft Support](https://support.microsoft.com/en-us/office/introduction-to-the-outlook-calendar-d94c5203-77c7-48ec-90a5-2e2bc10bd6f8) - [Microsoft Outlook (va.gov)](https://www.oit.va.gov/Services/TRM/ToolPage.aspx?tid=5684)   **VCM:**   - [Virtual Care Manager – Virtual Care Manager Community (blackboard.com)](https://vaots.blackboard.com/webapps/blackboard/execute/modulepage/view?course_id=_2540_1&cmp_tab_id=_3308_1&mode=view) - [Virtual Care Manager User Guide VCM 2.0 (va.gov)](https://mobile.va.gov/sites/default/files/user-manual-vcm.pdf)   **MS Teams:**   - [Veterans Affairs Teams Site - Home (sharepoint.com)](https://dvagov.sharepoint.com/sites/MSTeams) - [Microsoft Teams (va.gov)](https://www.oit.va.gov/Services/TRM/ToolPage.aspx?tid=14196) - [Teams Community of Practice (sharepoint.com)](https://dvagov.sharepoint.com/sites/MSTeams/LYNC/SitePages/Teams-Community-of-Practice(1).aspx) - [Bulletins - All Documents (sharepoint.com)](https://dvagov.sharepoint.com/sites/OITEPMOEPMDES/Projects/MSTeams/Bulletins/Forms/AllItems.aspx) |
|  | **Summary #26^4,5^:** Dedicated healthcare staff can prepare for an upcoming virtual appointment utilizing dedicated team member support with the use of digital VA issued equipment by using **CPRS** to look up Veteran's next appointment, then goes to **CPRS + Notes & Alerts** to scan previous notes related to issues with technology, find out what platform the Veteran used in the past and what type of equipment the Veteran was previously issued. A dedicated healthcare staff member can use **CPRS + ROES** to track ordered technology to prepare for follow up appointment to assess and set up of the device by contacting the Veteran via **telephone** to ensure they have proper equipment and performs a test run to prepare for their upcoming appointment. | - [OCC Telehealth Manual (blackboard.com)](https://vaots.blackboard.com/bbcswebdav/library/LibraryContent/Telehealth%20Foundations-Business-Administration/th_manual/th_manual/TH-manual.html) - [Veteran Device Ordering Process \| Digital Divide Consult \| VHA Telehealth Services Intranet (va.gov)](https://vaww.telehealth.va.gov/technology/devices/vet-process.asp) - [Connected Device Support - VHA Telehealth Services Intranet (va.gov)](https://vaww.telehealth.va.gov/technology/devices/cds.asp) | **CPRS:**   - [CPRS Technical Manual (va.gov)](https://www.va.gov/vdl/documents/Clinical/Comp_Patient_Recrd_Sys_(CPRS)/cprslmtm.pdf)   **CPRS + Notes & Alerts:**   - [CPRS Alerts and Notifications Tools.pptx (sharepoint.com)](https://dvagov.sharepoint.com/:p:/r/sites/InformaticsEducationVHABIL/_layouts/15/Doc.aspx?sourcedoc=%7BCDFF07C7-335F-4C6F-9CE4-E3295B8DD635%7D&file=CPRS%20Alerts%20and%20Notifications%20Tools.pptx&action=edit&mobileredirect=true&DefaultItemOpen=1)   **CPRS + ROES:**   - [Remote Order Entry System (ROES) - VA Authentication Federation Infrastructure (VAAFI)](https://www.va.gov/EAUTH/ROES/index.asp) |
|  | **Summary #28^3,4,5^:** Healthcare staff can prepare for an upcoming virtual appointment with the delivery of educational materials using a dedicated healthcare staff to provide training materials to the Veterans on how to use the virtual platform via **VA & Non-VA YouTube Videos, VA & Non-VA apps.** Healthcare staff can use the **telephone** to instruct the Veteran on how to download and enter data in the **CBTi-Coach app.** Healthcare staff can prepare for an upcoming virtual appointment utilizing dedicated team member support by using **telephone, telephone + Doximity, or VVC** to contact a Veteran to ensure the technology is working properly and provide troubleshooting for issues. Healthcare staff can use **WebEx** for Android users when they can’t connect with **VCM.** Before the start of the virtual session, healthcare staff can use **WebEx** or **VVC** to review an informed consent, explain how the visit will be conducted virtually and review features and functionalities of the platform. | - [Approved Video Technologies \| Following the Pandemic \| VHA Telehealth Services Intranet (va.gov)](https://vaww.telehealth.va.gov/technology/alt/post-pandemic.asp) - [Veteran Device Profiles and Setup \| Digital Divide Consult \| VHA Telehealth Services Intranet (va.gov)](https://vaww.telehealth.va.gov/technology/devices/veteran-devices.asp) - [Prepare for a VA Video Connect Visit \| Veteran Devices and Test Calls \| VHA Telehealth Services Intranet](https://vaww.telehealth.va.gov/pgm/vvc/prepare/test-call.asp) | **VA Apps:**   - [VA App Store \| VA Mobile](https://mobile.va.gov/appstore/)   **CBT-i Coach App:**   - [CBT-i Coach \| VA Mobile](https://mobile.va.gov/app/cbt-i-coach)   **VVC:**   - <https://vaots.blackboard.com/bbcswebdav/library/LibraryContent/VA%20Video%20Connect/VA%20Video%20Connect%20Web%202.X%20Provider%20Guide.pdf> - [VA Video Connect (VVC) – VA Video Connect (VVC... (blackboard.com)](https://vaots.blackboard.com/webapps/blackboard/execute/modulepage/view?course_id=_2591_1&cmp_tab_id=_3396_1&mode=view) - [VA Video Connect Fundamentals \| VHA Telehealth Services Intranet](https://vaww.telehealth.va.gov/pgm/vvc/index.asp) - [VA Video Connect \| VA Mobile](https://mobile.va.gov/app/va-video-connect)   **WebEx:**   - [https://help.webex.com/en-us/article/ruxycv/Sending-Invitations,-Reminders,-and-Registration-Notifications-in-Cisco-Webex-Training](https://help.webex.com/en-us/article/ruxycv/Sending-Invitations,-Reminders,-and-Registration-Notifications-in-Cisco-Webex-Training%20)   **Doximity:**   - [Doximity \| VA Mobile](https://mobile.va.gov/app/doximity) - [About Doximity](https://www.doximity.com/about/company)   **YouTube:**   - [U.S. Dept. of Veterans Affairs – YouTube](https://www.youtube.com/user/DeptVetAffairs) |
| **1.5 Preappointment Huddles** | **Summary #22^4,5^:** … Healthcare staff can use **MS Teams** to coordinate an [interdisciplinary] annual visit during a team huddle. | - [Microsoft Office Fact Sheet_8-2020 Final__Correction_508_9-2021_corrected_links11172021.pdf (sharepoint.com)](https://dvagov.sharepoint.com/sites/vacovetsprivacy/vhapo/Documents/Guidebooks,%20Fact%20Sheets%20and%20Practice%20Briefs/Microsoft%20Office%20Fact%20Sheet_8-2020%20Final__Correction_508_9-2021_corrected_links11172021.pdf) | **MS Teams:**   - [Veterans Affairs Teams Site - Home (sharepoint.com)](https://dvagov.sharepoint.com/sites/MSTeams) - [Microsoft Teams (va.gov)](https://www.oit.va.gov/Services/TRM/ToolPage.aspx?tid=14196) - [Teams Community of Practice (sharepoint.com)](https://dvagov.sharepoint.com/sites/MSTeams/LYNC/SitePages/Teams-Community-of-Practice(1).aspx) - [Bulletins - All Documents (sharepoint.com)](https://dvagov.sharepoint.com/sites/OITEPMOEPMDES/Projects/MSTeams/Bulletins/Forms/AllItems.aspx) |
|  | **Current Best Practice (Summary #13):** Staff can use **MS Teams** to conduct a huddle if a Veteran needs a follow-up. **MS Teams** can also be used to coordinate an annual visit. | - [Microsoft Office Fact Sheet_8-2020 Final__Correction_508_9-2021_corrected_links11172021.pdf (sharepoint.com)](https://dvagov.sharepoint.com/sites/vacovetsprivacy/vhapo/Documents/Guidebooks,%20Fact%20Sheets%20and%20Practice%20Briefs/Microsoft%20Office%20Fact%20Sheet_8-2020%20Final__Correction_508_9-2021_corrected_links11172021.pdf) | **MS Teams:**   - [Veterans Affairs Teams Site - Home (sharepoint.com)](https://dvagov.sharepoint.com/sites/MSTeams) - [Microsoft Teams (va.gov)](https://www.oit.va.gov/Services/TRM/ToolPage.aspx?tid=14196) - [Teams Community of Practice (sharepoint.com)](https://dvagov.sharepoint.com/sites/MSTeams/LYNC/SitePages/Teams-Community-of-Practice(1).aspx) - [Bulletins - All Documents (sharepoint.com)](https://dvagov.sharepoint.com/sites/OITEPMOEPMDES/Projects/MSTeams/Bulletins/Forms/AllItems.aspx) |
| **1.6 Follow-Up Appointment** | **Summary #2^1,3,5^:** Healthcare staff can schedule follow up appointments by using **SM (MHV), VVC Now, WebEx^1^, VCM + Outlook , CPRS** and **telephone.** Healthcare staff can use **SM (MHV)** to answer any questions and schedule medical appointments. | - [Scheduling -Telehealth – Scheduling - Telehealth Community (blackboard.com)](https://vaots.blackboard.com/webapps/blackboard/execute/modulepage/view?course_id=_2466_1&cmp_tab_id=_3176_1&mode=view) - [Prepare for a VA Video Connect Visit \| Scheduling \| VHA Telehealth Services Intranet](https://vaww.telehealth.va.gov/pgm/vvc/prepare/scheduling.asp) | **SM (MHV):**   - [My HealtheVet Resources – My HealtheVet Resources](https://vaots.blackboard.com/webapps/blackboard/execute/modulepage/view?course_id=_3174_1&cmp_tab_id=_4591_1&mode=view) - [Secure Messaging Through My HealtheVet - My HealtheVet Product (va.gov)](https://vaww.va.gov/MYHEALTHEVET/Secure_Messaging.asp)   **VVC NOW:**   - [VVC Now \| VA Mobile](https://mobile.va.gov/app/vvc-now)   **VVC:**   - [VA Video Connect \| VA Mobile](https://mobile.va.gov/app/va-video-connect)   **VCM:**   - [Virtual Care Manager – Virtual Care Manager Community (blackboard.com)](https://vaots.blackboard.com/webapps/blackboard/execute/modulepage/view?course_id=_2540_1&cmp_tab_id=_3308_1&mode=view) - <https://mobile.va.gov/sites/default/files/user-manual-vcm.pdf> - <https://vaots.blackboard.com/bbcswebdav/library/LibraryContent/Virtual%20Care%20Manager/Virtual%20Care%20Manager%20Scheduling%20Guide.pdf>   **Outlook:**   - [Create and send email in Outlook - Microsoft Support](https://support.microsoft.com/en-us/office/create-and-send-email-in-Outlook-19c32deb-08b6-4f90-a211-02bc5f77f360) - [Microsoft Outlook (va.gov)](https://www.oit.va.gov/Services/TRM/ToolPage.aspx?tid=5684)   **WebEx:**   - [Approved Video Technologies \| Following the Pandemic \| VHA Telehealth Services Intranet (va.gov)](https://vaww.telehealth.va.gov/technology/alt/post-pandemic.asp) - <https://help.webex.com/en-us/article/ruxycv/Sending-Invitations,-Reminders,-and-Registration-Notifications-in-Cisco-Webex-Training> - [How to use Webex Meetings - Tutorial - YouTube](https://www.youtube.com/watch?v=zdZCGln8yus)   **CPRS:**   - [CPRS Technical Manual (va.gov)](https://www.va.gov/vdl/documents/Clinical/Comp_Patient_Recrd_Sys_(CPRS)/cprslmtm.pdf) - [CPRS Session 1 Getting Started](https://www.youtube.com/watch?v=y52UtvS95F0) |
|  | **Summary #26^4,5^: …** A dedicated healthcare staff member can use **CPRS + ROES** to track ordered technology to prepare for follow up appointment to assess and set up of the device by contacting the Veteran via **telephone** to ensure they have proper equipment and performs a test run to prepare for their upcoming appointment. | - [OCC Telehealth Manual (blackboard.com)](https://vaots.blackboard.com/bbcswebdav/library/LibraryContent/Telehealth%20Foundations-Business-Administration/th_manual/th_manual/TH-manual.html) - [Veteran Device Ordering Process \| Digital Divide Consult \| VHA Telehealth Services Intranet (va.gov)](https://vaww.telehealth.va.gov/technology/devices/vet-process.asp) - [Connected Device Support - VHA Telehealth Services Intranet (va.gov)](https://vaww.telehealth.va.gov/technology/devices/cds.asp) | **CPRS:**   - [CPRS Technical Manual (va.gov)](https://www.va.gov/vdl/documents/Clinical/Comp_Patient_Recrd_Sys_(CPRS)/cprslmtm.pdf)   **CPRS + ROES:**   - [Remote Order Entry System (ROES) - VA Authentication Federation Infrastructure (VAAFI)](https://www.va.gov/EAUTH/ROES/index.asp) |
| **2. Patient & Staff Technology & Resource Access** | | | |
| **2.1** **Dedicated Resources for Device Consult or  Troubleshooting or  Responding to Alert** | **Summary #4^5^:** Dedicated IT team member or qualified team member may receive a viewer alert from another team member from **CPRS + Notes & Alerts, SM, MHV, MS Teams** or **MS Outlook** about a Veteran having difficulty connecting or needs set up for a virtual appointment. | - [Telehealth Care Coordinators - Training Resources - VHA Telehealth Services Intranet (va.gov)](https://vaww.telehealth.va.gov/roles/cc/index.asp) | **MS Teams:**   - [Veterans Affairs Teams Site - Home (sharepoint.com)](https://dvagov.sharepoint.com/sites/MSTeams) - [Microsoft Teams (va.gov)](https://www.oit.va.gov/Services/TRM/ToolPage.aspx?tid=14196) - [Teams Community of Practice (sharepoint.com)](https://dvagov.sharepoint.com/sites/MSTeams/LYNC/SitePages/Teams-Community-of-Practice(1).aspx) - [Bulletins - All Documents (sharepoint.com)](https://dvagov.sharepoint.com/sites/OITEPMOEPMDES/Projects/MSTeams/Bulletins/Forms/AllItems.aspx)   **MHV:**   - [My HealtheVet Resources – My HealtheVet Resources](https://vaots.blackboard.com/webapps/blackboard/execute/modulepage/view?course_id=_3174_1&cmp_tab_id=_4591_1&mode=view)   **SM (MHV):**   - [Secure Messaging Through My HealtheVet - My HealtheVet Product (va.gov)](https://vaww.va.gov/MYHEALTHEVET/Secure_Messaging.asp)   **CPRS:**   - [CPRS Technical Manual (va.gov)](https://www.va.gov/vdl/documents/Clinical/Comp_Patient_Recrd_Sys_(CPRS)/cprslmtm.pdf)   **CPRS + Notes & Alerts:**   - [CPRS Alerts and Notifications Tools.pptx (sharepoint.com)](https://dvagov.sharepoint.com/:p:/r/sites/InformaticsEducationVHABIL/_layouts/15/Doc.aspx?sourcedoc=%7BCDFF07C7-335F-4C6F-9CE4-E3295B8DD635%7D&file=CPRS%20Alerts%20and%20Notifications%20Tools.pptx&action=edit&mobileredirect=true&DefaultItemOpen=1) |
|  | **Summary #26^4,5^:** Dedicated healthcare staff can prepare for an upcoming virtual appointment utilizing dedicated team member support with the use of digital VA issued equipment by using **CPRS** to look up Veteran's next appointment, then goes to **CPRS + Notes & Alerts** to scan previous notes related to issues with technology, find out what platform the Veteran used in the past and what type of equipment the Veteran was previously issued. A dedicated healthcare staff member can use **CPRS + ROES** to track ordered technology to prepare for follow up appointment to assess and set up of the device by contacting the Veteran via telephone to ensure they have proper equipment and performs a test run to prepare for their upcoming appointment. | - [OCC Telehealth Manual (blackboard.com)](https://vaots.blackboard.com/bbcswebdav/library/LibraryContent/Telehealth%20Foundations-Business-Administration/th_manual/th_manual/TH-manual.html) - [Veteran Device Ordering Process \| Digital Divide Consult \| VHA Telehealth Services Intranet (va.gov)](https://vaww.telehealth.va.gov/technology/devices/vet-process.asp) - [Connected Device Support - VHA Telehealth Services Intranet (va.gov)](https://vaww.telehealth.va.gov/technology/devices/cds.asp) | **CPRS:**   - [CPRS Technical Manual (va.gov)](https://www.va.gov/vdl/documents/Clinical/Comp_Patient_Recrd_Sys_(CPRS)/cprslmtm.pdf)   **CPRS + Notes & Alerts**   - [CPRS Alerts and Notifications Tools.pptx (sharepoint.com)](https://dvagov.sharepoint.com/:p:/r/sites/InformaticsEducationVHABIL/_layouts/15/Doc.aspx?sourcedoc=%7BCDFF07C7-335F-4C6F-9CE4-E3295B8DD635%7D&file=CPRS%20Alerts%20and%20Notifications%20Tools.pptx&action=edit&mobileredirect=true&DefaultItemOpen=1)   **CPRS + ROES:**   - [Remote Order Entry System (ROES) - VA Authentication Federation Infrastructure (VAAFI)](https://www.va.gov/EAUTH/ROES/index.asp) |
| **2.2: Prepare for Visit & Assess Technology** | **Summary #16^1^:** You can use **VA & Non-VA YouTube Videos, VA & Non-VA apps**, and other applicable educational resources to provide training to prepare for a virtual visit and use the technology required. You can even provide a live demonstration with a Veteran. | - <https://connectedcare.va.gov/sites/default/files/2021-10/va-mobile-health-practice-guide.pdf> - [Telehealth Technology Home Page – Telehealth Technology (blackboard.com)](https://vaots.blackboard.com/webapps/blackboard/execute/modulepage/view?course_id=_2451_1&cmp_tab_id=_3154_1&mode=view) | **YouTube:**   - <https://www.youtube.com/user/DeptVetAffairs?reload=9>   **VA Mobile Apps**   - [App Store \| VA Mobile](https://mobile.va.gov/appstore) |
|  | **Summary #26^4,5^:** Dedicated healthcare staff can prepare for an upcoming virtual appointment utilizing dedicated team member support with the use of digital VA issued equipment by using **CPRS** to look up Veteran's next appointment, then goes to **CPRS + Notes & Alerts** to scan previous notes related to issues with technology, find out what platform the Veteran used in the past and what type of equipment the Veteran was previously issued. A dedicated healthcare staff member can use **CPRS + ROES** to track ordered technology to prepare for follow up appointment to assess and set up of the device by contacting the Veteran via telephone to ensure they have proper equipment and performs a test run to prepare for their upcoming appointment. | - [OCC Telehealth Manual (blackboard.com)](https://vaots.blackboard.com/bbcswebdav/library/LibraryContent/Telehealth%20Foundations-Business-Administration/th_manual/th_manual/TH-manual.html) - [Veteran Device Ordering Process \| Digital Divide Consult \| VHA Telehealth Services Intranet (va.gov)](https://vaww.telehealth.va.gov/technology/devices/vet-process.asp) - [Connected Device Support - VHA Telehealth Services Intranet (va.gov)](https://vaww.telehealth.va.gov/technology/devices/cds.asp) | **CPRS:**   - [CPRS Technical Manual (va.gov)](https://www.va.gov/vdl/documents/Clinical/Comp_Patient_Recrd_Sys_(CPRS)/cprslmtm.pdf)   **CPRS + Notes & Alerts:**   - [CPRS Alerts and Notifications Tools.pptx (sharepoint.com)](https://dvagov.sharepoint.com/:p:/r/sites/InformaticsEducationVHABIL/_layouts/15/Doc.aspx?sourcedoc=%7BCDFF07C7-335F-4C6F-9CE4-E3295B8DD635%7D&file=CPRS%20Alerts%20and%20Notifications%20Tools.pptx&action=edit&mobileredirect=true&DefaultItemOpen=1)   **CPRS + ROES:**   - [Remote Order Entry System (ROES) - VA Authentication Federation Infrastructure (VAAFI)](https://www.va.gov/EAUTH/ROES/index.asp) |
|  | **Summary #27^5^:** You can open **CPRS + ROES** to prepare for a call while simultaneously looking at a face sheet for the Veteran's contact information such as telephone number and email address. | - [cprsguium_ro.pdf (va.gov)](https://www.va.gov/vdl/documents/Clinical/Comp_Patient_Recrd_Sys_(CPRS)/cprsguium_ro.pdf) | **CPRS:**   - [CPRS Technical Manual (va.gov)](https://www.va.gov/vdl/documents/Clinical/Comp_Patient_Recrd_Sys_(CPRS)/cprslmtm.pdf)   **CPRS + ROES:**   - [Remote Order Entry System (ROES) - VA Authentication Federation Infrastructure (VAAFI)](https://www.va.gov/EAUTH/ROES/index.asp) |
| **2.3: Provide Troubleshooting** | **Summary #1^1,5^:** When experiencing connectivity issues or other technical problems during **VVC** appointment/session, provider or dedicated IT team member supplement their communication with the Veteran with a phone call **using Doximity** or **Cisco Jabber** to continue the session or troubleshoot the problem. | - [Connected Device Support - VHA Telehealth Services Intranet (va.gov)](https://vaww.telehealth.va.gov/technology/devices/cds.asp) | **Doximity:**   - <https://www.doximity.com/?_c=bWFya2V0aW5nOjphZHdvcmRzOjpkb3hpbWl0eV8yMDE3MDg%3D.bsY2OiP1dMj6Q2iFHU6UNh8d4Xw%3D&return_to=https%3A%2F%2Fwww.doximity.com%2Fnewsfeed%3Futm_source%3Dgoogle%26utm_campaign%3Dmarketing_adwords&utm_source=bing&utm_campaign=marketing_adwords&campaignid=376136971&adgroupid=1225955646554449&adid=&msclkid=3c9471951f2b11e6867c134572d9c6c7> - <https://mobile.va.gov/app/doximity>   **Cisco Jabber:**   - [https://www.oit.va.gov/Services/TRM/TRMRedirectPage.aspx?type=W^&tid=9777^](https://www.oit.va.gov/Services/TRM/TRMRedirectPage.aspx?type=W%5e&tid=9777%5e%20) - [Cisco Jabber for Windows - Cisco](https://www.cisco.com/c/en/us/support/unified-communications/jabber-windows/series.html)   **VVC:**   - <https://vaots.blackboard.com/bbcswebdav/library/LibraryContent/VA%20Video%20Connect/VA%20Video%20Connect%20Web%202.X%20Provider%20Guide.pdf> - [VA Video Connect \| VA Mobile](https://mobile.va.gov/app/va-video-connect) |
|  | **Summary #26^4,5^:** Dedicated healthcare staff can prepare for an upcoming virtual appointment utilizing dedicated team member support with the use of digital VA issued equipment by using **CPRS** to look up Veteran's next appointment, then goes to **CPRS + Notes & Alerts** to scan previous notes related to issues with technology, find out what platform the Veteran used in the past and what type of equipment the Veteran was previously issued. A dedicated healthcare staff member can use **CPRS + ROES** to track ordered technology to prepare for follow up appointment to assess and set up of the device by contacting the Veteran via telephone to ensure they have proper equipment and performs a test run to prepare for their upcoming appointment. | - [OCC Telehealth Manual (blackboard.com)](https://vaots.blackboard.com/bbcswebdav/library/LibraryContent/Telehealth%20Foundations-Business-Administration/th_manual/th_manual/TH-manual.html) - [Veteran Device Ordering Process \| Digital Divide Consult \| VHA Telehealth Services Intranet (va.gov)](https://vaww.telehealth.va.gov/technology/devices/vet-process.asp) - [Connected Device Support - VHA Telehealth Services Intranet (va.gov)](https://vaww.telehealth.va.gov/technology/devices/cds.asp) | **CPRS:**   - [CPRS Technical Manual (va.gov)](https://www.va.gov/vdl/documents/Clinical/Comp_Patient_Recrd_Sys_(CPRS)/cprslmtm.pdf)   **CPRS + Notes & Alerts:**   - [CPRS Alerts and Notifications Tools.pptx (sharepoint.com)](https://dvagov.sharepoint.com/:p:/r/sites/InformaticsEducationVHABIL/_layouts/15/Doc.aspx?sourcedoc=%7BCDFF07C7-335F-4C6F-9CE4-E3295B8DD635%7D&file=CPRS%20Alerts%20and%20Notifications%20Tools.pptx&action=edit&mobileredirect=true&DefaultItemOpen=1)   **CPRS + ROES:**   - [Remote Order Entry System (ROES) - VA Authentication Federation Infrastructure (VAAFI)](https://www.va.gov/EAUTH/ROES/index.asp) |
|  | **Summary #29^3^: MS Teams** can be used to assist with troubleshooting technology issues. | - [Microsoft Office Fact Sheet_8-2020 Final__Correction_508_9-2021_corrected_links11172021.pdf (sharepoint.com)](https://dvagov.sharepoint.com/sites/vacovetsprivacy/vhapo/Documents/Guidebooks,%20Fact%20Sheets%20and%20Practice%20Briefs/Microsoft%20Office%20Fact%20Sheet_8-2020%20Final__Correction_508_9-2021_corrected_links11172021.pdf) | **MS Teams:**   - [Veterans Affairs Teams Site - Home (sharepoint.com)](https://dvagov.sharepoint.com/sites/MSTeams) - [Microsoft Teams (va.gov)](https://www.oit.va.gov/Services/TRM/ToolPage.aspx?tid=14196) - [Teams Community of Practice (sharepoint.com)](https://dvagov.sharepoint.com/sites/MSTeams/LYNC/SitePages/Teams-Community-of-Practice(1).aspx) - [Bulletins - All Documents (sharepoint.com)](https://dvagov.sharepoint.com/sites/OITEPMOEPMDES/Projects/MSTeams/Bulletins/Forms/AllItems.aspx) |
| **2.4: Provide Training & Support** | **Summary #16^1^:** You can use **VA & Non-VA YouTube Videos, VA & Non-VA apps**, and other applicable educational resources to provide training to prepare for a virtual visit and use the technology required. You can even provide a live demonstration with a Veteran. | - <https://connectedcare.va.gov/sites/default/files/2021-10/va-mobile-health-practice-guide.pdf> - [Telehealth Technology Home Page – Telehealth Technology (blackboard.com)](https://vaots.blackboard.com/webapps/blackboard/execute/modulepage/view?course_id=_2451_1&cmp_tab_id=_3154_1&mode=view) | **YouTube:**   - <https://www.youtube.com/user/DeptVetAffairs?reload=9>   **VA Mobile Apps**   - [App Store \| VA Mobile](https://mobile.va.gov/appstore) |
|  | **Summary #28^3,4,5^:** Healthcare staff can prepare for an upcoming virtual appointment with the delivery of educational materials using a dedicated healthcare staff to provide training materials to the Veterans on how to use the virtual platform via **VA & Non-VA YouTube Videos, VA & Non-VA apps.** Healthcare staff can use the **telephone** to instruct the Veteran on how to download and enter data in the **CBTi-Coach app.** Healthcare staff can prepare for an upcoming virtual appointment utilizing dedicated team member support by using **telephone, telephone + Doximity, or VVC** to contact a Veteran to ensure the technology is working properly and provide troubleshooting for issues. Healthcare staff can use **WebEx** for Android users when they can’t connect with **VCM.** Before the start of the virtual session, healthcare staff can use **WebEx** or **VVC** to review an informed consent, explain how the visit will be conducted virtually and review features and functionalities of the platform. | - [Approved Video Technologies \| Following the Pandemic \| VHA Telehealth Services Intranet (va.gov)](https://vaww.telehealth.va.gov/technology/alt/post-pandemic.asp) - [Veteran Device Profiles and Setup \| Digital Divide Consult \| VHA Telehealth Services Intranet (va.gov)](https://vaww.telehealth.va.gov/technology/devices/veteran-devices.asp) - [Prepare for a VA Video Connect Visit \| Veteran Devices and Test Calls \| VHA Telehealth Services Intranet](https://vaww.telehealth.va.gov/pgm/vvc/prepare/test-call.asp) | **VA Apps:**   - [VA App Store \| VA Mobile](https://mobile.va.gov/appstore/)   **CBT-i Coach App:**   - [CBT-i Coach \| VA Mobile](https://mobile.va.gov/app/cbt-i-coach)   **VVC:**   - <https://vaots.blackboard.com/bbcswebdav/library/LibraryContent/VA%20Video%20Connect/VA%20Video%20Connect%20Web%202.X%20Provider%20Guide.pdf> - [VA Video Connect (VVC) – VA Video Connect (VVC... (blackboard.com)](https://vaots.blackboard.com/webapps/blackboard/execute/modulepage/view?course_id=_2591_1&cmp_tab_id=_3396_1&mode=view) - [VA Video Connect Fundamentals \| VHA Telehealth Services Intranet](https://vaww.telehealth.va.gov/pgm/vvc/index.asp) - [VA Video Connect \| VA Mobile](https://mobile.va.gov/app/va-video-connect)   **WebEx:**   - [https://help.webex.com/en-us/article/ruxycv/Sending-Invitations,-Reminders,-and-Registration-Notifications-in-Cisco-Webex-Training](https://help.webex.com/en-us/article/ruxycv/Sending-Invitations,-Reminders,-and-Registration-Notifications-in-Cisco-Webex-Training%20)   **Doximity:**   - [Doximity \| VA Mobile](https://mobile.va.gov/app/doximity) - [About Doximity](https://www.doximity.com/about/company)   **YouTube:**   - [U.S. Dept. of Veterans Affairs – YouTube](https://www.youtube.com/user/DeptVetAffairs) |
| **2.5: Obtaining Digital Equipment** | **Summary #21^5^:** Dedicated IT team member or qualified staff member can use **CPRS + ROES** for **iPad** orders for Veterans. The order can be tracked and when delivered, a follow-up appoint can be set up to instruct Veteran on how to set up and use the device. | - [VA iPad Loaner Program/Digital Divide (sharepoint.com)](https://dvagov.sharepoint.com/sites/SFC/Tele/VVC/SitePages/VA-iPad-Loaner-Program-Digital-Divide.aspx) | **CPRS:**   - [CPRS Technical Manual (va.gov)](https://www.va.gov/vdl/documents/Clinical/Comp_Patient_Recrd_Sys_(CPRS)/cprslmtm.pdf)   **CPRS + ROES:**   - [Remote Order Entry System (ROES) - VA Authentication Federation Infrastructure (VAAFI)](https://www.va.gov/EAUTH/ROES/index.asp) |
|  | **Summary #26^4,5^:** Dedicated healthcare staff can prepare for an upcoming virtual appointment utilizing dedicated team member support with the use of digital VA issued equipment by using **CPRS** to look up Veteran's next appointment, then goes to **CPRS + Notes & Alerts** to scan previous notes related to issues with technology, find out what platform the Veteran used in the past and what type of equipment the Veteran was previously issued. A dedicated healthcare staff member can use **CPRS + ROES** to track ordered technology to prepare for follow up appointment to assess and set up of the device by contacting the Veteran via telephone to ensure they have proper equipment and performs a test run to prepare for their upcoming appointment. | - [OCC Telehealth Manual (blackboard.com)](https://vaots.blackboard.com/bbcswebdav/library/LibraryContent/Telehealth%20Foundations-Business-Administration/th_manual/th_manual/TH-manual.html) - [Veteran Device Ordering Process \| Digital Divide Consult \| VHA Telehealth Services Intranet (va.gov)](https://vaww.telehealth.va.gov/technology/devices/vet-process.asp) - [Connected Device Support - VHA Telehealth Services Intranet (va.gov)](https://vaww.telehealth.va.gov/technology/devices/cds.asp) | **CPRS:**   - [CPRS Technical Manual (va.gov)](https://www.va.gov/vdl/documents/Clinical/Comp_Patient_Recrd_Sys_(CPRS)/cprslmtm.pdf)   **CPRS + Notes & Alerts:**   - [CPRS Alerts and Notifications Tools.pptx (sharepoint.com)](https://dvagov.sharepoint.com/:p:/r/sites/InformaticsEducationVHABIL/_layouts/15/Doc.aspx?sourcedoc=%7BCDFF07C7-335F-4C6F-9CE4-E3295B8DD635%7D&file=CPRS%20Alerts%20and%20Notifications%20Tools.pptx&action=edit&mobileredirect=true&DefaultItemOpen=1)   **CPRS + ROES:**   - [Remote Order Entry System (ROES) - VA Authentication Federation Infrastructure (VAAFI)](https://www.va.gov/EAUTH/ROES/index.asp?msclkid=2eb610a2d15211ecb85a288584a5fe61) |
|  | **Summary #36^1,4,5^:** During the history and examination phase of treatment, staff can use **CPRS + ROES** to consult for device issuance. | - [Veteran Device Ordering Process \| Digital Divide Consult \| VHA Telehealth Services Intranet (va.gov)](https://vaww.telehealth.va.gov/technology/devices/vet-process.asp) | **CPRS:**   - [CPRS Technical Manual (va.gov)](https://www.va.gov/vdl/documents/Clinical/Comp_Patient_Recrd_Sys_(CPRS)/cprslmtm.pdf) - [CPRS Session 1 Getting Started](https://www.youtube.com/watch?v=y52UtvS95F0) - [Consult Tool Box Archives \| Office of Information and Technology (va.gov)](https://vaww.oit.va.gov/category/products/release-notes/ctb/)   **CPRS + ROES:**   - [Remote Order Entry System (ROES) - VA Authentication Federation Infrastructure (VAAFI)](https://www.va.gov/EAUTH/ROES/index.asp) |
| **3. Communication** | | | |
| **3.1: Provider, Veteran, Non-veteran Communication** | **Summary #2^1,3,5^:** …Healthcare staff can use **SM (MHV)** to answer any questions and schedule medical appointments. | - [MHV_SM_HCT_User_Manual_August_2023.pdf (va.gov)](https://vaww.va.gov/MYHEALTHEVET/docs/secure_messaging/MHV_SM_HCT_User_Manual_August_2023.pdf) | **SM (MHV):**   - [My HealtheVet Resources – My HealtheVet Resources](https://vaots.blackboard.com/webapps/blackboard/execute/modulepage/view?course_id=_3174_1&cmp_tab_id=_4591_1&mode=view) - [Secure Messaging Through My HealtheVet - My HealtheVet Product (va.gov)](https://vaww.va.gov/MYHEALTHEVET/Secure_Messaging.asp) |
|  | **Summary #14^1,3,5^:** Provider follows up with Veteran via **SM (MHV), MS Outlook**, or **telephone** to get updates from the Veteran, assess progress after treatment plan, confirm device was received, and share education material. Communication with the Veteran can be done via **SM (MHV)** or **MS Outlook.** | - [Virtual Care Best Practices Community – Virtual Care ... (blackboard.com)](https://vaots.blackboard.com/webapps/blackboard/execute/modulepage/view?course_id=_3330_1&cmp_tab_id=_5023_1&mode=view) | **SM (MHV):**   - [Secure Messaging - My HealtheVet - My HealtheVet (va.gov)](https://www.myhealth.va.gov/mhv-portal-web/secure-messaging-spotlight) - [Secure Messaging Through My HealtheVet - My HealtheVet Product (va.gov)](https://vaww.va.gov/MYHEALTHEVET/Secure_Messaging.asp)   **Outlook:**   - [Microsoft Outlook (va.gov)](https://www.oit.va.gov/Services/TRM/ToolPage.aspx?tid=5684) |
|  | **Summary #15^3^: Direct texting** via an appropriate texting platform, or **SM (MHV)** provides asynchronous communication for non-verbal patients. These are appropriate forms of communication as an alternative to verbal communication. | - [Asynchronous Store and Forward Telehealth – Asynchronous... (blackboard.com)](https://vaots.blackboard.com/webapps/blackboard/execute/modulepage/view?course_id=_2439_1&cmp_tab_id=_3136_1&mode=view) | **Direct Texting:**   - [DirectMessagingCCPFactSheet.pdf (va.gov)](https://www.va.gov/VHIE/docs/DirectMessagingCCPFactSheet.pdf)   **SM (MHV):**   - [My HealtheVet Resources – My HealtheVet Resources](https://vaots.blackboard.com/webapps/blackboard/execute/modulepage/view?course_id=_3174_1&cmp_tab_id=_4591_1&mode=view) - [Secure Messaging Through My HealtheVet - My HealtheVet Product (va.gov)](https://vaww.va.gov/MYHEALTHEVET/Secure_Messaging.asp) |
|  | **Summary #18^1,2,3,4,5^:** Veteran shares treatment and post operation updates, or express concerns or any matter related to care via **SM (MHV)** or **telephone** to obtain provider’s feedback. Test results can be accessed with **MHV;** and images can be shared in **My VA Images** to facilitate these communications. | - [MHV_SM_HCT_User_Manual_August_2023.pdf (va.gov)](https://vaww.va.gov/MYHEALTHEVET/docs/secure_messaging/MHV_SM_HCT_User_Manual_August_2023.pdf) | **SM (MHV):**   - <https://www.myhealth.va.gov/mhv-portal-web/home> - [My HealtheVet Resources – My HealtheVet Resources](https://vaots.blackboard.com/webapps/blackboard/execute/modulepage/view?course_id=_3174_1&cmp_tab_id=_4591_1&mode=view) - [Secure Messaging Through My HealtheVet – My HealtheVet Product (va.gov)](https://vaww.va.gov/MYHEALTHEVET/Secure_Messaging.asp)   **My VA Images:**   - [My VA Images – My VA Images (MVAI) Community](https://vaots.blackboard.com/webapps/blackboard/execute/modulepage/view?course_id=_2480_1&cmp_tab_id=_3204_1&mode=view) - [Asynchronous SFT from Home My VA Images with VCM-Telehealth Operations Manual](https://learn-us-gov-west-1-prod-fleet01-xythos.s3.us-gov-west-1.amazonaws.com/612eabb1bae6a/1829967?response-cache-control=private%2C%20max-age%3D10800&response-content-disposition=inline%3B%20filename%2A%3DUTF-8%27%27Asynchronous%2520Store-and%2520Forward%2520Telehealth%2520from%2520Home%2520My%2520VA%2520Images%2520with%2520Virtual%2520Care%2520Manager-Telehealth%2520Operations%2520Manual.pdf&response-content-type=application%2Fpdf&X-Amz-Security-Token=FwoDYXdzEDUaDH1rNylD9mAF0StfNiLlBFq4%2FAB%2BwI5kYvt9bHMNvYg2VlTh%2FltGRCoNM8dkUJJ29R3WpD6yt63ricgawRC9BIUZ3irUUBUf2%2FS0IG2hKBT%2BfC7KcV3r2vqZaWx5FfxDrjjOnqbVOiaKrmavoDr6tJlgQkCIq58vfNaJNqkeq10gAJQI9w7JdUSA64FSHHa7iZvoqsGo2OuomNkGjipun9kj7roHgpkfn8S3BBTKSHZpumd45kst%2BmB49a6%2F8qGsTSurvmSLC6C%2FmT3p23ojNEge14v1vugYyLLGUyLBxSeCexWMFLqogjzSSuD7PClhDV8W%2BUuRERB24DZ%2FqvWmXxF4iU0yGEF5MsIOaDhv04Zujq9nqTDZ0C42VmOsjkh0UxvNC71GgvHaSxtwKtPiLK1oG3Bcw6c8jIun7R3arcDIhedtRWJ%2Bnhb6isufurcQQSg1fK1bxeAaSpVfmv7HDDZ4OC3kYEdWIi1QiFV%2BpjoMS1Yk34s%2BjOUmPekS5A7SJBcZ7CPB4m18QHP5Wetw7i7ViraucdZmPY9luRR%2BEEeFUUsjz7iyGz0nvlzk8nlt3K%2Ba%2BMIuN3C83sbAfVKhkiCa5rXNCPr%2FASOo5CsxYYA5PgGj7zfzXRmNXNp%2BQ%2BCUbex%2FoGoTgFFfvFGPoTmFdi9A2Z3Xe7UHhrPbS596orhLliKOUt6XbtZePHMvESMi4tpqGY%2FsOUnenpTvjq8VNmHrYA6AiFITrpnK33vcE7ItXfV%2By9cHRD2XYiAI21gFSvYRjOeUqSmCn%2B3yRkMekkJFJzCJqrnne1M05RJqnwv4Ft0xUGLWBR6vrKrbTlbwYv9DgNko4KKKvQYyQUE5H5GmFw5Qsy3czXLBLXizhkX%2BrErWuz2ulpgSvhlVic%2FpxwWPgiA1S%2FHb3nB2IY8A9fQ8ZU0px0%2BkZeuzjuX4&X-Amz-Algorithm=AWS4-HMAC-SHA256&X-Amz-Date=20250204T223000Z&X-Amz-SignedHeaders=host&X-Amz-Expires=10800&X-Amz-Credential=ASIAUMDY6U44W7SUOGKQ%2F20250204%2Fus-gov-west-1%2Fs3%2Faws4_request&X-Amz-Signature=6206807578f4b07356beaeec6883ee06963f1a45e24ad62cd623a033de76add4) - [My VA Images \| VHA Telehealth Services Intranet](https://vaww.telehealth.va.gov/pgm/mvai/index.asp) - [My VA Images FAQs](https://learn-us-gov-west-1-prod-fleet01-xythos.s3.us-gov-west-1.amazonaws.com/612eabb1bae6a/1856363?response-cache-control=private%2C%20max-age%3D10800&response-content-disposition=inline%3B%20filename%2A%3DUTF-8%27%27MY%2520VA%2520Images%2520FAQ.pdf&response-content-type=application%2Fpdf&X-Amz-Security-Token=FwoDYXdzEHoaDJwdXJkLIHpjrnQu5yLlBHtX8fdIkynj5PZ%2BhxNhtOTvvyF2aYPxjVDtfyGeOmpxKTtzQtq5SCBc4u9FlyqED%2BcALPCek66akU9ZIo1GnPqcKEm%2BEppo5bwAxrMJSMzamLt%2FJeFwMo1PABaa4VBC3iZ66cMtTH%2FXevw4aPkra8RAOvE7KFzw6OxwOj8RMtulHoldeEsjumq0d4P2ZVxbdRuzdeb5QXZ5NGA9gEVOwhjh87GVtdbQds1GnCfT%2FCMdoKpyJOdnkupE1qSvcNUHo1MLlfF2T%2F4ssMLkgCnocE4U0lGmNJjLB667YX7uNTOEi%2F3FwLf5SmApj0cY323lQFtSfbU2LCLAYbdpy7Q8zxl1e1LPCaaI65W1B5qon26fkSDjmmnxd2BcoWlZfnno5o1MlipzsvLDLMq9VIHHmIPYpK03Dw9JWvVfPs2wTtsg7Jk%2FM0cVaWMSQMWmmhzsj7CCF2ITl4nFm7WZiCpDYmzkYXZTfWki4S0ebRnSGwIOaN9Pis5efhd48spnjMc3SyN2szoOpT0XLWBcdLj6BDDf0WuLoGT2yrmuXPFcRu96s6ywfAgt6d86CEjFxyHvW9B4kJ4toHnhNittnEczTsEkVXshm3xvhakSW0MWVqiBMRkOmydfyzhIOxtZZLWONN5d%2BJ2HEc3D1SLTzrafeTUo2%2BeQ18JcjYipgCpTNYIGGLig5BIvhRHvT2ncl3yQ3feygb%2F9sdQXK8W1jwCFMdKtCBDqOwf%2FThKW1D2EMiIvbkJAdJjKXgYtwXi5WeZCEqfRzSTmeQnTqZUjlVIrkdj24dQsgISP7QW35vj4DmDDrKjqpQIo%2BMSZvQYyQc2S6lfGnf%2FriFqA7Xpw43f9s2X9R2qPbw%2BC5AzzECMmEtCfaCAHo3TQCanEwdV0HZTWYPW4oN6y12EuQ%2BCrvaMA&X-Amz-Algorithm=AWS4-HMAC-SHA256&X-Amz-Date=20250207T193000Z&X-Amz-SignedHeaders=host&X-Amz-Expires=10800&X-Amz-Credential=ASIAUMDY6U442RGZC5OF%2F20250207%2Fus-gov-west-1%2Fs3%2Faws4_request&X-Amz-Signature=c7922ac5ea532bac22f0df6ad4133dba7a9f91ada75638b282c3604047dc305d) |
|  | **Summary #30^1,2^:** Staff and providers encourage Veterans to use **SM (MHV)** to communicate and to use **VA Mobile apps** and tracking devices such as **FitBit** or **Apple Watch** for tracking communication and health data. | - [MHV_SM_HCT_User_Manual_August_2023.pdf (va.gov)](https://vaww.va.gov/MYHEALTHEVET/docs/secure_messaging/MHV_SM_HCT_User_Manual_August_2023.pdf) - [Peripheral Devices \| During the VA Video Connect Visit \| VHA Telehealth Services Intranet](https://vaww.telehealth.va.gov/pgm/vvc/during/peripherals.asp) | **VA Mobile Apps:**   - [VA Mobile Health Practice Guide](https://connectedcare.va.gov/sites/default/files/2021-10/va-mobile-health-practice-guide.pdf)   **SM (MHV):**   - <https://www.myhealth.va.gov/mhv-portal-web/home> - [My HealtheVet Resources – My HealtheVet Resources](https://vaots.blackboard.com/webapps/blackboard/execute/modulepage/view?course_id=_3174_1&cmp_tab_id=_4591_1&mode=view) - [Secure Messaging Through My HealtheVet – My HealtheVet Product (va.gov)](https://vaww.va.gov/MYHEALTHEVET/Secure_Messaging.asp)   **FitBit/Apple Watch:**   - [Digital Health Innovations - VA-Fitbit-HowToParticipate-1P.pdf - All Documents (sharepoint.com)](https://dvagov.sharepoint.com/sites/VHAV1NECIE/DHI/Shared%20Documents/Forms/AllItems.aspx?id=%2Fsites%2FVHAV1NECIE%2FDHI%2FShared%20Documents%2FVA%2DFitbit%2DHowToParticipate%2D1P%2Epdf&parent=%2Fsites%2FVHAV1NECIE%2FDHI%2FShared%20Documents) - [Digital Health Innovations - Fitbit Sense - Getting Started Guide - Veterans.pdf - All Documents (sharepoint.com)](https://dvagov.sharepoint.com/sites/VHAV1NECIE/DHI/Shared%20Documents/Forms/AllItems.aspx?id=%2Fsites%2FVHAV1NECIE%2FDHI%2FShared%20Documents%2FFitbit%20Sense%20%2D%20Getting%20Started%20Guide%20%2D%20Veterans%2Epdf&parent=%2Fsites%2FVHAV1NECIE%2FDHI%2FShared%20Documents) |
|  | **Summary #34^3^:** You can use **VVC** to lock a therapy session with Veterans so there are no unnecessary intrusions from others. Healthcare staff can also use **VVC** to perform 3-way calling and invite family members into sessions with Veterans. | - [VA Video Connect (VVC) – VA Video Connect (VVC... (blackboard.com)](https://vaots.blackboard.com/webapps/blackboard/execute/modulepage/view?course_id=_2591_1&cmp_tab_id=_3396_1&mode=view) | **VVC:**   - <https://vaots.blackboard.com/bbcswebdav/library/LibraryContent/VA%20Video%20Connect/VA%20Video%20Connect%20Web%202.X%20Provider%20Guide.pdf> |
| **3.2 Internal & External Interdisciplinary Coordination & Communication** | **Summary #3^1,2,3,4,5^:** Healthcare staff can use **MS Teams, MS Outlook, Telephone, VCM** for interdisciplinary communication and coordination regarding healthcare consultation, data collection planning, ordering equipment or to conduct team meetings and huddles. Specifically, IT members can use **VCM** to communicate or consult on IT issues with other providers. | - [Virtual Care Best Practices Community – Virtual Care ... (blackboard.com)](https://vaots.blackboard.com/webapps/blackboard/execute/modulepage/view?course_id=_3330_1&cmp_tab_id=_5023_1&mode=view) - [Microsoft Office Fact Sheet_8-2020 Final__Correction_508_9-2021_corrected_links11172021.pdf (sharepoint.com)](https://dvagov.sharepoint.com/sites/vacovetsprivacy/vhapo/Documents/Guidebooks,%20Fact%20Sheets%20and%20Practice%20Briefs/Microsoft%20Office%20Fact%20Sheet_8-2020%20Final__Correction_508_9-2021_corrected_links11172021.pdf) - [Remote Patient Monitoring-Home Telehealth – Remote ... (blackboard.com)](https://vaots.blackboard.com/webapps/blackboard/execute/modulepage/view?course_id=_2686_1&cmp_tab_id=_3628_1&mode=view) - [Remote Patient Monitoring-Home Telehealth Support Staff &ndash... (blackboard.com)](https://vaots.blackboard.com/webapps/blackboard/execute/modulepage/view?course_id=_2501_1&cmp_tab_id=_3225_1&mode=view) | **MS Teams:**   - [Veterans Affairs Teams Site - Home (sharepoint.com)](https://dvagov.sharepoint.com/sites/MSTeams) - [Microsoft Teams (va.gov)](https://www.oit.va.gov/Services/TRM/ToolPage.aspx?tid=14196) - [Teams Community of Practice (sharepoint.com)](https://dvagov.sharepoint.com/sites/MSTeams/LYNC/SitePages/Teams-Community-of-Practice(1).aspx) - [Bulletins - All Documents (sharepoint.com)](https://dvagov.sharepoint.com/sites/OITEPMOEPMDES/Projects/MSTeams/Bulletins/Forms/AllItems.aspx)   **MS Outlook:**   - [Introduction to the Outlook Calendar - Microsoft Support](https://support.microsoft.com/en-us/office/introduction-to-the-outlook-calendar-d94c5203-77c7-48ec-90a5-2e2bc10bd6f8) - [Microsoft Outlook (va.gov)](https://www.oit.va.gov/Services/TRM/ToolPage.aspx?tid=5684)   **VCM:**   - [Virtual Care Manager – Virtual Care Manager Community (blackboard.com)](https://vaots.blackboard.com/webapps/blackboard/execute/modulepage/view?course_id=_2540_1&cmp_tab_id=_3308_1&mode=view) |
|  | **Summary #4^5^:** Dedicated IT team member or qualified team member may receive a viewer alert from another team member from **CPRS + Notes & Alerts, SM (MHV), MS Teams** or **MS Outlook** about a Veteran having difficulty connecting or needs set up for a virtual appointment. | - [Telehealth Care Coordinators - Training Resources - VHA Telehealth Services Intranet (va.gov)](https://vaww.telehealth.va.gov/roles/cc/index.asp) | **CPRS:**   - [CPRS Technical Manual (va.gov)](https://www.va.gov/vdl/documents/Clinical/Comp_Patient_Recrd_Sys_(CPRS)/cprslmtm.pdf)   **CPRS + Notes & Alerts:**   - [CPRS Alerts and Notifications Tools.pptx (sharepoint.com)](https://dvagov.sharepoint.com/:p:/r/sites/InformaticsEducationVHABIL/_layouts/15/Doc.aspx?sourcedoc=%7BCDFF07C7-335F-4C6F-9CE4-E3295B8DD635%7D&file=CPRS%20Alerts%20and%20Notifications%20Tools.pptx&action=edit&mobileredirect=true&DefaultItemOpen=1)   **SM (MHV):**   - [My HealtheVet Resources – My HealtheVet Resources](https://vaots.blackboard.com/webapps/blackboard/execute/modulepage/view?course_id=_3174_1&cmp_tab_id=_4591_1&mode=view) - [Secure Messaging Through My HealtheVet - My HealtheVet Product (va.gov)](https://vaww.va.gov/MYHEALTHEVET/Secure_Messaging.asp)   **MS Teams:**   - [Microsoft Teams (va.gov)](https://www.oit.va.gov/Services/TRM/ToolPage.aspx?tid=14196)   **MS Outlook:**   - [Microsoft Outlook (va.gov)](https://www.oit.va.gov/Services/TRM/ToolPage.aspx?tid=5684) |
|  | **Summary #10^3,4^:** You can use **CPRS, telephone, MS Teams, VVC** among clinical team members, including other disciplines, to share information and discuss Veteran care during the session, to prepare for follow-ups and/or data collection, or to alert providers to join a session. Healthcare staff can use these VHRs to manage communication and notes about medications, appointments, or supplies. **CPRS** can be used to track notes throughout the care continuum phases. | - [Virtual Care Best Practices Community – Virtual Care ... (blackboard.com)](https://vaots.blackboard.com/webapps/blackboard/execute/modulepage/view?course_id=_3330_1&cmp_tab_id=_5023_1&mode=view) - [VA Video Connect (VVC) – VA Video Connect (VVC... (blackboard.com)](https://vaots.blackboard.com/webapps/blackboard/execute/modulepage/view?course_id=_2591_1&cmp_tab_id=_3396_1&mode=view) | **CPRS:**   - [CPRS Technical Manual (va.gov)](https://www.va.gov/vdl/documents/Clinical/Comp_Patient_Recrd_Sys_(CPRS)/cprslmtm.pdf)   **MS Teams:**   - [Veterans Affairs Teams Site - Home (sharepoint.com)](https://dvagov.sharepoint.com/sites/MSTeams) - [Microsoft Teams (va.gov)](https://www.oit.va.gov/Services/TRM/ToolPage.aspx?tid=14196) - [Teams Community of Practice (sharepoint.com)](https://dvagov.sharepoint.com/sites/MSTeams/LYNC/SitePages/Teams-Community-of-Practice(1).aspx) - [Bulletins - All Documents (sharepoint.com)](https://dvagov.sharepoint.com/sites/OITEPMOEPMDES/Projects/MSTeams/Bulletins/Forms/AllItems.aspx)   **VVC:**   - <https://vaots.blackboard.com/bbcswebdav/xid-512168_1> - [VA Video Connect \| VA Mobile](https://mobile.va.gov/app/va-video-connect) |
|  | **Summary #22^4,5^:** There are several ways healthcare staff can prepare for an upcoming virtual appointment. 1) Healthcare staff can use **MS Outlook** to block off time to ensure the healthcare staff is not double booked and save time by using **VCM** to see all virtual appointments in one location to provide reminder notes to the healthcare staff about an upcoming Veteran appointment. 2) Healthcare staff can use **MS Teams** to coordinate an [interdisciplinary] annual visit during a team huddle. | - [Scheduling -Telehealth – Scheduling - Telehealth Community (blackboard.com)](https://vaots.blackboard.com/webapps/blackboard/execute/modulepage/view?course_id=_2466_1&cmp_tab_id=_3176_1&mode=view) - [VVC Flip Book (blackboard.com)](https://vaots.blackboard.com/bbcswebdav/orgs/vaots_th_sched/thquickflipresource/index.html) - [Microsoft Office Fact Sheet_8-2020 Final__Correction_508_9-2021_corrected_links11172021.pdf (sharepoint.com)](https://dvagov.sharepoint.com/sites/vacovetsprivacy/vhapo/Documents/Guidebooks,%20Fact%20Sheets%20and%20Practice%20Briefs/Microsoft%20Office%20Fact%20Sheet_8-2020%20Final__Correction_508_9-2021_corrected_links11172021.pdf) | **MS Outlook:**   - [Introduction to the Outlook Calendar - Microsoft Support](https://support.microsoft.com/en-us/office/introduction-to-the-outlook-calendar-d94c5203-77c7-48ec-90a5-2e2bc10bd6f8) - [Microsoft Outlook (va.gov)](https://www.oit.va.gov/Services/TRM/ToolPage.aspx?tid=5684)   **VCM:**   - [Virtual Care Manager – Virtual Care Manager Community (blackboard.com)](https://vaots.blackboard.com/webapps/blackboard/execute/modulepage/view?course_id=_2540_1&cmp_tab_id=_3308_1&mode=view) - [Virtual Care Manager User Guide VCM 2.0 (va.gov)](https://mobile.va.gov/sites/default/files/user-manual-vcm.pdf)   **MS Teams:**   - [Veterans Affairs Teams Site - Home (sharepoint.com)](https://dvagov.sharepoint.com/sites/MSTeams) - [Microsoft Teams (va.gov)](https://www.oit.va.gov/Services/TRM/ToolPage.aspx?tid=14196) - [Teams Community of Practice (sharepoint.com)](https://dvagov.sharepoint.com/sites/MSTeams/LYNC/SitePages/Teams-Community-of-Practice(1).aspx) - [Bulletins - All Documents (sharepoint.com)](https://dvagov.sharepoint.com/sites/OITEPMOEPMDES/Projects/MSTeams/Bulletins/Forms/AllItems.aspx) |
|  | **Summary #25^3,4,5^:** Staff use several ways to send appointment links to the Veteran and healthcare staff at different time periods. Staff can use **VCM** to send an appointment link while on the **telephone** with the Veteran to have a link sent directly to their email. Alternatively, staff can use a **URL generator** to send a link via **VVC** for Veterans who are unable to navigate their email or locate the serial number [of VA issued device] in **CPRS + ROES** to create a static link in the **URL generator** to directly connect to the Veteran's VA issued device **[iPad]**. Healthcare staff can also use **MS Teams** to set up links for interdisciplinary appointments to coordinate the next provider in the queue to connect to the **VVC** link. | - [Scheduling -Telehealth – Scheduling - Telehealth Community (blackboard.com)](https://vaots.blackboard.com/webapps/blackboard/execute/modulepage/view?course_id=_2466_1&cmp_tab_id=_3176_1&mode=view) - [VVC Flip Book (blackboard.com)](https://vaots.blackboard.com/bbcswebdav/orgs/vaots_th_sched/thquickflipresource/index.html) - [Microsoft Office Fact Sheet_8-2020 Final__Correction_508_9-2021_corrected_links11172021.pdf (sharepoint.com)](https://dvagov.sharepoint.com/sites/vacovetsprivacy/vhapo/Documents/Guidebooks,%20Fact%20Sheets%20and%20Practice%20Briefs/Microsoft%20Office%20Fact%20Sheet_8-2020%20Final__Correction_508_9-2021_corrected_links11172021.pdf) - [VA iPad Loaner Program/Digital Divide (sharepoint.com)](https://dvagov.sharepoint.com/sites/SFC/Tele/VVC/SitePages/VA-iPad-Loaner-Program-Digital-Divide.aspx) | **VCM:**   - <https://mobile.va.gov/sites/default/files/user-manual-vcm.pdf> - <https://vaots.blackboard.com/bbcswebdav/xid-512168_1>   **VVC:**   - [VA Video Connect (VVC) – VA Video Connect (VVC... (blackboard.com)](https://vaots.blackboard.com/webapps/blackboard/execute/modulepage/view?course_id=_2591_1&cmp_tab_id=_3396_1&mode=view) - <https://vaots.blackboard.com/bbcswebdav/library/LibraryContent/VA%20Video%20Connect/VA%20Video%20Connect%20Web%202.X%20Provider%20Guide.pdf> - [VA Video Connect \| VA Mobile](https://mobile.va.gov/app/va-video-connect)   **URL Generator:**   - [URLs - VA.gov Design System](https://design.va.gov/content-style-guide/url-standards) - [vvc-url.xlsm (sharepoint.com)](https://dvagov.sharepoint.com/:x:/r/sites/VHA-Telehealth/_layouts/15/Doc.aspx?sourcedoc=%7BB74F5C10-C485-4FE2-A0FF-CEF98721F9A9%7D&file=vvc-url.xlsm&action=default&mobileredirect=true) - [Links - VA.gov Design System](https://design.va.gov/content-style-guide/links)   **CPRS + ROES:**   - [Remote Order Entry System (ROES) - VA Authentication Federation Infrastructure (VAAFI)](https://www.va.gov/EAUTH/ROES/index.asp)   **iPad**   - [Veteran Device Profiles and Setup \| Digital Divide Consult \| VHA Telehealth Services Intranet (va.gov)](https://vaww.telehealth.va.gov/technology/devices/veteran-devices.asp) - [VA Loaned Device App \| VHA Telehealth Services Intranet](https://vaww.telehealth.va.gov/technology/devices/vald.asp)   **MS Teams:**   - [Veterans Affairs Teams Site - Home (sharepoint.com)](https://dvagov.sharepoint.com/sites/MSTeams) - [Microsoft Teams (va.gov)](https://www.oit.va.gov/Services/TRM/ToolPage.aspx?tid=14196) - [Teams Community of Practice (sharepoint.com)](https://dvagov.sharepoint.com/sites/MSTeams/LYNC/SitePages/Teams-Community-of-Practice(1).aspx) - [Bulletins - All Documents (sharepoint.com)](https://dvagov.sharepoint.com/sites/OITEPMOEPMDES/Projects/MSTeams/Bulletins/Forms/AllItems.aspx) |
|  | **Summary #29^3^:** You can use **MS Teams** to support colleagues with troubleshooting technology issues. | - [Microsoft Office Fact Sheet_8-2020 Final__Correction_508_9-2021_corrected_links11172021.pdf (sharepoint.com)](https://dvagov.sharepoint.com/sites/vacovetsprivacy/vhapo/Documents/Guidebooks,%20Fact%20Sheets%20and%20Practice%20Briefs/Microsoft%20Office%20Fact%20Sheet_8-2020%20Final__Correction_508_9-2021_corrected_links11172021.pdf) | **MS Teams:**   - [Veterans Affairs Teams Site - Home (sharepoint.com)](https://dvagov.sharepoint.com/sites/MSTeams) - [Microsoft Teams (va.gov)](https://www.oit.va.gov/Services/TRM/ToolPage.aspx?tid=14196) - [Teams Community of Practice (sharepoint.com)](https://dvagov.sharepoint.com/sites/MSTeams/LYNC/SitePages/Teams-Community-of-Practice(1).aspx) - [Bulletins - All Documents (sharepoint.com)](https://dvagov.sharepoint.com/sites/OITEPMOEPMDES/Projects/MSTeams/Bulletins/Forms/AllItems.aspx) |
|  | **Summary #33^3^:** Healthcare staff can use **MS Teams** to alert clerk that Veteran has arrived for his or her appointment and can use the instant message feature to ask a clerk to update the Veterans records if necessary. | - [Microsoft Office Fact Sheet_8-2020 Final__Correction_508_9-2021_corrected_links11172021.pdf (sharepoint.com)](https://dvagov.sharepoint.com/sites/vacovetsprivacy/vhapo/Documents/Guidebooks,%20Fact%20Sheets%20and%20Practice%20Briefs/Microsoft%20Office%20Fact%20Sheet_8-2020%20Final__Correction_508_9-2021_corrected_links11172021.pdf) | **MS Teams:**   - [Veterans Affairs Teams Site - Home (sharepoint.com)](https://dvagov.sharepoint.com/sites/MSTeams) - [Microsoft Teams (va.gov)](https://www.oit.va.gov/Services/TRM/ToolPage.aspx?tid=14196) - [Teams Community of Practice (sharepoint.com)](https://dvagov.sharepoint.com/sites/MSTeams/LYNC/SitePages/Teams-Community-of-Practice(1).aspx) - [Bulletins - All Documents (sharepoint.com)](https://dvagov.sharepoint.com/sites/OITEPMOEPMDES/Projects/MSTeams/Bulletins/Forms/AllItems.aspx) |
| **3.3: Veteran Initiated Communication** | **Summary #18^1,2,3,4,5^:** Veteran shares treatment and post operation updates, or express concerns or any matter related to care via **SM (MHV)** or **telephone** to obtain provider’s feedback. Test results can be accessed with **MHV;** and images can be shared in **My VA Images** to facilitate these communications. | - [MHV_SM_HCT_User_Manual_August_2023.pdf (va.gov)](https://vaww.va.gov/MYHEALTHEVET/docs/secure_messaging/MHV_SM_HCT_User_Manual_August_2023.pdf) | **SM (MHV):**   - <https://www.myhealth.va.gov/mhv-portal-web/home> - [My HealtheVet Resources – My HealtheVet Resources](https://vaots.blackboard.com/webapps/blackboard/execute/modulepage/view?course_id=_3174_1&cmp_tab_id=_4591_1&mode=view) - [Secure Messaging Through My HealtheVet – My HealtheVet Product (va.gov)](https://vaww.va.gov/MYHEALTHEVET/Secure_Messaging.asp)   **My VA Images:**   - [My VA Images – My VA Images (MVAI) Community](https://vaots.blackboard.com/webapps/blackboard/execute/modulepage/view?course_id=_2480_1&cmp_tab_id=_3204_1&mode=view) - [Asynchronous SFT from Home My VA Images with VCM-Telehealth Operations Manual](https://learn-us-gov-west-1-prod-fleet01-xythos.s3.us-gov-west-1.amazonaws.com/612eabb1bae6a/1829967?response-cache-control=private%2C%20max-age%3D10800&response-content-disposition=inline%3B%20filename%2A%3DUTF-8%27%27Asynchronous%2520Store-and%2520Forward%2520Telehealth%2520from%2520Home%2520My%2520VA%2520Images%2520with%2520Virtual%2520Care%2520Manager-Telehealth%2520Operations%2520Manual.pdf&response-content-type=application%2Fpdf&X-Amz-Security-Token=FwoDYXdzEDUaDH1rNylD9mAF0StfNiLlBFq4%2FAB%2BwI5kYvt9bHMNvYg2VlTh%2FltGRCoNM8dkUJJ29R3WpD6yt63ricgawRC9BIUZ3irUUBUf2%2FS0IG2hKBT%2BfC7KcV3r2vqZaWx5FfxDrjjOnqbVOiaKrmavoDr6tJlgQkCIq58vfNaJNqkeq10gAJQI9w7JdUSA64FSHHa7iZvoqsGo2OuomNkGjipun9kj7roHgpkfn8S3BBTKSHZpumd45kst%2BmB49a6%2F8qGsTSurvmSLC6C%2FmT3p23ojNEge14v1vugYyLLGUyLBxSeCexWMFLqogjzSSuD7PClhDV8W%2BUuRERB24DZ%2FqvWmXxF4iU0yGEF5MsIOaDhv04Zujq9nqTDZ0C42VmOsjkh0UxvNC71GgvHaSxtwKtPiLK1oG3Bcw6c8jIun7R3arcDIhedtRWJ%2Bnhb6isufurcQQSg1fK1bxeAaSpVfmv7HDDZ4OC3kYEdWIi1QiFV%2BpjoMS1Yk34s%2BjOUmPekS5A7SJBcZ7CPB4m18QHP5Wetw7i7ViraucdZmPY9luRR%2BEEeFUUsjz7iyGz0nvlzk8nlt3K%2Ba%2BMIuN3C83sbAfVKhkiCa5rXNCPr%2FASOo5CsxYYA5PgGj7zfzXRmNXNp%2BQ%2BCUbex%2FoGoTgFFfvFGPoTmFdi9A2Z3Xe7UHhrPbS596orhLliKOUt6XbtZePHMvESMi4tpqGY%2FsOUnenpTvjq8VNmHrYA6AiFITrpnK33vcE7ItXfV%2By9cHRD2XYiAI21gFSvYRjOeUqSmCn%2B3yRkMekkJFJzCJqrnne1M05RJqnwv4Ft0xUGLWBR6vrKrbTlbwYv9DgNko4KKKvQYyQUE5H5GmFw5Qsy3czXLBLXizhkX%2BrErWuz2ulpgSvhlVic%2FpxwWPgiA1S%2FHb3nB2IY8A9fQ8ZU0px0%2BkZeuzjuX4&X-Amz-Algorithm=AWS4-HMAC-SHA256&X-Amz-Date=20250204T223000Z&X-Amz-SignedHeaders=host&X-Amz-Expires=10800&X-Amz-Credential=ASIAUMDY6U44W7SUOGKQ%2F20250204%2Fus-gov-west-1%2Fs3%2Faws4_request&X-Amz-Signature=6206807578f4b07356beaeec6883ee06963f1a45e24ad62cd623a033de76add4) - [My VA Images \| VHA Telehealth Services Intranet](https://vaww.telehealth.va.gov/pgm/mvai/index.asp) - [My VA Images FAQs](https://learn-us-gov-west-1-prod-fleet01-xythos.s3.us-gov-west-1.amazonaws.com/612eabb1bae6a/1856363?response-cache-control=private%2C%20max-age%3D10800&response-content-disposition=inline%3B%20filename%2A%3DUTF-8%27%27MY%2520VA%2520Images%2520FAQ.pdf&response-content-type=application%2Fpdf&X-Amz-Security-Token=FwoDYXdzEHoaDJwdXJkLIHpjrnQu5yLlBHtX8fdIkynj5PZ%2BhxNhtOTvvyF2aYPxjVDtfyGeOmpxKTtzQtq5SCBc4u9FlyqED%2BcALPCek66akU9ZIo1GnPqcKEm%2BEppo5bwAxrMJSMzamLt%2FJeFwMo1PABaa4VBC3iZ66cMtTH%2FXevw4aPkra8RAOvE7KFzw6OxwOj8RMtulHoldeEsjumq0d4P2ZVxbdRuzdeb5QXZ5NGA9gEVOwhjh87GVtdbQds1GnCfT%2FCMdoKpyJOdnkupE1qSvcNUHo1MLlfF2T%2F4ssMLkgCnocE4U0lGmNJjLB667YX7uNTOEi%2F3FwLf5SmApj0cY323lQFtSfbU2LCLAYbdpy7Q8zxl1e1LPCaaI65W1B5qon26fkSDjmmnxd2BcoWlZfnno5o1MlipzsvLDLMq9VIHHmIPYpK03Dw9JWvVfPs2wTtsg7Jk%2FM0cVaWMSQMWmmhzsj7CCF2ITl4nFm7WZiCpDYmzkYXZTfWki4S0ebRnSGwIOaN9Pis5efhd48spnjMc3SyN2szoOpT0XLWBcdLj6BDDf0WuLoGT2yrmuXPFcRu96s6ywfAgt6d86CEjFxyHvW9B4kJ4toHnhNittnEczTsEkVXshm3xvhakSW0MWVqiBMRkOmydfyzhIOxtZZLWONN5d%2BJ2HEc3D1SLTzrafeTUo2%2BeQ18JcjYipgCpTNYIGGLig5BIvhRHvT2ncl3yQ3feygb%2F9sdQXK8W1jwCFMdKtCBDqOwf%2FThKW1D2EMiIvbkJAdJjKXgYtwXi5WeZCEqfRzSTmeQnTqZUjlVIrkdj24dQsgISP7QW35vj4DmDDrKjqpQIo%2BMSZvQYyQc2S6lfGnf%2FriFqA7Xpw43f9s2X9R2qPbw%2BC5AzzECMmEtCfaCAHo3TQCanEwdV0HZTWYPW4oN6y12EuQ%2BCrvaMA&X-Amz-Algorithm=AWS4-HMAC-SHA256&X-Amz-Date=20250207T193000Z&X-Amz-SignedHeaders=host&X-Amz-Expires=10800&X-Amz-Credential=ASIAUMDY6U442RGZC5OF%2F20250207%2Fus-gov-west-1%2Fs3%2Faws4_request&X-Amz-Signature=c7922ac5ea532bac22f0df6ad4133dba7a9f91ada75638b282c3604047dc305d) |
| **3.4: Provider-Initiated Communication** | **Summary #14^1,3,5^:** Provider follows up with Veteran via **SM (MHV), MS Outlook,** or **telephone** to get updates from the Veteran, assess progress after treatment plan, confirm device was received, and share education material. Communication with the Veteran can be done via **SM (MHV)** or **MS Outlook.** | - [MHV_SM_HCT_User_Manual_August_2023.pdf (va.gov)](https://vaww.va.gov/MYHEALTHEVET/docs/secure_messaging/MHV_SM_HCT_User_Manual_August_2023.pdf) | **SM (MHV):**   - [My HealtheVet Resources – My HealtheVet Resources](https://vaots.blackboard.com/webapps/blackboard/execute/modulepage/view?course_id=_3174_1&cmp_tab_id=_4591_1&mode=view) - [Secure Messaging Through My HealtheVet – My HealtheVet Product (va.gov)](https://vaww.va.gov/MYHEALTHEVET/Secure_Messaging.asp)   **MS Outlook:**   - [Microsoft Outlook (va.gov)](https://www.oit.va.gov/Services/TRM/ToolPage.aspx?tid=5684) |
| **3.5 Adaptive Communication with Veteran** | **Summary #1^1,5^:** When experiencing connectivity issues or other technical problems during **VVC** appointment/session, Provider or dedicated IT team member supplement their communication with the Veteran with a phone call using **Doximity** or **Cisco Jabber** to continue the session or troubleshoot the problem. | - [Connected Device Support - VHA Telehealth Services Intranet (va.gov)](https://vaww.telehealth.va.gov/technology/devices/cds.asp) | **Cisco Jabber:**   - [https://www.oit.va.gov/Services/TRM/TRMRedirectPage.aspx?type=W^&tid=9777^](https://www.oit.va.gov/Services/TRM/TRMRedirectPage.aspx?type=W%5e&tid=9777%5e%20) - [Cisco Jabber for Windows - Cisco](https://www.cisco.com/c/en/us/support/unified-communications/jabber-windows/series.html)   **Doximity:**   - [https://www.doximity.com/?_c=bWFya2V0aW5nOjphZHdvcmRzOjpkb3hpbWl0eV8yMDE3MDg%3D.bsY2OiP1dMj6Q2iFHU6UNh8d4Xw%3D&return_to=https%3A%2F%2Fwww.doximity.com%2Fnewsfeed%3Futm_source%3Dgoogle%26utm_campaign%3Dmarketing_adwords&utm_source=bing&utm_campaign=marketing_adwords&campaignid=376136971&adgroupid=1225955646554449&adid=&msclkid=3c9471951f2b11e6867c134572d9c6c7](https://www.doximity.com/?_c=bWFya2V0aW5nOjphZHdvcmRzOjpkb3hpbWl0eV8yMDE3MDg%3D.bsY2OiP1dMj6Q2iFHU6UNh8d4Xw%3D&return_to=https%3A%2F%2Fwww.doximity.com%2Fnewsfeed%3Futm_source%3Dgoogle%26utm_campaign%3Dmarketing_adwords&utm_source=bing&utm_campaign=marketing_adwords&campaignid=376136971&adgroupid=1225955646554449&adid=&msclkid=3c9471951f2b11e6867c134572d9c6c7%20) - <https://mobile.va.gov/app/doximity>   **VVC:**   - <https://vaots.blackboard.com/bbcswebdav/library/LibraryContent/VA%20Video%20Connect/VA%20Video%20Connect%20Web%202.X%20Provider%20Guide.pdf> - [VA Video Connect \| VA Mobile](https://mobile.va.gov/app/va-video-connect) |
|  | **Summary #6^4^:** While in a remote environment, you can use **VVC** to see Veterans in their home environment and view the Veteran's refrigerator to incorporate Whole Health components (i.e., nutrition) during annual evaluations. With Veteran consent, non-Veterans such as caregivers can participate in the appointment*.* | - <https://vaww.telehealth.va.gov/pgm/vvc/during/peripherals.asp> | **VVC:**   - <https://vaots.blackboard.com/bbcswebdav/library/LibraryContent/VA%20Video%20Connect/VA%20Video%20Connect%20Web%202.X%20Provider%20Guide.pdf> - [VA Video Connect \| VA Mobile](https://mobile.va.gov/app/va-video-connect) - [VA Video Connect (VVC) – VA Video Connect (VVC... (blackboard.com)](https://vaots.blackboard.com/webapps/blackboard/execute/modulepage/view?course_id=_2591_1&cmp_tab_id=_3396_1&mode=view) |
|  | **Summary #8^5^:** You can use the **Live Whole Health app** or **SM (MHV)** to send a **Personal Health Inventory Questionnaire** to a Veteran. **MS Outlook** (do not reply) can be used in lieu of **SM (MHV)** if Veteran doesn't have an account. | - [Live Whole Health \| VA Mobile](https://mobile.va.gov/app/live-whole-health) - [10-773_PHI_May2020.pdf (va.gov)](https://www.va.gov/WHOLEHEALTH/docs/10-773_PHI_May2020.pdf) | **SM (MHV):**   - [My HealtheVet Resources – My HealtheVet Resources](https://vaots.blackboard.com/webapps/blackboard/execute/modulepage/view?course_id=_3174_1&cmp_tab_id=_4591_1&mode=view) - [Secure Messaging Through My HealtheVet – My HealtheVet Product (va.gov)](https://vaww.va.gov/MYHEALTHEVET/Secure_Messaging.asp)   **MS Outlook:**   - [Create and send email in Outlook - Microsoft Support](https://support.microsoft.com/en-us/office/create-and-send-email-in-outlook-19c32deb-08b6-4f90-a211-02bc5f77f360) |
|  | **Summary #15^3^: Direct texting** via an appropriate texting platform, or **SM (MHV)** provides asynchronous communication for non-verbal patients. These are appropriate forms of communication as an alternative to verbal communication. | - [Asynchronous Store and Forward Telehealth – Asynchronous... (blackboard.com)](https://vaots.blackboard.com/webapps/blackboard/execute/modulepage/view?course_id=_2439_1&cmp_tab_id=_3136_1&mode=view) | **Direct Texting:**   - [DirectMessagingCCPFactSheet.pdf (va.gov)](https://www.va.gov/VHIE/docs/DirectMessagingCCPFactSheet.pdf)   **SM (MHV):**   - [My HealtheVet Resources – My HealtheVet Resources](https://vaots.blackboard.com/webapps/blackboard/execute/modulepage/view?course_id=_3174_1&cmp_tab_id=_4591_1&mode=view) - [Secure Messaging Through My HealtheVet - My HealtheVet Product (va.gov)](https://vaww.va.gov/MYHEALTHEVET/Secure_Messaging.asp) |
| **3.6: Preparation for Communication with Veterans & Caregivers** | **Summary #17^5^:** Dedicated IT member prepares contact information through **CPRS** (from face sheet) and other forms/features needed for the virtual communication with the Veteran (such as **ROES**) before communicating with the Veteran. | - [cprssetup.pdf (va.gov)](https://www.va.gov/vdl/documents/Clinical/Comp_Patient_Recrd_Sys_(CPRS)/cprssetup.pdf) | **CPRS + ROES:**   - <https://www.va.gov/vdl/documents/Clinical/Remote_Order_Entry_Sys_(ROES)/rmpf3_0um.pdf?msclkid=>[2eb7cc0bd15211ec98d209e073e362de](https://www.va.gov/vdl/documents/Clinical/Remote_Order_Entry_Sys_(ROES)/rmpf3_0um.pdf?msclkid=2eb7cc0bd15211ec98d209e073e362de) - [Remote Order Entry System (ROES) - VA Authentication Federation Infrastructure (VAAFI)](https://www.va.gov/EAUTH/ROES/index.asp) |
| **3.7: Facilitating Virtual Communication & Access For In-patient** | **Summary #31^2^:** Provider in in-patient setting gives the Veteran the necessary device such as **iPad/tablet** to communicate with family. | - [VA iPad Loaner Program/Digital Divide (sharepoint.com)](https://dvagov.sharepoint.com/sites/SFC/Tele/VVC/SitePages/VA-iPad-Loaner-Program-Digital-Divide.aspx) | **Mobile Health Practice Guide**   - [va-mobile-health-practice-guide.pdf](https://connectedcare.va.gov/sites/default/files/2021-10/va-mobile-health-practice-guide.pdf) |
|  | **Summary #48^2,3,5^:** Provider uses **Annie app, SM (MHV), VVC, WebEx, MS Outlook** (blind email or encrypted), and **direct texting** to send education materials to Veterans. Provider uses **Get Well Network (VistA)** to provide in-patient/Veteran education via in-room television. | - [Virtual Care Best Practices Community – Virtual Care ... (blackboard.com)](https://vaots.blackboard.com/webapps/blackboard/execute/modulepage/view?course_id=_3330_1&cmp_tab_id=_5023_1&mode=view) | **Annie for Providers:**   - [Annie For Clinicians \| VA Mobile](https://mobile.va.gov/app/annie-app-clinicians) - [Annie App – Annie App Community (blackboard.com)](https://vaots.blackboard.com/webapps/blackboard/execute/modulepage/view?course_id=_2548_1&cmp_tab_id=_3324_1&mode=view) - [Annie app for Clinicians: Session One – YouTube](https://www.youtube.com/watch?v=h0Qt10Oq8ss)   **SM (MHV):**   - [Secure Messaging Through My HealtheVet - My HealtheVet Product (va.gov)](https://vaww.va.gov/MYHEALTHEVET/Secure_Messaging.asp) - [Secure Messaging Attachments - My HealtheVet - My HealtheVet (va.gov)](https://www.myhealth.va.gov/mhv-portal-web/web/myhealthevet/secure-messaging-attachments)   **VVC:**   - [VA Video Connect (VVC) – VA Video Connect (VVC... (blackboard.com)](https://vaots.blackboard.com/webapps/blackboard/execute/modulepage/view?course_id=_2591_1&cmp_tab_id=_3396_1&mode=view) - [VA Video Connect \| VA Mobile](https://mobile.va.gov/app/va-video-connect)   **WebEx:**   - [Approved Video Technologies \| Following the Pandemic \| VHA Telehealth Services Intranet (va.gov)](https://vaww.telehealth.va.gov/technology/alt/post-pandemic.asp) - <https://www.oit.va.gov/Services/TRM/ToolPage.aspx?tid=13521>   **MS Outlook:**   - <https://www.oit.va.gov/Services/TRM/ToolPage.aspx?tid=5684>   **Get Well Network:**   - <https://www.oit.va.gov/Services/TRM/ToolPage.aspx?tid=8065#:~:text=GetWellNetwork%20is%20an%20interactive%20patient%20communication%20system%20that,tasks%20such%20as%20diet%20selection%20and%20pain%20management> |
| **4.Patient Care Delivery** | | | |
| **4.1 Check-In & Triage** | **Summary #32^1,5^:** Healthcare staff can monitor Veteran check-in when in person or virtually for their scheduled appointment. Veteran can check-in using stand-alone self-service kiosks when in person. Healthcare staff can use **VCM** to locate the links for a virtual check-in of a Veteran. | - [https://www.va.gov/healthbenefits/vps/VPS_brochure_english.pdf](https://www.va.gov/healthbenefits/vps/VPS_brochure_english.pdf%20) - [Virtual Care Manager – Virtual Care Manager Community (blackboard.com)](https://vaots.blackboard.com/webapps/blackboard/execute/modulepage/view?course_id=_2540_1&cmp_tab_id=_3308_1&mode=view) | **VCM:**   - <https://vaots.blackboard.com/bbcswebdav/library/LibraryContent/Virtual%20Care%20Manager/Virtual%20Care%20Manager%20Scheduling%20Guide.pdf> - <https://mobile.va.gov/sites/default/files/user-manual-vcm.pdf> |
|  | **Summary #33^3^:** Healthcare staff can use **MS Teams** to alert clerk that Veteran has arrived for his or her appointment and can use the instant message feature to ask a clerk to update the Veterans records if necessary. | - [Microsoft Office Fact Sheet_8-2020 Final__Correction_508_9-2021_corrected_links11172021.pdf (sharepoint.com)](https://dvagov.sharepoint.com/sites/vacovetsprivacy/vhapo/Documents/Guidebooks,%20Fact%20Sheets%20and%20Practice%20Briefs/Microsoft%20Office%20Fact%20Sheet_8-2020%20Final__Correction_508_9-2021_corrected_links11172021.pdf) | **MS Teams:**   - [Veterans Affairs Teams Site - Home (sharepoint.com)](https://dvagov.sharepoint.com/sites/MSTeams) - [Microsoft Teams (va.gov)](https://www.oit.va.gov/Services/TRM/ToolPage.aspx?tid=14196) - [Teams Community of Practice (sharepoint.com)](https://dvagov.sharepoint.com/sites/MSTeams/LYNC/SitePages/Teams-Community-of-Practice(1).aspx) - [Bulletins - All Documents (sharepoint.com)](https://dvagov.sharepoint.com/sites/OITEPMOEPMDES/Projects/MSTeams/Bulletins/Forms/AllItems.aspx) |
| **4.2 Take History** | **Summary #7^1,3^:** Providers can use**VVC, VCM, MS Outlook**, and **Virtual Tool Rx app** to conduct virtual assessment, provide treatment, and make recommendations when taking history, performing an exam, or across all timeframes on the healthcare continuum. For example, a provider can send a Veteran exercise to perform at home using **MS Outlook** or **VCM** at any time across the healthcare continuum. | - [OCC Telehealth Manual (blackboard.com)](https://vaots.blackboard.com/bbcswebdav/library/LibraryContent/Telehealth%20Foundations-Business-Administration/th_manual/th_manual/TH-manual.html) | **VVC:**   - <https://www.tn.gov/content/dam/tn/veteranservices/learning/vso-tools/general-health-care/Video%20Connect%20Web%20User%20Guide%20for%20Veterans.pdf> - [VA Video Connect (VVC) – VA Video Connect (VVC... (blackboard.com)](https://vaots.blackboard.com/webapps/blackboard/execute/modulepage/view?course_id=_2591_1&cmp_tab_id=_3396_1&mode=view) - [VA Video Connect \| VA Mobile](https://mobile.va.gov/app/va-video-connect)   **VCM:**   - [Virtual Care Manager – Virtual Care Manager Community (blackboard.com)](https://vaots.blackboard.com/webapps/blackboard/execute/modulepage/view?course_id=_2540_1&cmp_tab_id=_3308_1&mode=view) - <https://mobile.va.gov/sites/default/files/user-manual-vcm.pdf> - [Virtual Care Manager \| VA Mobile](https://mobile.va.gov/app/virtual-care-manager)   **MS Outlook:**   - [Microsoft Outlook (va.gov)](https://www.oit.va.gov/Services/TRM/ToolPage.aspx?tid=5684)   **Virtual tool Rx:**   - <https://connectedcare.va.gov/sites/default/files/2022-10/VA-Virtual-Health-Toolkit-Prescription-Pad.pdf> |
|  | **Summary #12^1,3,5^: VCM** can be used to schedule a medical appointment with any Veteran, even if at a different clinic through the anywhere-to-anywhere directive. You can use **WebEx** or **VVC** to conduct a remote appointment with a Veteran to address weight, medical history, and presenting complaints. While using **VVC,** you can also access **CPRS** to review Veteran history, which can be kept open during the encounter with the Veteran. | - <https://telehealth.va.gov/> - [Scheduling -Telehealth – Scheduling - Telehealth Community (blackboard.com)](https://vaots.blackboard.com/webapps/blackboard/execute/modulepage/view?course_id=_2466_1&cmp_tab_id=_3176_1&mode=view) | **WebEx:**   - [Approved Video Technologies \| Following the Pandemic \| VHA Telehealth Services Intranet (va.gov)](https://vaww.telehealth.va.gov/technology/alt/post-pandemic.asp)   **VVC:**   - [VA Video Connect (VVC) – VA Video Connect (VVC... (blackboard.com)](https://vaots.blackboard.com/webapps/blackboard/execute/modulepage/view?course_id=_2591_1&cmp_tab_id=_3396_1&mode=view) - [https://vaots.blackboard.com/bbcswebdav/library/LibraryContent/VA Video Connect/VVC Instructional Videos/Provider-to-Scheduler Handoff in VVC video/Provider to Scheduler Handoff Video.html](https://vaots.blackboard.com/bbcswebdav/library/LibraryContent/VA%20Video%20Connect/VVC%20Instructional%20Videos/Provider-to-Scheduler%20Handoff%20in%20VVC%20video/Provider%20to%20Scheduler%20Handoff%20Video.html) - [VA Video Connect \| VA Mobile](https://mobile.va.gov/app/va-video-connect)   **VCM:**   - [Virtual Care Manager – Virtual Care Manager Community (blackboard.com)](https://vaots.blackboard.com/webapps/blackboard/execute/modulepage/view?course_id=_2540_1&cmp_tab_id=_3308_1&mode=view) - [Virtual Care Manager User Guide VCM 2.0 (va.gov)](https://mobile.va.gov/sites/default/files/user-manual-vcm.pdf)   **CPRS:**   - [CPRS Technical Manual (va.gov)](https://www.va.gov/vdl/documents/Clinical/Comp_Patient_Recrd_Sys_(CPRS)/cprslmtm.pdf)   [CPRS Session 1 Getting Started](https://www.youtube.com/watch?v=y52UtvS95F0) |
| **4.3 Conduct Assessment** | **Summary #5^4^:** You can use a **virtual remote stethoscope**with **VVC** and a headset to listen to a Veterans heart and lungs. The Veteran will send a link from **VVC** to allow the provider access and connect the device remotely to perform the activity. | - [Asynchronous Store and Forward Telehealth – Asynchronous... (blackboard.com)](https://vaots.blackboard.com/webapps/blackboard/execute/modulepage/view?course_id=_2439_1&cmp_tab_id=_3136_1) - <https://connectedcare.va.gov/about/outreach-toolkit> - [VA Video Connect (VVC) – VA Video Connect (VVC... (blackboard.com)](https://vaots.blackboard.com/webapps/blackboard/execute/modulepage/view?course_id=_2591_1&cmp_tab_id=_3396_1&mode=view) | **VVC:**   - <https://vaots.blackboard.com/bbcswebdav/library/LibraryContent/VA%20Video%20Connect/VA%20Video%20Connect%20Web%202.X%20Provider%20Guide.pdf> - [VA Video Connect \| VA Mobile](https://mobile.va.gov/app/va-video-connect)   **Virtual remote stethoscope:**   - <https://vaww.telehealth.va.gov/pgm/vvc/during/peripherals.asp> - [vaots.blackboard.com/bbcswebdav/library/Recycle Bin/Technology Videos/EKO Stethoscope Instructional Video/VVC and EKP Stethoscope Instructional VIdeo.html](https://vaots.blackboard.com/bbcswebdav/library/Recycle%20Bin/Technology%20Videos/EKO%20Stethoscope%20Instructional%20Video/VVC%20and%20EKP%20Stethoscope%20Instructional%20VIdeo.html) |
|  | **Summary #6^4^:** While in a remote environment, you can use **VVC** to see Veterans in their home environment and view the Veteran's refrigerator to incorporate Whole Health components (i.e., nutrition) during annual evaluations. With Veteran consent, non-Veterans such as caregivers can participate in the appointment. | - <https://vaww.telehealth.va.gov/pgm/vvc/during/peripherals.asp> | **VVC:**   - <https://vaots.blackboard.com/bbcswebdav/library/LibraryContent/VA%20Video%20Connect/VA%20Video%20Connect%20Web%202.X%20Provider%20Guide.pdf> - [VA Video Connect \| VA Mobile](https://mobile.va.gov/app/va-video-connect) - [VA Video Connect (VVC) – VA Video Connect (VVC... (blackboard.com)](https://vaots.blackboard.com/webapps/blackboard/execute/modulepage/view?course_id=_2591_1&cmp_tab_id=_3396_1&mode=view) |
|  | **Summary #7^1,3^:** Providers can use**VVC, VCM, MS Outlook**, and **Virtual Tool Rx app** to conduct virtual assessment, provide treatment, and make recommendations when taking history, performing an exam, or across all timeframes on the healthcare continuum. For example, a provider can send a Veteran exercise to perform at home using **MS Outlook** or **VCM** at any time across the healthcare continuum. | - [OCC Telehealth Manual (blackboard.com)](https://vaots.blackboard.com/bbcswebdav/library/LibraryContent/Telehealth%20Foundations-Business-Administration/th_manual/th_manual/TH-manual.html) | **VVC:**   - <https://www.tn.gov/content/dam/tn/veteranservices/learning/vso-tools/general-health-care/Video%20Connect%20Web%20User%20Guide%20for%20Veterans.pdf> - [VA Video Connect (VVC) – VA Video Connect (VVC... (blackboard.com)](https://vaots.blackboard.com/webapps/blackboard/execute/modulepage/view?course_id=_2591_1&cmp_tab_id=_3396_1&mode=view) - [VA Video Connect \| VA Mobile](https://mobile.va.gov/app/va-video-connect)   **VCM:**   - [Virtual Care Manager – Virtual Care Manager Community (blackboard.com)](https://vaots.blackboard.com/webapps/blackboard/execute/modulepage/view?course_id=_2540_1&cmp_tab_id=_3308_1&mode=view) - <https://mobile.va.gov/sites/default/files/user-manual-vcm.pdf> - [Virtual Care Manager \| VA Mobile](https://mobile.va.gov/app/virtual-care-manager)   **MS Outlook:**   - [Microsoft Outlook (va.gov)](https://www.oit.va.gov/Services/TRM/ToolPage.aspx?tid=5684)   **Virtual tool Rx:**   - <https://connectedcare.va.gov/sites/default/files/2022-10/VA-Virtual-Health-Toolkit-Prescription-Pad.pdf> |
|  | **Summary #12^1,3,5^:** …You can use **WebEx** or **VVC** to conduct a remote appointment with a Veteran to address weight, medical history, and presenting complaints. While using **VVC,** you can also access **CPRS** to review Veteran history, which can be kept open during the encounter with the Veteran. | - <https://telehealth.va.gov/> - [OCC Telehealth Manual (blackboard.com)](https://vaots.blackboard.com/bbcswebdav/library/LibraryContent/Telehealth%20Foundations-Business-Administration/th_manual/th_manual/TH-manual.html) | **WebEx:**   - [Approved Video Technologies \| Following the Pandemic \| VHA Telehealth Services Intranet (va.gov)](https://vaww.telehealth.va.gov/technology/alt/post-pandemic.asp)   **VVC:**   - [VA Video Connect (VVC) – VA Video Connect (VVC... (blackboard.com)](https://vaots.blackboard.com/webapps/blackboard/execute/modulepage/view?course_id=_2591_1&cmp_tab_id=_3396_1&mode=view) - [https://vaots.blackboard.com/bbcswebdav/library/LibraryContent/VA Video Connect/VVC Instructional Videos/Provider-to-Scheduler Handoff in VVC video/Provider to Scheduler Handoff Video.html](https://vaots.blackboard.com/bbcswebdav/library/LibraryContent/VA%20Video%20Connect/VVC%20Instructional%20Videos/Provider-to-Scheduler%20Handoff%20in%20VVC%20video/Provider%20to%20Scheduler%20Handoff%20Video.html) - [VA Video Connect \| VA Mobile](https://mobile.va.gov/app/va-video-connect)   **VCM:**   - [Virtual Care Manager – Virtual Care Manager Community (blackboard.com)](https://vaots.blackboard.com/webapps/blackboard/execute/modulepage/view?course_id=_2540_1&cmp_tab_id=_3308_1&mode=view) - [Virtual Care Manager User Guide VCM 2.0 (va.gov)](https://mobile.va.gov/sites/default/files/user-manual-vcm.pdf)   **CPRS:**   - [CPRS Technical Manual (va.gov)](https://www.va.gov/vdl/documents/Clinical/Comp_Patient_Recrd_Sys_(CPRS)/cprslmtm.pdf) - [CPRS Session 1 Getting Started](https://www.youtube.com/watch?v=y52UtvS95F0) |
|  | **Summary #34^3^:** You can use **VVC** to lock a therapy session with Veterans so there are no unnecessary intrusions from others. Healthcare staff can also use **VVC** to perform 3-way calling and invite family members into sessions with Veterans. | - [VA Video Connect (VVC) – VA Video Connect (VVC... (blackboard.com)](https://vaots.blackboard.com/webapps/blackboard/execute/modulepage/view?course_id=_2591_1&cmp_tab_id=_3396_1&mode=view) | **VVC:**   - <https://vaots.blackboard.com/bbcswebdav/library/LibraryContent/VA%20Video%20Connect/VA%20Video%20Connect%20Web%202.X%20Provider%20Guide.pdf> - [VA Video Connect \| VA Mobile](https://mobile.va.gov/app/va-video-connect) - [VA Video Connect (VVC) – VA Video Connect (VVC... (blackboard.com)](https://vaots.blackboard.com/webapps/blackboard/execute/modulepage/view?course_id=_2591_1&cmp_tab_id=_3396_1&mode=view) |
|  | **Summary #36^1,4,5^:** During the history and examination phase of treatment, staff can use **CPRS + ROES** to consult for device issuance. | - [Veteran Device Ordering Process \| Digital Divide Consult \| VHA Telehealth Services Intranet (va.gov)](https://vaww.telehealth.va.gov/technology/devices/vet-process.asp) | **CPRS:**   - [CPRS Technical Manual (va.gov)](https://www.va.gov/vdl/documents/Clinical/Comp_Patient_Recrd_Sys_(CPRS)/cprslmtm.pdf) - [CPRS Session 1 Getting Started](https://www.youtube.com/watch?v=y52UtvS95F0) - [Consult Tool Box Archives \| Office of Information and Technology (va.gov)](https://vaww.oit.va.gov/category/products/release-notes/ctb/)   **CPRS + ROES:**   - [Remote Order Entry System (ROES) - VA Authentication Federation Infrastructure (VAAFI)](https://www.va.gov/EAUTH/ROES/index.asp) |
|  | **Summary #38^3,4^:** When assessing the health of a Veteran, a significant amount of information needed is obtained by the Veteran. When conducting the appointment virtually, the Veteran can share their PGHD using a variety of VA mobile apps, such as **Annie app for Veterans.** | - <https://effectivehealthcare.ahrq.gov/sites/default/files/pdf/health-data-mapping-technicalbrief.pdf> - [Remote Patient Monitoring-Home Telehealth – Remote ... (blackboard.com)](https://vaots.blackboard.com/webapps/blackboard/execute/modulepage/view?course_id=_2686_1&cmp_tab_id=_3628_1&mode=view) - [Patient-Generated Health Data – Patient-Generated Health... (blackboard.com)](https://vaots.blackboard.com/webapps/blackboard/execute/modulepage/view?course_id=_2774_1&cmp_tab_id=_3774_1&mode=view) | **Annie App for Veterans:**   - [Annie App for Veterans \| VA Mobile](https://mobile.va.gov/app/annie-app-veterans)   **VA Mobile Apps:**   - [App Store \| VA Mobile](https://mobile.va.gov/appstore) |
|  | **Summary #39^4^:** You can use the **3D camera** to assist with tracking wound care. | - <https://effectivehealthcare.ahrq.gov/sites/default/files/pdf/health-data-mapping-technicalbrief.pdf> - [Patient-Generated Health Data – Patient-Generated Health... (blackboard.com)](https://vaots.blackboard.com/webapps/blackboard/execute/modulepage/view?course_id=_2774_1&cmp_tab_id=_3774_1&mode=view) | **3-D camera:**   - [Library: TeleWound Care – Blackboard Learn](https://vaots.blackboard.com/webapps/cmsmain/webui/library/LibraryContent/Outreach/TeleWound%20Care?action=frameset&subaction=view&uniq=-8bswn3) |
|  | **Summary #42^1,4,5^:** Monitored health information can be gathered and acquired of variety of ways. Patient Generated Health Data [PGHD] can be obtained asynchronously through equipment such as a wearable health tracking device **(fitBit, Apple Watch), Pulse oximeter, glucometer, digital scale, or blood pressure monitor.** Veterans can independently input monitored health indices using apps such as **CBT-i Coach app, Annie app for Veterans, and cardiac monitoring devices (i.e. Alivecor app, Zio patch).** For synchronous collection of monitored health data during an appointment, additional VHR, such as the **3D Camera** used for monitoring wounds, can be invaluable*.* | - [Remote Patient Monitoring-Home Telehealth – Remote ... (blackboard.com)](https://vaots.blackboard.com/webapps/blackboard/execute/modulepage/view?course_id=_2686_1&cmp_tab_id=_3628_1&mode=view) - [Remote Patient Monitoring - Home Telehealth Manual (blackboard.com)](https://vaots.blackboard.com/bbcswebdav/library/LibraryContent/RPM-HT/rpm_ht_manual/rpm-ht.html) - [Patient-Generated Health Data – Patient-Generated Health... (blackboard.com)](https://vaots.blackboard.com/webapps/blackboard/execute/modulepage/view?course_id=_2774_1&cmp_tab_id=_3774_1&mode=view) - [Virtual Care Manager - Patient Generated Data Guide](https://vaots.blackboard.com/bbcswebdav/xid-1218654_1) - [VA Video Connect (VVC) – VA Video Connect (VVC... (blackboard.com)](https://vaots.blackboard.com/webapps/blackboard/execute/modulepage/view?course_id=_2591_1&cmp_tab_id=_3396_1&mode=view) - [Technical Brief 38: Automated-Entry Patient-Generated Health Data for Chronic Conditions: The Evidence on Health Outcomes (ahrq.gov)](https://effectivehealthcare.ahrq.gov/sites/default/files/pdf/health-data-mapping-technicalbrief.pdf)   **Scoping Review for Sensor, Wearable, and Remote Patient Monitoring Competencies for Clinical Care & Training:**   - [Sensor, Wearable, and Remote Patient Monitoring Competencies for Clinical Care and Training: Scoping Review \| SpringerLink](https://link.springer.com/article/10.1007/s41347-020-00190-3) | **Pulse Oximeter:**   - [[1226744 (amazonaws.com)](https://learn-us-gov-west-1-prod-fleet01-xythos.s3.us-gov-west-1.amazonaws.com/612eabb1bae6a/1226744?response-cache-control=private%2C%20max-age%3D10800&response-content-disposition=inline%3B%20filename%2A%3DUTF-8%27%27VVC%2520Peripheral%2520Pairing%2520Guide%2520Pulse%2520Oximeter.pdf&response-content-type=application%2Fpdf&X-Amz-Security-Token=FwoDYXdzEI%2F%2F%2F%2F%2F%2F%2F%2F%2F%2F%2FwEaDFCZGOlKZL9Gl1wfpiLkBN7WZx3ou1W58W5Y4NGgAAWBaSP1%2BTxJm8ftHpasVKz8romt2kT7quGI7VYNBio9Wf6gdURlPqmT5E3Omi2zzGtPXSj35m0JxFT5CP74CYkTyPzC65W0K%2F4lh2c5CLAJwm2cYsC5n7%2F1zholgnOn8YCq%2BZbzTB2C1b1vOhX1aJGYGgcX%2FTqVWA%2B6%2FO%2Bc1aYN0VZfh5MnDAvnEBfq3XkcmDVBMOpyTvIjZ2SmegpCSEqV4sAdfsd0ODS5OW0FKayWsmhcov%2BAQFqyGK8vWgsbnr7crgok0ZDTroSVmQHIpyWI8cx6y8cwEMZM9mDn7jCHMERzquFB5enFwxwnqo8AfueUBHUwc1KQl2INZIzz4QJHnQjFi%2BEIsOgjugXTObG%2FksT1GeKzzTfKhPd7vKceMI8fvC0B75%2FKvLOs%2FGQ6Khw0aP%2F7AEY2QoP9rI3AxNs6ypjAAQIqUuYJBGpPCSeRaG8uZabYT8SOZvVWwlEtd36BvkfMzAVjPqZ%2F2D%2BV%2Bf9qaeZH312l9tnIpGiyYMB7ZQM8B%2FWJT0PKzLTE8ttYTkwmNGWhjM2H%2BDnFsJ%2Fy30wlEdd7m8RVzc8%2FoRcHz4MiE%2FK7JFvlv%2FbK0CjgNG%2BJgSB1DE%2FJ50aDkdg%2Bb58Quzvwj1KvfvmEjzMgtAlZW4ZIGNDOaFy8Ow112o4%2FtpL3LiipiWXkdhjXxPKBNdPb05DMRlwA4kNI%2BGEYYVQZ7wxJjFLVR0X7H%2FhyHf7m1zFd%2FeN%2BfGbzzNOAcpw8Bz7%2BcWhX%2F2r3bCFoc4qR4WOKz3kv932LSF%2FovLfmskTeEnbipLBfb1WWaCiujqysBjJB3da%2Flyr9JL1kJSsW0I7T7GhDu3D2L0TJ3eJEjSaBxuUJwn%2B8QXSaoXkXJX9AOcTsyxYvyKZ7tv%2Bfh0dUb%2B9pJ8I%3D&X-Amz-Algorithm=AWS4-HMAC-SHA256&X-Amz-Date=20231226T180000Z&X-Amz-SignedHeaders=host&X-Amz-Expires=10800&X-Amz-Credential=ASIAUMDY6U44UCHTYQE5%2F20231226%2Fus-gov-west-1%2Fs3%2Faws4_request&X-Amz-Signature=599e132c9cc637522de224d1589932a1ac272df37738908bf295965554a877d5)](https://learn-us-gov-west-1-prod-fleet01-xythos.s3.us-gov-west-1.amazonaws.com/612eabb1bae6a/1226744?response-cache-control=private%2C%20max-age%3D10800&response-content-disposition=inline%3B%20filename%2A%3DUTF-8%27%27VVC%2520Peripheral%2520Pairing%2520Guide%2520Pulse%2520Oximeter.pdf&response-content-type=application%2Fpdf&X-Amz-Security-Token=FwoDYXdzEI%2F%2F%2F%2F%2F%2F%2F%2F%2F%2F%2FwEaDFCZGOlKZL9Gl1wfpiLkBN7WZx3ou1W58W5Y4NGgAAWBaSP1%2BTxJm8ftHpasVKz8romt2kT7quGI7VYNBio9Wf6gdURlPqmT5E3Omi2zzGtPXSj35m0JxFT5CP74CYkTyPzC65W0K%2F4lh2c5CLAJwm2cYsC5n7%2F1zholgnOn8YCq%2BZbzTB2C1b1vOhX1aJGYGgcX%2FTqVWA%2B6%2FO%2Bc1aYN0VZfh5MnDAvnEBfq3XkcmDVBMOpyTvIjZ2SmegpCSEqV4sAdfsd0ODS5OW0FKayWsmhcov%2BAQFqyGK8vWgsbnr7crgok0ZDTroSVmQHIpyWI8cx6y8cwEMZM9mDn7jCHMERzquFB5enFwxwnqo8AfueUBHUwc1KQl2INZIzz4QJHnQjFi%2BEIsOgjugXTObG%2FksT1GeKzzTfKhPd7vKceMI8fvC0B75%2FKvLOs%2FGQ6Khw0aP%2F7AEY2QoP9rI3AxNs6ypjAAQIqUuYJBGpPCSeRaG8uZabYT8SOZvVWwlEtd36BvkfMzAVjPqZ%2F2D%2BV%2Bf9qaeZH312l9tnIpGiyYMB7ZQM8B%2FWJT0PKzLTE8ttYTkwmNGWhjM2H%2BDnFsJ%2Fy30wlEdd7m8RVzc8%2FoRcHz4MiE%2FK7JFvlv%2FbK0CjgNG%2BJgSB1DE%2FJ50aDkdg%2Bb58Quzvwj1KvfvmEjzMgtAlZW4ZIGNDOaFy8Ow112o4%2FtpL3LiipiWXkdhjXxPKBNdPb05DMRlwA4kNI%2BGEYYVQZ7wxJjFLVR0X7H%2FhyHf7m1zFd%2FeN%2BfGbzzNOAcpw8Bz7%2BcWhX%2F2r3bCFoc4qR4WOKz3kv932LSF%2FovLfmskTeEnbipLBfb1WWaCiujqysBjJB3da%2Flyr9JL1kJSsW0I7T7GhDu3D2L0TJ3eJEjSaBxuUJwn%2B8QXSaoXkXJX9AOcTsyxYvyKZ7tv%2Bfh0dUb%2B9pJ8I%3D&X-Amz-Algorithm=AWS4-HMAC-SHA256&X-Amz-Date=20231226T180000Z&X-Amz-SignedHeaders=host&X-Amz-Expires=10800&X-Amz-Credential=ASIAUMDY6U44UCHTYQE5%2F20231226%2Fus-gov-west-1%2Fs3%2Faws4_request&X-Amz-Signature=599e132c9cc637522de224d1589932a1ac272df37738908bf295965554a877d5)   **Cardiac monitoring device:**   - [KardiaMobile Card is Available for Veterans \| Kardia](https://www.kardia.com/gov)   **Sleep Clinic Resources:**   - [Sleep-Clinic Workflow](https://vaots.blackboard.com/bbcswebdav/xid-567502_1) - [Sleep Program – Sleep Program Community (blackboard.com)](https://vaots.blackboard.com/webapps/blackboard/execute/modulepage/view?course_id=_2554_1&cmp_tab_id=_3336_1&mode=view) - [Sleep Intranet Resource Center](https://vaww.telehealth.va.gov/clinic/tsleep/index.asp)   **FitBit/Apple Watch:**   - [Digital Health Innovations - VA-Fitbit-HowToParticipate-1P.pdf - All Documents (sharepoint.com)](https://dvagov.sharepoint.com/sites/VHAV1NECIE/DHI/Shared%20Documents/Forms/AllItems.aspx?id=%2Fsites%2FVHAV1NECIE%2FDHI%2FShared%20Documents%2FVA%2DFitbit%2DHowToParticipate%2D1P%2Epdf&parent=%2Fsites%2FVHAV1NECIE%2FDHI%2FShared%20Documents) - [Digital Health Innovations - Fitbit Sense - Getting Started Guide - Veterans.pdf - All Documents (sharepoint.com)](https://dvagov.sharepoint.com/sites/VHAV1NECIE/DHI/Shared%20Documents/Forms/AllItems.aspx?id=%2Fsites%2FVHAV1NECIE%2FDHI%2FShared%20Documents%2FFitbit%20Sense%20%2D%20Getting%20Started%20Guide%20%2D%20Veterans%2Epdf&parent=%2Fsites%2FVHAV1NECIE%2FDHI%2FShared%20Documents)   **Peripheral Devices:**   - [Peripheral Devices \| During the VA Video Connect Visit \| VHA Telehealth Services Intranet](https://vaww.telehealth.va.gov/pgm/vvc/during/peripherals.asp)   **Blood Pressure Monitor:**   - [569283 (amazonaws.com)](https://learn-us-gov-west-1-prod-fleet01-xythos.s3.us-gov-west-1.amazonaws.com/612eabb1bae6a/569283?response-cache-control=private%2C%20max-age%3D10800&response-content-disposition=inline%3B%20filename%2A%3DUTF-8%27%27VVC%2520Peripheral%2520Pairing%2520Guide%2520Blood%2520Pressure.pdf&response-content-type=application%2Fpdf&X-Amz-Security-Token=FwoDYXdzEKb%2F%2F%2F%2F%2F%2F%2F%2F%2F%2FwEaDNihyzG6BBJlm7g3RSLkBDWBa0eQrvjTRXFLZ%2BwSo0GkYtWskMLQ8zmO4mhKnKFaOD2ykn%2FDRPWlnm%2BGluvTUL9g2mmOnTQXkipUWWOsocG%2Fn3HXRgOsLAHMoN%2B7ZepEzlYZhSwS1JDoOfrPIK6MsA%2BJ280pwu47T2YwJxmQlS6IO4Bu4%2F%2BF2%2BztY0oB67q%2Fihc2UB6FKI2mmaLFG0N4zALTvF42vDKLmj%2BtnRbB6VGYCiZi1stzI0ac4ePMSdvqewi6EvcGmpFE%2Bo4nXRD%2BtnHb2bEFulCmMNAh5gsKvytBYInE%2BkFXkOmiokGy%2FQYwnL%2FEntDtohbVWsLgxwjSQq5Iv%2BDA7MIRCWbNI7a6s9fu%2FUZVSYogvxu1AhD9I1HQoDpaamiey1ByYqbyIGzLFpLso0sB%2FPK9iOkjmA8e5%2BMZmbePeNyjZjh3YXYNMTYZa669ZIYame8cjRsaqlDAUnw39GHjhZAAsiJEmLbc69m%2FEvIaJaaeGNZnMnSx9T6QQAtE3WeEtaVGXoqYB8%2FkcSnHRTOW%2BUxXOU09XMd%2F5mOaX8xOrPRYalIPPii3d4JAaEJiiQm%2BOIW%2BHKerWLiEPPbJRjFLkcb83UBvRQ%2F0i7qY6%2FW56%2FcMNsIRgwtTvSOoHY%2FkECJoAKl8eQMrTgIjY9zZd6hDhSFk5p%2BK3mzZOCIRS2RdJYtQ6K5IZemc81pwdKNj%2FBEia8GoM0EYHpR004qtnatpY3BYi%2Fa9H0n6xhnafw3F9ggO7TNo%2FO2Hs3qlfkmivXYkqX2nXwEE2NzCOzIN979RlCP9P8OcFWkdige75XPfVK7pO5Og8%2Bi5xfMcF3l4iCjikLGsBjJBGvkKrrZkpHMqODM2DzouIsUJlFAminwrt%2BO0qWhVDKzWcW7wFlG7xv4EaAu9Kpe380FXITNhz97VbGWfKUnzR%2Bo%3D&X-Amz-Algorithm=AWS4-HMAC-SHA256&X-Amz-Date=20231227T163000Z&X-Amz-SignedHeaders=host&X-Amz-Expires=10800&X-Amz-Credential=ASIAUMDY6U44RUDMCFOA%2F20231227%2Fus-gov-west-1%2Fs3%2Faws4_request&X-Amz-Signature=0a5513cba3a39b2a5aa8519e38a85e1c020e63504500cb11abd0842ed86879ce)   **3-D camera:**   - [Library: TeleWound Care – Blackboard Learn](https://vaots.blackboard.com/webapps/cmsmain/webui/library/LibraryContent/Outreach/TeleWound%20Care?action=frameset&subaction=view&uniq=-8bswn3)   **Annie App for Veterans:**   - [Annie For Veterans \| VA Mobile](https://mobile.va.gov/app/annie-app-veterans)   **CBT-i Coach:**   - [CBT-i Coach \| VA Mobile](https://mobile.va.gov/app/cbt-i-coach)   **VA App Store:**   - [VA App Store \| VA Mobile](https://mobile.va.gov/appstore/) |
| **4.4 Provide Treatment & Recommendations** | **Summary #7^1,3^:** Providers can use**VVC, VCM, MS Outlook**, and **Virtual Tool Rx app** to conduct virtual assessment, provide treatment, and make recommendations when taking history, performing an exam, or across all timeframes on the healthcare continuum. For example, a provider can send a Veteran exercise to perform at home using **MS Outlook** or **VCM** at any time across the healthcare continuum. | - [OCC Telehealth Manual (blackboard.com)](https://vaots.blackboard.com/bbcswebdav/library/LibraryContent/Telehealth%20Foundations-Business-Administration/th_manual/th_manual/TH-manual.html) | **VVC:**   - <https://www.tn.gov/content/dam/tn/veteranservices/learning/vso-tools/general-health-care/Video%20Connect%20Web%20User%20Guide%20for%20Veterans.pdf> - [VA Video Connect (VVC) – VA Video Connect (VVC... (blackboard.com)](https://vaots.blackboard.com/webapps/blackboard/execute/modulepage/view?course_id=_2591_1&cmp_tab_id=_3396_1&mode=view) - [VA Video Connect \| VA Mobile](https://mobile.va.gov/app/va-video-connect)   **VCM:**   - [Virtual Care Manager – Virtual Care Manager Community (blackboard.com)](https://vaots.blackboard.com/webapps/blackboard/execute/modulepage/view?course_id=_2540_1&cmp_tab_id=_3308_1&mode=view) - <https://mobile.va.gov/sites/default/files/user-manual-vcm.pdf> - [Virtual Care Manager \| VA Mobile](https://mobile.va.gov/app/virtual-care-manager)   **MS Outlook:**   - [Microsoft Outlook (va.gov)](https://www.oit.va.gov/Services/TRM/ToolPage.aspx?tid=5684)   **Virtual tool Rx:**   - <https://connectedcare.va.gov/sites/default/files/2022-10/VA-Virtual-Health-Toolkit-Prescription-Pad.pdf> |
|  | **Summary #19^5^:** Provider can use apps such as **CBT-i-Coach app, PTSD Coach app, CPT Coach app** and **Insomnia Coach app** to use during post-appointment for providing treatment and to manage and track PGHD. | - [Patient-Generated Health Data – Patient-Generated Health... (blackboard.com)](https://vaots.blackboard.com/webapps/blackboard/execute/modulepage/view?course_id=_2774_1&cmp_tab_id=_3774_1&mode=view) | **VA App Store:**  [VA App Store \| VA Mobile](https://mobile.va.gov/appstore/)  **CBT-i (Cognitive Behavioral Therapy for Insomnia) Coach App:**   - [CBT-i Coach \| VA Mobile](https://mobile.va.gov/app/cbt-i-coach)   **PTSD (Post Traumatic Stress Disorder) Coach App:**   - [PTSD Coach \| VA Mobile](https://mobile.va.gov/app/ptsd-coach)   **CPT (Cognitive Processing Therapy) Coach App:**   - [CPT Coach \| VA Mobile](https://mobile.va.gov/app/cpt-coach)   **Insomnia Coach App:**   - [Insomnia Coach \| VA Mobile](https://mobile.va.gov/app/insomnia-coach) |
|  | **Summary #34^3^:** You can use **VVC** to lock a therapy session with Veterans so there are no unnecessary intrusions from others. Healthcare staff can also use **VVC** to perform 3-way calling and invite family members into sessions with Veterans. | - [VA Video Connect (VVC) – VA Video Connect (VVC... (blackboard.com)](https://vaots.blackboard.com/webapps/blackboard/execute/modulepage/view?course_id=_2591_1&cmp_tab_id=_3396_1&mode=view) | **VVC:**   - <https://vaots.blackboard.com/bbcswebdav/library/LibraryContent/VA%20Video%20Connect/VA%20Video%20Connect%20Web%202.X%20Provider%20Guide.pdf> - [VA Video Connect \| VA Mobile](https://mobile.va.gov/app/va-video-connect) |
| **4.5 Conducting Group Appointment*** | **Current Best Practice #35: VVC** is the preferred platform to conduct group appointments, where the provider can promote healthy lifestyle and encourage the use of **VVC.** | - [Synchronous Video Telehealth for Group Visits – ... (blackboard.com)](https://vaots.blackboard.com/webapps/blackboard/content/listContentEditable.jsp?content_id=_153105_1&course_id=_2688_1) | **VVC:**   - [VA Video Connect \| VA Mobile](https://mobile.va.gov/app/va-video-connect) - [VA Video Connect (VVC) – VA Video Connect (VVC... (blackboard.com)](https://vaots.blackboard.com/webapps/blackboard/execute/modulepage/view?course_id=_2591_1&cmp_tab_id=_3396_1&mode=view) |
| **4.6: Follow Up** | **Summary #14^1,3,5^:** Provider follows up with Veteran via **SM (MHV), MS Outlook**, or **telephone** to get updates from the Veteran, assess progress after treatment plan, confirm device was received, and share education material. Communication with the Veteran can be done via **SM (MHV)** or **MS Outlook.** | - [MHV_SM_HCT_User_Manual_August_2023.pdf (va.gov)](https://vaww.va.gov/MYHEALTHEVET/docs/secure_messaging/MHV_SM_HCT_User_Manual_August_2023.pdf) | **SM (MHV):**   - [My HealtheVet Resources – My HealtheVet Resources](https://vaots.blackboard.com/webapps/blackboard/execute/modulepage/view?course_id=_3174_1&cmp_tab_id=_4591_1&mode=view) - [Secure Messaging Through My HealtheVet - My HealtheVet Product (va.gov)](https://vaww.va.gov/MYHEALTHEVET/Secure_Messaging.asp)   **MS Outlook:**   - [Microsoft Outlook (va.gov)](https://www.oit.va.gov/Services/TRM/ToolPage.aspx?tid=5684) |
| **5. Laboratory and Diagnostic Tests Management** | | | |
| **5.1: Request Laboratory Tests or Imaging*** | **Current Best Practice #56:** Provider uses **CPRS** to put in orders such as labs, consults and procedures. | - [CPRS Technical Manual (va.gov)](https://www.va.gov/vdl/documents/Clinical/Comp_Patient_Recrd_Sys_(CPRS)/cprslmtm.pdf) | **CPRS:**   - [cprssetup.pdf (va.gov)](https://www.va.gov/vdl/documents/Clinical/Comp_Patient_Recrd_Sys_(CPRS)/cprssetup.pdf) - [Consult Tool Box Archives \| Office of Information and Technology (va.gov)](https://vaww.oit.va.gov/category/products/release-notes/ctb/) |
| **5.2: Deliver Laboratory Tests or Imaging*** | **Current Best Practice #58:** Literature by Haun et al, published best practices in **SM (MHV)** for delivering labs, tests, and imaging **(My VA Images)** results. | - [Best Care Everywhere \| U.S. Government Bookstore (gpo.gov)](https://bookstore.gpo.gov/products/best-care-everywhere) - [Haun, J. N., Lind, J. D., Shimada, S. L., Martin, T. L., Gosline, R. M., Antinori, N., ... & Simon, S. R. (2014). Evaluating user experiences of the secure messaging tool on the Veterans Affairs’ patient portal system. *Journal of medical Internet research*, *16*(3), e2976.](https://www.jmir.org/2014/3/e75) - [Haun, J. N., Patel, N. R., Lind, J. D., & Antinori, N. (2015). Large-scale survey findings inform patients’ experiences in using secure messaging to engage in patient-provider communication and self-care management: a quantitative assessment. *Journal of medical Internet research*, *17*(12), e282.](https://www.jmir.org/2015/12/e282/) - [Haun, J. N., Lind, J. D., Shimada, S. L., & Simon, S. R. (2013). EVALUATING SECURE MESSAGING FROM THE VETERAN PERSPECTIVE: INFORMING THE ADOPTION AND SUSTAINED USE OF A PATIENT‐DRIVEN COMMUNICATION PLATFORM. *Annals of Anthropological Practice*, *37*(2), 57-74.](https://anthrosource.onlinelibrary.wiley.com/doi/abs/10.1111/napa.12029) - [Haun, J. N., Hathaway, W., Chavez, M., Antinori, N., Vetter, B., Miller, B. K., ... & Melillo, C. (2017). Clinical practice informs secure messaging benefits and best practices. *Applied Clinical Informatics*, *8*(04), 1003-1011.](https://www.thieme-connect.com/products/ejournals/html/10.4338/ACI-2017-05-RA-0088) - [Haun JN, Hathaway W, Chavez M, et al. Clinical Practice Informs Secure Messaging Benefits and Best Practices. *Appl Clin Inform*. 2017;8(4):1003-1011. doi:10.4338/ACI-2017-05-RA-0088](https://www.ncbi.nlm.nih.gov/pmc/articles/PMC5802310/) - [Haun, J. N., Chavez, M., Nazi, K., Antinori, N., Melillo, C., Cotner, B. A., ... & Noonan, A. (2017). Veterans’ preferences for exchanging information using veterans affairs health information technologies: focus group results and modeling simulations. *Journal of medical Internet research*, *19*(10), e359.](https://www.jmir.org/2017/10/e359/) | **SM (MHV):**   - [Secure Messaging Through My HealtheVet - My HealtheVet Product (va.gov)](https://vaww.va.gov/MYHEALTHEVET/Secure_Messaging.asp) - [MHV_Creating_Secure_Message_VA_Staff_QRG.pdf](https://vaww.va.gov/MYHEALTHEVET/docs/secure_messaging/MHV_Creating_Secure_Message_VA_Staff_QRG.pdf) - [VA Labs and Tests - My HealtheVet - My HealtheVet](https://www.myhealth.va.gov/faqs-labs-tests) - [VA Medical Images and Reports FAQs - My HealtheVet - My HealtheVet](https://www.myhealth.va.gov/what-are-va-medical-images-and-reports-faqs)   **My VA Images**   - [My VA Images – My VA Images (MVAI) Community](https://vaots.blackboard.com/webapps/blackboard/execute/modulepage/view?course_id=_2480_1&cmp_tab_id=_3204_1&mode=view) - [Asynchronous SFT from Home My VA Images with VCM-Telehealth Operations Manual](https://learn-us-gov-west-1-prod-fleet01-xythos.s3.us-gov-west-1.amazonaws.com/612eabb1bae6a/1829967?response-cache-control=private%2C%20max-age%3D10800&response-content-disposition=inline%3B%20filename%2A%3DUTF-8%27%27Asynchronous%2520Store-and%2520Forward%2520Telehealth%2520from%2520Home%2520My%2520VA%2520Images%2520with%2520Virtual%2520Care%2520Manager-Telehealth%2520Operations%2520Manual.pdf&response-content-type=application%2Fpdf&X-Amz-Security-Token=FwoDYXdzEDUaDH1rNylD9mAF0StfNiLlBFq4%2FAB%2BwI5kYvt9bHMNvYg2VlTh%2FltGRCoNM8dkUJJ29R3WpD6yt63ricgawRC9BIUZ3irUUBUf2%2FS0IG2hKBT%2BfC7KcV3r2vqZaWx5FfxDrjjOnqbVOiaKrmavoDr6tJlgQkCIq58vfNaJNqkeq10gAJQI9w7JdUSA64FSHHa7iZvoqsGo2OuomNkGjipun9kj7roHgpkfn8S3BBTKSHZpumd45kst%2BmB49a6%2F8qGsTSurvmSLC6C%2FmT3p23ojNEge14v1vugYyLLGUyLBxSeCexWMFLqogjzSSuD7PClhDV8W%2BUuRERB24DZ%2FqvWmXxF4iU0yGEF5MsIOaDhv04Zujq9nqTDZ0C42VmOsjkh0UxvNC71GgvHaSxtwKtPiLK1oG3Bcw6c8jIun7R3arcDIhedtRWJ%2Bnhb6isufurcQQSg1fK1bxeAaSpVfmv7HDDZ4OC3kYEdWIi1QiFV%2BpjoMS1Yk34s%2BjOUmPekS5A7SJBcZ7CPB4m18QHP5Wetw7i7ViraucdZmPY9luRR%2BEEeFUUsjz7iyGz0nvlzk8nlt3K%2Ba%2BMIuN3C83sbAfVKhkiCa5rXNCPr%2FASOo5CsxYYA5PgGj7zfzXRmNXNp%2BQ%2BCUbex%2FoGoTgFFfvFGPoTmFdi9A2Z3Xe7UHhrPbS596orhLliKOUt6XbtZePHMvESMi4tpqGY%2FsOUnenpTvjq8VNmHrYA6AiFITrpnK33vcE7ItXfV%2By9cHRD2XYiAI21gFSvYRjOeUqSmCn%2B3yRkMekkJFJzCJqrnne1M05RJqnwv4Ft0xUGLWBR6vrKrbTlbwYv9DgNko4KKKvQYyQUE5H5GmFw5Qsy3czXLBLXizhkX%2BrErWuz2ulpgSvhlVic%2FpxwWPgiA1S%2FHb3nB2IY8A9fQ8ZU0px0%2BkZeuzjuX4&X-Amz-Algorithm=AWS4-HMAC-SHA256&X-Amz-Date=20250204T223000Z&X-Amz-SignedHeaders=host&X-Amz-Expires=10800&X-Amz-Credential=ASIAUMDY6U44W7SUOGKQ%2F20250204%2Fus-gov-west-1%2Fs3%2Faws4_request&X-Amz-Signature=6206807578f4b07356beaeec6883ee06963f1a45e24ad62cd623a033de76add4) - [My VA Images \| VHA Telehealth Services Intranet](https://vaww.telehealth.va.gov/pgm/mvai/index.asp) - [My VA Images FAQs](https://learn-us-gov-west-1-prod-fleet01-xythos.s3.us-gov-west-1.amazonaws.com/612eabb1bae6a/1856363?response-cache-control=private%2C%20max-age%3D10800&response-content-disposition=inline%3B%20filename%2A%3DUTF-8%27%27MY%2520VA%2520Images%2520FAQ.pdf&response-content-type=application%2Fpdf&X-Amz-Security-Token=FwoDYXdzEHoaDJwdXJkLIHpjrnQu5yLlBHtX8fdIkynj5PZ%2BhxNhtOTvvyF2aYPxjVDtfyGeOmpxKTtzQtq5SCBc4u9FlyqED%2BcALPCek66akU9ZIo1GnPqcKEm%2BEppo5bwAxrMJSMzamLt%2FJeFwMo1PABaa4VBC3iZ66cMtTH%2FXevw4aPkra8RAOvE7KFzw6OxwOj8RMtulHoldeEsjumq0d4P2ZVxbdRuzdeb5QXZ5NGA9gEVOwhjh87GVtdbQds1GnCfT%2FCMdoKpyJOdnkupE1qSvcNUHo1MLlfF2T%2F4ssMLkgCnocE4U0lGmNJjLB667YX7uNTOEi%2F3FwLf5SmApj0cY323lQFtSfbU2LCLAYbdpy7Q8zxl1e1LPCaaI65W1B5qon26fkSDjmmnxd2BcoWlZfnno5o1MlipzsvLDLMq9VIHHmIPYpK03Dw9JWvVfPs2wTtsg7Jk%2FM0cVaWMSQMWmmhzsj7CCF2ITl4nFm7WZiCpDYmzkYXZTfWki4S0ebRnSGwIOaN9Pis5efhd48spnjMc3SyN2szoOpT0XLWBcdLj6BDDf0WuLoGT2yrmuXPFcRu96s6ywfAgt6d86CEjFxyHvW9B4kJ4toHnhNittnEczTsEkVXshm3xvhakSW0MWVqiBMRkOmydfyzhIOxtZZLWONN5d%2BJ2HEc3D1SLTzrafeTUo2%2BeQ18JcjYipgCpTNYIGGLig5BIvhRHvT2ncl3yQ3feygb%2F9sdQXK8W1jwCFMdKtCBDqOwf%2FThKW1D2EMiIvbkJAdJjKXgYtwXi5WeZCEqfRzSTmeQnTqZUjlVIrkdj24dQsgISP7QW35vj4DmDDrKjqpQIo%2BMSZvQYyQc2S6lfGnf%2FriFqA7Xpw43f9s2X9R2qPbw%2BC5AzzECMmEtCfaCAHo3TQCanEwdV0HZTWYPW4oN6y12EuQ%2BCrvaMA&X-Amz-Algorithm=AWS4-HMAC-SHA256&X-Amz-Date=20250207T193000Z&X-Amz-SignedHeaders=host&X-Amz-Expires=10800&X-Amz-Credential=ASIAUMDY6U442RGZC5OF%2F20250207%2Fus-gov-west-1%2Fs3%2Faws4_request&X-Amz-Signature=c7922ac5ea532bac22f0df6ad4133dba7a9f91ada75638b282c3604047dc305d) |
| **6. Referral Management** | | | |
| **6.1 Device Issuance** | **Summary #36^1,4,5^:** During the history and examination phase of treatment, staff can use **CPRS + ROES** to consult for device issuance. | - [Veteran Device Ordering Process \| Digital Divide Consult \| VHA Telehealth Services Intranet (va.gov)](https://vaww.telehealth.va.gov/technology/devices/vet-process.asp) | **CPRS:**   - [CPRS Technical Manual (va.gov)](https://www.va.gov/vdl/documents/Clinical/Comp_Patient_Recrd_Sys_(CPRS)/cprslmtm.pdf) - [CPRS Session 1 Getting Started](https://www.youtube.com/watch?v=y52UtvS95F0)   **CPRS + ROES:**   - [Remote Order Entry System (ROES) - VA Authentication Federation Infrastructure (VAAFI)](https://www.va.gov/EAUTH/ROES/index.asp) |
| **6.2 Provider Referrals for Care*** | **Current Best Practice #53:** Healthcare staff can use **MS Excel** to receive and manage referrals, for example, with Veterans who are interested in coming to WH Immersion. | - [Microsoft Excel (va.gov)](https://www.oit.va.gov/Services/TRM/ToolPage.aspx?tid=5612) | **MS Excel:**   - <https://dvagov.sharepoint.com/sites/O365CL/SitePages/CustomLearningViewer.aspx?subcategory=85c785c2-c873-4483-9728-64a367fee3f1> |
| **6.3 Referrals for Technology*** | **Current Best Practice #54:** A Digital Divide Consult can be placed to order and issue VA-loaned devices. Any VA staff member who intends to manage the Veteran’s care via **VVC** (or their designee) can place the Digital Divide Consult. The consult should be placed for Veterans who would benefit from **VVC** into their home but lack an affordable or quality internet connection or a video-capable device. The Veteran must also meet one or more of the clinical criteria listed on the consult. If the provider determines the Veteran’s care will be enhanced by using peripheral devices, these are ordered/identified via the consult. Peripherals include blood pressure monitors, weight scales, stethoscopes, pulse oximeters, and thermometers. The social worker places the Video Device Order Consult after determining the Veteran has qualified for a VA loaned device. The consult alerts the local ROES Coordinator of the devices (e.g., tablet, peripherals) that the Veteran requires to place the order in **ROES.** | - [Peripheral Devices \| During the VA Video Connect Visit \| VHA Telehealth Services Intranet](https://vaww.telehealth.va.gov/pgm/vvc/during/peripherals.asp) - [Veteran Device Ordering Process \| Digital Divide Consult \| VHA Telehealth Services Intranet (va.gov)](https://vaww.telehealth.va.gov/technology/devices/vet-process.asp) - [Veteran Device Profiles and Setup \| Digital Divide Consult \| VHA Telehealth Services Intranet (va.gov)](https://vaww.telehealth.va.gov/technology/devices/veteran-devices.asp) | **VVC:**   - [VA Video Connect (VVC) – VA Video Connect (VVC... (blackboard.com)](https://vaots.blackboard.com/webapps/blackboard/execute/modulepage/view?course_id=_2591_1&cmp_tab_id=_3396_1&mode=view) - [VA Video Connect \| VA Mobile](https://mobile.va.gov/app/va-video-connect)   **ROES**   - [Remote Order Entry System (ROES) - VA Authentication Federation Infrastructure (VAAFI)](https://www.va.gov/EAUTH/ROES/index.asp) |
| **7. Data Collection Management** | | | |
| **7.1 Program Feedback & Evaluation*** | **Current Best Practice #55:** Collaborations with Central Office indicate that **Qualtrics** and **REDCap** can be used for surveys between staff and/or Veterans externally outside VA firewall at home, as it is approved for storage of PHI and PII and has public-facing survey capability. | - [Center for Mobile Apps Research Resources & Services (CMARRS) - Center for Innovation to Implementation (Ci2i)](https://www.ci2i.research.va.gov/CMARRS/) - [SurveyGuidance.pdf (va.gov)](https://www.research.va.gov/resources/SurveyGuidance.pdf) | **Qualtrics:**   - [VHA ORD Qualtrics Portal - Home (sharepoint.com)](https://dvagov.sharepoint.com/sites/VHAORPPE/Qualtrics) - [Non-VHA Qualtrics Training Resources (sharepoint.com)](https://dvagov.sharepoint.com/sites/VHAORPPE/Qualtrics/SitePages/Qualtrics-Training-Resources.aspx)   **REDCap:**   - [VA REDCap (sharepoint.com)](https://dvagov.sharepoint.com/sites/VHAPugResearch/RRG/SitePages/VA-REDCap.aspx) - [FAQs page](https://gcc02.safelinks.protection.outlook.com/?url=https%3A%2F%2Fvaww.virec.research.va.gov%2FREDCap%2FFAQs.htm&data=05%7C01%7C%7C07ce688d7b33417a89b008db9368bdc0%7Ce95f1b23abaf45ee821db7ab251ab3bf%7C0%7C0%7C638265850289259457%7CUnknown%7CTWFpbGZsb3d8eyJWIjoiMC4wLjAwMDAiLCJQIjoiV2luMzIiLCJBTiI6Ik1haWwiLCJXVCI6Mn0%3D%7C3000%7C%7C%7C&sdata=vNHnE0LBqb3KsSmHrE%2Ff6z3OUiQLUxAbdwhHyjS8Dhg%3D&reserved=0) - [VA REDCap Ticket](https://gcc02.safelinks.protection.outlook.com/?url=https%3A%2F%2Fvaredcap.rcp.vaec.va.gov%2Fredcap%2Fsurveys%2F%3Fs%3DW7Y9ACRKKX&data=05%7C01%7C%7C07ce688d7b33417a89b008db9368bdc0%7Ce95f1b23abaf45ee821db7ab251ab3bf%7C0%7C0%7C638265850289259457%7CUnknown%7CTWFpbGZsb3d8eyJWIjoiMC4wLjAwMDAiLCJQIjoiV2luMzIiLCJBTiI6Ik1haWwiLCJXVCI6Mn0%3D%7C3000%7C%7C%7C&sdata=3U391REq4GWCbQDlLOphZiPgjJH5xGKgCynBdUkXK5U%3D&reserved=0) - [VA REDCap Guidelines](https://gcc02.safelinks.protection.outlook.com/?url=https%3A%2F%2Fvaww.virec.research.va.gov%2FREDCap%2FGuidelines.htm&data=05%7C01%7C%7C07ce688d7b33417a89b008db9368bdc0%7Ce95f1b23abaf45ee821db7ab251ab3bf%7C0%7C0%7C638265850289259457%7CUnknown%7CTWFpbGZsb3d8eyJWIjoiMC4wLjAwMDAiLCJQIjoiV2luMzIiLCJBTiI6Ik1haWwiLCJXVCI6Mn0%3D%7C3000%7C%7C%7C&sdata=tk0%2FGurUCpMU2BZ7QDF8h0IusJy2unbzuO0K8B1xT8g%3D&reserved=0) - [VIReCREDCapSupport@va.gov](mailto:VIReCREDCapSupport@va.gov) |
|  | **Summary #37^1,2,4,5^:** Providers can monitor health indices and identify abnormalities by reviewing the **Care Assessment Need (CAN) Risk Assessment (CPRS)** and other health data uploaded into the patients’ charts through the telehealth program. Additional health indices can be monitored when the patient shares PGHD during a healthcare visit that they collected or documented on various apps or devices, but it is important to promote the use of select VA mobile apps and/or preferred devices with the Veteran. | - [WebVRAM – WebVRAM Community (blackboard.com)](https://vaots.blackboard.com/webapps/blackboard/execute/modulepage/view?course_id=_2481_1&cmp_tab_id=_3205_1&mode=view) - [Remote Patient Monitoring - Home Telehealth Manual (blackboard.com)](https://vaots.blackboard.com/bbcswebdav/library/LibraryContent/RPM-HT/rpm_ht_manual/rpm-ht.html) - [Sensor, Wearable, and Remote Patient Monitoring Competencies for Clinical Care and Training: Scoping Review (amazonaws.com)](https://learn-us-gov-west-1-prod-fleet01-xythos.s3.us-gov-west-1.amazonaws.com/612eabb1bae6a/622493?response-cache-control=private%2C%20max-age%3D10800&response-content-disposition=inline%3B%20filename%2A%3DUTF-8%27%27Clinical%2520Competency%2520for%2520Use%2520of%2520Sensors%252C%2520Wearables%252C%2520Remote%2520Patient%2520Monitoring.pdf&response-content-type=application%2Fpdf&X-Amz-Security-Token=FwoDYXdzEJH%2F%2F%2F%2F%2F%2F%2F%2F%2F%2FwEaDN%2B8TpaTNSUJTFiqPyLlBA%2B5kzFuLAS0LEIGwvmZrYYdj%2B6H9i%2B%2FPmr3uyF5vTgI9kYOJy1u%2FgC9bNYQbDez59m%2FjoaI9sTaWRcV3aB29nqxcJv9Q8c%2BdHxQFNRpSp4KGJhrw%2FOFatJan2idYrhoU0FPCcyNMLe7dSMDH3EY4VIR3grGJoTlOXXCSMIvaks0zsBSdm1xPT169C6JOVnvA9Nai50%2BL4U77MXt8gVUbnCKRlT%2BPpui1RKtK7HXVY%2BUvmmYTQs1g1rLtMVqxfhfMAFx9zn903tYfygZtV4m5HPI7OZcSY0DLYIftnHCO2G5%2Bo%2Fb3d%2FO285zDwLArwvAU48Cx7CEOOZxZlCWEbg0RtyRtDBMuIzYa8ln0CmVnW8wHK686yZ8oqWE%2BvS5fKD5lKx4aIaGUQSiXBW0YfrRSbL0ATldILZNqj8GdUdTx3PgSY9puuwWeuK3TGiZYAG1mCTub4fvhXJsTCD3AeRGGIAOE80hRNWVpZ%2FmWoi6UHCvrj1AMWYS33KEg4MDe70hVlhipMyr0OYC6h48iuWUokajZkggNNFYTxLwlBzGkRtJCBL0GALdXIX7rvigarOrm6lbCHMgaqS%2FO%2F0V%2Bt2fjJ84CMs%2FymP6gbhCtgE0PSUWdD%2FVOf%2FV2SwsFEsb2p9wOv9MRoYevhGDodZGq1HU5jUUTSfkWqjUXYRBhyQoOxvoNoZdYfnDDoME1LCBcqU4lU3ifYSknek0MQFLI5tspXwun7oXIZSIye16V%2Bu%2FexvmwfBbqbjn96Gx0Sp9j87F%2F%2BbwOzzz7TivuX%2BIje5pE%2FYPWsdsJRG9KmDYXDbhmYGk2Ay9e8QoiMbvogYyQafPFJsmsF407MKRClAq4l1m7rRICxHftwW0hu4mkPdci677%2B3Nqgk4QrIm5vXDbV0MAVwx2tREjY4mMcywv%2Bheb&X-Amz-Algorithm=AWS4-HMAC-SHA256&X-Amz-Date=20230510T193000Z&X-Amz-SignedHeaders=host&X-Amz-Expires=10800&X-Amz-Credential=ASIAUMDY6U444MOL72AQ%2F20230510%2Fus-gov-west-1%2Fs3%2Faws4_request&X-Amz-Signature=e02a75f342a825c18b499f1f5969f8af962964b836bf714a56cc5f7991f7bed5) - [Technical Brief 38: Automated-Entry Patient-Generated Health Data for Chronic Conditions: The Evidence on Health Outcomes (ahrq.gov)](https://effectivehealthcare.ahrq.gov/sites/default/files/pdf/health-data-mapping-technicalbrief.pdf) | **CAN:**   - [Study gauges VA providers' views on predictive-analytics tool that assesses patient risk](https://www.research.va.gov/currents/1119-Researchers-study-VA-patient-data-tool-that-assesses-probability-of-hospitalization-and-death.cfm) - [713-notes.pdf (va.gov)](https://www.hsrd.research.va.gov/for_researchers/cyber_seminars/archives/713-notes.pdf)   **Peripherals:**   - <https://vaww.telehealth.va.gov/pgm/vvc/during/peripherals.asp> |
| **7.2 Documenting Data Summaries** | **Summary #37^1,2,4,5^:** Providers can monitor health indices and identify abnormalities by reviewing the **Care Assessment Need (CAN) Risk Assessment (CPRS)** and other health data uploaded into the patients’ charts through the telehealth program. Additional health indices can be monitored when the patient shares PGHD during a healthcare visit that they collected or documented on various apps or devices, but it is important to promote the use of select VA mobile apps and/or preferred devices with the Veteran. | - [WebVRAM – WebVRAM Community (blackboard.com)](https://vaots.blackboard.com/webapps/blackboard/execute/modulepage/view?course_id=_2481_1&cmp_tab_id=_3205_1&mode=view) - [Remote Patient Monitoring - Home Telehealth Manual (blackboard.com)](https://vaots.blackboard.com/bbcswebdav/library/LibraryContent/RPM-HT/rpm_ht_manual/rpm-ht.html) - [Technical Brief 38: Automated-Entry Patient-Generated Health Data for Chronic Conditions: The Evidence on Health Outcomes (ahrq.gov)](https://effectivehealthcare.ahrq.gov/sites/default/files/pdf/health-data-mapping-technicalbrief.pdf) | **CAN:**   - [Study gauges VA providers' views on predictive-analytics tool that assesses patient risk](https://www.research.va.gov/currents/1119-Researchers-study-VA-patient-data-tool-that-assesses-probability-of-hospitalization-and-death.cfm) - [713-notes.pdf (va.gov)](https://www.hsrd.research.va.gov/for_researchers/cyber_seminars/archives/713-notes.pdf)   **Peripherals:**   - <https://vaww.telehealth.va.gov/pgm/vvc/during/peripherals.asp> |
| **7.3 Outcome Measures** | **Summary #8^5^:** You can use the **Live Whole Health app** or **SM (MHV)** to send a **Personal Health Inventory Questionnaire** to a Veteran. **MS Outlook** (do not reply) can be used in lieu of **SM (MHV)** if Veteran doesn't have an account. | - [Live Whole Health \| VA Mobile](https://mobile.va.gov/app/live-whole-health) - [10-773_PHI_May2020.pdf (va.gov)](https://www.va.gov/WHOLEHEALTH/docs/10-773_PHI_May2020.pdf) | **SM (MHV):**   - S[My HealtheVet Resources – My HealtheVet Resources](https://vaots.blackboard.com/webapps/blackboard/execute/modulepage/view?course_id=_3174_1&cmp_tab_id=_4591_1&mode=view) - [Secure Messaging Through My HealtheVet - My HealtheVet Product (va.gov)](https://vaww.va.gov/MYHEALTHEVET/Secure_Messaging.asp)   **MS Outlook:**   - [Create and send email in Outlook - Microsoft Support](https://support.microsoft.com/en-us/office/create-and-send-email-in-outlook-19c32deb-08b6-4f90-a211-02bc5f77f360) |
|  | **Summary #18^1,2,3,4,5^:** Veteran shares treatment and post operation updates, or express concerns or any matter related to care via **SM (MHV)** or **telephone** to obtain provider’s feedback. Test results can be accessed with **MHV;** and images can be shared in **My VA Images** to facilitate these communications. | - [MHV_SM_HCT_User_Manual_August_2023.pdf (va.gov)](https://vaww.va.gov/MYHEALTHEVET/docs/secure_messaging/MHV_SM_HCT_User_Manual_August_2023.pdf) | **SM (MHV):**   - <https://www.myhealth.va.gov/mhv-portal-web/home> - [My HealtheVet Resources – My HealtheVet Resources](https://vaots.blackboard.com/webapps/blackboard/execute/modulepage/view?course_id=_3174_1&cmp_tab_id=_4591_1&mode=view) - [Secure Messaging Through My HealtheVet – My HealtheVet Product (va.gov)](https://vaww.va.gov/MYHEALTHEVET/Secure_Messaging.asp)   **My VA Images:**   - [My VA Images – My VA Images (MVAI) Community](https://vaots.blackboard.com/webapps/blackboard/execute/modulepage/view?course_id=_2480_1&cmp_tab_id=_3204_1&mode=view) - [Asynchronous SFT from Home My VA Images with VCM-Telehealth Operations Manual](https://learn-us-gov-west-1-prod-fleet01-xythos.s3.us-gov-west-1.amazonaws.com/612eabb1bae6a/1829967?response-cache-control=private%2C%20max-age%3D10800&response-content-disposition=inline%3B%20filename%2A%3DUTF-8%27%27Asynchronous%2520Store-and%2520Forward%2520Telehealth%2520from%2520Home%2520My%2520VA%2520Images%2520with%2520Virtual%2520Care%2520Manager-Telehealth%2520Operations%2520Manual.pdf&response-content-type=application%2Fpdf&X-Amz-Security-Token=FwoDYXdzEDUaDH1rNylD9mAF0StfNiLlBFq4%2FAB%2BwI5kYvt9bHMNvYg2VlTh%2FltGRCoNM8dkUJJ29R3WpD6yt63ricgawRC9BIUZ3irUUBUf2%2FS0IG2hKBT%2BfC7KcV3r2vqZaWx5FfxDrjjOnqbVOiaKrmavoDr6tJlgQkCIq58vfNaJNqkeq10gAJQI9w7JdUSA64FSHHa7iZvoqsGo2OuomNkGjipun9kj7roHgpkfn8S3BBTKSHZpumd45kst%2BmB49a6%2F8qGsTSurvmSLC6C%2FmT3p23ojNEge14v1vugYyLLGUyLBxSeCexWMFLqogjzSSuD7PClhDV8W%2BUuRERB24DZ%2FqvWmXxF4iU0yGEF5MsIOaDhv04Zujq9nqTDZ0C42VmOsjkh0UxvNC71GgvHaSxtwKtPiLK1oG3Bcw6c8jIun7R3arcDIhedtRWJ%2Bnhb6isufurcQQSg1fK1bxeAaSpVfmv7HDDZ4OC3kYEdWIi1QiFV%2BpjoMS1Yk34s%2BjOUmPekS5A7SJBcZ7CPB4m18QHP5Wetw7i7ViraucdZmPY9luRR%2BEEeFUUsjz7iyGz0nvlzk8nlt3K%2Ba%2BMIuN3C83sbAfVKhkiCa5rXNCPr%2FASOo5CsxYYA5PgGj7zfzXRmNXNp%2BQ%2BCUbex%2FoGoTgFFfvFGPoTmFdi9A2Z3Xe7UHhrPbS596orhLliKOUt6XbtZePHMvESMi4tpqGY%2FsOUnenpTvjq8VNmHrYA6AiFITrpnK33vcE7ItXfV%2By9cHRD2XYiAI21gFSvYRjOeUqSmCn%2B3yRkMekkJFJzCJqrnne1M05RJqnwv4Ft0xUGLWBR6vrKrbTlbwYv9DgNko4KKKvQYyQUE5H5GmFw5Qsy3czXLBLXizhkX%2BrErWuz2ulpgSvhlVic%2FpxwWPgiA1S%2FHb3nB2IY8A9fQ8ZU0px0%2BkZeuzjuX4&X-Amz-Algorithm=AWS4-HMAC-SHA256&X-Amz-Date=20250204T223000Z&X-Amz-SignedHeaders=host&X-Amz-Expires=10800&X-Amz-Credential=ASIAUMDY6U44W7SUOGKQ%2F20250204%2Fus-gov-west-1%2Fs3%2Faws4_request&X-Amz-Signature=6206807578f4b07356beaeec6883ee06963f1a45e24ad62cd623a033de76add4) - [My VA Images \| VHA Telehealth Services Intranet](https://vaww.telehealth.va.gov/pgm/mvai/index.asp) - [My VA Images FAQs](https://learn-us-gov-west-1-prod-fleet01-xythos.s3.us-gov-west-1.amazonaws.com/612eabb1bae6a/1856363?response-cache-control=private%2C%20max-age%3D10800&response-content-disposition=inline%3B%20filename%2A%3DUTF-8%27%27MY%2520VA%2520Images%2520FAQ.pdf&response-content-type=application%2Fpdf&X-Amz-Security-Token=FwoDYXdzEHoaDJwdXJkLIHpjrnQu5yLlBHtX8fdIkynj5PZ%2BhxNhtOTvvyF2aYPxjVDtfyGeOmpxKTtzQtq5SCBc4u9FlyqED%2BcALPCek66akU9ZIo1GnPqcKEm%2BEppo5bwAxrMJSMzamLt%2FJeFwMo1PABaa4VBC3iZ66cMtTH%2FXevw4aPkra8RAOvE7KFzw6OxwOj8RMtulHoldeEsjumq0d4P2ZVxbdRuzdeb5QXZ5NGA9gEVOwhjh87GVtdbQds1GnCfT%2FCMdoKpyJOdnkupE1qSvcNUHo1MLlfF2T%2F4ssMLkgCnocE4U0lGmNJjLB667YX7uNTOEi%2F3FwLf5SmApj0cY323lQFtSfbU2LCLAYbdpy7Q8zxl1e1LPCaaI65W1B5qon26fkSDjmmnxd2BcoWlZfnno5o1MlipzsvLDLMq9VIHHmIPYpK03Dw9JWvVfPs2wTtsg7Jk%2FM0cVaWMSQMWmmhzsj7CCF2ITl4nFm7WZiCpDYmzkYXZTfWki4S0ebRnSGwIOaN9Pis5efhd48spnjMc3SyN2szoOpT0XLWBcdLj6BDDf0WuLoGT2yrmuXPFcRu96s6ywfAgt6d86CEjFxyHvW9B4kJ4toHnhNittnEczTsEkVXshm3xvhakSW0MWVqiBMRkOmydfyzhIOxtZZLWONN5d%2BJ2HEc3D1SLTzrafeTUo2%2BeQ18JcjYipgCpTNYIGGLig5BIvhRHvT2ncl3yQ3feygb%2F9sdQXK8W1jwCFMdKtCBDqOwf%2FThKW1D2EMiIvbkJAdJjKXgYtwXi5WeZCEqfRzSTmeQnTqZUjlVIrkdj24dQsgISP7QW35vj4DmDDrKjqpQIo%2BMSZvQYyQc2S6lfGnf%2FriFqA7Xpw43f9s2X9R2qPbw%2BC5AzzECMmEtCfaCAHo3TQCanEwdV0HZTWYPW4oN6y12EuQ%2BCrvaMA&X-Amz-Algorithm=AWS4-HMAC-SHA256&X-Amz-Date=20250207T193000Z&X-Amz-SignedHeaders=host&X-Amz-Expires=10800&X-Amz-Credential=ASIAUMDY6U442RGZC5OF%2F20250207%2Fus-gov-west-1%2Fs3%2Faws4_request&X-Amz-Signature=c7922ac5ea532bac22f0df6ad4133dba7a9f91ada75638b282c3604047dc305d) |
|  | **Summary #40^5^: Qualtrics** will send an autogenerated email to a healthcare team member if a Veteran endorses suicidal ideation. Collaborations with Central Office indicate that **Qualtrics** and **REDCap** can be used for surveys between staff and/or Veterans externally outside VA firewall at home, as it is approved for storage of PHI and PII and has public-facing survey capability. | - [Center for Mobile Apps Research Resources & Services (CMARRS) - Center for Innovation to Implementation (Ci2i)](https://www.ci2i.research.va.gov/CMARRS/) - [SurveyGuidance.pdf (va.gov)](https://www.research.va.gov/resources/SurveyGuidance.pdf) | **Qualtrics:**   - [VHA ORD Qualtrics Portal - Home (sharepoint.com)](https://dvagov.sharepoint.com/sites/VHAORPPE/Qualtrics) - [Non-VHA Qualtrics Training Resources (sharepoint.com)](https://dvagov.sharepoint.com/sites/VHAORPPE/Qualtrics/SitePages/Qualtrics-Training-Resources.aspx)   **REDCap:**   - [VA REDCap (sharepoint.com)](https://dvagov.sharepoint.com/sites/VHAPugResearch/RRG/SitePages/VA-REDCap.aspx) - [FAQs page](https://gcc02.safelinks.protection.outlook.com/?url=https%3A%2F%2Fvaww.virec.research.va.gov%2FREDCap%2FFAQs.htm&data=05%7C01%7C%7C07ce688d7b33417a89b008db9368bdc0%7Ce95f1b23abaf45ee821db7ab251ab3bf%7C0%7C0%7C638265850289259457%7CUnknown%7CTWFpbGZsb3d8eyJWIjoiMC4wLjAwMDAiLCJQIjoiV2luMzIiLCJBTiI6Ik1haWwiLCJXVCI6Mn0%3D%7C3000%7C%7C%7C&sdata=vNHnE0LBqb3KsSmHrE%2Ff6z3OUiQLUxAbdwhHyjS8Dhg%3D&reserved=0) - [VA REDCap Ticket](https://gcc02.safelinks.protection.outlook.com/?url=https%3A%2F%2Fvaredcap.rcp.vaec.va.gov%2Fredcap%2Fsurveys%2F%3Fs%3DW7Y9ACRKKX&data=05%7C01%7C%7C07ce688d7b33417a89b008db9368bdc0%7Ce95f1b23abaf45ee821db7ab251ab3bf%7C0%7C0%7C638265850289259457%7CUnknown%7CTWFpbGZsb3d8eyJWIjoiMC4wLjAwMDAiLCJQIjoiV2luMzIiLCJBTiI6Ik1haWwiLCJXVCI6Mn0%3D%7C3000%7C%7C%7C&sdata=3U391REq4GWCbQDlLOphZiPgjJH5xGKgCynBdUkXK5U%3D&reserved=0) - [VA REDCap Guidelines](https://gcc02.safelinks.protection.outlook.com/?url=https%3A%2F%2Fvaww.virec.research.va.gov%2FREDCap%2FGuidelines.htm&data=05%7C01%7C%7C07ce688d7b33417a89b008db9368bdc0%7Ce95f1b23abaf45ee821db7ab251ab3bf%7C0%7C0%7C638265850289259457%7CUnknown%7CTWFpbGZsb3d8eyJWIjoiMC4wLjAwMDAiLCJQIjoiV2luMzIiLCJBTiI6Ik1haWwiLCJXVCI6Mn0%3D%7C3000%7C%7C%7C&sdata=tk0%2FGurUCpMU2BZ7QDF8h0IusJy2unbzuO0K8B1xT8g%3D&reserved=0) - [VIReCREDCapSupport@va.gov](mailto:VIReCREDCapSupport@va.gov) |
|  | **Summary #41:** You can use the **Care Assessment Need (CAN) Risk Assessment (CPRS)** to assess who has an elevated CAN score to group Veterans who fulfill that criteria. | - [713-notes.pdf (va.gov)](https://www.hsrd.research.va.gov/for_researchers/cyber_seminars/archives/713-notes.pdf) | **CPRS:**   - [CPRS Technical Manual (va.gov)](https://www.va.gov/vdl/documents/Clinical/Comp_Patient_Recrd_Sys_(CPRS)/cprslmtm.pdf)   **Care Assessment Need (CAN):**   - [Study gauges VA providers' views on predictive-analytics tool that assesses patient risk](https://www.research.va.gov/currents/1119-Researchers-study-VA-patient-data-tool-that-assesses-probability-of-hospitalization-and-death.cfm) |
|  | **Summary #42^1,4,5^:** Monitored health information can be gathered and acquired of variety of ways. Patient Generated Health Data [PGHD] can be obtained asynchronously through equipment such as a wearable health tracking device **(fitBit, Apple Watch), Pulse oximeter, glucometer, digital scale, or blood pressure monitor.** Veterans can independently input monitored health indices using apps such as **CBT-i Coach app, Annie app for Veterans, and cardiac monitoring devices (i.e. Alivecor app, Zio patch).** For synchronous collection of monitored health data during an appointment, additional VHR, such as the **3D Camera** used for monitoring wounds, can be invaluable*.* | - [Remote Patient Monitoring-Home Telehealth – Remote ... (blackboard.com)](https://vaots.blackboard.com/webapps/blackboard/execute/modulepage/view?course_id=_2686_1&cmp_tab_id=_3628_1&mode=view) - [Remote Patient Monitoring - Home Telehealth Manual (blackboard.com)](https://vaots.blackboard.com/bbcswebdav/library/LibraryContent/RPM-HT/rpm_ht_manual/rpm-ht.html) - [Patient-Generated Health Data – Patient-Generated Health... (blackboard.com)](https://vaots.blackboard.com/webapps/blackboard/execute/modulepage/view?course_id=_2774_1&cmp_tab_id=_3774_1&mode=view) - [Virtual Care Manager - Patient Generated Data Guide](https://vaots.blackboard.com/bbcswebdav/xid-1218654_1) - [VA Video Connect (VVC) – VA Video Connect (VVC... (blackboard.com)](https://vaots.blackboard.com/webapps/blackboard/execute/modulepage/view?course_id=_2591_1&cmp_tab_id=_3396_1&mode=view) - [Cardiology Wearables Use Cases (blackboard.com)](https://vaots.blackboard.com/bbcswebdav/library/LibraryContent/Cardiology/Cardiology%20Wearables%20Use%20Cases.pdf) - [Technical Brief 38: Automated-Entry Patient-Generated Health Data for Chronic Conditions: The Evidence on Health Outcomes (ahrq.gov)](https://effectivehealthcare.ahrq.gov/sites/default/files/pdf/health-data-mapping-technicalbrief.pdf)   **Scoping Review for Sensor, Wearable, and Remote Patient Monitoring Competencies for Clinical Care & Training:**  [Sensor, Wearable, and Remote Patient Monitoring Competencies for Clinical Care and Training: Scoping Review \| SpringerLink](https://link.springer.com/article/10.1007/s41347-020-00190-3) | **Pulse Oximeter:**   - [1226744 (amazonaws.com)](https://learn-us-gov-west-1-prod-fleet01-xythos.s3.us-gov-west-1.amazonaws.com/612eabb1bae6a/1226744?response-cache-control=private%2C%20max-age%3D10800&response-content-disposition=inline%3B%20filename%2A%3DUTF-8%27%27VVC%2520Peripheral%2520Pairing%2520Guide%2520Pulse%2520Oximeter.pdf&response-content-type=application%2Fpdf&X-Amz-Security-Token=FwoDYXdzEI%2F%2F%2F%2F%2F%2F%2F%2F%2F%2F%2FwEaDFCZGOlKZL9Gl1wfpiLkBN7WZx3ou1W58W5Y4NGgAAWBaSP1%2BTxJm8ftHpasVKz8romt2kT7quGI7VYNBio9Wf6gdURlPqmT5E3Omi2zzGtPXSj35m0JxFT5CP74CYkTyPzC65W0K%2F4lh2c5CLAJwm2cYsC5n7%2F1zholgnOn8YCq%2BZbzTB2C1b1vOhX1aJGYGgcX%2FTqVWA%2B6%2FO%2Bc1aYN0VZfh5MnDAvnEBfq3XkcmDVBMOpyTvIjZ2SmegpCSEqV4sAdfsd0ODS5OW0FKayWsmhcov%2BAQFqyGK8vWgsbnr7crgok0ZDTroSVmQHIpyWI8cx6y8cwEMZM9mDn7jCHMERzquFB5enFwxwnqo8AfueUBHUwc1KQl2INZIzz4QJHnQjFi%2BEIsOgjugXTObG%2FksT1GeKzzTfKhPd7vKceMI8fvC0B75%2FKvLOs%2FGQ6Khw0aP%2F7AEY2QoP9rI3AxNs6ypjAAQIqUuYJBGpPCSeRaG8uZabYT8SOZvVWwlEtd36BvkfMzAVjPqZ%2F2D%2BV%2Bf9qaeZH312l9tnIpGiyYMB7ZQM8B%2FWJT0PKzLTE8ttYTkwmNGWhjM2H%2BDnFsJ%2Fy30wlEdd7m8RVzc8%2FoRcHz4MiE%2FK7JFvlv%2FbK0CjgNG%2BJgSB1DE%2FJ50aDkdg%2Bb58Quzvwj1KvfvmEjzMgtAlZW4ZIGNDOaFy8Ow112o4%2FtpL3LiipiWXkdhjXxPKBNdPb05DMRlwA4kNI%2BGEYYVQZ7wxJjFLVR0X7H%2FhyHf7m1zFd%2FeN%2BfGbzzNOAcpw8Bz7%2BcWhX%2F2r3bCFoc4qR4WOKz3kv932LSF%2FovLfmskTeEnbipLBfb1WWaCiujqysBjJB3da%2Flyr9JL1kJSsW0I7T7GhDu3D2L0TJ3eJEjSaBxuUJwn%2B8QXSaoXkXJX9AOcTsyxYvyKZ7tv%2Bfh0dUb%2B9pJ8I%3D&X-Amz-Algorithm=AWS4-HMAC-SHA256&X-Amz-Date=20231226T180000Z&X-Amz-SignedHeaders=host&X-Amz-Expires=10800&X-Amz-Credential=ASIAUMDY6U44UCHTYQE5%2F20231226%2Fus-gov-west-1%2Fs3%2Faws4_request&X-Amz-Signature=599e132c9cc637522de224d1589932a1ac272df37738908bf295965554a877d5)   **Cardiac monitoring device:**   - [KardiaMobile Card is Available for Veterans \| Kardia](https://www.kardia.com/gov)   **Sleep Clinic Resources:**   - [Sleep-Clinic Workflow](https://vaots.blackboard.com/bbcswebdav/xid-567502_1) - [Sleep Program – Sleep Program Community (blackboard.com)](https://vaots.blackboard.com/webapps/blackboard/execute/modulepage/view?course_id=_2554_1&cmp_tab_id=_3336_1&mode=view) - [Sleep Intranet Resource Center](https://vaww.telehealth.va.gov/clinic/tsleep/index.asp)   **Blood Pressure Monitor:**   - [569283 (amazonaws.com)](https://learn-us-gov-west-1-prod-fleet01-xythos.s3.us-gov-west-1.amazonaws.com/612eabb1bae6a/569283?response-cache-control=private%2C%20max-age%3D10800&response-content-disposition=inline%3B%20filename%2A%3DUTF-8%27%27VVC%2520Peripheral%2520Pairing%2520Guide%2520Blood%2520Pressure.pdf&response-content-type=application%2Fpdf&X-Amz-Security-Token=FwoDYXdzEKb%2F%2F%2F%2F%2F%2F%2F%2F%2F%2FwEaDNihyzG6BBJlm7g3RSLkBDWBa0eQrvjTRXFLZ%2BwSo0GkYtWskMLQ8zmO4mhKnKFaOD2ykn%2FDRPWlnm%2BGluvTUL9g2mmOnTQXkipUWWOsocG%2Fn3HXRgOsLAHMoN%2B7ZepEzlYZhSwS1JDoOfrPIK6MsA%2BJ280pwu47T2YwJxmQlS6IO4Bu4%2F%2BF2%2BztY0oB67q%2Fihc2UB6FKI2mmaLFG0N4zALTvF42vDKLmj%2BtnRbB6VGYCiZi1stzI0ac4ePMSdvqewi6EvcGmpFE%2Bo4nXRD%2BtnHb2bEFulCmMNAh5gsKvytBYInE%2BkFXkOmiokGy%2FQYwnL%2FEntDtohbVWsLgxwjSQq5Iv%2BDA7MIRCWbNI7a6s9fu%2FUZVSYogvxu1AhD9I1HQoDpaamiey1ByYqbyIGzLFpLso0sB%2FPK9iOkjmA8e5%2BMZmbePeNyjZjh3YXYNMTYZa669ZIYame8cjRsaqlDAUnw39GHjhZAAsiJEmLbc69m%2FEvIaJaaeGNZnMnSx9T6QQAtE3WeEtaVGXoqYB8%2FkcSnHRTOW%2BUxXOU09XMd%2F5mOaX8xOrPRYalIPPii3d4JAaEJiiQm%2BOIW%2BHKerWLiEPPbJRjFLkcb83UBvRQ%2F0i7qY6%2FW56%2FcMNsIRgwtTvSOoHY%2FkECJoAKl8eQMrTgIjY9zZd6hDhSFk5p%2BK3mzZOCIRS2RdJYtQ6K5IZemc81pwdKNj%2FBEia8GoM0EYHpR004qtnatpY3BYi%2Fa9H0n6xhnafw3F9ggO7TNo%2FO2Hs3qlfkmivXYkqX2nXwEE2NzCOzIN979RlCP9P8OcFWkdige75XPfVK7pO5Og8%2Bi5xfMcF3l4iCjikLGsBjJBGvkKrrZkpHMqODM2DzouIsUJlFAminwrt%2BO0qWhVDKzWcW7wFlG7xv4EaAu9Kpe380FXITNhz97VbGWfKUnzR%2Bo%3D&X-Amz-Algorithm=AWS4-HMAC-SHA256&X-Amz-Date=20231227T163000Z&X-Amz-SignedHeaders=host&X-Amz-Expires=10800&X-Amz-Credential=ASIAUMDY6U44RUDMCFOA%2F20231227%2Fus-gov-west-1%2Fs3%2Faws4_request&X-Amz-Signature=0a5513cba3a39b2a5aa8519e38a85e1c020e63504500cb11abd0842ed86879ce)   **FitBit/Apple Watch:**   - [Digital Health Innovations - VA-Fitbit-HowToParticipate-1P.pdf - All Documents (sharepoint.com)](https://dvagov.sharepoint.com/sites/VHAV1NECIE/DHI/Shared%20Documents/Forms/AllItems.aspx?id=%2Fsites%2FVHAV1NECIE%2FDHI%2FShared%20Documents%2FVA%2DFitbit%2DHowToParticipate%2D1P%2Epdf&parent=%2Fsites%2FVHAV1NECIE%2FDHI%2FShared%20Documents) - [Digital Health Innovations - Fitbit Sense - Getting Started Guide - Veterans.pdf - All Documents (sharepoint.com)](https://dvagov.sharepoint.com/sites/VHAV1NECIE/DHI/Shared%20Documents/Forms/AllItems.aspx?id=%2Fsites%2FVHAV1NECIE%2FDHI%2FShared%20Documents%2FFitbit%20Sense%20%2D%20Getting%20Started%20Guide%20%2D%20Veterans%2Epdf&parent=%2Fsites%2FVHAV1NECIE%2FDHI%2FShared%20Documents)   **Peripheral Devices:**   - [Peripheral Devices \| During the VA Video Connect Visit \| VHA Telehealth Services Intranet](https://vaww.telehealth.va.gov/pgm/vvc/during/peripherals.asp)   **3-D camera:**   - [Library: TeleWound Care – Blackboard Learn](https://vaots.blackboard.com/webapps/cmsmain/webui/library/LibraryContent/Outreach/TeleWound%20Care?action=frameset&subaction=view&uniq=-8bswn3)   **Annie App for Veterans:**   - [Annie For Veterans \| VA Mobile](https://mobile.va.gov/app/annie-app-veterans)   **CBT-i Coach:**   - [CBT-i Coach \| VA Mobile](https://mobile.va.gov/app/cbt-i-coach)   **VA App Store:**   - [VA App Store \| VA Mobile](https://mobile.va.gov/appstore/) |
|  | **Summary #50^1,2,4^:** Through the telehealth program, you can monitor health indices such as sugar levels and can use a **Blood Pressure Monitor** to measure and automatically populate vital signs directly into **CPRS** to avoid errors. Telehealth vitals can be compiled into a note that tracks progress over time, which is saved on **CPRS**. You can also review the **Care Assessment Need (CAN) Risk Assessment** in **CPRS.** | - [Patient-Generated Health Data – Patient-Generated Health... (blackboard.com)](https://vaots.blackboard.com/webapps/blackboard/execute/modulepage/view?course_id=_2774_1&cmp_tab_id=_3774_1&mode=view) - <https://effectivehealthcare.ahrq.gov/sites/default/files/pdf/health-data-mapping-technicalbrief.pdf> | **Blood Pressure Monitor:**   - [569283 (amazonaws.com)](https://learn-us-gov-west-1-prod-fleet01-xythos.s3.us-gov-west-1.amazonaws.com/612eabb1bae6a/569283?response-cache-control=private%2C%20max-age%3D10800&response-content-disposition=inline%3B%20filename%2A%3DUTF-8%27%27VVC%2520Peripheral%2520Pairing%2520Guide%2520Blood%2520Pressure.pdf&response-content-type=application%2Fpdf&X-Amz-Security-Token=FwoDYXdzEKb%2F%2F%2F%2F%2F%2F%2F%2F%2F%2FwEaDNihyzG6BBJlm7g3RSLkBDWBa0eQrvjTRXFLZ%2BwSo0GkYtWskMLQ8zmO4mhKnKFaOD2ykn%2FDRPWlnm%2BGluvTUL9g2mmOnTQXkipUWWOsocG%2Fn3HXRgOsLAHMoN%2B7ZepEzlYZhSwS1JDoOfrPIK6MsA%2BJ280pwu47T2YwJxmQlS6IO4Bu4%2F%2BF2%2BztY0oB67q%2Fihc2UB6FKI2mmaLFG0N4zALTvF42vDKLmj%2BtnRbB6VGYCiZi1stzI0ac4ePMSdvqewi6EvcGmpFE%2Bo4nXRD%2BtnHb2bEFulCmMNAh5gsKvytBYInE%2BkFXkOmiokGy%2FQYwnL%2FEntDtohbVWsLgxwjSQq5Iv%2BDA7MIRCWbNI7a6s9fu%2FUZVSYogvxu1AhD9I1HQoDpaamiey1ByYqbyIGzLFpLso0sB%2FPK9iOkjmA8e5%2BMZmbePeNyjZjh3YXYNMTYZa669ZIYame8cjRsaqlDAUnw39GHjhZAAsiJEmLbc69m%2FEvIaJaaeGNZnMnSx9T6QQAtE3WeEtaVGXoqYB8%2FkcSnHRTOW%2BUxXOU09XMd%2F5mOaX8xOrPRYalIPPii3d4JAaEJiiQm%2BOIW%2BHKerWLiEPPbJRjFLkcb83UBvRQ%2F0i7qY6%2FW56%2FcMNsIRgwtTvSOoHY%2FkECJoAKl8eQMrTgIjY9zZd6hDhSFk5p%2BK3mzZOCIRS2RdJYtQ6K5IZemc81pwdKNj%2FBEia8GoM0EYHpR004qtnatpY3BYi%2Fa9H0n6xhnafw3F9ggO7TNo%2FO2Hs3qlfkmivXYkqX2nXwEE2NzCOzIN979RlCP9P8OcFWkdige75XPfVK7pO5Og8%2Bi5xfMcF3l4iCjikLGsBjJBGvkKrrZkpHMqODM2DzouIsUJlFAminwrt%2BO0qWhVDKzWcW7wFlG7xv4EaAu9Kpe380FXITNhz97VbGWfKUnzR%2Bo%3D&X-Amz-Algorithm=AWS4-HMAC-SHA256&X-Amz-Date=20231227T163000Z&X-Amz-SignedHeaders=host&X-Amz-Expires=10800&X-Amz-Credential=ASIAUMDY6U44RUDMCFOA%2F20231227%2Fus-gov-west-1%2Fs3%2Faws4_request&X-Amz-Signature=0a5513cba3a39b2a5aa8519e38a85e1c020e63504500cb11abd0842ed86879ce)   **CPRS:**   - [CPRS Technical Manual (va.gov)](https://www.va.gov/vdl/documents/Clinical/Comp_Patient_Recrd_Sys_(CPRS)/cprslmtm.pdf)   **CAN:**   - [Study gauges VA providers' views on predictive-analytics tool that assesses patient risk](https://www.research.va.gov/currents/1119-Researchers-study-VA-patient-data-tool-that-assesses-probability-of-hospitalization-and-death.cfm) - [713-notes.pdf (va.gov)](https://www.hsrd.research.va.gov/for_researchers/cyber_seminars/archives/713-notes.pdf) |
|  | **Summary #51^1^:** You can collect Veteran generated health data through **Apple watch/FitBit, Alivecor app,** and/or **Pulse Oximeter machine** for vitals and cardiac information. All can be and some are currently integrated to automatically upload data to **Share My Health Data (SMHD) app** for providers to view. | - [Patient-Generated Health Data – Patient-Generated Health... (blackboard.com)](https://vaots.blackboard.com/webapps/blackboard/execute/modulepage/view?course_id=_2774_1&cmp_tab_id=_3774_1&mode=view) - [Peripheral Devices \| During the VA Video Connect Visit \| VHA Telehealth Services Intranet](https://vaww.telehealth.va.gov/pgm/vvc/during/peripherals.asp) - <https://effectivehealthcare.ahrq.gov/sites/default/files/pdf/health-data-mapping-technicalbrief.pdf> | **FitBit/Apple Watch:**   - [Digital Health Innovations - VA-Fitbit-HowToParticipate-1P.pdf - All Documents (sharepoint.com)](https://dvagov.sharepoint.com/sites/VHAV1NECIE/DHI/Shared%20Documents/Forms/AllItems.aspx?id=%2Fsites%2FVHAV1NECIE%2FDHI%2FShared%20Documents%2FVA%2DFitbit%2DHowToParticipate%2D1P%2Epdf&parent=%2Fsites%2FVHAV1NECIE%2FDHI%2FShared%20Documents) - [Digital Health Innovations - Fitbit Sense - Getting Started Guide - Veterans.pdf - All Documents (sharepoint.com)](https://dvagov.sharepoint.com/sites/VHAV1NECIE/DHI/Shared%20Documents/Forms/AllItems.aspx?id=%2Fsites%2FVHAV1NECIE%2FDHI%2FShared%20Documents%2FFitbit%20Sense%20%2D%20Getting%20Started%20Guide%20%2D%20Veterans%2Epdf&parent=%2Fsites%2FVHAV1NECIE%2FDHI%2FShared%20Documents)   **Alivecor(app):**   - [KardiaMobile Card is Available for Veterans \| Kardia](https://www.kardia.com/gov)   **Pulse Oximeter machine:**   - [1226744 (amazonaws.com)](https://learn-us-gov-west-1-prod-fleet01-xythos.s3.us-gov-west-1.amazonaws.com/612eabb1bae6a/1226744?response-cache-control=private%2C%20max-age%3D10800&response-content-disposition=inline%3B%20filename%2A%3DUTF-8%27%27VVC%2520Peripheral%2520Pairing%2520Guide%2520Pulse%2520Oximeter.pdf&response-content-type=application%2Fpdf&X-Amz-Security-Token=FwoDYXdzEI%2F%2F%2F%2F%2F%2F%2F%2F%2F%2F%2FwEaDFCZGOlKZL9Gl1wfpiLkBN7WZx3ou1W58W5Y4NGgAAWBaSP1%2BTxJm8ftHpasVKz8romt2kT7quGI7VYNBio9Wf6gdURlPqmT5E3Omi2zzGtPXSj35m0JxFT5CP74CYkTyPzC65W0K%2F4lh2c5CLAJwm2cYsC5n7%2F1zholgnOn8YCq%2BZbzTB2C1b1vOhX1aJGYGgcX%2FTqVWA%2B6%2FO%2Bc1aYN0VZfh5MnDAvnEBfq3XkcmDVBMOpyTvIjZ2SmegpCSEqV4sAdfsd0ODS5OW0FKayWsmhcov%2BAQFqyGK8vWgsbnr7crgok0ZDTroSVmQHIpyWI8cx6y8cwEMZM9mDn7jCHMERzquFB5enFwxwnqo8AfueUBHUwc1KQl2INZIzz4QJHnQjFi%2BEIsOgjugXTObG%2FksT1GeKzzTfKhPd7vKceMI8fvC0B75%2FKvLOs%2FGQ6Khw0aP%2F7AEY2QoP9rI3AxNs6ypjAAQIqUuYJBGpPCSeRaG8uZabYT8SOZvVWwlEtd36BvkfMzAVjPqZ%2F2D%2BV%2Bf9qaeZH312l9tnIpGiyYMB7ZQM8B%2FWJT0PKzLTE8ttYTkwmNGWhjM2H%2BDnFsJ%2Fy30wlEdd7m8RVzc8%2FoRcHz4MiE%2FK7JFvlv%2FbK0CjgNG%2BJgSB1DE%2FJ50aDkdg%2Bb58Quzvwj1KvfvmEjzMgtAlZW4ZIGNDOaFy8Ow112o4%2FtpL3LiipiWXkdhjXxPKBNdPb05DMRlwA4kNI%2BGEYYVQZ7wxJjFLVR0X7H%2FhyHf7m1zFd%2FeN%2BfGbzzNOAcpw8Bz7%2BcWhX%2F2r3bCFoc4qR4WOKz3kv932LSF%2FovLfmskTeEnbipLBfb1WWaCiujqysBjJB3da%2Flyr9JL1kJSsW0I7T7GhDu3D2L0TJ3eJEjSaBxuUJwn%2B8QXSaoXkXJX9AOcTsyxYvyKZ7tv%2Bfh0dUb%2B9pJ8I%3D&X-Amz-Algorithm=AWS4-HMAC-SHA256&X-Amz-Date=20231226T180000Z&X-Amz-SignedHeaders=host&X-Amz-Expires=10800&X-Amz-Credential=ASIAUMDY6U44UCHTYQE5%2F20231226%2Fus-gov-west-1%2Fs3%2Faws4_request&X-Amz-Signature=599e132c9cc637522de224d1589932a1ac272df37738908bf295965554a877d5)   **Share My Health Data (SMHD) app:**   - [Share My Health Data \| VA Mobile](https://mobile.va.gov/app/share-my-health-data) |
| **8. PGHD Management** | | | |
| **8.1 Consult for PGHD Device Issuance** | **Summary #36^1,4,5^:** During the history and examination phase of treatment, staff can use **CPRS + ROES** to consult for device issuance. | - [Veteran Device Ordering Process \| Digital Divide Consult \| VHA Telehealth Services Intranet (va.gov)](https://vaww.telehealth.va.gov/technology/devices/vet-process.asp) | **CPRS:**   - [CPRS Technical Manual (va.gov)](https://www.va.gov/vdl/documents/Clinical/Comp_Patient_Recrd_Sys_(CPRS)/cprslmtm.pdf) - [CPRS Session 1 Getting Started](https://www.youtube.com/watch?v=y52UtvS95F0) - [Consult Tool Box Archives \| Office of Information and Technology (va.gov)](https://vaww.oit.va.gov/category/products/release-notes/ctb/)   **CPRS + ROES:**   - [Remote Order Entry System (ROES) - VA Authentication Federation Infrastructure (VAAFI)](https://www.va.gov/EAUTH/ROES/index.asp) |
|  | **Current Best Practice (Summary #52)*** Consults for PGHD Device issuance are entered through the **Remote Order Entry System [ROES] (CPRS).** A significant amount of PGHD can be collected using **VA apps,** which can be prescribed using the **VA Virtual Toolkit Prescription Pad.** Consults for device issuance of items addressing the digital divide, such as **iPads,** are entered through the **Consults Tool (CPRS).** Additional PGHD equipment, such as a wearable tracking device **(i.e. Fitbit)** can be ordered through **LEAF.** | - [Denver Acquisition and Logistics Center Remote Order Entry System (ROES) - VA Authentication Federation Infrastructure (VAAFI)](https://www.va.gov/EAUTH/ROES/DALC_ROES.asp) - <https://vaww.telehealth.va.gov/technology/devices/vet-process.asp> - <https://vaots.blackboard.com/bbcswebdav/xid-714516_1> - [Remote Patient Monitoring - Home Telehealth Manual (blackboard.com)](https://vaots.blackboard.com/bbcswebdav/library/LibraryContent/RPM-HT/rpm_ht_manual/rpm-ht.html) - <https://vaww.hsrd.research.va.gov/publications/esp/pt-adherence.pdf> | **VA Virtual Health Toolkit Prescription Pad:**   - [VA Virtual Health Toolkit Prescription Pad](https://connectedcare.va.gov/sites/default/files/2022-10/VA-Virtual-Health-Toolkit-Prescription-Pad.pdf)   **iPad:**   - [VA iPad Loaner Program/Digital Divide (sharepoint.com)](https://dvagov.sharepoint.com/sites/SFC/Tele/VVC/SitePages/VA-iPad-Loaner-Program-Digital-Divide.aspx)   **Consults Tool [CPRS]:**   - [Consult Tool Box Archives \| Office of Information and Technology (va.gov)](https://vaww.oit.va.gov/category/products/release-notes/ctb/)   **FitBit/Apple Watch:**   - [Digital Health Innovations - VA-Fitbit-HowToParticipate-1P.pdf - All Documents (sharepoint.com)](https://dvagov.sharepoint.com/sites/VHAV1NECIE/DHI/Shared%20Documents/Forms/AllItems.aspx?id=%2Fsites%2FVHAV1NECIE%2FDHI%2FShared%20Documents%2FVA%2DFitbit%2DHowToParticipate%2D1P%2Epdf&parent=%2Fsites%2FVHAV1NECIE%2FDHI%2FShared%20Documents) - [Digital Health Innovations - Fitbit Sense - Getting Started Guide - Veterans.pdf - All Documents (sharepoint.com)](https://dvagov.sharepoint.com/sites/VHAV1NECIE/DHI/Shared%20Documents/Forms/AllItems.aspx?id=%2Fsites%2FVHAV1NECIE%2FDHI%2FShared%20Documents%2FFitbit%20Sense%20%2D%20Getting%20Started%20Guide%20%2D%20Veterans%2Epdf&parent=%2Fsites%2FVHAV1NECIE%2FDHI%2FShared%20Documents)   **LEAF:**   - [LEAF Landing Page \| James A. Haley Veterans' Hospital (va.gov)](https://leaf.va.gov/VISN8/673/jhvh_leaf_homepage/) |
| **8.2: Track PGHD, Record Vitals & Monitor Health Indices** | **Summary #9^2,5^:** You can promote the use of **VA mobile apps** and **FitBit/Apple watch. FitBit** can be used to track health, sleep, steps, and pulse and to track Veteran heart rate monitoring in situations like when they are experiencing anxiety. | - <https://effectivehealthcare.ahrq.gov/sites/default/files/pdf/health-data-mapping-technicalbrief.pdf> - [Patient-Generated Health Data – Patient-Generated Health... (blackboard.com)](https://vaots.blackboard.com/webapps/blackboard/execute/modulepage/view?course_id=_2774_1&cmp_tab_id=_3774_1&mode=view) | **VA Mobile Apps**   - [App Store \| VA Mobile](https://mobile.va.gov/appstore?)   **FitBit/Apple Watch:**   - [Digital Health Innovations - VA-Fitbit-HowToParticipate-1P.pdf - All Documents (sharepoint.com)](https://dvagov.sharepoint.com/sites/VHAV1NECIE/DHI/Shared%20Documents/Forms/AllItems.aspx?id=%2Fsites%2FVHAV1NECIE%2FDHI%2FShared%20Documents%2FVA%2DFitbit%2DHowToParticipate%2D1P%2Epdf&parent=%2Fsites%2FVHAV1NECIE%2FDHI%2FShared%20Documents) - [Digital Health Innovations - Fitbit Sense - Getting Started Guide - Veterans.pdf - All Documents (sharepoint.com)](https://dvagov.sharepoint.com/sites/VHAV1NECIE/DHI/Shared%20Documents/Forms/AllItems.aspx?id=%2Fsites%2FVHAV1NECIE%2FDHI%2FShared%20Documents%2FFitbit%20Sense%20%2D%20Getting%20Started%20Guide%20%2D%20Veterans%2Epdf&parent=%2Fsites%2FVHAV1NECIE%2FDHI%2FShared%20Documents) |
|  | **Summary #11^1,4^:** When a Veteran enters vitals (BP, HR) to **Annie App for Veterans,** these data and other activities are generated into a dashboard in which you can view in the **Annie app for Clinicians.** You can also use the **Annie app for Clinicians** to track Veteran-specific health data like bladder and bowel care. | - <https://effectivehealthcare.ahrq.gov/sites/default/files/pdf/health-data-mapping-technicalbrief.pdf> - [Patient-Generated Health Data – Patient-Generated Health... (blackboard.com)](https://vaots.blackboard.com/webapps/blackboard/execute/modulepage/view?course_id=_2774_1&cmp_tab_id=_3774_1&mode=view) | **Annie App for Veterans (app):**   - [Annie For Veterans \| VA Mobile](https://mobile.va.gov/app/annie-app-veterans)   **Annie app for Clinicians (app):**   - [Annie For Clinicians \| VA Mobile](https://mobile.va.gov/app/annie-app-clinicians) - [Annie app for Clinicians: Session One – YouTube](https://www.youtube.com/watch?v=h0Qt10Oq8ss) - [Annie App – Annie App Community (blackboard.com)](https://vaots.blackboard.com/webapps/blackboard/execute/modulepage/view?course_id=_2548_1&cmp_tab_id=_3324_1&mode=view) |
|  | **Summary #19^5^:** Provider can use apps such as **CBT-i-Coach app, PTSD Coach app, CPT Coach app** and **Insomnia Coach app** to use during post-appointment for providing treatment and to manage and track PGHD. | - [Patient-Generated Health Data – Patient-Generated Health... (blackboard.com)](https://vaots.blackboard.com/webapps/blackboard/execute/modulepage/view?course_id=_2774_1&cmp_tab_id=_3774_1&mode=view) | **VA App Store:**   - [VA App Store \| VA Mobile](https://mobile.va.gov/appstore/)   **CBT-i (Cognitive Behavioral Therapy for Insomnia) Coach App:**   - [CBT-i Coach \| VA Mobile](https://mobile.va.gov/app/cbt-i-coach)   **PTSD (Post Traumatic Stress Disorder) Coach App:**   - [PTSD Coach \| VA Mobile](https://mobile.va.gov/app/ptsd-coach)   **CPT (Cognitive Processing Therapy) Coach App:**   - [CPT Coach \| VA Mobile](https://mobile.va.gov/app/cpt-coach)   **Insomnia Coach App:**   - [Insomnia Coach \| VA Mobile](https://mobile.va.gov/app/insomnia-coach) |
|  | **Summary #30^1,2^:** Staff and providers encourage Veterans to use **SM (MHV)** to communicate and to use **VA Mobile apps** and tracking devices such as **FitBit** or **Apple Watch** for tracking communication and health data. | - [MHV_SM_HCT_User_Manual_August_2023.pdf (va.gov)](https://vaww.va.gov/MYHEALTHEVET/docs/secure_messaging/MHV_SM_HCT_User_Manual_August_2023.pdf) - [Peripheral Devices \| During the VA Video Connect Visit \| VHA Telehealth Services Intranet](https://vaww.telehealth.va.gov/pgm/vvc/during/peripherals.asp) | **VA Mobile Apps:**   - [VA Mobile Health Practice Guide](https://connectedcare.va.gov/sites/default/files/2021-10/va-mobile-health-practice-guide.pdf)   **FitBit/Apple Watch:**   - [Digital Health Innovations - VA-Fitbit-HowToParticipate-1P.pdf - All Documents (sharepoint.com)](https://dvagov.sharepoint.com/sites/VHAV1NECIE/DHI/Shared%20Documents/Forms/AllItems.aspx?id=%2Fsites%2FVHAV1NECIE%2FDHI%2FShared%20Documents%2FVA%2DFitbit%2DHowToParticipate%2D1P%2Epdf&parent=%2Fsites%2FVHAV1NECIE%2FDHI%2FShared%20Documents) - [Digital Health Innovations - Fitbit Sense - Getting Started Guide - Veterans.pdf - All Documents (sharepoint.com)](https://dvagov.sharepoint.com/sites/VHAV1NECIE/DHI/Shared%20Documents/Forms/AllItems.aspx?id=%2Fsites%2FVHAV1NECIE%2FDHI%2FShared%20Documents%2FFitbit%20Sense%20%2D%20Getting%20Started%20Guide%20%2D%20Veterans%2Epdf&parent=%2Fsites%2FVHAV1NECIE%2FDHI%2FShared%20Documents)   **SM (MHV):**   - <https://www.myhealth.va.gov/mhv-portal-web/home> - [My HealtheVet Resources – My HealtheVet Resources](https://vaots.blackboard.com/webapps/blackboard/execute/modulepage/view?course_id=_3174_1&cmp_tab_id=_4591_1&mode=view) - [Secure Messaging Through My HealtheVet – My HealtheVet Product (va.gov)](https://vaww.va.gov/MYHEALTHEVET/Secure_Messaging.asp) |
|  | **Summary #37^1,2,4,5^:** Providers can monitor health indices and identify abnormalities by reviewing the **Care Assessment Need (CAN) Risk Assessment (CPRS)** and other health data uploaded into the patients’ charts through the telehealth program. Additional health indices can be monitored when the patient shares PGHD during a healthcare visit that they collected or documented on various apps or devices, but it is important to promote the use of select VA mobile apps and/or preferred devices with the Veteran. | - [WebVRAM – WebVRAM Community (blackboard.com)](https://vaots.blackboard.com/webapps/blackboard/execute/modulepage/view?course_id=_2481_1&cmp_tab_id=_3205_1&mode=view) - [Remote Patient Monitoring - Home Telehealth Manual (blackboard.com)](https://vaots.blackboard.com/bbcswebdav/library/LibraryContent/RPM-HT/rpm_ht_manual/rpm-ht.html) - [Sensor, Wearable, and Remote Patient Monitoring Competencies for Clinical Care and Training: Scoping Review (amazonaws.com)](https://learn-us-gov-west-1-prod-fleet01-xythos.s3.us-gov-west-1.amazonaws.com/612eabb1bae6a/622493?response-cache-control=private%2C%20max-age%3D10800&response-content-disposition=inline%3B%20filename%2A%3DUTF-8%27%27Clinical%2520Competency%2520for%2520Use%2520of%2520Sensors%252C%2520Wearables%252C%2520Remote%2520Patient%2520Monitoring.pdf&response-content-type=application%2Fpdf&X-Amz-Security-Token=FwoDYXdzEJH%2F%2F%2F%2F%2F%2F%2F%2F%2F%2FwEaDN%2B8TpaTNSUJTFiqPyLlBA%2B5kzFuLAS0LEIGwvmZrYYdj%2B6H9i%2B%2FPmr3uyF5vTgI9kYOJy1u%2FgC9bNYQbDez59m%2FjoaI9sTaWRcV3aB29nqxcJv9Q8c%2BdHxQFNRpSp4KGJhrw%2FOFatJan2idYrhoU0FPCcyNMLe7dSMDH3EY4VIR3grGJoTlOXXCSMIvaks0zsBSdm1xPT169C6JOVnvA9Nai50%2BL4U77MXt8gVUbnCKRlT%2BPpui1RKtK7HXVY%2BUvmmYTQs1g1rLtMVqxfhfMAFx9zn903tYfygZtV4m5HPI7OZcSY0DLYIftnHCO2G5%2Bo%2Fb3d%2FO285zDwLArwvAU48Cx7CEOOZxZlCWEbg0RtyRtDBMuIzYa8ln0CmVnW8wHK686yZ8oqWE%2BvS5fKD5lKx4aIaGUQSiXBW0YfrRSbL0ATldILZNqj8GdUdTx3PgSY9puuwWeuK3TGiZYAG1mCTub4fvhXJsTCD3AeRGGIAOE80hRNWVpZ%2FmWoi6UHCvrj1AMWYS33KEg4MDe70hVlhipMyr0OYC6h48iuWUokajZkggNNFYTxLwlBzGkRtJCBL0GALdXIX7rvigarOrm6lbCHMgaqS%2FO%2F0V%2Bt2fjJ84CMs%2FymP6gbhCtgE0PSUWdD%2FVOf%2FV2SwsFEsb2p9wOv9MRoYevhGDodZGq1HU5jUUTSfkWqjUXYRBhyQoOxvoNoZdYfnDDoME1LCBcqU4lU3ifYSknek0MQFLI5tspXwun7oXIZSIye16V%2Bu%2FexvmwfBbqbjn96Gx0Sp9j87F%2F%2BbwOzzz7TivuX%2BIje5pE%2FYPWsdsJRG9KmDYXDbhmYGk2Ay9e8QoiMbvogYyQafPFJsmsF407MKRClAq4l1m7rRICxHftwW0hu4mkPdci677%2B3Nqgk4QrIm5vXDbV0MAVwx2tREjY4mMcywv%2Bheb&X-Amz-Algorithm=AWS4-HMAC-SHA256&X-Amz-Date=20230510T193000Z&X-Amz-SignedHeaders=host&X-Amz-Expires=10800&X-Amz-Credential=ASIAUMDY6U444MOL72AQ%2F20230510%2Fus-gov-west-1%2Fs3%2Faws4_request&X-Amz-Signature=e02a75f342a825c18b499f1f5969f8af962964b836bf714a56cc5f7991f7bed5) - [Technical Brief 38: Automated-Entry Patient-Generated Health Data for Chronic Conditions: The Evidence on Health Outcomes (ahrq.gov)](https://effectivehealthcare.ahrq.gov/sites/default/files/pdf/health-data-mapping-technicalbrief.pdf) | **CAN:**   - [Study gauges VA providers' views on predictive-analytics tool that assesses patient risk](https://www.research.va.gov/currents/1119-Researchers-study-VA-patient-data-tool-that-assesses-probability-of-hospitalization-and-death.cfm) - [713-notes.pdf (va.gov)](https://www.hsrd.research.va.gov/for_researchers/cyber_seminars/archives/713-notes.pdf)   **Peripherals:**   - <https://vaww.telehealth.va.gov/pgm/vvc/during/peripherals.asp> |
|  | **Summary #39^4^:** You can use the **3D camera** to assist with tracking wound care. | - <https://effectivehealthcare.ahrq.gov/sites/default/files/pdf/health-data-mapping-technicalbrief.pdf> - [Patient-Generated Health Data – Patient-Generated Health... (blackboard.com)](https://vaots.blackboard.com/webapps/blackboard/execute/modulepage/view?course_id=_2774_1&cmp_tab_id=_3774_1&mode=view) | **3-D camera:**   - [Library: TeleWound Care – Blackboard Learn](https://vaots.blackboard.com/webapps/cmsmain/webui/library/LibraryContent/Outreach/TeleWound%20Care?action=frameset&subaction=view&uniq=-8bswn3) |
|  | **Summary #42^1,4,5^:** Monitored health information can be gathered and acquired of variety of ways. Patient Generated Health Data [PGHD] can be obtained asynchronously through equipment such as a wearable health tracking device **(fitBit, Apple Watch), Pulse oximeter, glucometer, digital scale, or blood pressure monitor.** Veterans can independently input monitored health indices using apps such as **CBT-i Coach app, Annie app for Veterans, and cardiac monitoring devices (i.e. Alivecor app, Zio patch).** For synchronous collection of monitored health data during an appointment, additional VHR, such as the **3D Camera** used for monitoring wounds, can be invaluable*.* | - [Remote Patient Monitoring-Home Telehealth – Remote ... (blackboard.com)](https://vaots.blackboard.com/webapps/blackboard/execute/modulepage/view?course_id=_2686_1&cmp_tab_id=_3628_1&mode=view) - [Remote Patient Monitoring - Home Telehealth Manual (blackboard.com)](https://vaots.blackboard.com/bbcswebdav/library/LibraryContent/RPM-HT/rpm_ht_manual/rpm-ht.html) - [Patient-Generated Health Data – Patient-Generated Health... (blackboard.com)](https://vaots.blackboard.com/webapps/blackboard/execute/modulepage/view?course_id=_2774_1&cmp_tab_id=_3774_1&mode=view) - [Virtual Care Manager - Patient Generated Data Guide](https://vaots.blackboard.com/bbcswebdav/xid-1218654_1) - [VA Video Connect (VVC) – VA Video Connect (VVC... (blackboard.com)](https://vaots.blackboard.com/webapps/blackboard/execute/modulepage/view?course_id=_2591_1&cmp_tab_id=_3396_1&mode=view) - [Cardiology Wearables Use Cases (blackboard.com)](https://vaots.blackboard.com/bbcswebdav/library/LibraryContent/Cardiology/Cardiology%20Wearables%20Use%20Cases.pdf) - [Technical Brief 38: Automated-Entry Patient-Generated Health Data for Chronic Conditions: The Evidence on Health Outcomes (ahrq.gov)](https://effectivehealthcare.ahrq.gov/sites/default/files/pdf/health-data-mapping-technicalbrief.pdf)   **Scoping Review for Sensor, Wearable, and Remote Patient Monitoring Competencies for Clinical Care & Training:**   - [Sensor, Wearable, and Remote Patient Monitoring Competencies for Clinical Care and Training: Scoping Review \| SpringerLink](https://link.springer.com/article/10.1007/s41347-020-00190-3) | **Pulse Oximeter:**   - [1226744 (amazonaws.com)](https://learn-us-gov-west-1-prod-fleet01-xythos.s3.us-gov-west-1.amazonaws.com/612eabb1bae6a/1226744?response-cache-control=private%2C%20max-age%3D10800&response-content-disposition=inline%3B%20filename%2A%3DUTF-8%27%27VVC%2520Peripheral%2520Pairing%2520Guide%2520Pulse%2520Oximeter.pdf&response-content-type=application%2Fpdf&X-Amz-Security-Token=FwoDYXdzEI%2F%2F%2F%2F%2F%2F%2F%2F%2F%2F%2FwEaDFCZGOlKZL9Gl1wfpiLkBN7WZx3ou1W58W5Y4NGgAAWBaSP1%2BTxJm8ftHpasVKz8romt2kT7quGI7VYNBio9Wf6gdURlPqmT5E3Omi2zzGtPXSj35m0JxFT5CP74CYkTyPzC65W0K%2F4lh2c5CLAJwm2cYsC5n7%2F1zholgnOn8YCq%2BZbzTB2C1b1vOhX1aJGYGgcX%2FTqVWA%2B6%2FO%2Bc1aYN0VZfh5MnDAvnEBfq3XkcmDVBMOpyTvIjZ2SmegpCSEqV4sAdfsd0ODS5OW0FKayWsmhcov%2BAQFqyGK8vWgsbnr7crgok0ZDTroSVmQHIpyWI8cx6y8cwEMZM9mDn7jCHMERzquFB5enFwxwnqo8AfueUBHUwc1KQl2INZIzz4QJHnQjFi%2BEIsOgjugXTObG%2FksT1GeKzzTfKhPd7vKceMI8fvC0B75%2FKvLOs%2FGQ6Khw0aP%2F7AEY2QoP9rI3AxNs6ypjAAQIqUuYJBGpPCSeRaG8uZabYT8SOZvVWwlEtd36BvkfMzAVjPqZ%2F2D%2BV%2Bf9qaeZH312l9tnIpGiyYMB7ZQM8B%2FWJT0PKzLTE8ttYTkwmNGWhjM2H%2BDnFsJ%2Fy30wlEdd7m8RVzc8%2FoRcHz4MiE%2FK7JFvlv%2FbK0CjgNG%2BJgSB1DE%2FJ50aDkdg%2Bb58Quzvwj1KvfvmEjzMgtAlZW4ZIGNDOaFy8Ow112o4%2FtpL3LiipiWXkdhjXxPKBNdPb05DMRlwA4kNI%2BGEYYVQZ7wxJjFLVR0X7H%2FhyHf7m1zFd%2FeN%2BfGbzzNOAcpw8Bz7%2BcWhX%2F2r3bCFoc4qR4WOKz3kv932LSF%2FovLfmskTeEnbipLBfb1WWaCiujqysBjJB3da%2Flyr9JL1kJSsW0I7T7GhDu3D2L0TJ3eJEjSaBxuUJwn%2B8QXSaoXkXJX9AOcTsyxYvyKZ7tv%2Bfh0dUb%2B9pJ8I%3D&X-Amz-Algorithm=AWS4-HMAC-SHA256&X-Amz-Date=20231226T180000Z&X-Amz-SignedHeaders=host&X-Amz-Expires=10800&X-Amz-Credential=ASIAUMDY6U44UCHTYQE5%2F20231226%2Fus-gov-west-1%2Fs3%2Faws4_request&X-Amz-Signature=599e132c9cc637522de224d1589932a1ac272df37738908bf295965554a877d5)   **Cardiac monitoring device:**   - [KardiaMobile Card is Available for Veterans \| Kardia](https://www.kardia.com/gov)   **Sleep Clinic Resources:**   - [Sleep-Clinic Workflow](https://vaots.blackboard.com/bbcswebdav/xid-567502_1) - [Sleep Program – Sleep Program Community (blackboard.com)](https://vaots.blackboard.com/webapps/blackboard/execute/modulepage/view?course_id=_2554_1&cmp_tab_id=_3336_1&mode=view) - [Sleep Intranet Resource Center](https://vaww.telehealth.va.gov/clinic/tsleep/index.asp)   **Blood Pressure Monitor:**   - [569283 (amazonaws.com)](https://learn-us-gov-west-1-prod-fleet01-xythos.s3.us-gov-west-1.amazonaws.com/612eabb1bae6a/569283?response-cache-control=private%2C%20max-age%3D10800&response-content-disposition=inline%3B%20filename%2A%3DUTF-8%27%27VVC%2520Peripheral%2520Pairing%2520Guide%2520Blood%2520Pressure.pdf&response-content-type=application%2Fpdf&X-Amz-Security-Token=FwoDYXdzEKb%2F%2F%2F%2F%2F%2F%2F%2F%2F%2FwEaDNihyzG6BBJlm7g3RSLkBDWBa0eQrvjTRXFLZ%2BwSo0GkYtWskMLQ8zmO4mhKnKFaOD2ykn%2FDRPWlnm%2BGluvTUL9g2mmOnTQXkipUWWOsocG%2Fn3HXRgOsLAHMoN%2B7ZepEzlYZhSwS1JDoOfrPIK6MsA%2BJ280pwu47T2YwJxmQlS6IO4Bu4%2F%2BF2%2BztY0oB67q%2Fihc2UB6FKI2mmaLFG0N4zALTvF42vDKLmj%2BtnRbB6VGYCiZi1stzI0ac4ePMSdvqewi6EvcGmpFE%2Bo4nXRD%2BtnHb2bEFulCmMNAh5gsKvytBYInE%2BkFXkOmiokGy%2FQYwnL%2FEntDtohbVWsLgxwjSQq5Iv%2BDA7MIRCWbNI7a6s9fu%2FUZVSYogvxu1AhD9I1HQoDpaamiey1ByYqbyIGzLFpLso0sB%2FPK9iOkjmA8e5%2BMZmbePeNyjZjh3YXYNMTYZa669ZIYame8cjRsaqlDAUnw39GHjhZAAsiJEmLbc69m%2FEvIaJaaeGNZnMnSx9T6QQAtE3WeEtaVGXoqYB8%2FkcSnHRTOW%2BUxXOU09XMd%2F5mOaX8xOrPRYalIPPii3d4JAaEJiiQm%2BOIW%2BHKerWLiEPPbJRjFLkcb83UBvRQ%2F0i7qY6%2FW56%2FcMNsIRgwtTvSOoHY%2FkECJoAKl8eQMrTgIjY9zZd6hDhSFk5p%2BK3mzZOCIRS2RdJYtQ6K5IZemc81pwdKNj%2FBEia8GoM0EYHpR004qtnatpY3BYi%2Fa9H0n6xhnafw3F9ggO7TNo%2FO2Hs3qlfkmivXYkqX2nXwEE2NzCOzIN979RlCP9P8OcFWkdige75XPfVK7pO5Og8%2Bi5xfMcF3l4iCjikLGsBjJBGvkKrrZkpHMqODM2DzouIsUJlFAminwrt%2BO0qWhVDKzWcW7wFlG7xv4EaAu9Kpe380FXITNhz97VbGWfKUnzR%2Bo%3D&X-Amz-Algorithm=AWS4-HMAC-SHA256&X-Amz-Date=20231227T163000Z&X-Amz-SignedHeaders=host&X-Amz-Expires=10800&X-Amz-Credential=ASIAUMDY6U44RUDMCFOA%2F20231227%2Fus-gov-west-1%2Fs3%2Faws4_request&X-Amz-Signature=0a5513cba3a39b2a5aa8519e38a85e1c020e63504500cb11abd0842ed86879ce)   **FitBit/Apple Watch:**   - [Digital Health Innovations - VA-Fitbit-HowToParticipate-1P.pdf - All Documents (sharepoint.com)](https://dvagov.sharepoint.com/sites/VHAV1NECIE/DHI/Shared%20Documents/Forms/AllItems.aspx?id=%2Fsites%2FVHAV1NECIE%2FDHI%2FShared%20Documents%2FVA%2DFitbit%2DHowToParticipate%2D1P%2Epdf&parent=%2Fsites%2FVHAV1NECIE%2FDHI%2FShared%20Documents) - [Digital Health Innovations - Fitbit Sense - Getting Started Guide - Veterans.pdf - All Documents (sharepoint.com)](https://dvagov.sharepoint.com/sites/VHAV1NECIE/DHI/Shared%20Documents/Forms/AllItems.aspx?id=%2Fsites%2FVHAV1NECIE%2FDHI%2FShared%20Documents%2FFitbit%20Sense%20%2D%20Getting%20Started%20Guide%20%2D%20Veterans%2Epdf&parent=%2Fsites%2FVHAV1NECIE%2FDHI%2FShared%20Documents)   **Peripheral Devices:**   - [Peripheral Devices \| During the VA Video Connect Visit \| VHA Telehealth Services Intranet](https://vaww.telehealth.va.gov/pgm/vvc/during/peripherals.asp)   **3-D camera:**   - [Library: TeleWound Care – Blackboard Learn](https://vaots.blackboard.com/webapps/cmsmain/webui/library/LibraryContent/Outreach/TeleWound%20Care?action=frameset&subaction=view&uniq=-8bswn3)   **Annie App for Veterans:**   - [Annie For Veterans \| VA Mobile](https://mobile.va.gov/app/annie-app-veterans)   **CBT-i Coach:**   - [CBT-i Coach \| VA Mobile](https://mobile.va.gov/app/cbt-i-coach)   **VA App Store:**   - [VA App Store \| VA Mobile](https://mobile.va.gov/appstore/) |
|  |  |  |  |
|  | **Summary #49^4,5^:** You can use the **CBT-i Coach app** and the data from **Apple Watch/FitBit** to review and track weekly sleep cycles and can adjust the schedule when needed. You can utilize information from the service connection disability rating and vitals generated in both **CPRS** and **CBT-i** Coach to ensure Veterans are improving over the course of time together with the provider. | - <https://effectivehealthcare.ahrq.gov/sites/default/files/pdf/health-data-mapping-technicalbrief.pdf> - [Patient-Generated Health Data – Patient-Generated Health... (blackboard.com)](https://vaots.blackboard.com/webapps/blackboard/execute/modulepage/view?course_id=_2774_1&cmp_tab_id=_3774_1&mode=view) | **CBT-I Coach:**   - [CBT-i Coach \| VA Mobile](https://mobile.va.gov/app/cbt-i-coach)   **FitBit/Apple Watch:**   - [Digital Health Innovations - VA-Fitbit-HowToParticipate-1P.pdf - All Documents (sharepoint.com)](https://dvagov.sharepoint.com/sites/VHAV1NECIE/DHI/Shared%20Documents/Forms/AllItems.aspx?id=%2Fsites%2FVHAV1NECIE%2FDHI%2FShared%20Documents%2FVA%2DFitbit%2DHowToParticipate%2D1P%2Epdf&parent=%2Fsites%2FVHAV1NECIE%2FDHI%2FShared%20Documents) - [Digital Health Innovations - Fitbit Sense - Getting Started Guide - Veterans.pdf - All Documents (sharepoint.com)](https://dvagov.sharepoint.com/sites/VHAV1NECIE/DHI/Shared%20Documents/Forms/AllItems.aspx?id=%2Fsites%2FVHAV1NECIE%2FDHI%2FShared%20Documents%2FFitbit%20Sense%20%2D%20Getting%20Started%20Guide%20%2D%20Veterans%2Epdf&parent=%2Fsites%2FVHAV1NECIE%2FDHI%2FShared%20Documents) |
|  | **Summary #50^1,2,4^:** Through the telehealth program, you can monitor health indices such as sugar levels and can use a **Blood Pressure Monitor** to measure and automatically populate vital signs directly into **CPRS** to avoid errors. Telehealth vitals can be compiled into a note that tracks progress over time, which is saved on **CPRS.** You can also review the **Care Assessment Need (CAN) Risk Assessment** in **CPRS.** | - [Patient-Generated Health Data – Patient-Generated Health... (blackboard.com)](https://vaots.blackboard.com/webapps/blackboard/execute/modulepage/view?course_id=_2774_1&cmp_tab_id=_3774_1&mode=view) - <https://effectivehealthcare.ahrq.gov/sites/default/files/pdf/health-data-mapping-technicalbrief.pdf> | **Blood Pressure Monitor:**   - [569283 (amazonaws.com)](https://learn-us-gov-west-1-prod-fleet01-xythos.s3.us-gov-west-1.amazonaws.com/612eabb1bae6a/569283?response-cache-control=private%2C%20max-age%3D10800&response-content-disposition=inline%3B%20filename%2A%3DUTF-8%27%27VVC%2520Peripheral%2520Pairing%2520Guide%2520Blood%2520Pressure.pdf&response-content-type=application%2Fpdf&X-Amz-Security-Token=FwoDYXdzEKb%2F%2F%2F%2F%2F%2F%2F%2F%2F%2FwEaDNihyzG6BBJlm7g3RSLkBDWBa0eQrvjTRXFLZ%2BwSo0GkYtWskMLQ8zmO4mhKnKFaOD2ykn%2FDRPWlnm%2BGluvTUL9g2mmOnTQXkipUWWOsocG%2Fn3HXRgOsLAHMoN%2B7ZepEzlYZhSwS1JDoOfrPIK6MsA%2BJ280pwu47T2YwJxmQlS6IO4Bu4%2F%2BF2%2BztY0oB67q%2Fihc2UB6FKI2mmaLFG0N4zALTvF42vDKLmj%2BtnRbB6VGYCiZi1stzI0ac4ePMSdvqewi6EvcGmpFE%2Bo4nXRD%2BtnHb2bEFulCmMNAh5gsKvytBYInE%2BkFXkOmiokGy%2FQYwnL%2FEntDtohbVWsLgxwjSQq5Iv%2BDA7MIRCWbNI7a6s9fu%2FUZVSYogvxu1AhD9I1HQoDpaamiey1ByYqbyIGzLFpLso0sB%2FPK9iOkjmA8e5%2BMZmbePeNyjZjh3YXYNMTYZa669ZIYame8cjRsaqlDAUnw39GHjhZAAsiJEmLbc69m%2FEvIaJaaeGNZnMnSx9T6QQAtE3WeEtaVGXoqYB8%2FkcSnHRTOW%2BUxXOU09XMd%2F5mOaX8xOrPRYalIPPii3d4JAaEJiiQm%2BOIW%2BHKerWLiEPPbJRjFLkcb83UBvRQ%2F0i7qY6%2FW56%2FcMNsIRgwtTvSOoHY%2FkECJoAKl8eQMrTgIjY9zZd6hDhSFk5p%2BK3mzZOCIRS2RdJYtQ6K5IZemc81pwdKNj%2FBEia8GoM0EYHpR004qtnatpY3BYi%2Fa9H0n6xhnafw3F9ggO7TNo%2FO2Hs3qlfkmivXYkqX2nXwEE2NzCOzIN979RlCP9P8OcFWkdige75XPfVK7pO5Og8%2Bi5xfMcF3l4iCjikLGsBjJBGvkKrrZkpHMqODM2DzouIsUJlFAminwrt%2BO0qWhVDKzWcW7wFlG7xv4EaAu9Kpe380FXITNhz97VbGWfKUnzR%2Bo%3D&X-Amz-Algorithm=AWS4-HMAC-SHA256&X-Amz-Date=20231227T163000Z&X-Amz-SignedHeaders=host&X-Amz-Expires=10800&X-Amz-Credential=ASIAUMDY6U44RUDMCFOA%2F20231227%2Fus-gov-west-1%2Fs3%2Faws4_request&X-Amz-Signature=0a5513cba3a39b2a5aa8519e38a85e1c020e63504500cb11abd0842ed86879ce)   **CPRS:**   - [CPRS Technical Manual (va.gov)](https://www.va.gov/vdl/documents/Clinical/Comp_Patient_Recrd_Sys_(CPRS)/cprslmtm.pdf)   **CAN:**   - [Study gauges VA providers' views on predictive-analytics tool that assesses patient risk](https://www.research.va.gov/currents/1119-Researchers-study-VA-patient-data-tool-that-assesses-probability-of-hospitalization-and-death.cfm) - [713-notes.pdf (va.gov)](https://www.hsrd.research.va.gov/for_researchers/cyber_seminars/archives/713-notes.pdf) |
|  | **Summary #52^1^:** You can collect Veteran generated health data through **Apple watch/FitBit, Alivecor app,** and/or **Pulse Oximeter machine** for vitals and cardiac information. All can be and some are currently integrated to automatically upload data to **Share My Health Data (SMHD) app** for providers to view. | - [Patient-Generated Health Data – Patient-Generated Health... (blackboard.com)](https://vaots.blackboard.com/webapps/blackboard/execute/modulepage/view?course_id=_2774_1&cmp_tab_id=_3774_1&mode=view) - [Peripheral Devices \| During the VA Video Connect Visit \| VHA Telehealth Services Intranet](https://vaww.telehealth.va.gov/pgm/vvc/during/peripherals.asp) - <https://effectivehealthcare.ahrq.gov/sites/default/files/pdf/health-data-mapping-technicalbrief.pdf> | **FitBit/Apple Watch:**   - [Digital Health Innovations - VA-Fitbit-HowToParticipate-1P.pdf - All Documents (sharepoint.com)](https://dvagov.sharepoint.com/sites/VHAV1NECIE/DHI/Shared%20Documents/Forms/AllItems.aspx?id=%2Fsites%2FVHAV1NECIE%2FDHI%2FShared%20Documents%2FVA%2DFitbit%2DHowToParticipate%2D1P%2Epdf&parent=%2Fsites%2FVHAV1NECIE%2FDHI%2FShared%20Documents) - [Digital Health Innovations - Fitbit Sense - Getting Started Guide - Veterans.pdf - All Documents (sharepoint.com)](https://dvagov.sharepoint.com/sites/VHAV1NECIE/DHI/Shared%20Documents/Forms/AllItems.aspx?id=%2Fsites%2FVHAV1NECIE%2FDHI%2FShared%20Documents%2FFitbit%20Sense%20%2D%20Getting%20Started%20Guide%20%2D%20Veterans%2Epdf&parent=%2Fsites%2FVHAV1NECIE%2FDHI%2FShared%20Documents)   **Alivecor(app):**   - [KardiaMobile Card is Available for Veterans \| Kardia](https://www.kardia.com/gov)   **Pulse Oximeter machine:**   - [1226744 (amazonaws.com)](https://learn-us-gov-west-1-prod-fleet01-xythos.s3.us-gov-west-1.amazonaws.com/612eabb1bae6a/1226744?response-cache-control=private%2C%20max-age%3D10800&response-content-disposition=inline%3B%20filename%2A%3DUTF-8%27%27VVC%2520Peripheral%2520Pairing%2520Guide%2520Pulse%2520Oximeter.pdf&response-content-type=application%2Fpdf&X-Amz-Security-Token=FwoDYXdzEI%2F%2F%2F%2F%2F%2F%2F%2F%2F%2F%2FwEaDFCZGOlKZL9Gl1wfpiLkBN7WZx3ou1W58W5Y4NGgAAWBaSP1%2BTxJm8ftHpasVKz8romt2kT7quGI7VYNBio9Wf6gdURlPqmT5E3Omi2zzGtPXSj35m0JxFT5CP74CYkTyPzC65W0K%2F4lh2c5CLAJwm2cYsC5n7%2F1zholgnOn8YCq%2BZbzTB2C1b1vOhX1aJGYGgcX%2FTqVWA%2B6%2FO%2Bc1aYN0VZfh5MnDAvnEBfq3XkcmDVBMOpyTvIjZ2SmegpCSEqV4sAdfsd0ODS5OW0FKayWsmhcov%2BAQFqyGK8vWgsbnr7crgok0ZDTroSVmQHIpyWI8cx6y8cwEMZM9mDn7jCHMERzquFB5enFwxwnqo8AfueUBHUwc1KQl2INZIzz4QJHnQjFi%2BEIsOgjugXTObG%2FksT1GeKzzTfKhPd7vKceMI8fvC0B75%2FKvLOs%2FGQ6Khw0aP%2F7AEY2QoP9rI3AxNs6ypjAAQIqUuYJBGpPCSeRaG8uZabYT8SOZvVWwlEtd36BvkfMzAVjPqZ%2F2D%2BV%2Bf9qaeZH312l9tnIpGiyYMB7ZQM8B%2FWJT0PKzLTE8ttYTkwmNGWhjM2H%2BDnFsJ%2Fy30wlEdd7m8RVzc8%2FoRcHz4MiE%2FK7JFvlv%2FbK0CjgNG%2BJgSB1DE%2FJ50aDkdg%2Bb58Quzvwj1KvfvmEjzMgtAlZW4ZIGNDOaFy8Ow112o4%2FtpL3LiipiWXkdhjXxPKBNdPb05DMRlwA4kNI%2BGEYYVQZ7wxJjFLVR0X7H%2FhyHf7m1zFd%2FeN%2BfGbzzNOAcpw8Bz7%2BcWhX%2F2r3bCFoc4qR4WOKz3kv932LSF%2FovLfmskTeEnbipLBfb1WWaCiujqysBjJB3da%2Flyr9JL1kJSsW0I7T7GhDu3D2L0TJ3eJEjSaBxuUJwn%2B8QXSaoXkXJX9AOcTsyxYvyKZ7tv%2Bfh0dUb%2B9pJ8I%3D&X-Amz-Algorithm=AWS4-HMAC-SHA256&X-Amz-Date=20231226T180000Z&X-Amz-SignedHeaders=host&X-Amz-Expires=10800&X-Amz-Credential=ASIAUMDY6U44UCHTYQE5%2F20231226%2Fus-gov-west-1%2Fs3%2Faws4_request&X-Amz-Signature=599e132c9cc637522de224d1589932a1ac272df37738908bf295965554a877d5)   **Share My Health Data (SMHD) app:**   - [Share My Health Data \| VA Mobile](https://mobile.va.gov/app/share-my-health-data) |
| **8.3: Measure PGHD, Vitals & Health Indices** | **Summary #11^1,4^:** When a Veteran enters vitals (BP, HR) to **Annie App for Veterans,** these data and other activities are generated into a dashboard in which you can view in the **Annie app for Clinicians.** You can also use the **Annie app for Clinicians** to track Veteran-specific health data like bladder and bowel care. | - <https://effectivehealthcare.ahrq.gov/sites/default/files/pdf/health-data-mapping-technicalbrief.pdf> - [Patient-Generated Health Data – Patient-Generated Health... (blackboard.com)](https://vaots.blackboard.com/webapps/blackboard/execute/modulepage/view?course_id=_2774_1&cmp_tab_id=_3774_1&mode=view) | **Annie App for Veterans (app):**   - [Annie For Veterans \| VA Mobile](https://mobile.va.gov/app/annie-app-veterans)   **Annie app for Clinicians (app):**   - [Annie For Clinicians \| VA Mobile](https://mobile.va.gov/app/annie-app-clinicians) - [Annie app for Clinicians: Session One – YouTube](https://www.youtube.com/watch?v=h0Qt10Oq8ss) - [Annie App – Annie App Community (blackboard.com)](https://vaots.blackboard.com/webapps/blackboard/execute/modulepage/view?course_id=_2548_1&cmp_tab_id=_3324_1&mode=view) |
|  | **Summary #42^1,4,5^:** Monitored health information can be gathered and acquired of variety of ways. Patient Generated Health Data [PGHD] can be obtained asynchronously through equipment such as a wearable health tracking device **(fitBit, Apple Watch), Pulse oximeter, glucometer, digital scale, or blood pressure monitor.** Veterans can independently input monitored health indices using apps such as **CBT-i Coach app, Annie app for Veterans, and cardiac monitoring devices (i.e. Alivecor app, Zio patch).** For synchronous collection of monitored health data during an appointment, additional VHR, such as the **3D Camera** used for monitoring wounds, can be invaluable*.* | - [Remote Patient Monitoring-Home Telehealth – Remote ... (blackboard.com)](https://vaots.blackboard.com/webapps/blackboard/execute/modulepage/view?course_id=_2686_1&cmp_tab_id=_3628_1&mode=view) - [Remote Patient Monitoring - Home Telehealth Manual (blackboard.com)](https://vaots.blackboard.com/bbcswebdav/library/LibraryContent/RPM-HT/rpm_ht_manual/rpm-ht.html) - [Patient-Generated Health Data – Patient-Generated Health... (blackboard.com)](https://vaots.blackboard.com/webapps/blackboard/execute/modulepage/view?course_id=_2774_1&cmp_tab_id=_3774_1&mode=view) - [Virtual Care Manager - Patient Generated Data Guide](https://vaots.blackboard.com/bbcswebdav/xid-1218654_1) - [VA Video Connect (VVC) – VA Video Connect (VVC... (blackboard.com)](https://vaots.blackboard.com/webapps/blackboard/execute/modulepage/view?course_id=_2591_1&cmp_tab_id=_3396_1&mode=view) - [Cardiology Wearables Use Cases (blackboard.com)](https://vaots.blackboard.com/bbcswebdav/library/LibraryContent/Cardiology/Cardiology%20Wearables%20Use%20Cases.pdf) - [Technical Brief 38: Automated-Entry Patient-Generated Health Data for Chronic Conditions: The Evidence on Health Outcomes (ahrq.gov)](https://effectivehealthcare.ahrq.gov/sites/default/files/pdf/health-data-mapping-technicalbrief.pdf)   **Scoping Review for Sensor, Wearable, and Remote Patient Monitoring Competencies for Clinical Care & Training:**   - [Sensor, Wearable, and Remote Patient Monitoring Competencies for Clinical Care and Training: Scoping Review \| SpringerLink](https://link.springer.com/article/10.1007/s41347-020-00190-3) | **Pulse Oximeter:**   - [1226744 (amazonaws.com)](https://learn-us-gov-west-1-prod-fleet01-xythos.s3.us-gov-west-1.amazonaws.com/612eabb1bae6a/1226744?response-cache-control=private%2C%20max-age%3D10800&response-content-disposition=inline%3B%20filename%2A%3DUTF-8%27%27VVC%2520Peripheral%2520Pairing%2520Guide%2520Pulse%2520Oximeter.pdf&response-content-type=application%2Fpdf&X-Amz-Security-Token=FwoDYXdzEI%2F%2F%2F%2F%2F%2F%2F%2F%2F%2F%2FwEaDFCZGOlKZL9Gl1wfpiLkBN7WZx3ou1W58W5Y4NGgAAWBaSP1%2BTxJm8ftHpasVKz8romt2kT7quGI7VYNBio9Wf6gdURlPqmT5E3Omi2zzGtPXSj35m0JxFT5CP74CYkTyPzC65W0K%2F4lh2c5CLAJwm2cYsC5n7%2F1zholgnOn8YCq%2BZbzTB2C1b1vOhX1aJGYGgcX%2FTqVWA%2B6%2FO%2Bc1aYN0VZfh5MnDAvnEBfq3XkcmDVBMOpyTvIjZ2SmegpCSEqV4sAdfsd0ODS5OW0FKayWsmhcov%2BAQFqyGK8vWgsbnr7crgok0ZDTroSVmQHIpyWI8cx6y8cwEMZM9mDn7jCHMERzquFB5enFwxwnqo8AfueUBHUwc1KQl2INZIzz4QJHnQjFi%2BEIsOgjugXTObG%2FksT1GeKzzTfKhPd7vKceMI8fvC0B75%2FKvLOs%2FGQ6Khw0aP%2F7AEY2QoP9rI3AxNs6ypjAAQIqUuYJBGpPCSeRaG8uZabYT8SOZvVWwlEtd36BvkfMzAVjPqZ%2F2D%2BV%2Bf9qaeZH312l9tnIpGiyYMB7ZQM8B%2FWJT0PKzLTE8ttYTkwmNGWhjM2H%2BDnFsJ%2Fy30wlEdd7m8RVzc8%2FoRcHz4MiE%2FK7JFvlv%2FbK0CjgNG%2BJgSB1DE%2FJ50aDkdg%2Bb58Quzvwj1KvfvmEjzMgtAlZW4ZIGNDOaFy8Ow112o4%2FtpL3LiipiWXkdhjXxPKBNdPb05DMRlwA4kNI%2BGEYYVQZ7wxJjFLVR0X7H%2FhyHf7m1zFd%2FeN%2BfGbzzNOAcpw8Bz7%2BcWhX%2F2r3bCFoc4qR4WOKz3kv932LSF%2FovLfmskTeEnbipLBfb1WWaCiujqysBjJB3da%2Flyr9JL1kJSsW0I7T7GhDu3D2L0TJ3eJEjSaBxuUJwn%2B8QXSaoXkXJX9AOcTsyxYvyKZ7tv%2Bfh0dUb%2B9pJ8I%3D&X-Amz-Algorithm=AWS4-HMAC-SHA256&X-Amz-Date=20231226T180000Z&X-Amz-SignedHeaders=host&X-Amz-Expires=10800&X-Amz-Credential=ASIAUMDY6U44UCHTYQE5%2F20231226%2Fus-gov-west-1%2Fs3%2Faws4_request&X-Amz-Signature=599e132c9cc637522de224d1589932a1ac272df37738908bf295965554a877d5)   **Cardiac monitoring device:**   - [KardiaMobile Card is Available for Veterans \| Kardia](https://www.kardia.com/gov)   **Sleep Clinic Resources:**   - [Sleep-Clinic Workflow](https://vaots.blackboard.com/bbcswebdav/xid-567502_1) - [Sleep Program – Sleep Program Community (blackboard.com)](https://vaots.blackboard.com/webapps/blackboard/execute/modulepage/view?course_id=_2554_1&cmp_tab_id=_3336_1&mode=view) - [Sleep Intranet Resource Center](https://vaww.telehealth.va.gov/clinic/tsleep/index.asp)   **Blood Pressure Monitor:**   - [569283 (amazonaws.com)](https://learn-us-gov-west-1-prod-fleet01-xythos.s3.us-gov-west-1.amazonaws.com/612eabb1bae6a/569283?response-cache-control=private%2C%20max-age%3D10800&response-content-disposition=inline%3B%20filename%2A%3DUTF-8%27%27VVC%2520Peripheral%2520Pairing%2520Guide%2520Blood%2520Pressure.pdf&response-content-type=application%2Fpdf&X-Amz-Security-Token=FwoDYXdzEKb%2F%2F%2F%2F%2F%2F%2F%2F%2F%2FwEaDNihyzG6BBJlm7g3RSLkBDWBa0eQrvjTRXFLZ%2BwSo0GkYtWskMLQ8zmO4mhKnKFaOD2ykn%2FDRPWlnm%2BGluvTUL9g2mmOnTQXkipUWWOsocG%2Fn3HXRgOsLAHMoN%2B7ZepEzlYZhSwS1JDoOfrPIK6MsA%2BJ280pwu47T2YwJxmQlS6IO4Bu4%2F%2BF2%2BztY0oB67q%2Fihc2UB6FKI2mmaLFG0N4zALTvF42vDKLmj%2BtnRbB6VGYCiZi1stzI0ac4ePMSdvqewi6EvcGmpFE%2Bo4nXRD%2BtnHb2bEFulCmMNAh5gsKvytBYInE%2BkFXkOmiokGy%2FQYwnL%2FEntDtohbVWsLgxwjSQq5Iv%2BDA7MIRCWbNI7a6s9fu%2FUZVSYogvxu1AhD9I1HQoDpaamiey1ByYqbyIGzLFpLso0sB%2FPK9iOkjmA8e5%2BMZmbePeNyjZjh3YXYNMTYZa669ZIYame8cjRsaqlDAUnw39GHjhZAAsiJEmLbc69m%2FEvIaJaaeGNZnMnSx9T6QQAtE3WeEtaVGXoqYB8%2FkcSnHRTOW%2BUxXOU09XMd%2F5mOaX8xOrPRYalIPPii3d4JAaEJiiQm%2BOIW%2BHKerWLiEPPbJRjFLkcb83UBvRQ%2F0i7qY6%2FW56%2FcMNsIRgwtTvSOoHY%2FkECJoAKl8eQMrTgIjY9zZd6hDhSFk5p%2BK3mzZOCIRS2RdJYtQ6K5IZemc81pwdKNj%2FBEia8GoM0EYHpR004qtnatpY3BYi%2Fa9H0n6xhnafw3F9ggO7TNo%2FO2Hs3qlfkmivXYkqX2nXwEE2NzCOzIN979RlCP9P8OcFWkdige75XPfVK7pO5Og8%2Bi5xfMcF3l4iCjikLGsBjJBGvkKrrZkpHMqODM2DzouIsUJlFAminwrt%2BO0qWhVDKzWcW7wFlG7xv4EaAu9Kpe380FXITNhz97VbGWfKUnzR%2Bo%3D&X-Amz-Algorithm=AWS4-HMAC-SHA256&X-Amz-Date=20231227T163000Z&X-Amz-SignedHeaders=host&X-Amz-Expires=10800&X-Amz-Credential=ASIAUMDY6U44RUDMCFOA%2F20231227%2Fus-gov-west-1%2Fs3%2Faws4_request&X-Amz-Signature=0a5513cba3a39b2a5aa8519e38a85e1c020e63504500cb11abd0842ed86879ce)   **FitBit/Apple Watch:**   - [Digital Health Innovations - VA-Fitbit-HowToParticipate-1P.pdf - All Documents (sharepoint.com)](https://dvagov.sharepoint.com/sites/VHAV1NECIE/DHI/Shared%20Documents/Forms/AllItems.aspx?id=%2Fsites%2FVHAV1NECIE%2FDHI%2FShared%20Documents%2FVA%2DFitbit%2DHowToParticipate%2D1P%2Epdf&parent=%2Fsites%2FVHAV1NECIE%2FDHI%2FShared%20Documents) - [Digital Health Innovations - Fitbit Sense - Getting Started Guide - Veterans.pdf - All Documents (sharepoint.com)](https://dvagov.sharepoint.com/sites/VHAV1NECIE/DHI/Shared%20Documents/Forms/AllItems.aspx?id=%2Fsites%2FVHAV1NECIE%2FDHI%2FShared%20Documents%2FFitbit%20Sense%20%2D%20Getting%20Started%20Guide%20%2D%20Veterans%2Epdf&parent=%2Fsites%2FVHAV1NECIE%2FDHI%2FShared%20Documents)   **Peripheral Devices:**   - [Peripheral Devices \| During the VA Video Connect Visit \| VHA Telehealth Services Intranet](https://vaww.telehealth.va.gov/pgm/vvc/during/peripherals.asp)   **3-D camera:**   - [Library: TeleWound Care – Blackboard Learn](https://vaots.blackboard.com/webapps/cmsmain/webui/library/LibraryContent/Outreach/TeleWound%20Care?action=frameset&subaction=view&uniq=-8bswn3)   **Annie App for Veterans:**   - [Annie For Veterans \| VA Mobile](https://mobile.va.gov/app/annie-app-veterans)   **CBT-i Coach:**   - [CBT-i Coach \| VA Mobile](https://mobile.va.gov/app/cbt-i-coach)   **VA App Store:**   - [VA App Store \| VA Mobile](https://mobile.va.gov/appstore/) |
|  | **Summary #49^4,5^:** You can use the **CBT-i Coach app** and the data from **Apple Watch/FitBit** to review and track weekly sleep cycles and can adjust the schedule when needed. You can utilize information from the service connection disability rating and vitals generated in both **CPRS** and **CBT-i** Coach to ensure Veterans are improving over the course of time together with the provider. | - <https://effectivehealthcare.ahrq.gov/sites/default/files/pdf/health-data-mapping-technicalbrief.pdf> - [Patient-Generated Health Data – Patient-Generated Health... (blackboard.com)](https://vaots.blackboard.com/webapps/blackboard/execute/modulepage/view?course_id=_2774_1&cmp_tab_id=_3774_1&mode=view) | **CBT-I Coach:**   - [CBT-i Coach \| VA Mobile](https://mobile.va.gov/app/cbt-i-coach)   **FitBit/Apple Watch:**   - [Digital Health Innovations - VA-Fitbit-HowToParticipate-1P.pdf - All Documents (sharepoint.com)](https://dvagov.sharepoint.com/sites/VHAV1NECIE/DHI/Shared%20Documents/Forms/AllItems.aspx?id=%2Fsites%2FVHAV1NECIE%2FDHI%2FShared%20Documents%2FVA%2DFitbit%2DHowToParticipate%2D1P%2Epdf&parent=%2Fsites%2FVHAV1NECIE%2FDHI%2FShared%20Documents) - [Digital Health Innovations - Fitbit Sense - Getting Started Guide - Veterans.pdf - All Documents (sharepoint.com)](https://dvagov.sharepoint.com/sites/VHAV1NECIE/DHI/Shared%20Documents/Forms/AllItems.aspx?id=%2Fsites%2FVHAV1NECIE%2FDHI%2FShared%20Documents%2FFitbit%20Sense%20%2D%20Getting%20Started%20Guide%20%2D%20Veterans%2Epdf&parent=%2Fsites%2FVHAV1NECIE%2FDHI%2FShared%20Documents) |
|  | **Summary #50^1,2,4^:** Through the telehealth program, you can monitor health indices such as sugar levels and can use a **Blood Pressure Monitor** to measure and automatically populate vital signs directly into **CPRS** to avoid errors. Telehealth vitals can be compiled into a note that tracks progress over time, which is saved on **CPRS**. You can also review the **Care Assessment Need (CAN) Risk Assessment** in **CPRS.** | - [Patient-Generated Health Data – Patient-Generated Health... (blackboard.com)](https://vaots.blackboard.com/webapps/blackboard/execute/modulepage/view?course_id=_2774_1&cmp_tab_id=_3774_1&mode=view) - <https://effectivehealthcare.ahrq.gov/sites/default/files/pdf/health-data-mapping-technicalbrief.pdf> | **Blood Pressure Monitor:**   - [569283 (amazonaws.com)](https://learn-us-gov-west-1-prod-fleet01-xythos.s3.us-gov-west-1.amazonaws.com/612eabb1bae6a/569283?response-cache-control=private%2C%20max-age%3D10800&response-content-disposition=inline%3B%20filename%2A%3DUTF-8%27%27VVC%2520Peripheral%2520Pairing%2520Guide%2520Blood%2520Pressure.pdf&response-content-type=application%2Fpdf&X-Amz-Security-Token=FwoDYXdzEKb%2F%2F%2F%2F%2F%2F%2F%2F%2F%2FwEaDNihyzG6BBJlm7g3RSLkBDWBa0eQrvjTRXFLZ%2BwSo0GkYtWskMLQ8zmO4mhKnKFaOD2ykn%2FDRPWlnm%2BGluvTUL9g2mmOnTQXkipUWWOsocG%2Fn3HXRgOsLAHMoN%2B7ZepEzlYZhSwS1JDoOfrPIK6MsA%2BJ280pwu47T2YwJxmQlS6IO4Bu4%2F%2BF2%2BztY0oB67q%2Fihc2UB6FKI2mmaLFG0N4zALTvF42vDKLmj%2BtnRbB6VGYCiZi1stzI0ac4ePMSdvqewi6EvcGmpFE%2Bo4nXRD%2BtnHb2bEFulCmMNAh5gsKvytBYInE%2BkFXkOmiokGy%2FQYwnL%2FEntDtohbVWsLgxwjSQq5Iv%2BDA7MIRCWbNI7a6s9fu%2FUZVSYogvxu1AhD9I1HQoDpaamiey1ByYqbyIGzLFpLso0sB%2FPK9iOkjmA8e5%2BMZmbePeNyjZjh3YXYNMTYZa669ZIYame8cjRsaqlDAUnw39GHjhZAAsiJEmLbc69m%2FEvIaJaaeGNZnMnSx9T6QQAtE3WeEtaVGXoqYB8%2FkcSnHRTOW%2BUxXOU09XMd%2F5mOaX8xOrPRYalIPPii3d4JAaEJiiQm%2BOIW%2BHKerWLiEPPbJRjFLkcb83UBvRQ%2F0i7qY6%2FW56%2FcMNsIRgwtTvSOoHY%2FkECJoAKl8eQMrTgIjY9zZd6hDhSFk5p%2BK3mzZOCIRS2RdJYtQ6K5IZemc81pwdKNj%2FBEia8GoM0EYHpR004qtnatpY3BYi%2Fa9H0n6xhnafw3F9ggO7TNo%2FO2Hs3qlfkmivXYkqX2nXwEE2NzCOzIN979RlCP9P8OcFWkdige75XPfVK7pO5Og8%2Bi5xfMcF3l4iCjikLGsBjJBGvkKrrZkpHMqODM2DzouIsUJlFAminwrt%2BO0qWhVDKzWcW7wFlG7xv4EaAu9Kpe380FXITNhz97VbGWfKUnzR%2Bo%3D&X-Amz-Algorithm=AWS4-HMAC-SHA256&X-Amz-Date=20231227T163000Z&X-Amz-SignedHeaders=host&X-Amz-Expires=10800&X-Amz-Credential=ASIAUMDY6U44RUDMCFOA%2F20231227%2Fus-gov-west-1%2Fs3%2Faws4_request&X-Amz-Signature=0a5513cba3a39b2a5aa8519e38a85e1c020e63504500cb11abd0842ed86879ce)   **CPRS:**   - [CPRS Technical Manual (va.gov)](https://www.va.gov/vdl/documents/Clinical/Comp_Patient_Recrd_Sys_(CPRS)/cprslmtm.pdf)   **CAN:**   - [Study gauges VA providers' views on predictive-analytics tool that assesses patient risk](https://www.research.va.gov/currents/1119-Researchers-study-VA-patient-data-tool-that-assesses-probability-of-hospitalization-and-death.cfm) - [713-notes.pdf (va.gov)](https://www.hsrd.research.va.gov/for_researchers/cyber_seminars/archives/713-notes.pdf) |
| **8.4:Veteran-Based VHR** | **Summary #11^1,4^:** When a Veteran enters vitals (BP, HR) to **Annie App for Veterans,** these data and other activities are generated into a dashboard in which you can view in the **Annie app for Clinicians.** You can also use the **Annie app for Clinicians** to track Veteran-specific health data like bladder and bowel care. | - <https://effectivehealthcare.ahrq.gov/sites/default/files/pdf/health-data-mapping-technicalbrief.pdf> - [Patient-Generated Health Data – Patient-Generated Health... (blackboard.com)](https://vaots.blackboard.com/webapps/blackboard/execute/modulepage/view?course_id=_2774_1&cmp_tab_id=_3774_1&mode=view) | **Annie App for Veterans (app):**   - [Annie For Veterans \| VA Mobile](https://mobile.va.gov/app/annie-app-veterans)   **Annie app for Clinicians (app):**   - [Annie For Clinicians \| VA Mobile](https://mobile.va.gov/app/annie-app-clinicians) - [Annie app for Clinicians: Session One – YouTube](https://www.youtube.com/watch?v=h0Qt10Oq8ss) - [Annie App – Annie App Community (blackboard.com)](https://vaots.blackboard.com/webapps/blackboard/execute/modulepage/view?course_id=_2548_1&cmp_tab_id=_3324_1&mode=view) |
|  | **Summary #19^5^:** Provider can use apps such as **CBT-i-Coach app, PTSD Coach app, CPT Coach app** and **Insomnia Coach app** to use during post-appointment for providing treatment and to manage and track PGHD. | - [Patient-Generated Health Data – Patient-Generated Health... (blackboard.com)](https://vaots.blackboard.com/webapps/blackboard/execute/modulepage/view?course_id=_2774_1&cmp_tab_id=_3774_1&mode=view) | **CBT-i (Cognitive Behavioral Therapy for Insomnia) Coach App:**   - [CBT-i Coach \| VA Mobile](https://mobile.va.gov/app/cbt-i-coach)   **PTSD (Post Traumatic Stress Disorder) Coach App:**   - [PTSD Coach \| VA Mobile](https://mobile.va.gov/app/ptsd-coach)   **CPT (Cognitive Processing Therapy) Coach App:**   - [CPT Coach \| VA Mobile](https://mobile.va.gov/app/cpt-coach)   **Insomnia Coach App:**   - [Insomnia Coach \| VA Mobile](https://mobile.va.gov/app/insomnia-coach)   **VA App Store:**   - [VA App Store \| VA Mobile](https://mobile.va.gov/appstore/) |
|  | **Summary #30^1,2^:** Staff and providers encourage Veterans to use **SM (MHV)** to communicate and to use **VA Mobile apps** and tracking devices such as **FitBit** or **Apple Watch** for tracking communication and health data. | - [MHV_SM_HCT_User_Manual_August_2023.pdf (va.gov)](https://vaww.va.gov/MYHEALTHEVET/docs/secure_messaging/MHV_SM_HCT_User_Manual_August_2023.pdf) - [Peripheral Devices \| During the VA Video Connect Visit \| VHA Telehealth Services Intranet](https://vaww.telehealth.va.gov/pgm/vvc/during/peripherals.asp) | **VA Mobile Apps:**   - [VA Mobile Health Practice Guide](https://connectedcare.va.gov/sites/default/files/2021-10/va-mobile-health-practice-guide.pdf)   **SM (MHV):**   - <https://www.myhealth.va.gov/mhv-portal-web/home> - [My HealtheVet Resources – My HealtheVet Resources](https://vaots.blackboard.com/webapps/blackboard/execute/modulepage/view?course_id=_3174_1&cmp_tab_id=_4591_1&mode=view) - [Secure Messaging Through My HealtheVet – My HealtheVet Product (va.gov)](https://vaww.va.gov/MYHEALTHEVET/Secure_Messaging.asp)   **FitBit/Apple Watch:**   - [Digital Health Innovations - VA-Fitbit-HowToParticipate-1P.pdf - All Documents (sharepoint.com)](https://dvagov.sharepoint.com/sites/VHAV1NECIE/DHI/Shared%20Documents/Forms/AllItems.aspx?id=%2Fsites%2FVHAV1NECIE%2FDHI%2FShared%20Documents%2FVA%2DFitbit%2DHowToParticipate%2D1P%2Epdf&parent=%2Fsites%2FVHAV1NECIE%2FDHI%2FShared%20Documents) - [Digital Health Innovations - Fitbit Sense - Getting Started Guide - Veterans.pdf - All Documents (sharepoint.com)](https://dvagov.sharepoint.com/sites/VHAV1NECIE/DHI/Shared%20Documents/Forms/AllItems.aspx?id=%2Fsites%2FVHAV1NECIE%2FDHI%2FShared%20Documents%2FFitbit%20Sense%20%2D%20Getting%20Started%20Guide%20%2D%20Veterans%2Epdf&parent=%2Fsites%2FVHAV1NECIE%2FDHI%2FShared%20Documents) |
|  | **Summary #38^3,4^:** When assessing the health of a Veteran, a significant amount of information needed is obtained by the Veteran. When conducting the appointment virtually, the Veteran can share their PGHD using a variety of VA mobile apps, such as **Annie app for Veterans.** | - <https://effectivehealthcare.ahrq.gov/sites/default/files/pdf/health-data-mapping-technicalbrief.pdf> - [Remote Patient Monitoring-Home Telehealth – Remote ... (blackboard.com)](https://vaots.blackboard.com/webapps/blackboard/execute/modulepage/view?course_id=_2686_1&cmp_tab_id=_3628_1&mode=view) - [Patient-Generated Health Data – Patient-Generated Health... (blackboard.com)](https://vaots.blackboard.com/webapps/blackboard/execute/modulepage/view?course_id=_2774_1&cmp_tab_id=_3774_1&mode=view) | **VA Mobile Apps:**   - [App Store \| VA Mobile](https://mobile.va.gov/appstore)   **Annie App for Veterans:**   - [Annie App for Veterans \| VA Mobile](https://mobile.va.gov/app/annie-app-veterans) |
|  | **Summary #42^1,4,5^:** Monitored health information can be gathered and acquired of variety of ways. Patient Generated Health Data [PGHD] can be obtained asynchronously through equipment such as a wearable health tracking device **(fitBit, Apple Watch), Pulse oximeter, glucometer, digital scale, or blood pressure monitor.** Veterans can independently input monitored health indices using apps such as **CBT-i Coach app, Annie app for Veterans, and cardiac monitoring devices (i.e. Alivecor app, Zio patch).** For synchronous collection of monitored health data during an appointment, additional VHR, such as the **3D Camera** used for monitoring wounds, can be invaluable*.* | - [Remote Patient Monitoring-Home Telehealth – Remote ... (blackboard.com)](https://vaots.blackboard.com/webapps/blackboard/execute/modulepage/view?course_id=_2686_1&cmp_tab_id=_3628_1&mode=view) - [Remote Patient Monitoring - Home Telehealth Manual (blackboard.com)](https://vaots.blackboard.com/bbcswebdav/library/LibraryContent/RPM-HT/rpm_ht_manual/rpm-ht.html) - [Patient-Generated Health Data – Patient-Generated Health... (blackboard.com)](https://vaots.blackboard.com/webapps/blackboard/execute/modulepage/view?course_id=_2774_1&cmp_tab_id=_3774_1&mode=view) - [Virtual Care Manager - Patient Generated Data Guide](https://vaots.blackboard.com/bbcswebdav/xid-1218654_1) - [VA Video Connect (VVC) – VA Video Connect (VVC... (blackboard.com)](https://vaots.blackboard.com/webapps/blackboard/execute/modulepage/view?course_id=_2591_1&cmp_tab_id=_3396_1&mode=view) - [Cardiology Wearables Use Cases (blackboard.com)](https://vaots.blackboard.com/bbcswebdav/library/LibraryContent/Cardiology/Cardiology%20Wearables%20Use%20Cases.pdf) - [Technical Brief 38: Automated-Entry Patient-Generated Health Data for Chronic Conditions: The Evidence on Health Outcomes (ahrq.gov)](https://effectivehealthcare.ahrq.gov/sites/default/files/pdf/health-data-mapping-technicalbrief.pdf)   **Scoping Review for Sensor, Wearable, and Remote Patient Monitoring Competencies for Clinical Care & Training:**   - [Sensor, Wearable, and Remote Patient Monitoring Competencies for Clinical Care and Training: Scoping Review \| SpringerLink](https://link.springer.com/article/10.1007/s41347-020-00190-3) | **Pulse Oximeter:**   - [1226744 (amazonaws.com)](https://learn-us-gov-west-1-prod-fleet01-xythos.s3.us-gov-west-1.amazonaws.com/612eabb1bae6a/1226744?response-cache-control=private%2C%20max-age%3D10800&response-content-disposition=inline%3B%20filename%2A%3DUTF-8%27%27VVC%2520Peripheral%2520Pairing%2520Guide%2520Pulse%2520Oximeter.pdf&response-content-type=application%2Fpdf&X-Amz-Security-Token=FwoDYXdzEI%2F%2F%2F%2F%2F%2F%2F%2F%2F%2F%2FwEaDFCZGOlKZL9Gl1wfpiLkBN7WZx3ou1W58W5Y4NGgAAWBaSP1%2BTxJm8ftHpasVKz8romt2kT7quGI7VYNBio9Wf6gdURlPqmT5E3Omi2zzGtPXSj35m0JxFT5CP74CYkTyPzC65W0K%2F4lh2c5CLAJwm2cYsC5n7%2F1zholgnOn8YCq%2BZbzTB2C1b1vOhX1aJGYGgcX%2FTqVWA%2B6%2FO%2Bc1aYN0VZfh5MnDAvnEBfq3XkcmDVBMOpyTvIjZ2SmegpCSEqV4sAdfsd0ODS5OW0FKayWsmhcov%2BAQFqyGK8vWgsbnr7crgok0ZDTroSVmQHIpyWI8cx6y8cwEMZM9mDn7jCHMERzquFB5enFwxwnqo8AfueUBHUwc1KQl2INZIzz4QJHnQjFi%2BEIsOgjugXTObG%2FksT1GeKzzTfKhPd7vKceMI8fvC0B75%2FKvLOs%2FGQ6Khw0aP%2F7AEY2QoP9rI3AxNs6ypjAAQIqUuYJBGpPCSeRaG8uZabYT8SOZvVWwlEtd36BvkfMzAVjPqZ%2F2D%2BV%2Bf9qaeZH312l9tnIpGiyYMB7ZQM8B%2FWJT0PKzLTE8ttYTkwmNGWhjM2H%2BDnFsJ%2Fy30wlEdd7m8RVzc8%2FoRcHz4MiE%2FK7JFvlv%2FbK0CjgNG%2BJgSB1DE%2FJ50aDkdg%2Bb58Quzvwj1KvfvmEjzMgtAlZW4ZIGNDOaFy8Ow112o4%2FtpL3LiipiWXkdhjXxPKBNdPb05DMRlwA4kNI%2BGEYYVQZ7wxJjFLVR0X7H%2FhyHf7m1zFd%2FeN%2BfGbzzNOAcpw8Bz7%2BcWhX%2F2r3bCFoc4qR4WOKz3kv932LSF%2FovLfmskTeEnbipLBfb1WWaCiujqysBjJB3da%2Flyr9JL1kJSsW0I7T7GhDu3D2L0TJ3eJEjSaBxuUJwn%2B8QXSaoXkXJX9AOcTsyxYvyKZ7tv%2Bfh0dUb%2B9pJ8I%3D&X-Amz-Algorithm=AWS4-HMAC-SHA256&X-Amz-Date=20231226T180000Z&X-Amz-SignedHeaders=host&X-Amz-Expires=10800&X-Amz-Credential=ASIAUMDY6U44UCHTYQE5%2F20231226%2Fus-gov-west-1%2Fs3%2Faws4_request&X-Amz-Signature=599e132c9cc637522de224d1589932a1ac272df37738908bf295965554a877d5)   **Cardiac monitoring device:**   - [KardiaMobile Card is Available for Veterans \| Kardia](https://www.kardia.com/gov)   **Sleep Clinic Resources:**   - [Sleep-Clinic Workflow](https://vaots.blackboard.com/bbcswebdav/xid-567502_1) - [Sleep Program – Sleep Program Community (blackboard.com)](https://vaots.blackboard.com/webapps/blackboard/execute/modulepage/view?course_id=_2554_1&cmp_tab_id=_3336_1&mode=view) - [Sleep Intranet Resource Center](https://vaww.telehealth.va.gov/clinic/tsleep/index.asp)   **Blood Pressure Monitor:**   - [569283 (amazonaws.com)](https://learn-us-gov-west-1-prod-fleet01-xythos.s3.us-gov-west-1.amazonaws.com/612eabb1bae6a/569283?response-cache-control=private%2C%20max-age%3D10800&response-content-disposition=inline%3B%20filename%2A%3DUTF-8%27%27VVC%2520Peripheral%2520Pairing%2520Guide%2520Blood%2520Pressure.pdf&response-content-type=application%2Fpdf&X-Amz-Security-Token=FwoDYXdzEKb%2F%2F%2F%2F%2F%2F%2F%2F%2F%2FwEaDNihyzG6BBJlm7g3RSLkBDWBa0eQrvjTRXFLZ%2BwSo0GkYtWskMLQ8zmO4mhKnKFaOD2ykn%2FDRPWlnm%2BGluvTUL9g2mmOnTQXkipUWWOsocG%2Fn3HXRgOsLAHMoN%2B7ZepEzlYZhSwS1JDoOfrPIK6MsA%2BJ280pwu47T2YwJxmQlS6IO4Bu4%2F%2BF2%2BztY0oB67q%2Fihc2UB6FKI2mmaLFG0N4zALTvF42vDKLmj%2BtnRbB6VGYCiZi1stzI0ac4ePMSdvqewi6EvcGmpFE%2Bo4nXRD%2BtnHb2bEFulCmMNAh5gsKvytBYInE%2BkFXkOmiokGy%2FQYwnL%2FEntDtohbVWsLgxwjSQq5Iv%2BDA7MIRCWbNI7a6s9fu%2FUZVSYogvxu1AhD9I1HQoDpaamiey1ByYqbyIGzLFpLso0sB%2FPK9iOkjmA8e5%2BMZmbePeNyjZjh3YXYNMTYZa669ZIYame8cjRsaqlDAUnw39GHjhZAAsiJEmLbc69m%2FEvIaJaaeGNZnMnSx9T6QQAtE3WeEtaVGXoqYB8%2FkcSnHRTOW%2BUxXOU09XMd%2F5mOaX8xOrPRYalIPPii3d4JAaEJiiQm%2BOIW%2BHKerWLiEPPbJRjFLkcb83UBvRQ%2F0i7qY6%2FW56%2FcMNsIRgwtTvSOoHY%2FkECJoAKl8eQMrTgIjY9zZd6hDhSFk5p%2BK3mzZOCIRS2RdJYtQ6K5IZemc81pwdKNj%2FBEia8GoM0EYHpR004qtnatpY3BYi%2Fa9H0n6xhnafw3F9ggO7TNo%2FO2Hs3qlfkmivXYkqX2nXwEE2NzCOzIN979RlCP9P8OcFWkdige75XPfVK7pO5Og8%2Bi5xfMcF3l4iCjikLGsBjJBGvkKrrZkpHMqODM2DzouIsUJlFAminwrt%2BO0qWhVDKzWcW7wFlG7xv4EaAu9Kpe380FXITNhz97VbGWfKUnzR%2Bo%3D&X-Amz-Algorithm=AWS4-HMAC-SHA256&X-Amz-Date=20231227T163000Z&X-Amz-SignedHeaders=host&X-Amz-Expires=10800&X-Amz-Credential=ASIAUMDY6U44RUDMCFOA%2F20231227%2Fus-gov-west-1%2Fs3%2Faws4_request&X-Amz-Signature=0a5513cba3a39b2a5aa8519e38a85e1c020e63504500cb11abd0842ed86879ce)   **FitBit/Apple Watch:**   - [Digital Health Innovations - VA-Fitbit-HowToParticipate-1P.pdf - All Documents (sharepoint.com)](https://dvagov.sharepoint.com/sites/VHAV1NECIE/DHI/Shared%20Documents/Forms/AllItems.aspx?id=%2Fsites%2FVHAV1NECIE%2FDHI%2FShared%20Documents%2FVA%2DFitbit%2DHowToParticipate%2D1P%2Epdf&parent=%2Fsites%2FVHAV1NECIE%2FDHI%2FShared%20Documents) - [Digital Health Innovations - Fitbit Sense - Getting Started Guide - Veterans.pdf - All Documents (sharepoint.com)](https://dvagov.sharepoint.com/sites/VHAV1NECIE/DHI/Shared%20Documents/Forms/AllItems.aspx?id=%2Fsites%2FVHAV1NECIE%2FDHI%2FShared%20Documents%2FFitbit%20Sense%20%2D%20Getting%20Started%20Guide%20%2D%20Veterans%2Epdf&parent=%2Fsites%2FVHAV1NECIE%2FDHI%2FShared%20Documents)   **Peripheral Devices:**   - [Peripheral Devices \| During the VA Video Connect Visit \| VHA Telehealth Services Intranet](https://vaww.telehealth.va.gov/pgm/vvc/during/peripherals.asp)   **3-D camera:**   - [Library: TeleWound Care – Blackboard Learn](https://vaots.blackboard.com/webapps/cmsmain/webui/library/LibraryContent/Outreach/TeleWound%20Care?action=frameset&subaction=view&uniq=-8bswn3)   **Annie App for Veterans:**   - [Annie For Veterans \| VA Mobile](https://mobile.va.gov/app/annie-app-veterans)   **CBT-i Coach:**   - [CBT-i Coach \| VA Mobile](https://mobile.va.gov/app/cbt-i-coach)   **VA App Store:**   - [VA App Store \| VA Mobile](https://mobile.va.gov/appstore/) |
|  |  |  |  |
|  | **Summary #43^1,4^:** The Veteran can request refill of medications 3 different ways: Requests for more refills can be sent to the provider using **SM (MHV)** or- if there are already refills available, they can order them through **VA Prescriptions Refill (MHV)** or use the **Rx Refill app.** | - [498237 (amazonaws.com)](https://learn-us-gov-west-1-prod-fleet01-xythos.s3.us-gov-west-1.amazonaws.com/612eabb1bae6a/498237?response-cache-control=private%2C%20max-age%3D10800&response-content-disposition=inline%3B%20filename%2A%3DUTF-8%27%27VA%2520Prescription%2520Refills%2520and%2520COVID-19.pdf&response-content-type=application%2Fpdf&X-Amz-Security-Token=FwoDYXdzEJP%2F%2F%2F%2F%2F%2F%2F%2F%2F%2FwEaDFx%2FNVtAwHATXzcqniLlBE3diRWWSiKCHkP7isSq0G%2FFyaEDzzByq82ALTS413TW%2Fqwpt77gsKBXxfzgawaUbH2x4s8xOxk4gling%2BnV6BQSLnOcXF71r7KnUSGPgjktE0r3fp5iO1nCcfehw%2B3JU58MpFmS3dFzbiIuRFBDGMWq8m1HeHYFag5ncBwkH96bBVaQMo39kQu13pJfzNGDwDsb0U%2BVIXTwruxZGfmbc90shERXh1bI4LPGYjZuAr71iXQ4hkOWgI9%2BbgdZCCqC%2FgcEtA%2BWng%2F5WFwj%2BddMwT5%2FJyQHnDRbKDLebbvzQn0B0lWD2rg0cDqusXTn6oSO9hmL1uJa1lYKp8GYP4q8f%2BvMdohCqQ9ym5zPEV38yz6VG8uCDc5P4tmxcoHPHT187M9XOT%2BNtx96z1yGun9Rn3SkhTbLc0s5kOwo%2B2jXeCGwsrMP%2BVQZQHi7LipPSeW5VeidUyTADtTfxr5rnBMYqa3chljfWh73g6I0VtGhPvF%2Fr2qxCiLImuOzRW2bNjMZkqn6PcWB3dmplfJzDkkYQ8lCiTGVHh2oEidNWHNVmLObf3cXD7arJgs%2FPRnQWMj22%2Fw5GQimSC6IlfZ92%2B7L4E6IgVO6D0BPdHyvgIkmYZ44KLvqaU85socaCzQkZ2UcZmeV%2BE11UT80AnXa6mAhJ2RDOeBTcphSjZYbJNn7OErHeMrEFHkn1F%2BJkS8yyGO08a%2F%2B0UyUDQjuIeB5fCloThctAWNyJiO%2BL%2F7p5y9bfc6%2FtXM%2FGiTeSVL%2Fjiew1SadjvQ5ad32NxdIvDo8aulIiEN66HbOGJOa4doQqBxxmc3jtmjvdmco8PusrAYyQRwkt1jY2L6%2FqyNZ0m8QiCqm5ewu1cNwwVJfXI6sIfOxZdHZf5xjYtsCZPHq5cgpkF%2FhtmYzCK05V1mnfwEeRJ5p&X-Amz-Algorithm=AWS4-HMAC-SHA256&X-Amz-Date=20231226T210000Z&X-Amz-SignedHeaders=host&X-Amz-Expires=10800&X-Amz-Credential=ASIAUMDY6U444EJKJP5C%2F20231226%2Fus-gov-west-1%2Fs3%2Faws4_request&X-Amz-Signature=c0187abb25cba3dc9e35d7b2458dd3328d9292f3548bfcc9146dde99f742ff39) | **SM (MHV):**   - <https://www.myhealth.va.gov/mhv-portal-web/home>   **VA Prescription Refill (MHV):**   - [Managing Your Prescription Refills Online - My HealtheVet - My HealtheVet (va.gov)](https://www.myhealth.va.gov/managing-your-prescription-refills)   **Rx Refill App:**   - [Rx Refill \| VA Mobile](https://mobile.va.gov/app/rx-refill) |
|  | **Summary #50^4,5^:** You can use the **CBT-i Coach app** and the data from **Apple Watch/FitBit** to review and track weekly sleep cycles and can adjust the schedule when needed. You can utilize information from the service connection disability rating and vitals generated in both **CPRS** and **CBT-i** Coach to ensure Veterans are improving over the course of time together with the provider. | - <https://effectivehealthcare.ahrq.gov/sites/default/files/pdf/health-data-mapping-technicalbrief.pdf> - [Patient-Generated Health Data – Patient-Generated Health... (blackboard.com)](https://vaots.blackboard.com/webapps/blackboard/execute/modulepage/view?course_id=_2774_1&cmp_tab_id=_3774_1&mode=view) | **CBT-I Coach:**   - [CBT-i Coach \| VA Mobile](https://mobile.va.gov/app/cbt-i-coach)   **FitBit/Apple Watch:**   - [Digital Health Innovations - VA-Fitbit-HowToParticipate-1P.pdf - All Documents (sharepoint.com)](https://dvagov.sharepoint.com/sites/VHAV1NECIE/DHI/Shared%20Documents/Forms/AllItems.aspx?id=%2Fsites%2FVHAV1NECIE%2FDHI%2FShared%20Documents%2FVA%2DFitbit%2DHowToParticipate%2D1P%2Epdf&parent=%2Fsites%2FVHAV1NECIE%2FDHI%2FShared%20Documents) - [Digital Health Innovations - Fitbit Sense - Getting Started Guide - Veterans.pdf - All Documents (sharepoint.com)](https://dvagov.sharepoint.com/sites/VHAV1NECIE/DHI/Shared%20Documents/Forms/AllItems.aspx?id=%2Fsites%2FVHAV1NECIE%2FDHI%2FShared%20Documents%2FFitbit%20Sense%20%2D%20Getting%20Started%20Guide%20%2D%20Veterans%2Epdf&parent=%2Fsites%2FVHAV1NECIE%2FDHI%2FShared%20Documents) |
| **9. Medication Management** | | | |
| **9.1 Requesting RX or Treatment by Veteran** | **Summary #43^1,4^:** The Veteran can request refill of medications 3 different ways: Requests for more refills can be sent to the provider using **SM (MHV)** or- if there are already refills available, they can order them through **VA Prescriptions Refill (MHV)** or use the **Rx Refill app.** | - [498237 (amazonaws.com)](https://learn-us-gov-west-1-prod-fleet01-xythos.s3.us-gov-west-1.amazonaws.com/612eabb1bae6a/498237?response-cache-control=private%2C%20max-age%3D10800&response-content-disposition=inline%3B%20filename%2A%3DUTF-8%27%27VA%2520Prescription%2520Refills%2520and%2520COVID-19.pdf&response-content-type=application%2Fpdf&X-Amz-Security-Token=FwoDYXdzEJP%2F%2F%2F%2F%2F%2F%2F%2F%2F%2FwEaDFx%2FNVtAwHATXzcqniLlBE3diRWWSiKCHkP7isSq0G%2FFyaEDzzByq82ALTS413TW%2Fqwpt77gsKBXxfzgawaUbH2x4s8xOxk4gling%2BnV6BQSLnOcXF71r7KnUSGPgjktE0r3fp5iO1nCcfehw%2B3JU58MpFmS3dFzbiIuRFBDGMWq8m1HeHYFag5ncBwkH96bBVaQMo39kQu13pJfzNGDwDsb0U%2BVIXTwruxZGfmbc90shERXh1bI4LPGYjZuAr71iXQ4hkOWgI9%2BbgdZCCqC%2FgcEtA%2BWng%2F5WFwj%2BddMwT5%2FJyQHnDRbKDLebbvzQn0B0lWD2rg0cDqusXTn6oSO9hmL1uJa1lYKp8GYP4q8f%2BvMdohCqQ9ym5zPEV38yz6VG8uCDc5P4tmxcoHPHT187M9XOT%2BNtx96z1yGun9Rn3SkhTbLc0s5kOwo%2B2jXeCGwsrMP%2BVQZQHi7LipPSeW5VeidUyTADtTfxr5rnBMYqa3chljfWh73g6I0VtGhPvF%2Fr2qxCiLImuOzRW2bNjMZkqn6PcWB3dmplfJzDkkYQ8lCiTGVHh2oEidNWHNVmLObf3cXD7arJgs%2FPRnQWMj22%2Fw5GQimSC6IlfZ92%2B7L4E6IgVO6D0BPdHyvgIkmYZ44KLvqaU85socaCzQkZ2UcZmeV%2BE11UT80AnXa6mAhJ2RDOeBTcphSjZYbJNn7OErHeMrEFHkn1F%2BJkS8yyGO08a%2F%2B0UyUDQjuIeB5fCloThctAWNyJiO%2BL%2F7p5y9bfc6%2FtXM%2FGiTeSVL%2Fjiew1SadjvQ5ad32NxdIvDo8aulIiEN66HbOGJOa4doQqBxxmc3jtmjvdmco8PusrAYyQRwkt1jY2L6%2FqyNZ0m8QiCqm5ewu1cNwwVJfXI6sIfOxZdHZf5xjYtsCZPHq5cgpkF%2FhtmYzCK05V1mnfwEeRJ5p&X-Amz-Algorithm=AWS4-HMAC-SHA256&X-Amz-Date=20231226T210000Z&X-Amz-SignedHeaders=host&X-Amz-Expires=10800&X-Amz-Credential=ASIAUMDY6U444EJKJP5C%2F20231226%2Fus-gov-west-1%2Fs3%2Faws4_request&X-Amz-Signature=c0187abb25cba3dc9e35d7b2458dd3328d9292f3548bfcc9146dde99f742ff39) | **SM (MHV):**   - <https://www.myhealth.va.gov/mhv-portal-web/home>   **VA Prescription Refill (MHV):**   - [Managing Your Prescription Refills Online - My HealtheVet - My HealtheVet (va.gov)](https://www.myhealth.va.gov/managing-your-prescription-refills)   **Rx Refill App:**   - [Rx Refill \| VA Mobile](https://mobile.va.gov/app/rx-refill) |
| **9.2 Refill & Track Medication** | **Summary #43^1,4^:** The Veteran can request refill of medications 3 different ways: Requests for more refills can be sent to the provider using **SM (MHV)** or- if there are already refills available, they can order them through **VA Prescriptions Refill (MHV)** or use the **Rx Refill app.** | - [498237 (amazonaws.com)](https://learn-us-gov-west-1-prod-fleet01-xythos.s3.us-gov-west-1.amazonaws.com/612eabb1bae6a/498237?response-cache-control=private%2C%20max-age%3D10800&response-content-disposition=inline%3B%20filename%2A%3DUTF-8%27%27VA%2520Prescription%2520Refills%2520and%2520COVID-19.pdf&response-content-type=application%2Fpdf&X-Amz-Security-Token=FwoDYXdzEJP%2F%2F%2F%2F%2F%2F%2F%2F%2F%2FwEaDFx%2FNVtAwHATXzcqniLlBE3diRWWSiKCHkP7isSq0G%2FFyaEDzzByq82ALTS413TW%2Fqwpt77gsKBXxfzgawaUbH2x4s8xOxk4gling%2BnV6BQSLnOcXF71r7KnUSGPgjktE0r3fp5iO1nCcfehw%2B3JU58MpFmS3dFzbiIuRFBDGMWq8m1HeHYFag5ncBwkH96bBVaQMo39kQu13pJfzNGDwDsb0U%2BVIXTwruxZGfmbc90shERXh1bI4LPGYjZuAr71iXQ4hkOWgI9%2BbgdZCCqC%2FgcEtA%2BWng%2F5WFwj%2BddMwT5%2FJyQHnDRbKDLebbvzQn0B0lWD2rg0cDqusXTn6oSO9hmL1uJa1lYKp8GYP4q8f%2BvMdohCqQ9ym5zPEV38yz6VG8uCDc5P4tmxcoHPHT187M9XOT%2BNtx96z1yGun9Rn3SkhTbLc0s5kOwo%2B2jXeCGwsrMP%2BVQZQHi7LipPSeW5VeidUyTADtTfxr5rnBMYqa3chljfWh73g6I0VtGhPvF%2Fr2qxCiLImuOzRW2bNjMZkqn6PcWB3dmplfJzDkkYQ8lCiTGVHh2oEidNWHNVmLObf3cXD7arJgs%2FPRnQWMj22%2Fw5GQimSC6IlfZ92%2B7L4E6IgVO6D0BPdHyvgIkmYZ44KLvqaU85socaCzQkZ2UcZmeV%2BE11UT80AnXa6mAhJ2RDOeBTcphSjZYbJNn7OErHeMrEFHkn1F%2BJkS8yyGO08a%2F%2B0UyUDQjuIeB5fCloThctAWNyJiO%2BL%2F7p5y9bfc6%2FtXM%2FGiTeSVL%2Fjiew1SadjvQ5ad32NxdIvDo8aulIiEN66HbOGJOa4doQqBxxmc3jtmjvdmco8PusrAYyQRwkt1jY2L6%2FqyNZ0m8QiCqm5ewu1cNwwVJfXI6sIfOxZdHZf5xjYtsCZPHq5cgpkF%2FhtmYzCK05V1mnfwEeRJ5p&X-Amz-Algorithm=AWS4-HMAC-SHA256&X-Amz-Date=20231226T210000Z&X-Amz-SignedHeaders=host&X-Amz-Expires=10800&X-Amz-Credential=ASIAUMDY6U444EJKJP5C%2F20231226%2Fus-gov-west-1%2Fs3%2Faws4_request&X-Amz-Signature=c0187abb25cba3dc9e35d7b2458dd3328d9292f3548bfcc9146dde99f742ff39) | **SM (MHV):**   - [Secure Messaging - My HealtheVet - My HealtheVet (va.gov)](https://www.myhealth.va.gov/secure-messaging-spotlight)   **VA Prescription Refill (MHV):**   - [Managing Your Prescription Refills Online - My HealtheVet - My HealtheVet (va.gov)](https://www.myhealth.va.gov/managing-your-prescription-refills)   **Rx Refill App:**   - [Rx Refill \| VA Mobile](https://mobile.va.gov/app/rx-refill) |
| **10. Resources & Education** | | | |
| **10.1 Obtaining & Organizing Educational Material** | **Summary #44^1,2^:** Provider can obtain educational materials appropriate for the patient veteran through: CPRS linked resources such as **Krames, VISN 8 Nucleus** and other systems such as **SM​ (MHV), MHV, VA YouTube channels,** and **VISTA-Get Well Network for (inpatients).** Material might include written material, videos, animation, and images. | - [Virtual Care Best Practices Community – Virtual Care ... (blackboard.com)](https://vaots.blackboard.com/webapps/blackboard/execute/modulepage/view?course_id=_3330_1&cmp_tab_id=_5023_1&mode=view) | **Krames:**   - [Krames Online - Patient Education Library](https://vanortherncalifornia.kramesonline.com/) - <https://www.kramesondemand.com/Browse.aspx>   **VISN 8 Nucleus:**   - <https://visn8.nucleushealth.com/>   **SM (MHV):**   - [My HealtheVet Resources – My HealtheVet Resources](https://vaots.blackboard.com/webapps/blackboard/execute/modulepage/view?course_id=_3174_1&cmp_tab_id=_4591_1&mode=view) - [Secure Messaging Through My HealtheVet – My HealtheVet Product (va.gov)](https://vaww.va.gov/MYHEALTHEVET/Secure_Messaging.asp)   **YouTube:**   - <https://www.youtube.com/user/DeptVetAffairs?reload=9>   **Micromedex:**   - <https://www.micromedexsolutions.com/micromedex2/librarian/cxnaccess?institution=institution%5eBAYPINESVA%5eBPVA1&action=home>   **Veteran’s Health Library:**   - [MedlinePlus - Health Information from the National Library of Medicine](https://www.medlineplus.gov/) |
|  | **Summary #45^2^:** Provider organizes selected educational material by topics/relevance in folders using **Krames.** | - [Krames Online - Patient Education Library](https://vanortherncalifornia.kramesonline.com/) | **Krames:**   - <https://www.kramesondemand.com/Browse.aspx> |
| **10.2 Delivering Educational Material to Patients** | **Summary #7^1,3^: …** To deliver educational material, a provider can send a Veteran exercise to perform at home using **MS Outlook** or **VCM** at any time across the healthcare continuum. | - [Virtual Care Best Practices Community – Virtual Care ... (blackboard.com)](https://vaots.blackboard.com/webapps/blackboard/execute/modulepage/view?course_id=_3330_1&cmp_tab_id=_5023_1&mode=view) - [Virtual Care Manager – Virtual Care Manager Community (blackboard.com)](https://vaots.blackboard.com/webapps/blackboard/execute/modulepage/view?course_id=_2540_1&cmp_tab_id=_3308_1&mode=view) | **MS Outlook:**   - [Microsoft Outlook (va.gov)](https://www.oit.va.gov/Services/TRM/ToolPage.aspx?tid=5684)   **VCM:**   - <https://mobile.va.gov/sites/default/files/user-manual-vcm.pdf> - [Virtual Care Manager \| VA Mobile](https://mobile.va.gov/app/virtual-care-manager) |
|  | **Summary #8^5^:** You can use the **Live Whole Health app** or **SM (MHV)** to send a Personal Health Inventory Questionnaire to a Veteran. **MS Outlook** (do not reply) can be used in lieu of **SM (MHV)** if Veteran doesn't have an account. | - [Live Whole Health \| VA Mobile](https://mobile.va.gov/app/live-whole-health) - [10-773_PHI_May2020.pdf (va.gov)](https://www.va.gov/WHOLEHEALTH/docs/10-773_PHI_May2020.pdf) | **Live Whole Health App**   - [Circle of Health Graphic](https://mobile.va.gov/sites/default/files/documents/live-whole-health_0.pdf)   **SM (MHV):**   - [My HealtheVet Resources – My HealtheVet Resources](https://vaots.blackboard.com/webapps/blackboard/execute/modulepage/view?course_id=_3174_1&cmp_tab_id=_4591_1&mode=view) - [Secure Messaging Through My HealtheVet – My HealtheVet Product (va.gov)](https://vaww.va.gov/MYHEALTHEVET/Secure_Messaging.asp)   **MS Outlook:**   - [Microsoft Outlook (va.gov)](https://www.oit.va.gov/Services/TRM/ToolPage.aspx?tid=5684) |
|  | **Summary #14^1,3,5^:** Provider follows up with Veteran via **SM (MHV), MS Outlook**, or **telephone** to get updates from the Veteran, assess progress after treatment plan, confirm device was received, and share education material. Communication with the Veteran can be done via **SM (MHV)** or **MS Outlook.** | - [MHV_SM_HCT_User_Manual_August_2023.pdf (va.gov)](https://vaww.va.gov/MYHEALTHEVET/docs/secure_messaging/MHV_SM_HCT_User_Manual_August_2023.pdf) | **SM (MHV):**   - [My HealtheVet Resources – My HealtheVet Resources](https://vaots.blackboard.com/webapps/blackboard/execute/modulepage/view?course_id=_3174_1&cmp_tab_id=_4591_1&mode=view) - [Secure Messaging Through My HealtheVet - My HealtheVet Product (va.gov)](https://vaww.va.gov/MYHEALTHEVET/Secure_Messaging.asp)   **MS Outlook:**   - [Microsoft Outlook (va.gov)](https://www.oit.va.gov/Services/TRM/ToolPage.aspx?tid=5684) |
|  | **Summary #20^2^:** Veteran-based VHR such as **Annie app for Veterans**, can also be used for the Veteran to receive reminders or educational material. | - [Annie App – Annie App Community (blackboard.com)](https://vaots.blackboard.com/webapps/blackboard/execute/modulepage/view?course_id=_2548_1&cmp_tab_id=_3324_1&mode=view) | **Annie App for Veterans:**   - [Annie App for Veterans \| VA Mobile](https://mobile.va.gov/app/annie-app-veterans)   **Annie App for Clinicians**:   - [Annie App for Clinicians \| VA Mobile](https://mobile.va.gov/app/annie-app-clinicians) |
|  | **Summary #28^3,4,5^:** Healthcare staff can prepare for an upcoming virtual appointment with the delivery of educational materials using a dedicated healthcare staff to provide training materials to the Veterans on how to use the virtual platform via **VA & Non-VA YouTube Videos, VA & Non-VA apps…** | - [Prepare for a VA Video Connect Visit \| Veteran Devices and Test Calls \| VHA Telehealth Services Intranet](https://vaww.telehealth.va.gov/pgm/vvc/prepare/test-call.asp) | **VA Apps:**   - [VA App Store \| VA Mobile](https://mobile.va.gov/appstore/)   **YouTube:**   - [U.S. Dept. of Veterans Affairs – YouTube](https://www.youtube.com/user/DeptVetAffairs) |
|  | **Summary #46^2,3^:** Provider uses **SM (MHV), Krames, Veterans Health Library** to refer and/or provide education materials to Veteran. For example, Provider can deliver a medical animation regarding a Veteran’s medical condition for educational purposes. Providers can also use **Annie App** to deliver educational material. | - [VMH132_MHV_Secure_Messaging_Handbook.pdf (va.gov)](https://vaww.va.gov/MYHEALTHEVET/docs/secure_messaging/VMH132_MHV_Secure_Messaging_Handbook.pdf) | **SM (MHV):**   - [My HealtheVet Resources – My HealtheVet Resources](https://vaots.blackboard.com/webapps/blackboard/execute/modulepage/view?course_id=_3174_1&cmp_tab_id=_4591_1&mode=view) - [Secure Messaging Through My HealtheVet – My HealtheVet Product (va.gov)](https://vaww.va.gov/MYHEALTHEVET/Secure_Messaging.asp)   **Krames:**   - [Krames Online - Patient Education Library](https://vanortherncalifornia.kramesonline.com/) - <https://www.kramesondemand.com/Browse.aspx>   **Veteran’s Health Library:**   - [MedlinePlus - Health Information from the National Library of Medicine](https://www.medlineplus.gov/)   **Annie for Providers:**   - [Annie App for Veterans \| VA Mobile](https://mobile.va.gov/app/annie-app-veterans) - [Annie App – Annie App Community (blackboard.com)](https://vaots.blackboard.com/webapps/blackboard/execute/modulepage/view?course_id=_2548_1&cmp_tab_id=_3324_1&mode=view) |
|  | **Summary #48^2,3,5^:** Provider uses **Annie app, SM (MHV), VVC, WebEx, MS Outlook** (blind email or encrypted), and **direct texting** to send education materials to Veterans. Provider uses **Get Well Network (VistA)** to provide in-patient/Veteran education via in-room television. | - [Virtual Care Best Practices Community – Virtual Care ... (blackboard.com)](https://vaots.blackboard.com/webapps/blackboard/execute/modulepage/view?course_id=_3330_1&cmp_tab_id=_5023_1&mode=view) | **Annie for Providers:**   - [Annie For Clinicians \| VA Mobile](https://mobile.va.gov/app/annie-app-clinicians) - [Annie App – Annie App Community (blackboard.com)](https://vaots.blackboard.com/webapps/blackboard/execute/modulepage/view?course_id=_2548_1&cmp_tab_id=_3324_1&mode=view) - [Annie app for Clinicians: Session One – YouTube](https://www.youtube.com/watch?v=h0Qt10Oq8ss)     **SM (MHV):**   - [Secure Messaging Through My HealtheVet - My HealtheVet Product (va.gov)](https://vaww.va.gov/MYHEALTHEVET/Secure_Messaging.asp) - [Secure Messaging Attachments - My HealtheVet - My HealtheVet (va.gov)](https://www.myhealth.va.gov/mhv-portal-web/web/myhealthevet/secure-messaging-attachments)   **VVC:**   - [VA Video Connect (VVC) – VA Video Connect (VVC... (blackboard.com)](https://vaots.blackboard.com/webapps/blackboard/execute/modulepage/view?course_id=_2591_1&cmp_tab_id=_3396_1&mode=view) - [VA Video Connect \| VA Mobile](https://mobile.va.gov/app/va-video-connect)   **WebEx:**   - [Approved Video Technologies \| Following the Pandemic \| VHA Telehealth Services Intranet (va.gov)](https://vaww.telehealth.va.gov/technology/alt/post-pandemic.asp) - <https://www.oit.va.gov/Services/TRM/ToolPage.aspx?tid=13521>   **MS Outlook:**   - <https://www.oit.va.gov/Services/TRM/ToolPage.aspx?tid=5684>   **Get Well Network:**   - <https://www.oit.va.gov/Services/TRM/ToolPage.aspx?tid=8065#:~:text=GetWellNetwork%20is%20an%20interactive%20patient%20communication%20system%20that,tasks%20such%20as%20diet%20selection%20and%20pain%20management> |
| **10.3 Continuing Education & Access Resources for Providers** | **Summary #47^2,5^:** Staff can use **SharePoint, Teams,** and **Outlook** to deliver education materials to internal team members. For example, **Teams** and **Outlook** are used to share educational information with staff, while **SharePoint** can be used to share service updates. | - [Microsoft Teams Community - Home (sharepoint.com)](file:///C:\Users\VHATAMMcMahJ\AppData\Local\Microsoft\Windows\AppData\Local\Microsoft\MicrosoftTeams) - [SharePoint Community of Practice - Home](https://dvagov.sharepoint.com/sites/SharePointCommunityofPractice/SitePages/Home.aspx) - [Training Videos - Home (sharepoint.com)](https://dvagov.sharepoint.com/sites/vhatrainingvideos) | **SharePoint**   - [VA SharePoint Platform - Home](https://dvagov.sharepoint.com/sites/OITSharePointPlatform)   **Teams:**   - [Microsoft Teams (va.gov)](https://www.oit.va.gov/Services/TRM/ToolPage.aspx?tid=14196) - [Microsoft Teams Training Videos](https://support.microsoft.com/en-us/office/microsoft-teams-video-training-4f108e54-240b-4351-8084-b1089f0d21d7)   **Outlook:**   - [Create and send email in Outlook - Microsoft Support](https://support.microsoft.com/en-us/office/create-and-send-email-in-outlook-19c32deb-08b6-4f90-a211-02bc5f77f360) - [Microsoft Outlook (va.gov)](https://www.oit.va.gov/Services/TRM/ToolPage.aspx?tid=5684) - [Create and send email in Outlook - Microsoft Support](https://support.microsoft.com/en-us/office/create-and-send-email-in-outlook-19c32deb-08b6-4f90-a211-02bc5f77f360) - [Microsoft Outlook (va.gov)](https://www.oit.va.gov/Services/TRM/ToolPage.aspx?tid=5684) |
| **11. Documentation** | | | |
| **11.1 Chart Review & Check Records or Laboratory Tests or Imaging** | **Summary #10^3,4^:** You can use **CPRS, telephone, MS Teams, VVC** among clinical team members, including other disciplines, to share information and discuss Veteran care during the session, to prepare for follow-ups and/or data collection, or to alert providers to join a session. Healthcare staff can use these VHRs to manage communication and notes about medications, appointments, or supplies. **CPRS** can be used to track notes throughout the care continuum phases. | - [Virtual Care Best Practices Community – Virtual Care ... (blackboard.com)](https://vaots.blackboard.com/webapps/blackboard/execute/modulepage/view?course_id=_3330_1&cmp_tab_id=_5023_1&mode=view) - [VA Video Connect (VVC) – VA Video Connect (VVC... (blackboard.com)](https://vaots.blackboard.com/webapps/blackboard/execute/modulepage/view?course_id=_2591_1&cmp_tab_id=_3396_1&mode=view) | **CPRS:**   - [CPRS Technical Manual (va.gov)](https://www.va.gov/vdl/documents/Clinical/Comp_Patient_Recrd_Sys_(CPRS)/cprslmtm.pdf)   **MS Teams:**   - [Veterans Affairs Teams Site - Home (sharepoint.com)](https://dvagov.sharepoint.com/sites/MSTeams) - [Microsoft Teams (va.gov)](https://www.oit.va.gov/Services/TRM/ToolPage.aspx?tid=14196) - [Teams Community of Practice (sharepoint.com)](https://dvagov.sharepoint.com/sites/MSTeams/LYNC/SitePages/Teams-Community-of-Practice(1).aspx) - [Bulletins - All Documents (sharepoint.com)](https://dvagov.sharepoint.com/sites/OITEPMOEPMDES/Projects/MSTeams/Bulletins/Forms/AllItems.aspx)   **VVC:**   - <https://vaots.blackboard.com/bbcswebdav/xid-512168_1> - [VA Video Connect \| VA Mobile](https://mobile.va.gov/app/va-video-connect) |
| **11.2 Provider Notes** | **Summary #10^3,4^:** You can use **CPRS, telephone, MS Teams, VVC** among clinical team members, including other disciplines, to share information and discuss Veteran care during the session, to prepare for follow-ups and/or data collection, or to alert providers to join a session. Healthcare staff can use these VHRs to manage communication and notes about medications, appointments, or supplies. **CPRS** can be used to track notes throughout the care continuum phases. | - [Virtual Care Best Practices Community – Virtual Care ... (blackboard.com)](https://vaots.blackboard.com/webapps/blackboard/execute/modulepage/view?course_id=_3330_1&cmp_tab_id=_5023_1&mode=view) - [VA Video Connect (VVC) – VA Video Connect (VVC... (blackboard.com)](https://vaots.blackboard.com/webapps/blackboard/execute/modulepage/view?course_id=_2591_1&cmp_tab_id=_3396_1&mode=view) | **CPRS:**   - [CPRS Technical Manual (va.gov)](https://www.va.gov/vdl/documents/Clinical/Comp_Patient_Recrd_Sys_(CPRS)/cprslmtm.pdf)   **MS Teams:**   - [Veterans Affairs Teams Site - Home (sharepoint.com)](https://dvagov.sharepoint.com/sites/MSTeams) - [Microsoft Teams (va.gov)](https://www.oit.va.gov/Services/TRM/ToolPage.aspx?tid=14196) - [Teams Community of Practice (sharepoint.com)](https://dvagov.sharepoint.com/sites/MSTeams/LYNC/SitePages/Teams-Community-of-Practice(1).aspx) - [Bulletins - All Documents (sharepoint.com)](https://dvagov.sharepoint.com/sites/OITEPMOEPMDES/Projects/MSTeams/Bulletins/Forms/AllItems.aspx)   **VVC:**   - <https://vaots.blackboard.com/bbcswebdav/xid-512168_1> - [VA Video Connect \| VA Mobile](https://mobile.va.gov/app/va-video-connect) |

* Indicates an established best practice

^1^Cardiology

^2^Education

^3^Physical Medicine and Rehabilitation (PM&R)

^4^Spinal Cord Injury (SCI)

^5^Whole Health (WH)
