# Supplementary material for: Super Users’ Reported Best Practices for Coordinating Proactive Integrated Use of Virtual Health Care Resources: Prospective Concurrent Mixed Methods Human-Centered Design Study
Source: J Med Internet Res. 2025 Nov 14;27:e81414. doi: 10.2196/81414 (PMC12663705; doi:10.2196/81414)
Supplement: Multimedia Appendix 10 [file jmir_v27i1e81414_app10.pdf]

Use this grid to map VHR use throughout a clinical workflow. Best practice use identified across 5 service lines *(See legend to the right)*.

RED = De-implement

* Current Practice

Super User Integrated VHR Use Best Practice Matrix (N=60)

Mapping Best Practice VHR use by service across 11 clinical care activities and 43 sub-tasks.

Service Line Legend

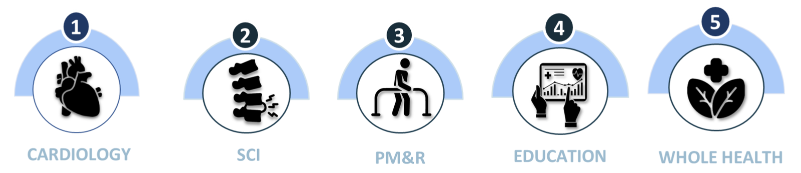

Virtual Healthcare Resource / Task	Scheduling Medical Appointment	Sending Appointment Reminder to Veteran	Sharing Virtual Appointment Link/Access	Preparing for Virtual Appointment	Pre-Appointment Huddles	Follow-up Appointment	Dedicated Resources For Device Consult/ Troubleshooting/ Responding To Alert	Prepare for Visit/Assess Technology	Provide Troubleshooting	Provide Training/ Support	Obtaining Digital Equipment	Provider, Veteran, Non-Veteran Communication	Internal/External Interdisciplinary Coordination & Communication	Veteran-Initiated Communication	Provider-Initiated Communication	Adaptive Communication with Veteran	Preparation for Communication with Veterans & Caregivers	Facilitating Virtual Communication & Access for In-patient	Check in/Triage	Take History	Conducting Virtual Assessment	Providing Treatment/ Recommendations	Conducting Group Appointment*	Follow-Up	Request Labs/ Tests/ Imaging*	Deliver Labs/ Tests/Imaging Results*	Consult for Device Issuance	Provider Referrals for Care*	Referrals for Technology*	Program Feedback/ Evaluation*	Documenting Data Summaries	Outcome Measures	Consult for PGHD Device Issuance*	Track PGHD, Record Vitals & Monitor Health Indices	Measure PGHD, Vitals & Health Indices	Veteran-Based VHR	Requesting RX/ Treatment By Veteran	Refill & Track Medication	Obtaining & Organizing Educational Material	Delivering Educational Material to Patients	Continuing Education and Access Resources for Providers	Chart Review & Check Records/ Labs/Imaging	Provider Notes
Activity	1 Appointment Management						2 Patient & Staff Technology Resource					3 Communication & Referrals							4 Patient Care Delivery						5 Labs/Tests Management		6 Referral Management			7 Data Collection Management			8 PGHD Management				9 Medication Management		10 Resources & Education			11 Documentation	
3D Camera																																										
Alivecor (app)																																										
Annie App for Clinicians																																										
Annie App for Veterans																																										
Blood Pressure Monitor (peripheral device)																																										
Care Assessment Need (CAN) Risk Assessment (CPRS)																																										
CBT-I Coach (app)																																										
Cisco Jabber																																										
Consults (CPRS)																																*									
Computerized Patient Record System (CPRS)																								*																	
CPT Coach (app)																																										
Digital Scale (peripheral device)																																										
Direct Texting																																										
Doximity																																										
Excel (Microsoft Suite)																											*														
FitBit/Apple Watch																																*									
Flags (CPRS)																																										
Get Well Network (VistA)																																										
Glucometer																																										
In-Patient Blood Pressure Machine (CPRS)																																										
In-Patient Vital Machine (CPRS)																																										
Insomnia Coach (app)																																										
iPad																																*									
KRAMES/KRAMES On Demand																																										
Labs & Tests (CPRS)																																										
LEAF																																										
Live Whole Health App																																*									
My HealtheVet (MHV)																																										
Notes & Alerts (CPRS)																																										
Outlook (Microsoft Suite)																																										
Personal Health Inventory Questionnaire																																										
PowerPoint (Microsoft Suite)																																										
PTSD Coach (app)																																										
Pulse Oximeter (peripheral device)																																										
Qualtrics																																										
REDCap																													*												
Remote Order Entry System (ROES) (CPRS)																													*			*									
RX Refill App (MHV)																												*													
Secure Messaging (MHV)																																										
Share My Health Data App																									*																
SharePoint (Microsoft Suite)																																										
Stethoscope (peripheral device)																																										
Survey Monkey																																										
Teams (Microsoft Suite)																																										
Telephone																																										
Uniform Resource Locator (URL) Generator																																										
VA & Non-VA apps																																										
VA & Non-VA YouTube Videos																																										
VA Prescriptions Refill (MHV)																																										
VA Video Connect (VVC/VVC Now)																																										
VA Virtual Toolkit Prescription Pad																																										
VetLink Kiosk																																*									
Virtual Care Manager (VCM)																																										
Virtual Tool Rx (app)																																										
VISN 8 Nucleus (CPRS)																																										
WebEx																																										
Weight Management Scale (Peripheral device)																																										
Word (Microsoft Suite)																																										
Zio Patch																																										
Zoom																																										
