# Supplementary material for: Super Users’ Reported Best Practices for Coordinating Proactive Integrated Use of Virtual Health Care Resources: Prospective Concurrent Mixed Methods Human-Centered Design Study
Source: J Med Internet Res. 2025 Nov 14;27:e81414. doi: 10.2196/81414 (PMC12663705; doi:10.2196/81414)
Supplement: Supplementary file 9 [file jmir_v27i1e81414_app4.docx]

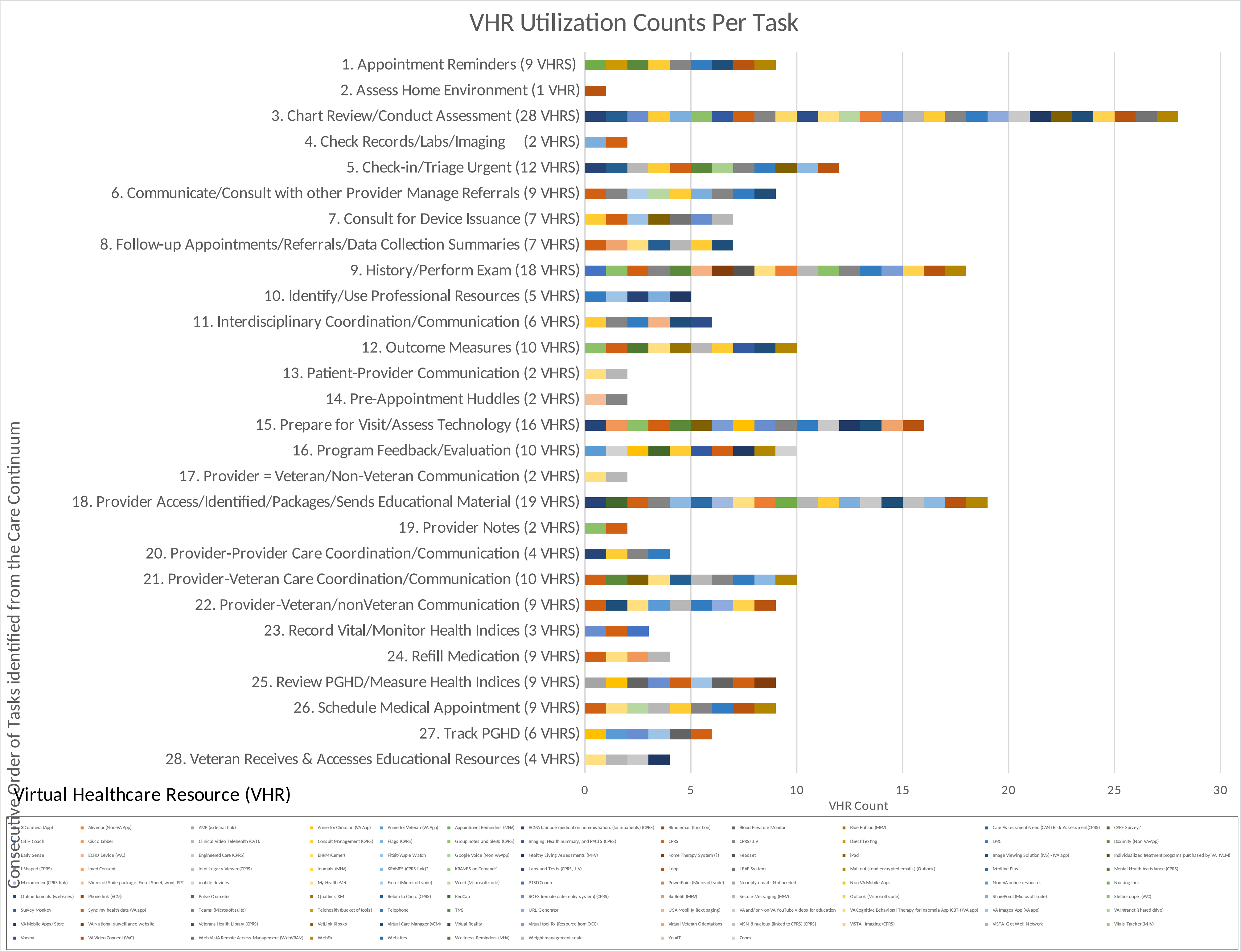
VHR Utilization Counts Per Task
1. Appointment Reminders (9 VHRS)
2. Assess Home Environment (1 VHR)
3. Chart Review/Conduct Assessment (28 VHRS)
4. Check Records/Labs/Imaging (2 VHRS)
5. Check-in/Triage Urgent (12 VHRS)
6. Communicate/Consult with other Provider Manage Referrals (9 VHRS)
7. Consult for Device Issuance (7 VHRS)
8. Follow-up Appointments/Referrals/Data Collection Summaries (7 VHRS)
9. History/Perform Exam (18 VHRS)
10. Identify/Use Professional Resources (5 VHRS)
11. Interdisciplinary Coordination/Communication (6 VHRS)
12. Outcome Measures (10 VHRS)
13. Patient-Provider Communication (2 VHRS)
14. Pre-Appointment Huddles (2 VHRS)
15. Prepare for Visit/Assess Technology (16 VHRS)
16. Program Feedback/Evaluation (10 VHRS)
17. Provider = Veteran/Non-Veteran Communication (2 VHRS)
18. Provider Access/Identified/Packages/Sends Educational Material (19 VHRS)
19. Provider Notes (2 VHRS)
20. Provider-Provider Care Coordination/Communication (4 VHRS)
21. Provider-Veteran Care Coordination/Communication (10 VHRS)
22. Provider-Veteran/nonVeteran Communication (9 VHRS)
23. Record Vital/Monitor Health Indices (3 VHRS)
24. Refill Medication (9 VHRS)
25. Review PGHD/Measure Health Indices (9 VHRS)
26. Schedule Medical Appointment (9 VHRS)
27. Track PGHD (6 VHRS)
28. Veteran Receives & Accesses Educational Resources (4 VHRS)
Consecutive Order of Tasks identified from the Care Continuum
Virtual Healthcare Resource (VHR)
0
5
10
15
20
25
30
VHR Count
3D camera (App)
Alivecor (Non-VA App)
AMP (external link)
Annie for Clinician (VA App)
Annie for Veteran (VA App)
Appointment Reminders (MHV)
BCMA barcode medication administration. (for inpatients) (CPRS)
Blind email (function)
Blood Pressure Monitor
Blue Button (MHV)
Care Assessment Need (CAN) Risk Assessment(CPRS)
CARF Survey?
CBT-I Coach
Cisco Jabber
Clinical Video Telehealth (CVT).
Consult Management (CPRS)
Flags (CPRS)
Group notes and alerts (CPRS)
Imaging, Health Summary, and PACTS (CPRS)
CPRS
CPRS/JLV
Direct Texting
DMC
Doximity (Non VA-App)
Early Sense
ECHO Device (VVC)
Engineered Care (CPRS)
EHRM (Cerner)
FitBit/Apple Watch
Google Voice (Non VA-App)
Healthy Living Assessments (MHV)
Home Therapy System (?)
Headset
iPad
Image Viewing Solution (IVS) - (VA app)
Individualized treatment programs purchased by VA. (VCM)
I-Shaped (CPRS)
Imed Consent
Joint Legacy Viewer (CPRS)
Journals (MHV)
KRAMES (CPRS link)?
KRAMES on Demand?
Labs and Tests (CPRS, JLV)
Loop
LEAF System
Mail out (send encrypted emails) (Outlook)
Medline Plus
Mental Health Assistance (CPRS)
Micromedex (CPRS link)
Microsoft Suite package- Excel Sheet, word, PPT
mobile devices
My HealtheVet
Excel (Microsoft suite)
Word (Microsoft suite)
PTSD Coach
PowerPoint (Microsoft suite)
No reply email - Not needed
Non-VA Mobile Apps
Non-VA online resources
Nursing Link
Online Journals (websites)
Phone link (VCM)
Pulse Oximeter
Qualtrics XM
Return to Clinic (CPRS)
RedCap
ROES (remote order entry system) (CPRS)
Rx Refill (MHV)
Secure Messaging (MHV)
Outlook (Microsoft suite)
SharePoint (Microsoft suite)
Stethoscope (VVC)
Survey Monkey
Sync my health data (VA app)
Teams (Microsoft suite)
Telehealth (bucket of tools)
Telephone
TMS
URL Generator
USA Mobility (text,paging)
VA and/or Non-VA YouTube videos for education
VA Cognitive Behavioral Therapy for insomnia App (CBTI) (VA app)
VA Images App (VA app)
VA Intranet (shared drive)
VA Mobile Apps/Store
VA National surveillance website
Veterans Health Library (CPRS)
VetLink Kiosks
Virtual Care Manager (VCM)
Virtual Reality
Virtual tool Rx (Resource from OCC)
Virtual Veteran Orientations
VISN 8 nucleus (linked to CPRS) (CPRS)
VISTA - Imaging (CPRS)
VISTA- Get Well Network
Vitals Tracker (MHV)
Vocera
VA Video Connect (VVC)
Web VistA Remote Access Management (WebVRAM)
WebEx
Websites
Wellness Reminders (MHV)
Weight management scale
YourIT
Zoom
